# Direct Synthesis of Benzhydryl-Functionalized 3,4-Dihydropyridin-2-ones from 2-Pyridones and Their Use in the Formation of Bridged δ-Lactams

**DOI:** 10.3390/molecules29225274

**Published:** 2024-11-07

**Authors:** Zofia M. Myk, Jacek G. Sośnicki, Łukasz Struk

**Affiliations:** 1 Department of Organic and Physical Chemistry, Faculty of Chemical Technology and Engineering, West Pomeranian University of Technologyin Szczecin, Al. Piastów 42, 71-065 Szczecin, Poland; zofia.myk@zut.edu.pl (Z.M.M.); lukasz.struk@zut.edu.pl (Ł.S.); 2 Center for Advanced Materials and Manufacturing Process Engineering (CAMMPE), Al. Piastów 42, 71-065 Szczecin, Poland

**Keywords:** 2-pyridones, enelactams, bridged lactams, benzhydryllithium, magnesiates, 3,4-dihydropyridin-2-ones, TIPSOTf, benzomorphanones

## Abstract

A method for the synthesis of C4-benzhydryl-functionalized 3,4-dihydropyridin-2-ones using complementary addition of benzhydryllithium and/or benzhydrylmagnesiate reagents to 2-pyridones, with high regioselectivity triggered by substituents, is described. A partially stereoselective cyclization was successfully demonstrated using TfOH and/or TIPSOTf as Brønsted and Lewis acids, respectively, leading to C6-phenyl-functionalized 7,8-benzomorphanones. It is also shown that the use of functionalized δ-enelactams obtained with an active methoxy-substituted benzyl group at C3 enabled the preparation of a new C3–C6 bridged system within the δ-lactam framework.

## 1. Introduction

A broad spectrum of biological activity and unique reactivity of different nature make 2-pyridones privileged scaffolds in drug discovery [1,2,3] and valuable precursors for synthesizing natural and naturally inspired compounds [4]. 3,4-Dihydropyridin-2-ones—partially saturated derivatives of 2-pyridones, belonging to the group of δ-enelactams—have also attracted much attention as pharmacophores and as functional building units applicable in synthesis, mainly because of the presence of the double bond adjacent to a nitrogen atom [5]. From among the variety of methods of their synthesis that have been reported, the addition of organometallic species to 2-pyridones is of fundamental importance, even though the reactions proceed with different C4 vs. C6 regioselectivity, leading to 3,4- or 3,6-dihydropyridin-2-ones, respectively.

Seebach and co-workers have reported the first regioselective nucleophilic 1,4 addition to 2-pyridone. The sole product—C4-functionalized 3,4-dihydropyridin-2-one—was obtained using a chiral, non-racemic hindered lithium enolate as a nucleophilic reagent [6]. Other reports on the intermolecular addition of organometallic compounds to non-activated 2-pyridones proposed the addition of *n*-BuLi to *N*H 2-pirydones [7] and the addition of phenyl Grignard reagents to *N*R 2-pyridones in the presence of iron salts [8], both leading regioselectively to C6 adduct. In order to extend the range of applicability of the above synthetic methods, we have reported on the nucleophilic addition of lithium magnesiates of type R_3_MgLi to 2-pyridones, permitting straightforward access to allyl [9,10,11,12,13], vinyl [14], and benzyl/benzyl-type [15,16,17,18] functionalized dihydropyridones. However, concerning regioselectivity, it should be noted that in these reactions, in general, *N*-alkyl 2-pyridones yielded mainly C6-addition products. In contrast, *N*H(Li) 2-pyridones led regioselectively to C4 adduct (except for the vinylation reaction). Comparatively, the *N*-phenyl substituent led to an almost equimolar mixture of C6 and C4 regioisomeric adducts, except for the additions of vinylmagnesiate, as in these cases the addition occurred selectively at C6, regardless of the type of substituent at the nitrogen atom.

**Figure 1:**
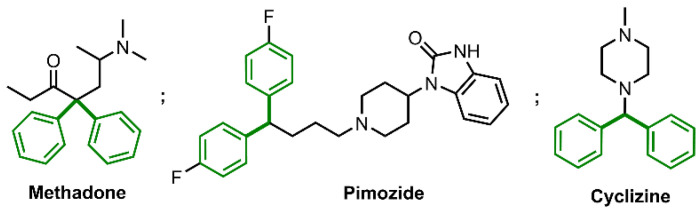
Representative commercial drugs bearing benzhydryl moiety (marked in green): methadone, which is the chemically simplest opioid drug known; pimozide, the first antipsychotic drug for the treatment of Tourette syndrome [20]; and cyclizine, which exhibited anticholinergic and antihistamine activity [21].

Continuing our studies on the synthesis of functionalized 3,4-dihydropyridon-2-ones, we were encouraged to check whether it would be possible to introduce a benzhydryl moiety into a 2-pyridone ring by using benzhydryllithium and benzhydrylmagnesium “ate” reagents, bearing in mind that the benzhydryl group is a common structural motif present in a variety of bioactive compounds, including commercial drugs (Figure 1), and that C4-benzhydryl-functionalized 3,4-dihydropyridin-2-ones could be potentially used in cyclization reactions leading to, i.e., functionalized benzomorphanones [19].

## 2. Results and Discussion

**Scheme 1:**
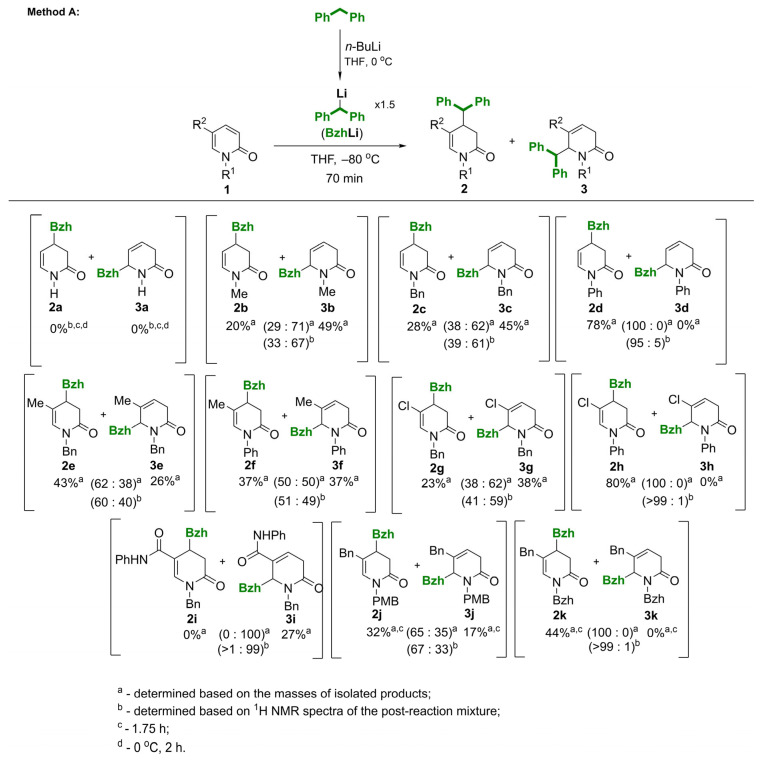
Addition of diphenylmethyllithium (**BzhLi**) to 2-pyridones (Method A).

At the first stage of our study, which aimed at the synthesis of C4-benzhydryl-functionalized 3,4-dihydropyridin-2-ones, diphenylmethyllithium (**BzhLi**), easily generated from diphenylmethane using *n*-BuLi in THF at 0 °C [22], was added. However, in the first attempt, no expected reaction effect between *N*-lithiated 2-pyridone **1a** and **BzhLi** was observed, even when a two-fold molar excess of the latter was used. Following this, the other *N*-substituted 2-pyridones were tested under treatment with **BzhLi** (Scheme 1). After a short optimization, the best reaction conditions were the generation of **BzhLi** at 0 °C for 25 min and its use in the addition reaction in 1.5-fold excess at −80 °C for 70 min. Both steps were performed in THF as a solvent. Under these conditions, *N*-Ph-substituted 2-pyridone **1d** led to a sole C4 adduct **2d** in a 78% yield, while *N*-Me (**1b**) and *N*-Bn (**1c**) led to a mixture of C4 and C6 adducts in the ratios of 33:67 and 61:39, respectively, but with good total yields. The use of *N*-Bn derivative, equipped with a C5–Cl group (**1g**), disclosed the lack of significant changes in the regioselectivity of this addition in comparison to the addition to **1c**, while the presence of a C5-Me substituent (**1e**) shifted the regioselectivity to favor the C4 adduct (Scheme 1). In contrast, comparing C4 vs. C6 adduct distribution for *N*-Ph substrates (**1d** and **1f**, **1h**), the tendency to increase regioselectivity towards the C6 adduct was observed for C5-Me 2-pyridone **1f**. In comparison, for C5–Cl derivative **1h**, the formation of a sole C4 adduct was observed. However, it should be noted that amongst the tested C5-functionalized derivatives in the reaction with **BzhLi**, the presence of a secondary amide group (substrate **1i**) had the most significant impact on obtaining the C6 adduct. Only C6 adduct **3i** was obtained in this case, but in a low yield of 27%.

At the end of this part of the study, it was found that the use of 2-pyridone **1k** with a benzhydryl substituent at the nitrogen atom resulted in the formation of C4 adduct **2k** as the only product. This result indicates that the steric effect of the benzhydryl group is responsible for the full regioselectivity, which could be confirmed by the lower regioselectivity in the case of the addition reaction to derivative **1j** with a smaller *N*-PMB group (Scheme 1).

**Scheme 2:**
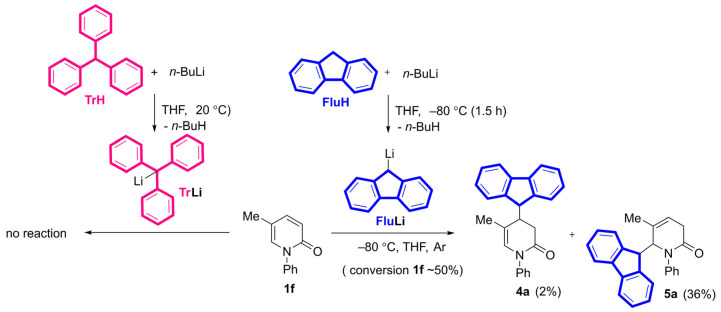
Attempts to add **TrLi** and **FluLi** to 2-pyridone **1f**.

Encouraged by the successful addition of **BzhLi** to *N*-substituted 2-pyridone, we were next prompted to check whether it would be possible to add fluorenyllithium (**FluLi**) and trityllithium (**TrLi**) reagents, which can also be generated by lithiation of fluorene [23] and triphenylmethane [24], respectively. The results are presented in Scheme 2. The reactions tested using 2-pyridone **1f** revealed a lack of reactivity when treated with **TrLi**.

The application of **FluLi** yielded 36% of C6 adduct and only 2% of C4 adduct, with no full conversion. Because these results were not satisfactory regarding the reactivity and regioselectivity, **TrLi** and **FluLi** were not used further in the study.

**Scheme 3:**
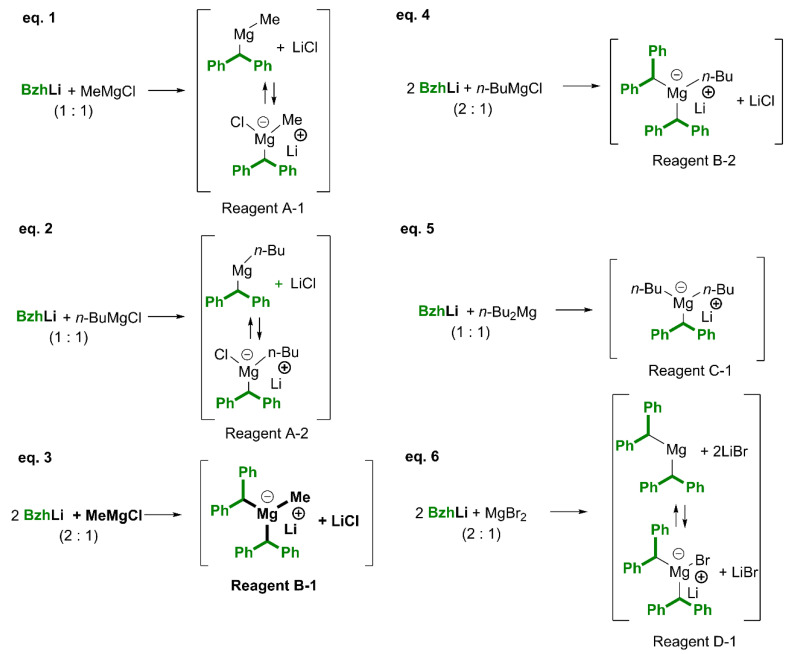
Variety of benzhydrylmagnesiates potentially formed between **BzhLi** and magnesium compounds.

Nevertheless, building the research plan on the previous success of adding benzylmagnesiates to *N*-substituted 2-pyridone [15,16,17,18], we decided to check the reactivity of benzhydrylmagnesiates as a novel addition reagent. Given our promising results using benzylmagnesiates, an attempt to apply benzhydrylmagnesiates was planned as a consistent step in our research. However, in contrast to the previously applied magnesiate preparation method, consisting of mixing of benzyl magnesium chloride (as a donor of the benzyl group) and alkyllithium compounds at a 1:2 molar ratio, in this attempt we mixed an easily generated **BhzLi** reagent with various magnesium compounds, including Grignards, *n*-Bu_2_Mg or MgBr_2_, at a 1:1 and/or 2:1 molar ratio, respectively. The probable structures of lithium magnesiates formed in the mixture in THF at 0 °C are shown in Scheme 3. From among the reagents tested in the reactions with 2-pyridone **1e** in THF at −80 °C (Table 1), the complex **B-1** formed by combining **BzhLi** and MeMgLi at the molar ratio of 2:1 produced the best results in terms of yield and regioselectivity when used in a concentration of 0.08 mol/mL (Table 1, entry 3), which could be further improved slightly by reducing the concentration more than twice (Table 1, entry 5).

Interestingly, good results were also achieved for reagents **C-1** and **D-1**, obtained by mixing **BzhLi** and *n*-Bu_2_Mg or MgBr_2_ at the molar ratio of 1:1 and 2:1, respectively. In these cases, the yields of 82% (**C-1**) and 76% (**D-1**) were attained upon full conversion, but with worse regioselectivity than when reagent **B-1** was used. It is worth noting that the obtained result is better than that achieved using **BzhLi** alone in terms of efficiency and selectivity (see Scheme 1).

**Scheme 4:**
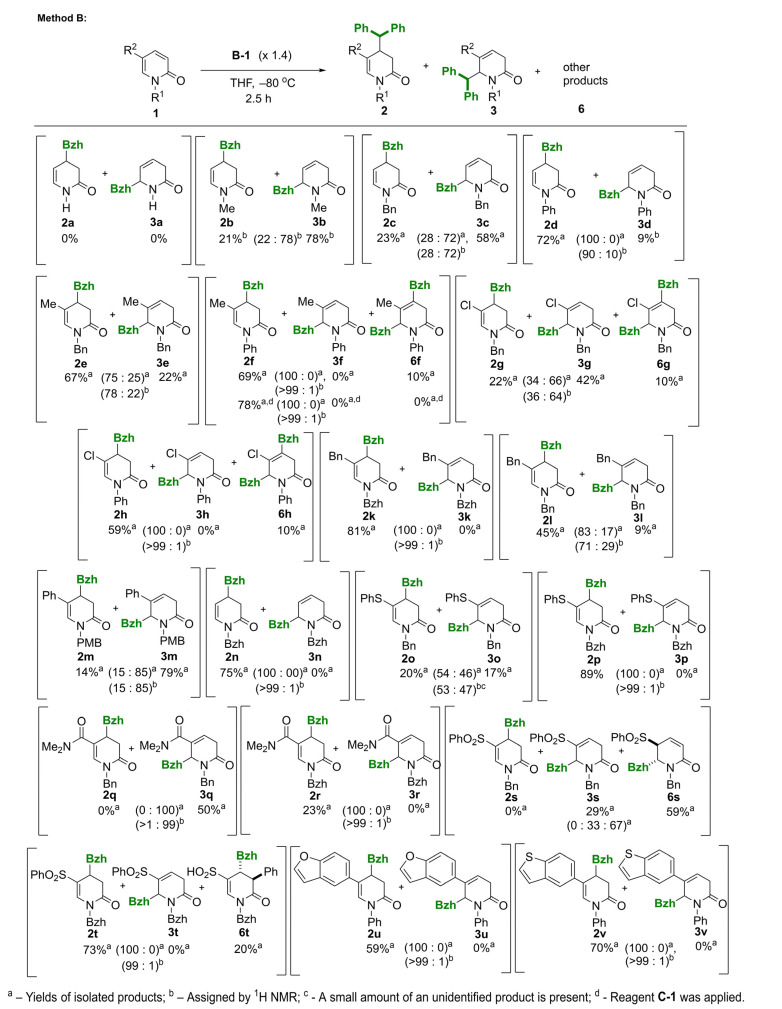
Addition of benzhydrylmagnesiate **B-1** to 2-pyridones in the optimized conditions (Method B).

Having established the best reaction conditions, we evaluated the scope and limitations of reagent **B-1** addition to various *N*- and C5-substituted 2-pyridones (see Scheme 4). Our findings showed that the optimized protocol enabled benzhydrylation only for *N*-substituted 2-pyridones, because the *N*Li 2-pyridone derived from the *N*H derivative remained unchanged. The results demonstrated that the yield and product distributions were dependent on the nature of the substituents. Regarding the substituents on the nitrogen atom, the regioselectivity is generally similar to that observed for the addition of **BzhLi**. Specifically, 2-pyridones with *N*-alkyl groups produced a mixture of 1,4 and 1,6 adducts, whereas *N*-Ph 2-pyridones generally only yielded C4-benzhydrylated products. Similarly to the reaction of **BzhLi** with *N*-Bzh-substituted 2-pyridone **1k** (Scheme 1), the reactions of magnesiate **B-1** with **1k** selectively provided 1,4-addition products, albeit with better yields (see Scheme 4). The other *N*-Bzh derivatives of 2-pyridone (**1n**, **1p**, **1r**, and **1t**) also led regioselectively to C4-addition products, generally in good yields, except for **1r**.

The influence of the substituents at the C5 atom depends on their electronic nature. Generally, it can be assumed that electron-donating substituents favor the increase in the yield of 1,4 adduct, while electron-withdrawing substituents cause the addition to C6 carbon atom to be preferred. However, the reactions with 2-pyridones having a bulky benzhydryl group at the nitrogen atom are exceptions. In the case of these 2-pyridones, regardless of the nature of the substituent at C5 atom, the only product is the C4-benzhydrylated compound, as mentioned earlier. This result indicates a predominant steric effect on the distribution of products. A striking example is the complete regioselectivity reversal in the case of derivatives with 5-dimethylaminocarbonyl groups and with an *N*-benzyl (**1q**) and an *N*-benzhydryl (**1r**) substituent, respectively (Scheme 4).

**Scheme 5:**
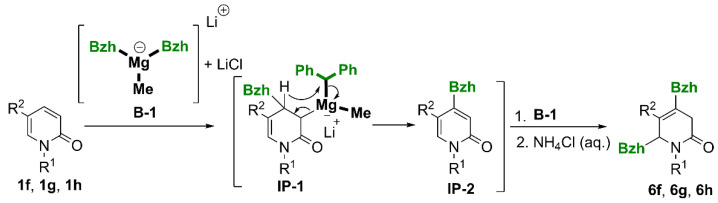
Probable mechanism of formation of by-products **6f**, **6g**, and **6h**.

However, a few differences were noted between the reactions with the addition of **B-1** reagent and those with the use of **BzhLi**. The most significant difference was the unexpected appearance of additional products **6f**, **6g**, and **6h** (each isolated in 10% yield), which contained two benzhydryl groups in the 2-pyridone ring (Scheme 4) when applying **B-1**. Based on their structures, it can be assumed that these products were likely formed as a result of the decomposition of the organomagnesium-addition product **IP-1** through β elimination towards the intermediate 2-pyridone (**IP-2**). This intermediate then takes part in the subsequent addition of excess magnesiate **B-1**, as shown in Scheme 5. Examples of β elimination involving Grignard compounds are described in the literature [25]. However, it should be noted that by using reagent **C-1** in the reaction with **2f**, the formation of product **6f** (Scheme 4) was avoided.

**Scheme 6:**
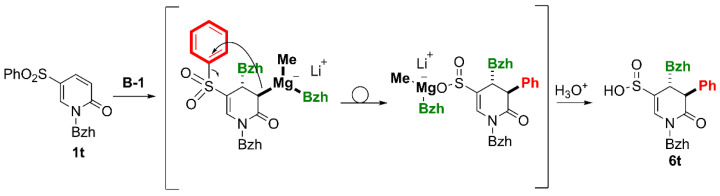
Proposed mechanism of **6t** product formation as a variant of the Truce–Smiles rearrangement.

Furthermore, treating 2-pyridones **1s** and **1t**, equipped with a 5-phenylsulfonyl group, with magnesiate **B-1** led to the formation of the most intriguing by-products. On the one hand, for *N*-Bn 2-pyridone **1s**, 1,4 adduct (**2s**) was absent, while 1,6 adduct (**3s**) was present, along with its **6s** isomer, with a shifted double bond. On the other hand, in the reaction with *N*-Bzh 2-pyridone **1t**, apart from the C4 product **2t** formation (**3t** was absent), the rearrangement product **6t**, with a benzene ring transferred from the phenylsulfone group to the C3 position of the lactam, was formed. This type of rearrangement can be classified as one of the variants of the Truce–Smiles rearrangement, whose main stages are shown in Scheme 6. It should be noted that, although many examples of this type of rearrangement using organolithium compounds are known [26,27], the Truce–Smiles rearrangement initiated by organomagnesium compounds of the “ate” type is described in this work for the first time. Moreover, because of a significant amount of the obtained product (yield of 20%), further studies on this rearrangement are worth continuing within another project.

**Scheme 7:**
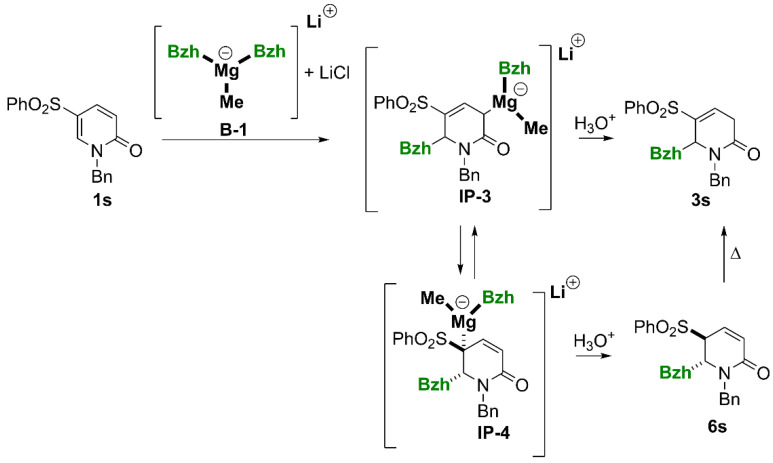
Probable mechanism formation of products **3s** and **6s**.

The origin of compound **6s** is depicted in Scheme 7. The formation of both isomers, **3s** and **6s**, can be explained by the difference in anion stabilization by the carbonyl and sulfonic groups in the intermediate magnesium **IP-3** and **IP-4**, respectively, formed by the addition of **B-1** to 2-pyridone. An additional observation that product **6s** was less stable and transformed into isomer **3s** on heating indicates that product **3s** was thermodynamically more stable than **6s**.

**Scheme 8:**
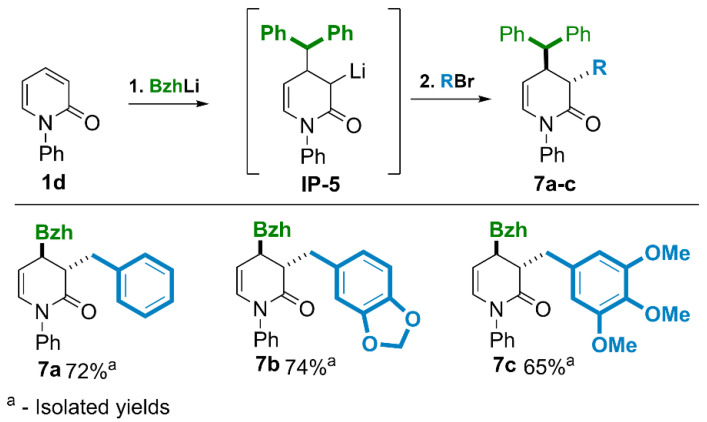
Chemo-, regio- and stereoselective one-pot synthesis of 3,4-*trans*-disubstituted δ-enelactams.

In the next phase of the study on the reaction of introducing a benzhydryl group at the C4 position of the 2-pyridone ring, we attempted to combine this process with the introduction of a benzyl group at the C-3 position using a one-pot method. We used an organolithium reagent (**BzhLi**) and *N*-Ph 2-pyridone **1d**, primarily due to the high yield of addition product **2d** and the complete regioselectivity of the addition at the C4 atom (Scheme 1). Considering the regioselectivity of the addition and the predicted mechanism of the entire transformation, it was expected that the organolithium-addition products would form intermediate product **IP-5** with the lithium atom located at the C3 carbon atom, thanks to stabilization by the carbonyl group. It is worth noting that this reaction sequence involving a benzhydryl nucleophile has not been reported as yet. In the alkylation step, BnBr and benzyl bromides substituted with methoxy groups as electrophiles were used (Scheme 8). As a result, the reaction proceeded in a chemo-, regio- and stereoselective manner, leading to 3,4-*trans*-disubstituted products as sole isomers in good yields (products **7a**, **7b**, and **7c**).

With a relatively wide range of 3,4-dihydropyridones at our disposal, we proceeded to perform cyclization reactions via the acyliminium cation, generated using Brøensted and Lewis acids, in the hope of obtaining 6-phenyl-7,8-benzomorphanone derivatives. A literature survey indicated that such a cyclization of 4-benzyl-3,4-dihydropyridones can be achieved using tin(IV) chloride in a mixture with hydrochloric acid [19]. Additionally, we have recently identified triisopropylsilyltrifluoromethanesulfonate (TIPSOTf) as a potent reagent for cyclization in various δ-enelactams, including 4-benzyl derivatives [5].

**Scheme 9:**
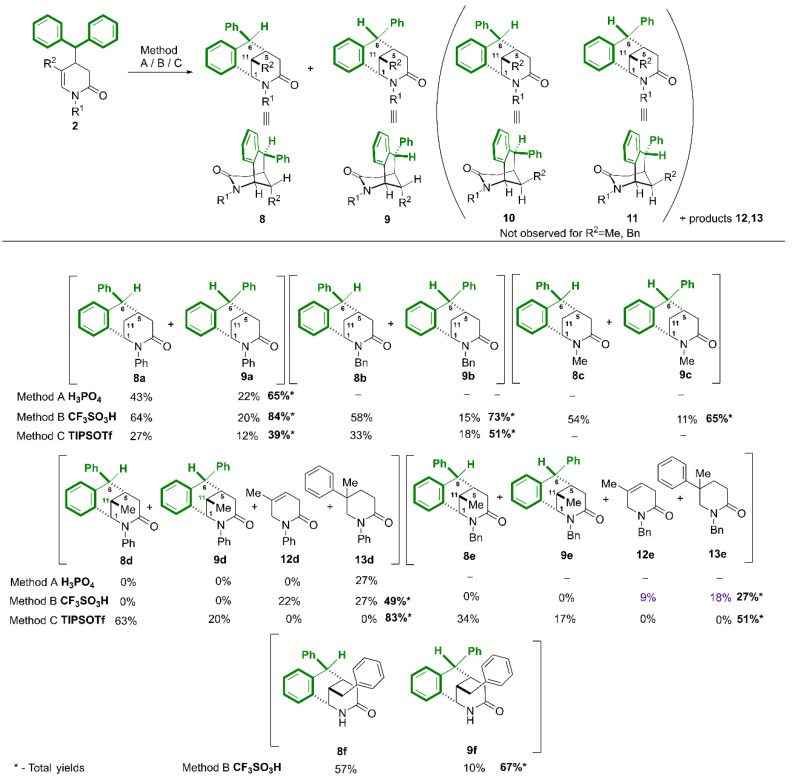
Cyclization of 4-benzhydryl-3,4-dihydropyridin-2-ones upon treatment with H_3_PO_4_ (Method A) and CF_3_SO_3_H (Method B) as Brøensted acids and TIPSOTf (Method C) as a Lewis acid towards 6-phenyl-7,8-benzomorphanones **8** and **9**.

Following a brief screening of the reaction of **2d** with available acids, we selected H_3_PO_4_ and CF_3_SO_3_H as Brøensted acids and TIPSOTf as a Lewis acid. After adjusting the reaction conditions, we found that for H_3_PO_4_, the best conditions included the use of a 60-fold excess of 85% phosphoric(V) acid without solvent at 120 °C and the reaction time of 2–3 h (Method A). When using triflic acid, the use of a 7.5-fold excess and CH_3_CN as a solvent was necessary, and the reaction needed to be carried out for 20–24 h at room temperature (Method B). In the case of TIPSOTf, the best conditions were those used earlier for 4-benzyl-3,4-dihydropyridones [5], i.e., reflux for 24 h of a mixture consisting of **2d**, a 2.5-fold excess of TIPSOTF, and acetonitrile as a solvent (Method C). Under these conditions, for derivative **2d**, products **8a** and **9a** were obtained partially stereoselectively in yields of 43% and 22% (Method A), 64% and 20% (Method B), or 27% and 12% (Method C), respectively (Scheme 9).

Next, we tested the cyclization reactions of *N*-Me-, *N*-Bn-, and C5-H/Me-substituted δ-enelactams using all or selected proposed methods, considering that 5-substituted substrates can theoretically lead to four isomeric products, **8**–**11** (Scheme 9). The results led to a conclusion that triflic acid (TfOH) was a good choice for the cyclization of C5-H-substituted substrates, while C5-Me derivatives decomposed upon treatment with TfOH to yield products **12** and **13**. In contrast, in the case of C5-Me derivatives, the TIPSOT reagent worked better, with no decomposition products observed. This effect is probably a result of two different reaction mechanisms occurring under the influence of TfOH and TIPSOTf, as discussed earlier [5]. It is important to note that derivative **2k** cyclized to *N*H benzomorphanones **8f** and **9f** when treated with CF_3_SO_3_H, with removal of the *N*-Bzh group.

The above data indicate that isomer **8** is the dominant species obtained by all the methods used, when the formation of two isomers **8** and **9** is possible (C5-H substrates) or when two isomers **8** and **9** are formed from the four possible ones (**8**–**11**, C5-Me substrates). We determined their structures through extensive and precise NMR structural analyses of derivatives **8** and **9** (see below) and identified a simple parameter for their easy recognition using ^1^H NMR spectra. Thus, for derivative **8**, the ^1^H NMR signal from the CH-6 carbon atom (labelled as CHα-6) was a singlet, occurring in the range of 4.14–4.32 ppm. Meanwhile, for derivative **9**, the ^1^H NMR signal from the CH-6 carbon atom (labelled as CHβ-6) was a doublet, occurring in the range of 4.57–4.63 ppm, with a coupling constant ^3^*J* ranging from 5.6 to 6.4 Hz.

**Scheme 10:**
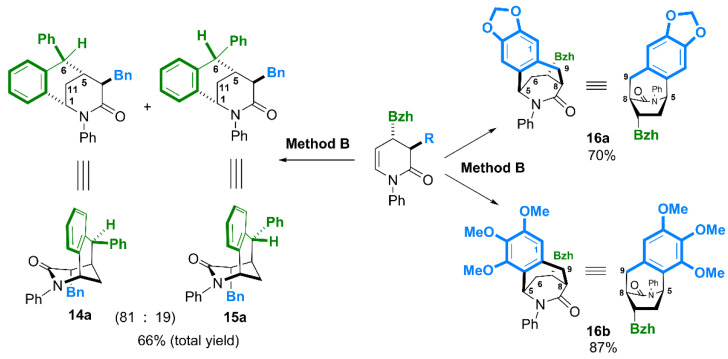
Two directions of cyclization observed in 3,4-disubstituted δ-enelactams **7a**–**7c**, caused by the lack or presence of methoxy groups in the benzyl group.

In the cyclization reaction group, we conducted an interesting test to determine the direction of bridge formation in 3,4-disubstituted derivatives containing C3-methoxybenzyl groups (**7b**–**7c**) in comparison to that in compound **7a**, which has an unsubstituted C3-benzyl group. As expected, the increased nucleophilicity of benzene rings due to the presence of methoxy groups caused this ring to participate in cyclization in derivatives **7b** and **7c**, forming a rare polycyclic system **16** in good yields, while, in contrast, compound **7a**, with an unsubstituted benzene ring, provided 7,8-benzomorphans **14a** and **15a** in the ratio of 81:19 (Scheme 10).

The structures of all compounds were elucidated based on the analyses of 1D NMR (^1^H, ^13^C, and ^13^C-DEPT-135) and 2D NMR (^1^H,^1^H DFQ-COSY, ^13^C,^1^H COSY, ^1^H,^1^H NOESY, ^1^H,^13^C HMQC, and ^1^H,^13^C HMBC) spectra, recorded in CDCl_3_ or toluene-*d*_8_ solutions, and by HRMS analyses. The product configurations were established by comparing the experimental vicinal coupling constants, refined from the ^1^H NMR spectra, with the theoretical values calculated using Haasnoot correlation [28] based on dihedral angles found in PM3-optimized structures [29]. Simultaneously, the proposed configurations were verified by the ^1^H,^1^H NOESY through-space interactions between the juxtaposed hydrogen atoms. The diagnostic NOEs found in the NOESY spectra and vicinal coupling constants for the representative compounds are presented in the Supplementary Materials.

## 3. Materials and Methods

### 3.1. General Chemical Procedures

Melting points were determined on a Boetius hot stage apparatus (Franz Küstner Nachf. KG, HMK, Dresden, Germany). ^1^H,^13^C NMR spectroscopic measurements were performed on a Bruker DPX 400 Avance III HD spectrometer (Bruker BioSpin AG, Ettlingen, Germany) operating at 400.2 and 100.6 MHz. TMS was used as internal standard, and the spectra were acquired for the samples in 5 mm probes at 21 °C. For NMR analyses, the MestReNova (version 12.0.4) program was used. Conformational analyses were performed on the basis of PM3 calculated structures (HyperChem 7.52) and vicinal coupling constants calculated by the MSpin program (version 2.3.4.). For detailed peak assignments, 2D spectra were recorded using standard Bruker software (TopSpin 3.5pl4) (^1^H,^1^H DFQCOSY; ^13^C,^1^H COSY; ^1^H,^13^C HMQC; ^1^H,^1^H NOESY; ^1^H,^13^C HMBC). In the ^1^H,^1^H NOESY spectra, an optimized mixing time varying from 0.7 s to 0.8 s was used. The ^1^H,^13^C HMBC long-range correlations were obtained for *J*_C,H_ = 10 Hz. The standard abbreviations for multiplicities were used (s = singlet, d = doublet, t = triplet, q = quartet, quint = quintet, m = multiplet, sxt = sextet, spt = septet, etc.). Gas chromatography–mass spectrometry (GC-MS) measurements were carried out on an Agilent 7820B GC (Agilent Technologies, Inc. Santa Clara, USA ) system equipped with a mass (Agilent 5977E MSD, Agilent Technologies, Inc. Santa Clara, USA) and an FID detector. HRMS analyses (ESI+) were performed on an Agilent 6546 LC/Q-TOF (Agilent Technologies, Inc. Santa Clara, USA) using acetonitrile or a mixture of acetonitrile/methanol as a solvent.

Reactions in tetrahydrofuran (THF) and acetonitrile (MeCN) were performed under argon in flame-dried flasks, and liquid components were added from a syringe. Anhydrous toluene and THF were purified by distillation over sodium metal under argon prior to use. Anhydrous MeCN was purified by filtration through a pad of Al_2_O_3_. Products were purified by flash column chromatography on silica gel (63–200 μm, Merck, Darmstadt, Germany) using appropriate solvents. Crude post-reaction mixtures were analyzed by GC-MS and ^1^H NMR spectroscopy. *n*-BuLi (2.5 M in hexane), MeMgCl (3.0 M in THF), 2-pyridones **1a** and **1b**, and benzyl bromide were purchased from Aldrich (Merck Group, St. Louis, MO, USA). 2-Methoxypyridine, TIPSOTf, and TfOH were purchased from Fluorochem (Derbyshire, United Kingdom). Diphenylmethane was purchased from Acros Organics (Thermo Fisher Scientific Inc. USA.). (Bromomethyl)-1,2,3-trimethoxybenzene [30], piperonyl bromide [31], methoxy-*N*-phenylnicotinamide [32], 5-(phenylthio)-2-methoxypyridnie [32], 6-methoxy-*N*,*N*-dimethylnicotinamide [32], 2-methoxy-5-phenyl-pyridine [13], and 5-(benzofuran-5’-yl)pyridin-2(1*H*)-one [18] as substrates were obtained according to procedures described earlier.

### 3.2. Synthesis of 4-Benzhydryl-3,4-dihydropyridin-2(1H)-ones (***2***)/6-Benzhydryl-3,6-dihydropyridin-2(1H)-ones (***3***) and By-Products ***6***

Method A (addition of BzhLi to 2-pyridones).

A 25 mL Schlenk flask was charged with 16 mL of anhydrous THF and placed in an ice bath at 0 °C, 0.815 g of diphenylmethane (1.5 equiv., 4.858 mmol) was added, and subsequently 2.04 mL of *n*-BuLi (2.5 M in hexanes, 1.575 equiv., 5.102 mmol) were carefully added dropwise with a syringe and stirred for 25 min at 0 °C. The orange-red solution was then transferred with a syringe to a second 50 mL flask placed in a −80 °C bath in which 2-pyridone (3.239 mmol) had been previously dissolved in 16 mL of anhydrous tetrahydrofuran. The reaction was carried out for 70 min at −80 °C, after which saturated ammonium chloride solution (ca. 5 mL) was added. The solution was warmed to rt and extracted with ethyl acetate (3 × 80 mL), and the organic layer was dried over anhydrous magnesium sulfate, concentrated, and purified by column chromatography using silica gel as the stationary phase.

Method B (addition of magnesiate **B-1** to 2-pyridones).

A 50 mL Schlenk flask was charged with 10.7 mL of anhydrous THF under argon and placed in an ice bath, and then 0.8 g of diphenylmethane (2.8 equiv., 4.756 mmol) and 2 mL of *n*-BuLi (2.94 equiv., 4.993 mmol, 2.5 M solution in hexanes) were added dropwise with a syringe and stirred for 25 min, maintaining the temperature at 0 °C. Then, 0.8 mL of MeMgCl (1.4 equiv., 3.0 M solution in THF) was added and stirred at 0 °C for a further 25 min (a slight color change to carmine red was observed). The solution was then transferred with a syringe to a 100 mL Schlenk flask placed in a −80 °C bath, in which 1.698 mmol of 2-pyridone were dissolved in 36 mL of anhydrous tetrahydrofuran. The reactions were carried out for 2.5–3 h. The rest of the procedure was carried out as in Method A.

(Note: In the syntheses of the compounds listed below, proportional amounts of reagents and solvents were used in relation to the amount of starting 2-pyridone.)

*(4RS)-4-Benzhydry-1-methyl-3,4-dihydropyridin-2(1H)-one* (**2b**).

Yield of 20% (Method A, 0.127 g, from 0.25 g of **1b**); yield of 21% (Method B. Yield was determined by an internal standard method using ^1^H NMR). The crude product, purified by column chromatography (SiO_2_, hexane/ethyl acetate = 3:1), yielded a beige solid, m.p. 103–105 °C. ^1^H NMR (400 MHz, CDCl_3_): δ 2.28 (dd, *J* = 16.2, 10.0 Hz, 1H, CHH-3), 2.46 (dd, *J* = 16.2, 6.4 Hz, 1H, CHH-3), 3.03 (s, 3H, NCH_3_), 3.37 (ddddd, *J* = 11.0, 10.0, 6.4, 3.5, 2.0 Hz, 1H, CH-4), 3.72 (d, *J* = 11.2 Hz, 1H, 4-CH), 4.93 (ddd, *J* = 7.8, 3.5, 0.8 Hz, 1H, =CH-5), 5.95 (dd, *J* = 7.8, 1.8 Hz, 1H, =CH-6), 7.11–7.34 (m, 10H, 2 × C_6_H_5_). ^13^C{H} NMR (100.6 MHz, CDCl_3_): δ 33.47 (NCH_3_), 36.07 (CH-4), 36.42 (CH_2_-3), 56.10 (4-CH), 109.29 (=CH-5), 126.59, 126.70, 127.85 (2C), 128.21 (2C), 128.65 (2C), 128.79 (2C), ArH, 130.50 (=CH-6), 142.35, 142.52 (Ar), 169.16 (C=O). GC–MS (EI, 70 eV) *m*/*z*: 277 (<1) [M^+•^], 167 (41), 165 (30), 152 (13), 110 (100). HRMS (ESI-TOF): *m*/*z* calcd. for C_19_H_20_NO[M + H]^+^, 278.1545; found, 278.1539.

*(6RS)-6-Benzhydryl-1-methyl-3,6-dihydropyridin-2(1H)-one* (**3b**).

Yield of 49% (Method A, 0.31 g from 0.25 g of **1b**); yield of 78% (Method B. Yield was determined by an internal standard method using ^1^H NMR). The crude product, purified by column chromatography (SiO_2_, hexane/ethyl acetate = 3:1), yielded a colorless oil. ^1^H NMR (400 MHz, CDCl_3_) δ 2.08 (dq, *J* = 22.0, 3.4 Hz, 1H, CHH-3), 2.68 (ddd, *J* = 22.0, 4.9, 2.2 Hz, 1H, CHH-3), 2.93 (s, 3H, NCH_3_), 4.41 (d, *J* = 5.5 Hz, 1H, 6-CH), 4.63–4.72 (m, 1H, CH-6), 5.73 (dddd, *J* = 10.1, 5.0, 2.2, 0.7 Hz, 1H, =CH-5), 5.89 (ddd, *J* = 10.1, 4.6, 3.1 Hz, 1H, =CH-6), 7.19–7.35 (m, 10H, 2 × Ph). ^13^C{H} NMR (100.6 MHz, CDCl_3_): δ 32.17 (CH_2_-3), 33.98, NCH_3_, 54.49 (6-CH), 64.68 (CH-6), 124.13 (=CH-4), 125.35 (=CH-5), 126.87, 127.21, 128.23, 128.53, 128.60, 129.74, 138.82, 140.22 (2 × Ph), 168.82 (C=O). GC–MS (EI, 70 eV) *m*/*z*: 277 (<1) [M^+•^], 202 (100), 167 (17), 165 (16), 110 (100). HRMS (ESI-TOF): *m*/*z* calcd. for C_19_H_20_NO[M + H]^+^, 278.1545; found, 278.1539.

*(4RS)-4-Benzhydryl-1-benzyl-3,4-dihydropyridin-2(1H)-one* (**2c**).

Yield of 28% (Method A, 0.114 g from 0.234 g of **1c**); yield of 23% (Method B, 0.138 g from 0.315 g of **1c**). The crude product, purified by column chromatography (SiO_2_, hexane/ethyl acetate = 3:1), yielded a white solid, m.p. = 105–114 °C. ^1^H NMR (400 MHz, CDCl_3_): δ 2.35 (dd, *J* = 16.1, 9.7 Hz, 1H, CHH-3), 2.53 (dd, *J* = 16.1, 6.2 Hz, 1H, CHH-3), 3.26–3.45 (m, 1H, CH-4), 3.69 (d, *J* = 11.3 Hz, 1H, 4-CH), 4.62–4.72 (m, 2H, NCH_2_), 4.93 (dd, *J* = 7.9, 3.6 Hz, 1H, =CH-5), 5.97 (dd, *J* = 7.9, 1.7 Hz, 1H, =CH-6), 7.09–7.41 (m, 15H, ArH). ^13^C{H} NMR (101 MHz, CDCl_3_): δ 36.02 (CH-4), 36.62 (CH_2_-3), 48.79 (NCH_2_), 55.99 (4-CH_2_),109.99 (=CH-5), 126.56, 126.72, 127.57, 127.77, 127.85, 128.15, 128.62, 128.69, 128.81 (ArH), 129.03 (=CH-6), 137.22, 142.27, 142.51 (Ar), 168.84 (C=O). GC–MS (EI, 70 eV) *m*/*z*: 353 (>1) [M^+•^], 186 (100) [M-167(benzhydryl radical)], 165 (15), 91 (97). HRMS (ESI-TOF): *m*/*z* calcd. for C_25_H_24_NO[M + H]^+^, 354.1858; found, 354.1852.

*(6RS)-6-Benzhydryl-1-benzyl-3,6-dihydropyridyn-2(1H)-one* (**3c**).

Yield of 45% (Method A, 0.2 g); yield of 58% (0.346 g, Method B). The crude product, purified by column chromatography (SiO_2_, hexane/ethyl acetate = 3:1), yielded a white solid, m.p. = 124–125 °C. ^1^H NMR (400 MHz, CDCl_3_): δ 2.23 (dq, *J* = 21.2, 2.8 Hz, 1H, CHH-3), 2.82 (ddd, *J* = 21.1, 5.1, 1.8 Hz, 1H,CHH-3), 3.53 (d, *J* = 15.3 Hz, 1H, NCHH), 4.41 (d, *J* = 6.1 Hz, 1H, 6-CH), 4.52–4.63 (m, 1H, CH-6), 5.54 (d, *J* = 15.3 Hz, 1H,NCHH), 5.77 (ddd, *J* = 10.0, 5.1, 2.0 Hz, 1H, =CH-4), 5.85 (ddd, *J* = 10.0, 4.8, 3.1 Hz, 1H, =CH-5), 7.09–7.18 (m, 2H, ArH), 7.19–7.37 (m, 13H, ArH). ^13^C{H} NMR (101 MHz, CDCl_3_): δ 32.63 (CH_2_-2), 47.12 (NCH_2_), 54.54 (6-CH), 60.47 (CH-6), 125.13 (=CH-5), 125.29 (=CH-4), 126.93, 127.19, 127.41, 127.81, 128.30, 128.61, 128.69. 129.70 (ArH), 136.89, 139.10, 140.27 (Ar), 169.25 (C=O). GC–MS (EI, 70 eV) *m*/*z*: 353 (<1) [M^+•^], 186 (76) [M^+•^-167(benzhydryl radical)], 165 (20), 91 (100). HRMS (ESI-TOF): *m*/*z* calcd. for C_25_H_24_NO[M + H]^+^, 354.1858; found, 354.1852.

*(4RS)-4-Benzhydryl-1-phenyl-3,4-dihydropyridin-2(1H)-one* (**2d**).

Yield of 78% (3.088 g, Method A, from 2 g of **1d**); yield of 72% (0.339 g, Method B, from 0.29 g of **1d**). The crude product, purified by column chromatography (SiO_2_, hexane/ethyl acetate = 4:1), yielded a pink solid, m.p. = 100–106 °C. ^1^H NMR (400 MHz, CDCl_3_): δ 2.49 (dd, *J* = 15.9, 9.9 Hz, 1H, CHH-3), 2.65 (ddd, *J* = 15.9, 6.1, 1.0 Hz, 1H, CHH-3), 3.41–3.59 (m, 1H, CH-4), 3.81 (d, *J* = 11.1 Hz, 1H, 4-CH), 5.09 (ddd, *J* = 7.9, 3.6, 1.0 Hz, 1H, =CH-5), 6.23 (dd, *J* = 7.9, 1.8 Hz, 1H, =CH-6), 7.03–7.45 (m, 15H, ArH). ^13^C{H} NMR (101 MHz, CDCl_3_): δ 36.00 (CH-4), 37.49 (CH_2_-3), 56.21 (4-CH), 110.14 (=CH-5), 125.91 (2C), 126.67, 126.77, 127.01, 127.89 (2C), 128.24 (2C), 128.72 (2C), 128.83 (2C), 129.06 (2C), 130.51 (=CH-6), (ArH) 140.29, 142.20, 142.43 (Ar), 168.54 (C=O). GC–MS (EI, 70 eV) *m*/*z*: 339 (<1), [M^+•^], 172 (100) [M^+•^-167(benzhydryl radical)], 144 (8), 77 (10). HRMS (ESI-TOF): *m*/*z* calcd. for C_24_H_22_NO[M + H]^+^, 340.1701; found, 340.1696.

*(4RS)-4-Benzhydryl-1-benzyl-5-methyl-3,4-dihydropyridin-2(1H)-one* (**2e**).

Yield of 43% (0.198 g, Method A, from z 0.25 g of **1e**); yield of 67% (0.246 g, Method B, from 0.2 g of **1e**). The crude product, purified by column chromatography (SiO_2_, hexane/ethyl acetate = 4:1), yielded a white solid, m.p. = 132–134 °C. ^1^H NMR (400 MHz, CDCl_3_): δ 1.04 (d, *J* = 1.5 Hz, 3H, 5-CH_3_), 2.44 (dd, *J* = 16.0, 1.8 Hz, 1H, CHH-3), 2.61 (dd, *J* = 16.0, 6.3 Hz, 1H, CHH-3), 2.88 (ddd, *J* = 11.2, 6.3, 1.8 Hz, 1H, CH-4), 3.69 (d, *J* = 11.2 Hz, 1H, 4-CH), 4.33 (d, *J* = 14.6 Hz, 1H, NCHH), 5.00 (d, *J* = 14.6 Hz, 1H, NCHH), 5.79 (d, *J* = 1.5 Hz, 1H, =CH-6), 6.76–7.01 (m, 2H, ArH), 7.04–7.49 (m, 13H, ArH). ^13^C{H} NMR (101 MHz, CDCl_3_) δ 19.88 (5-CH_3_), 35.81 (CH_2_-3), 41.36 (CH-4), 48.65 (NCH_2_), 53.13 (4-CH), 120.52 (=C-5), 124.57 (=CH-6), 126.35, 126.69, 127.71, 128.11 (2C), 128.22 (2C), 128.29 (2C), 128.37 (2C), 128.71 (2C), 128.75 (2C), ArH), 137.74, 141.61, 143.05 (Ar), 167.83 (C=O). GC–MS (EI, 70 eV) *m*/*z*: 367 (<1), [M^+•^], 200 (68) [M^+•^-167(benzhydryl radical)], 165 (14), 158 (12), 91 (100). HRMS (ESI-TOF) *m*/*z*: [M + H]^+^ calcd. for C_26_H_25_NO, 368.2014; found, 368.2009.

*(6RS)-6-Benzhydryl-1-benzyl-5-methyl-3,6-dihydropyridin-2(1H)-one* (**3e**).

Yield of 26% (0.12 g, Method A, from z 0.25 g of **1e**); yield of 22% (0.08 g, Method B, from 0.2 g of **1e**). The crude product purified by column chromatography (SiO_2_, hexane/ethyl acetate = 4:1) gave yellow oil. ^1^H NMR (400 MHz, CDCl_3_): δ 1.47 (p, *J* = 1.2 Hz, 3H, 5-CH_3_), 2.04 (dq, *J* = 20.7, 2.6 Hz, 1H, CHH-3), 2.70 (ddt, *J* = 20.7, 5.9, 1.0 Hz, 1H, CHH-3), 3.30 (d, *J* = 15.4 Hz, 1H, NCHH), 4.22–4.44 (m, 2H, CH-6, 6-CH), 5.33–5.53 (m, 2H, NCHH, =CH-4), 6.97–7.04 (m, 2H, ArH), 7.21–7.38 (m, 13H, ArH). ^13^C{H} NMR (101 MHz, CDCl_3_) δ 21.45 (5-CH_3_), 32.86 (CH_2_-3), 47.80 (NCH_2_), 54.46 (6-CH), 65.66 (CH-6), 120.89 (=CH-4), 127.11, 127.26 (2C), 127.54 (2C), 128.42 (2C), 128.45 (2C), 128.60 (2C), 129.09 (2C), 129.75 (2C), (ArH), 134.23 (=C-5), 137.07, 138.51, 139.57 (Ar), 170.27 (C=O). GC-MS: *m*/*z* = 367 (<1), [M^+•^], 200 (69) [M^+•^-167(benzhydryl radical)], 165 (18), 91 (100). HRMS (ESI-TOF) *m*/*z*: [M + H]^+^ calcd. for C_26_H_25_NO, 368.2014; found, 368.2009.

*(4RS)-4-Benzhydryl-5-methyl-1-phenyl-3,4-dihydropyridin-2(1H)-one* (**2f**).

Yield of 37% (0.42 g, Method A, from z 0.6 g of **1f**); yield of 69% (Method B, from 0.3146 g of **1e**), yield of 78% (magnesiate C-1 was used). The crude product, purified by column chromatography (SiO_2_, hexane/ethyl acetate = 4:1), yielded a white solid, m.p. = 154–155 °C. ^1^H NMR (400 MHz, CDCl_3_): δ 1.23 (d, *J* = 1.3 Hz, 3 H, 5-CH_3_), 2.59 (dd, *J* = 15.9, 1.6 Hz, 1 H, CHH-3), 2.81 (dd, *J* = 15.9, 6.5 Hz, 1 H, CHH-3), 3.04 (ddd, 1 H, *J* = 10.4, 6.5, 1.6 Hz, CH-4), 4.01 (d, *J* = 10.4 Hz, 1 H, 4-CH), 6.06 (q, *J* = 1.3 Hz, 1 H, =CH-6), 7.16–7.22 (m, 2 H, ArH), 7.24–7.33 (m, 11 H, ArH), 7.38–7.43 (m, 2 H, ArH). ^13^C{H} NMR (100 MHz, CDCl_3_): δ 19.84 (5-CH_3_), 36.59 (CH_2_-3), 41.27 (CH-4), 53.55 (4-CH),120.31 (C-5), 125.65 (ArH), 126.34 (=CH-6), 126.58, 126.69, 126.80, 128.35, 128.42, 128.47, 128.71, 128.96 (ArH), 140.35, 141.38, 142.98 (Ar), 167.50 (C=O). GC-MS (EI, 70 eV) *m*/*z*: 353 (<1), [M^+•^], 186 (100) [M^+•^-167(benzhydryl radical)], 158 (12), 143 (15), 77 (11). HRMS (ESI-TOF) *m*/*z*: [M + H]^+^ calcd. for C_25_H_23_NO, 354.1858; found, 354.1852.

*(6RS)-6-Benzhydryl-5-methyl-1-phenyl-3,6-dihydropyridin-2(1H)-one* (**3f**).

Yield of 37% (0.42 g, Method A, from z 0.6 g of **1f**); yield of 0% (Method B). The crude product, purified by column chromatography (SiO_2_, hexane/ethyl acetate = 4:1), yielded yellow oil. ^1^H NMR (400 MHz, CDCl_3_) δ 1.44 (dt, *J* = 2.6, 1.1 Hz, 3H, CH_3_), 2.38 (dq, *J* = 20.4, 2.6 Hz, 1H, CHH-3), 2.76 (ddt, *J* = 20.4, 5.8, 1.1 Hz, 1H, CHH-3), 4.35 (d, *J* = 5.9 Hz, 1H, 6-CH), 5.03 (dd, *J* = 5.9, 2.6 Hz, 1H, CH-6), 5.58 (dt, *J* = 5.8, 1.7 Hz, 1H, =CH-4), 7.10–7.37 (m, 15H, 3 × C_6_H_5_). ^13^C{H} NMR (101 MHz, CDCl_3_) δ 22.14 (CH_3_), 33.51 (CH_2_-3), 54.66 (6-CH), 71.33 (CH-6), 121.67 (=CH-4), 126.65, 126.67, 127.32, 127.50 (2C), 128.26 (4C), 128.34 (2C), 128.95 (2C), 130.10 (2C), (ArH), 134.29 (=C-5), 137.92, 139.91, 141.79 (Ar), 168.96 (C=O). GC-MS (EI, 70 eV) *m*/*z:* 353 (<1) [M^+•^], 186 (100) [M^+•^-167(benzhydryl radical)], 158 (13), 143 (14). HRMS (ESI-TOF) *m*/*z:* [M + H]^+^ calcd. for C_25_H_23_NO, 354.1858; found, 354.1852.

*(6RS)-4,6-Dibenzhydryl-5-methyl-1-phenyl-3,6-dihydropyridin-2(1H)-one* (**6f**).

Yield of 0% (Method A); yield of 10% (Method B). The crude product, purified by column chromatography (SiO_2_, hexane/ethyl acetate = 4:1), yielded a white solid, m.p. = 128–129 °C. ^1^H NMR (400 MHz, CDCl_3_) δ 1.47 (d, *J* = 2.4 Hz, 3H, CH_3_), 2.42 (dt, *J* = 19.9, 2.4 Hz, 1H, CHH-3), 2.92 (d, *J* = 19.9 Hz, 1H, CHH-3), 4.26 (d, *J* = 7.5 Hz, 1H, 6-CH), 5.08 (dd, *J* = 7.5, 1.7 Hz, 1H, CH-6), 5.27 (s, 1H, 4-CH), 6.95–7.00 (m, 2H, C_6_H_5_), 7.03–7.44 (m, 23H, 5 × C_6_H_5_). ^13^C{H} NMR (101 MHz, CDCl_3_) δ 18.52 (CH_3_), 36.07 (CH_2_-3), 51.76 (4-CH), 55.57 (6-CH), 72.38 (CH-6), 126.41, 126.53, 126.72, 126.82, 127.09, 127.23 (2C), 128.26 (2C), 128.28 (2C), 128.36 (2C), 128.53 (2C), 128.58 (2C), 128.60 (2C), 128.76 (2C), 129.51 (2C), 129.68 (2C), (ArH), 130.35 (C-5), 131.72, 138.81, 139.78, 140.70, 141.53, 141.87 (Ar, C-4), 169.40 (C=O). GC-MS (EI, 70 eV) *m*/*z* = 519 (>1), [M^+•^], 351 (100) [M^+•^-1-167 (benzhydryl radical)], 350 (68), 246 (45), 208 (32), 165 (23), 77 (51). HRMS (ESI-TOF) *m*/*z:* [M + H]^+^ calcd. for C_38_H_34_NO, 520.2640; found, 520.2635.

*(4RS)-4-Benzhydryl-1-benzyl-5-chloro-3,4-dihydropyridin-2(1H)-one* (**2g**).

Yield of 23% (0.08 g, Method A, from 0.2 g of **1g**); yield of 22% (0.15 g, Method B, from 0.373 g of **1g**). The crude product, purified by column chromatography (SiO_2_, hexane/ethyl acetate = 4:1), yielded a white solid, m.p. = 127–131 °C. ^1^H NMR (400 MHz, CDCl_3_) δ 2.66 (dd, *J* = 16.5, 1.9 Hz, 1H, CHH-3), 2.88 (dd, *J* = 16.5, 7.5 Hz, 1H, CHH-3), 3.32 (td, *J* = 8.8, 7.5, 1.9 Hz, 1H, CH-4), 4.00 (d, *J* = 8.8 Hz, 1H, 4-CH), 4.40 (d, *J* = 14.7 Hz, 1H, NCHH), 4.57 (d, *J* = 14.7 Hz, 1H, NCHH), 6.14 (s, 1H, =CH-6), 6.96–7.10 (m, 2H, ArH), 7.12–7.45 (m, 13H, ArH). ^13^C{H} NMR (101 MHz, CDCl_3_) δ 35.32 (CH_2_-3), 43.28 (CH-4), 48.81 (NCH_2_), 52.31 (4-CH), 116.89 (=C-5), 126.62, 126.91, 127.09, 127.96, 128.19 (2C), 128.22 (2C), 128.49 (2C), 128.61 (2C), 128.72 (2C), 128.85 (2C), (ArH), 136.60, 140.41, 141.35 (Ar), 166.59 (C=O). GC-MS (EI 70 eV) *m*/*z:* 387 (<1) [M^+•^], 222 (12), 220 (35) [M^+•^-167(benzhydryl radical)], 167 (49), 165 (23), 152 (12), 91 (100). HRMS (ESI-TOF) *m*/*z*: [M + H]^+^ calcd. for C_25_H_22_ClNO, 388.1468; found, 388.1463.

*(6RS)-6-Benzhydryl-1-benzyl-5-chloro-3,6-dihydropyridin-2(1H)-one* (**3g**).

Yield of 38% (0.133 g, Method A); yield of 42% (0.274 g, Method B). The crude product, purified by column chromatography (SiO_2_, hexane/ethyl acetate = 4:1), yielded a semi-solid. ^1^H NMR (400 MHz, CDCl_3_) δ 1.61 (dt, *J* = 21.1, 2.5 Hz, 1H, CHH-3), 2.63 (ddd, *J* = 21.1, 5.9, 1.0 Hz, 1H, CHH-3), 3.35 (d, *J* = 15.3 Hz, 1H, NCHH), 4.62–4.68 (m, 2H, CH-6, 6-CH), 5.52 (d, *J* = 15.3 Hz, 1H, NCHH), 5.68 (dd, *J* = 5.9, 2.5 Hz, 1H, =CH-4), 6.90 (dd, *J* = 7.3, 2.2 Hz, 2H, ArH), 7.21–7.41 (m, 13H, ArH). ^13^C{H} NMR (101 MHz, CDCl_3_) δ 32.80 (CH_2_-3), 47.71 (NCH_2_), 52.98 (6-CH), 66.58 (CH-6), 122.94 (=CH-4), 127.06, 127.51, 127.66 (2C), 127.79 (ArH), 127.91 (=C-5), 128.28 (2C), 128.37 (2C), 128.62 (2C), 128.70 (2C), 131.03 (2C), (ArH), 135.75, 136.20, 139.54 (Ar), 168.25 (C=O). GC-MS (EI 70 eV) *m*/*z:* 387 (<1) [M^+•^], 222 (12), 220 (36) [M^+•^-167(benzhydryl radical)],167 (47), 165 (21), 152 (11), 91 (100). HRMS (ESI-TOF) *m*/*z:* [M + H]^+^ calcd. for C_25_H_22_ClNO, 388.1468; found, 388.1463.

*(6RS)-4,6-Dibenzhydryl-1-benzyl-5-chloro-3,6-dihydropyridin-2(1H)-one* (**6g**).

Yield of 0% (Method A); yield of 10% (0.095 g, Method B). The crude product, purified by column chromatography (SiO_2_, hexane/ethyl acetate = 5:1), yielded a white solid, m.p. = 171–174 °C. ^1^H NMR (400 MHz, CDCl_3_) δ 1.42 (dd, *J* = 20.5, 2.8 Hz, 1H, CHH-3), 2.66 (d, *J* = 20.5 Hz, 1H, CHH-3), 3.30 (d, *J* = 15.3 Hz, 1H, NCHH), 4.66 (d, *J* = 2.8 Hz, 1H, 6-CH), 4.73 (t, *J* = 2.8 Hz, 1H, CH-6), 5.48 (d, *J* = 15.3 Hz, 1H, NCHH), 5.51 (s, 1H, 4-CH), 6.84–6.95 (m, 4H, ArH), 7.01 (d, *J* = 7.5 Hz, 2H, ArH), 7.16–7.38 (m, 19H). ^13^C{H} NMR (101 MHz, CDCl_3_) δ 35.05 (CH_2_-5), 47.77 (NCH_2_), 52.22 (4-CH), 53.38 (6-CH), 67.14 (CH-6), 124.84 (=C-5), 126.82, 126.94, 127.02, 127.48 (2C), 127.55, 127.66, 128.18 (2C), 128.26 (2C), 128.42 (2C), 128.45 (2C), 128.63 (2C), 128.70 (4C), 129.43 (2C), 130.79 (2C), (ArH), 133.42, 136.03, 136.31, 139.67 (2C), 139.80 (Ar), 169.12 (C=O). GC-MS (EI, 70 eV) *m*/*z*: 553 (<1), [M^+•^], 387 (14) [M^+•^-167 (benzhydryl radical) 385 (30), 384 (18), 165 (11), 91 (100). HRMS (ESI-TOF) *m*/*z*: [M + H]^+^ calcd. for C_38_H_32_ClNO, 554.2251; found, 554.2245.

*(4RS)-4-Benzhydryl-5-chloro-1-phenyl-3,4-dihydropyridin-2(1H)-one* (**2h**).

Yield of 80% (0.269 g, Method A, from 0.185 g of **1h**); yield of 59% (0.377 g, Method B, from 0.349 g of **1h**). The crude product, purified by column chromatography (SiO_2_, hexane/ethyl acetate = 3:1), yielded a white solid, m.p. = 117–119 °C. ^1^H NMR (400 MHz, CDCl_3_) δ 2.88 (dd, *J* = 16.6, 1.6 Hz, 1 H, CHH-3), 3.10 (dd, *J* = 16.6, 8.2 Hz, 1 H, CHH-3), 3.52 (ddd, *J* = 8.2, 6.9, 1.6 Hz, 1 H, CH-4), 4.36 (d, *J* = 6.9 Hz, 1H), 6.38 (s, 1 H =CH-6), 6.90–7.01 (m, 2 H, C_6_H_5_), 7.20–7.37 (m, 13 H, ArH). ^13^C({H} NMR (101 MHz, CDCl_3_) *δ* 35.47 (CH_2_-3), 43.15 (CH-4), 51.88 (4-CH), 116.55 (=C-5), 125.82 (2C), 126.72, 127.26, 127.34, 128.43 (2C), 128.50 (2C), 128.54 (2C), (ArH), 128.82 (=CH-6), 129.02 (2C), 129.32 (2C), 139.43, 140.15, 141, 27 (Ar), 165.99 (C=O). GC-MS (EI 70 eV) *m*/*z:* 373 (1) [M^+•^], 208 (46), 207 (26), 206 (100), 168 (15), 167 (83), 165 (31), 152 (18), 77 (13). HRMS (ESI-TOF) *m*/*z:* [M + H]^+^ calcd. for C_24_H_20_ClNO, 374.1312; found, 374.1306.

*(6RS)-4,6-dibenzhydryl-5-chloro-1-phenyl-3,6-dihydropyridin-2(1H)-one* (**3h**).

Yield of 0% (Method A); yield of 10% (Method B). The crude product, purified by column chromatography (SiO_2_, hexane/ethyl acetate = 4:1), yielded yellow oil. ^1^H NMR (400 MHz, CDCl_3_) δ 1.97 (dd, *J* = 20.2, 2.5 Hz, 1H, CHH-3), 2.86 (d, *J* = 20.2 Hz, 1H, CHH-3), 4.52 (d, *J* = 4.5 Hz, 1H, 6-CH), 5.46 (dd, *J* = 4.5, 2.5 Hz, 1H, CH-6), 5.60 (s, 1H, 4-CH), 6.95–7.37 (m, 25H). ^13^C{H} NMR (101 MHz, CDCl_3_) δ 35.80 (CH_2_-3), 52.33 (4-CH), 54.24 (6-CH), 71.51 (CH-6), 124.86 (=C-5), 126.82, 127.00, 127.05 (2C), 127.14, 127.40 (2C), 128.16 (2C), 128.19 (2C), 128.31 (2C), 128.61 (2C), 128.67 (2C), 129.04 (2C), 129.21 (2C), 129.46 (2C), 129.66 (2C), (ArH), 133.88, 137.39, 138.23, 139.62, 139.70, 140.89 (Ar), 167.89 (C=O). GC-MS (EI 70 eV) *m*/*z:* 539 (<1), [M^+•^], 374 (10), 373 (52), 372 (42), 371 (100), 370 (55), 336 (27), 308 (17), 306 (32), 307 (11), 266 (20), 130 (20), 202 (25), 167 (15), 165 (45), 152 (22), 141 (10), 104 (23), 77 (64), 51 (17). HRMS (ESI-TOF) *m*/*z:* [M + H]^+^ calcd. for C_37_H_30_ClNO, 540.2094; found, 540.2089.

*(6RS)-2-Benzhydryl-1-benzyl-6-oxo-N-phenyl-1,2,5,6-tetrahydropyridine-3-carbox-amide* (**3i**).

Yield of 27% (0.0633 g, Method A, using 0.154 g of **1i**). The crude product, purified by column chromatography (SiO_2_, hexane/ethyl acetate = 1:1), yielded a white solid, m.p. = 206–210 °C. ^1^H NMR (400 MHz, CDCl_3_) δ 2.30 (br s, 3H, NCH_3_), 2.67 (br s, 3H, NCH_3_), 2.86 (d, *J* = 15.4 Hz, 1H, NCHH), 2.96 (ddd, *J* = 20.7, 2.2, 1.6 Hz, 1H, CHH-3), 3.10 (dd, *J* = 20.7, 6.2 Hz, 1H, CHH-3), 4.23 (d, *J* = 9.0 Hz, 1H, 6-CH), 5.08 (d, *J* = 15.4 Hz, 1H, NCHH), 5.17 (dd, *J* = 9.0, 1.6 Hz, 1H, CH-6), 5.92 (dd, *J* = 6.2, 2.2 Hz, 1H, =CH-4), 7.01–7.10 (m, 2H, ArH), 7.17–7.38 (m, 13H, ArH). ^13^C{H} NMR (101 MHz, CDCl_3_) δ 33.03 (CH_3_-2), 34.90 (br, NCH_3_), 38.08 (br, NCH_3_), 49.33 (NCH_2_), 57.29 (6-CH), 62.57 (CH-6), 126.30 (=CH-4), 127.35, 127.38, 127.46, 127.75 (2C), 128.48 (2C), 128.53 (2C), 128.77 (2C), 128.81 (2C), 129.12 (2C), 135.93, 136.63, 139.59, 139.79 (Ar), 168.63 (C=O), 168.69 (C=O). GC-MS (EI 70 eV) *m*/*z*: 472 (<1), [M^+•^], 305 (43) [M^+•^-benzhydryl radical (167)], 91 (100). HRMS (ESI-TOF) *m*/*z*: [M + H]^+^ calcd. for C_32_H_28_N_2_O_2_, 473.2229; found, 473.2224.

*(4RS)-4-Benzhydryl-5-benzyl-1-(4-methoxybenzyl)-3,4-dihydropyridin-2(1H)-one* (**2j**).

Yield of 32% (0.159 g, method A, using 0.32 g of **1i**). The crude product, purified by column chromatography (SiO_2_, hexane/ethyl acetate = 6:1), yielded a white solid, m.p. = 70–72 °C. ^1^H NMR (400 MHz, CDCl_3_) δ 2.11 (dd, *J* = 15.5, 1.7 Hz, 1H, 5-CHH), 2.36 (dd, *J* = 15.9, 2.1 Hz, 1H, CHH-3), 2.43 (dd, *J* = 15.9, 5.7 Hz, 1H, CHH-3), 2.71 (d, *J* = 15.5 Hz, 1H, 5-CHH), 2.80 (ddd, *J* = 11.7, 5.7, 2.1 Hz, 1H, CH-4), 3.65 (d, *J* = 11.7 Hz, 1H, 4-CH), 4.15 (d, *J* = 14.4 Hz, 1H, NCHH), 3.85 (s, 3H, OCH_3_), 5.11 (d, *J* = 14.4 Hz, 1H, NCHH), 5.80 (d, *J* = 1.7 Hz, 1H, =CH-6), 6.85–7.03 (m, 6H, ArH), 7.12–7.30 (m, 11H, ArH), 7.30–7.40 (m, 2H, ArH). ^13^C{H} NMR (101 MHz, CDCl_3_) δ 36.34 (CH_2_-3), 38.52 (CH-4), 39.69 (5-CH_2_), 48.26 (NCH_2_), 53.20 (4-CH), 55.40 (OCH_3_), 114.11 (2C, ArH), 124.54 (=C-5), 126.16 (=CH-6), 126.32, 126.62, 126.72, 128.14 (2C), 128.22 (2C), 128.39 (2C), 128.53 (2C), 128.76 (2C), 128.82 (2C), 129.74 (2C), (ArH), 129.93, 139.29, 141.48, 143.11, 159.32 (Ar), 167.83 (C=O). GC-MS (EI 70 eV) *m*/*z*: 473 (<1) [M^+•^], 306 [M^+•^-167(benzhydryl radical)], 207 (12), 121 (100). HRMS (ESI-TOF) *m*/*z*: [M + H]^+^ calcd. for C_33_H_31_NO_2_, 474.2433; found, 474.2428.

*(6RS)-6-Benzhydryl-5-benzyl-1-(4-methoxybenzyl)-3,6-dihydropyridin-2(1H)-one* (**3j**).

Yield of 17% (0.084 g, Method A). The crude product, purified by column chromatography (SiO_2_, hexane/ethyl acetate = 6:1), yielded brown oil. ^1^H NMR (400 MHz, CDCl_3_) δ 2.00 (d, *J* = 20.7 Hz, 1H, CHH-3), 2.71 (dd, *J* = 20.7, 5.9 Hz, 1H, CHH-3), 2.87 (ddd, *J* = 15.3, 2.4, 1.5 Hz, 1H, 5-CHH), 3.14 (d, *J* = 15.3 Hz, 1H, 5-CHH), 3.18 (d, *J* = 14.9 Hz, 1H, NCHH), 3.78 (s, 3H, OCH_3_), 4.34 (d, *J* = 5.1 Hz, 1H, 6-CH), 4.38 (dd, *J* = 5.1, 1.5 Hz, 1H, CH-6), 5.28 (d, *J* = 14.9 Hz, 1H, NCHH), 5.44 (dd, *J* = 5.8, 1.5 Hz, 1H, =CH-3), 6.68 (d, *J* = 8.4 Hz, 2H, ArH), 6.74–6.84 (m, 4H, ArH), 7.08–7.16 (m, 3H, ArH), 7.26–7.38 (m, 10H, ArH). ^13^C{H} NMR (101 MHz, CDCl_3_) δ 33.03 (CH_2_-3), 40.97 (5-CH_2_), 47.37 (NCH_2_), 54.63 (6-CH), 55.19 (OCH_3_), 63.21, 113.81 (2C), 122.64 (=CH-4), 126.30, 127.18, 127.29, 128.35 (2C), 128.46 (2C), 128.48 (2C), 128.73 (2C), (ArH), 128.77 (=C-5), 129.01 (2C), 129.26 (2C), 129.82 (2C), (ArH), 137.75, 138.01, 138.41, 139.42, 158.68 (Ar), 170.14 (C=O). GC-MS (EI 70 eV) *m*/*z*: 473 (<1) [M^+•^], 306 (8) [M^+•^-167 (benzhydryl radical)], 281 (37), 253 (15), 208 (12), 207 (100), 191 (11), 133 (12), 121 (62), 73 (26). HRMS (ESI-TOF) *m*/*z*: [M + H]^+^ calcd. for C_33_H_31_NO_2_, 474.2433; found, 474.2428.

*(4RS-)-1,4-Dibenzhydryl-5-benzyl-3,4-dihydropyridin-2(1H)-one* (**2k**).

Yield of 44% (0.065 g, Method A, using 0.1 g of **1k**). The crude product, purified by column chromatography (SiO_2_, hexane/ethyl acetate = 8:1), yielded solid, m.p. = 153–155 °C. ^1^H NMR (400 MHz, CDCl_3_) δ 2.07 (dd, *J* = 15.5, 1.7 Hz, 1H, 5-CHH), 2.42 (dd, *J* = 15.9, 1.9 Hz, 1H, CHH-3), 2.54 (dd, *J* = 15.9, 5.9 Hz, 1H, CHH-3), 2.67 (d, *J* = 15.5 Hz, 1H, 5-CHH), 2.81 (ddd, *J* = 11.8, 5.9, 1.9 Hz, 1H, CH-4), 3.63 (d, *J* = 11.8 Hz, 1H, 4-CH), 5.85 (d, *J* = 1.7 Hz, 1H, =CH-6), 6.79–6.87 (m, 2H, ArH), 6.90–6.96 (m, 2H, ArH), 7.12–7.37 (m, 17H, ArH, NCH), 7.39–7.56 (m, 5H, ArH). ^13^C{H} NMR (101 MHz, CDCl_3_) δ 36.60 (CH_3_-2), 38.03 (CH-4), 39.92 (5-CH_2_), 53.22 (4-CH), 58.58 (NCH), 123.94 (=CH-6), 124.35 (=C-5), 126.28, 126.63, 126.73, 127.38, 127.96, 128.12 (4C), 128.22 (2C), 128.36 (2C), 128.49 (2C), 128.53 (2C), 128.72 (2C), 128.76 (2C), 128.80 (2C), 129.24 (2C), (ArH), 138.41, 139.28, 140.06, 141.48, 143.13 (Ar), 168.00 (C=O). GC-MS (EI 70 eV) *m*/*z*: 519 (<1) [M^+•^], 352 (18) [M^+•^-167 (benzhydryl radical], 207 (21), 167 (100), 165 (20). HRMS (ESI-TOF) *m*/*z*: [M + H]^+^ calcd. for C_38_H_34_NO, 520.2640; found, 520.2635.

*(6RS)-4-Benzhydryl-1,5-dibenzyl-3,4-dihydropyridin-2(1H)-one* (**2l**).

Yield of 45% (0.1383 g, Method B, using 0.1 g of **1l**). The crude product, purified by column chromatography (SiO_2_, hexane/ethyl acetate = 8:1), yielded white solid, m.p. = 184–187 °C. ^1^H NMR (400 MHz, CDCl_3_) δ 2.11 (dd, *J* = 15.5, 1.7 Hz, 1H, 5-CHH), 2.38 (dd, *J* = 15.9, 2.1 Hz, 1H, CHH-3), 2.45 (dd, *J* = 15.9, 5.7 Hz, 1H, CHH-3), 2.71 (d, *J* = 15.5 Hz, 1H, 5-CHH), 2.81 (ddd, *J* = 11.6, 5.7, 2.1 Hz, 1H, CH-4), 3.66 (d, *J* = 11.6 Hz, 1H, 4-CH), 4.19 (d, *J* = 14.5 Hz, 1H, NCHH), 5.20 (d, *J* = 14.4 Hz, 1H, NCHH), 5.81 (d, *J* = 1.7 Hz, 1H, =CH-6), 6.87–6.97 (m, 4H, ArH), 7.13–7.29 (m, 11H, ArH), 7.36–7.49 (m, 5H, ArH). ^13^C{H} NMR (101 MHz, CDCl_3_) δ 36.29 (CH_2_-3), 38.52 (CH-4), 39.67 (5-CH_2_), 48.88 (NCH_2_), 53.22 (4-CH), 124.58 (=C-5), 126.22 (=CH-6), 126.33, 126.62, 126.73, 127.82, 128.13 (2C), 128.23 (2C), 128.41 (4C), 128.50 (2C), 128.78 (4C), 128.82 (2C), (ArH), 137.76, 139.27, 141.47, 143.10 (Ar), 167.89 (C=O). GC-MS (EI 70 eV) *m*/*z*: 443 (<1), [M^+•^], 277 (17) [M^+•^-167 (benzhydryl radical)], 276 (77), 91 (100). HRMS (ESI-TOF) *m*/*z*: [M + H]^+^ calcd. for C_32_H_29_ClNO, 444.2327; found, 444.2322.

*(6RS)-6-Benzhydryl-1,5-dibenzyl-3,6-dihydropyridin-2(1H)-one* (**3l**).

Yield of 9% (0.029 g, Method B, using 0.1 g of **1l**). White solid, m.p. = 148–150 °C. ^1^H NMR (400 MHz, CDCl_3_) δ 2.04 (d, *J* = 20.7 Hz, 1H, CHH-3), 2.74 (dd, *J* = 20.7, 5.8 Hz, 1H, CHH-3), 2.89 (dt, *J* = 16.2, 3.0, 2.0 Hz, 1H, 5-CHH), 3.18 (d, *J* = 16.2 Hz, 1H, 5-CHH), 3.23 (d, *J* = 15.7 Hz, 1H, NCHH), 4.35 (d, *J* = 5.1 Hz, 1H, 6-CH), 4.40 (dd, *J* = 5.1, 2.0 Hz, 1H, CH-6), 5.32 (d, *J* = 15.3 Hz, 1H, NCHH), 5.45 (d, *J* = 5.8 Hz, 1H, =CH-4), 6.78–6.89 (m, 4H, ArH), 7.11–7.20 (m, 6H, ArH), 7.24–7.37 (m, 10H, ArH). ^13^C{H} NMR (101 MHz, CDCl_3_) δ 33.03 (CH_2_-3), 41.00 (5-CH_2_), 48.10 (NCH_2_), 54.71 (6-CH), 63.73 (CH-6), 122.64 (=CH-4), 126.42, 127.12, 127.21, 127.34, 127.60 (2C), 128.42 (4C), 128.49 (2C), 128.51 (2C), 128.79 (2C), 129.22 (2C), 129.85 (2C), (ArH), 136.78, 137.83, 137.97, 138.40, 139.45 (Ar, =C-5), 170.19 (C=O). GC-MS (EI 70 eV) *m*/*z*: 443 (<1) [M^+•^], 276 (100) [M^+•^-167(benzhydryl radical)], 91 (86). HRMS (ESI-TOF) *m*/*z*: [M + H]^+^ calcd. for C_32_H_29_NO, 444.2327; found, 444.2322.

*(4RS)-4-Benzhydryl-1-(4-methoxybenzyl)-5-phenyl-3,4-dihydropyridin-2(1H)-one* (**2m**).

Yield of 14% (0.426 g, Method B, using 1.94 g of **1m**). The crude product, purified by column chromatography (SiO_2_, hexane/ethyl acetate = 8:1), yielded a white solid, m.p. = 122–124 °C. ^1^H NMR (400 MHz, CDCl_3_) δ 2.70 (dd, *J* = 16.1, 1.7 Hz, 1H, CHH-3), 2.81 (dd, *J* = 16.1, 6.4 Hz, 1H, CHH-3), 3.74 (ddd, *J* = 9.6, 6.4, 1.7 Hz, 1H, CH-4), 3.81 (d, *J* = 9.6 Hz, 1H, 4-CH), 3.83 (s, 3H, OCH_3_), 4.43 (d, *J* = 14.5 Hz, 1H, NCHH), 4.80 (d, *J* = 14.5 Hz, 1H, NCHH), 6.23 (s, 1H, =CH-6), 6.78–6.83 (m, 2H, ArH), 6.88 (ddd, *J* = 8.0, 4.3, 1.8 Hz, 5H, ArH), 6.91–6.96 (m, 2H, ArH), 7.02–7.11 (m, 3H, ArH), 7.16–7.34 (m, 7H, ArH). ^13^C{H} NMR (101 MHz, CDCl_3_) δ 35.70 (CH_2_-3), 38.89 (CH-4), 48.42 (NCH_2_), 53.18 (4-CH), 55.37 (OCH_3_), 114.19 (2C, ArH), 123.90 (=C-5), 125.85 (2C), 126.06, 126.21 (ArH), 126.31 (=CH-6), 126.82, 127.64 (2C), 128.08 (2C), 128.46 (2C), 128.58 (4C), (ArH), 129.51 (Ar), 129.63 (2C, ArH), 138.81, 141.35, 141.89, 159.32 (Ar), 167.96 (C=O). GC-MS (EI 70 eV) *m*/*z*: 459 (<1) [M^+•^], 292 (15) [M^+•^-167 (benzhydryl radical)], 207 (16), 167 (10), 121 (100). HRMS (ESI-TOF) *m*/*z*: [M + H]^+^ calcd. for C_32_H_29_NO_2_, 460.2277; found, 360.2271.

*(6RS)-6-Benzhydryl-1-(4-methoxybenzyl)-5-phenyl-3,6-dihydropyridin-2(1H)-one* (**3m**).

Yield of 79% (2.41 g, Method B, using 1.94 g of **1m**). The crude product, purified by column chromatography (SiO_2_, hexane/ethyl acetate = 4:1), yielded yellow oil. ^1^H NMR (400 MHz, CDCl_3_) δ 2.17 (dt, *J* = 20.8, 2.4 Hz, 1H, CHH-3), 2.92 (dd, *J* = 20.8, 6.0 Hz, 1H, CHH-3), 3.11 (d, *J* = 15.1 Hz, 1H, NCHH), 3.82 (s, 3H, OCH_3_), 4.15 (d, *J* = 4.8 Hz, 1H, 6-CH), 5.11 (dd, *J* = 4.8, 2.4 Hz, 1H, CH-6), 5.52 (d, *J* = 15.1 Hz, 1H, NCHH), 5.76 (dd, *J* = 6.0, 2.4 Hz, 1H, =CH-4), 6.83–6.96 (m, 6H, ArH), 7.11–7.40 (m, 13H, ArH). ^13^C{H} NMR (101 MHz, CDCl_3_) δ 33.37 (CH_2_-3), 47.46 (NCH_2_), 54.98 (6-CH), 55.33 (OCH_3_), 63.89 (CH-6), 114.05 (2C), 122.28, 126.34 (2C), 126.65, 127.49, 127.54, 128.20 (2C), 128.24 (2C), 128.40 (2C), 128.64 (2C), 128.87 (=C-5), 129.06 (2C), 130.50 (2C), (ArH), 137.75, 139.21, 139.63, 140.24, 158.97 (Ar), 170.08 (C=O). (EI 70 eV) *m*/*z*: 459 (<1) [M^+•^], 458 (3), 292 (15) [M^+•^-167 (benzhydryl radical)], 291 (56), 121 (100), 116 (20), 89 (53), 73 (17), 51 (38). HRMS (ESI-TOF) *m*/*z*: [M + H]+ calcd. for C_32_H_21_NS, 460.2277; found, 460.2271.

*(4RS)-1,4-Dibenzhydryl-3,4-dihydropyridin-2(1H)-one* (**2n**).

Yield of 75% (0.436 g, Method B, using 0.35 g of **1n**). The crude product, purified by column chromatography (SiO_2_, hexane/ethyl acetate = 7:1), yielded transparent oil. ^1^H NMR (400 MHz, CDCl_3_) δ 2.40 (dd, *J* = 16.1, 9.2 Hz, 1H, CHH-3), 2.60 (dd, *J* = 16.1, 6.1 Hz, 1H, CHH-3), 3.25–3.42 (m, 1H, CH-4), 3.68 (d, *J* = 11.5 Hz, 1H, 4-CH), 4.92 (dd, *J* = 8.1, 3.8 Hz, 1H, =CH-5), 5.95 (dd, *J* = 8.1, 1.5 Hz, 1H, =CH-6), 6.52–7.53 (m, 21H, 4 × C_6_H_5_, NCH). ^13^C{H} NMR (101 MHz, CDCl_3_) δ 35.74 (CH-4), 36.85 (CH_2_-3), 55.91 (4-CH), 58.97 (NCH), 109.64 (=CH-5), 126.55, 126.73 (ArH), 126.92 (=CH-6), 127.59, 127.66, 127.88 (2C), 128.13 (2C), 128.56 (4C), 128.60 (4C), 128.63 (2C), 128.81 (2C), (ArH), 139.18, 139.40, 142.23, 142.62 (Ar), 168.80 (C=O). GC-MS (EI 70 eV) *m*/*z*: 429 (<1) [M^+•^], 262 (21) [M^+•^-167 (benzhydryl radical)], 167 (100), 165 (24), 152 (13). HRMS (ESI-TOF) *m*/*z*: [M + H]^+^ calcd. for C_31_H_27_NO, 430.2171; found, 430.2165.

*(4RS)-4-Benzhydryl-1-benzyl-5-(phenylthio)-3,4-dihydropyridin-2(1H)-one* (**2o**).

Yield of 20% (0.05 g, Method B, using 0.155 g of **1o**). The crude product, purified by column chromatography (SiO_2_, hexane/ethyl acetate = 6:1), yielded a white solid, m.p. 168–170 °C. ^1^H NMR (400 MHz, CDCl_3_) δ 2.68 (dd, *J* = 16.4, 2.7 Hz, 1H, CHH-3), 2.74 (dd, *J* = 16.4, 6.2 Hz, 1H, CHH-3), 3.17 (ddd, *J* = 8.9, 6.3, 2.6 Hz, 1H, CH-4), 3.99 (d, *J* = 8.9 Hz, 1H, 4-CH), 4.38 (d, *J* = 14.6 Hz, 1H, NCHH), 4.69 (d, *J* = 14.7 Hz, 1H, NCHH), 6.46 (s, 1H, =CH-6), 6.97 (dd, *J* = 7.6, 1.9 Hz, 2H, ArH), 7.11–7.44 (m, 18H, ArH). ^13^C{H} NMR (101 MHz, CDCl_3_) δ 36.02 (CH_2_-3), 40.44 (CH-4), 48.94 (NCH_2_), 52.71 (4-CH), 115.03 (=C-5), 126.39, 126.48, 126.92, 127.95, 128.08 (2C), 128.27 (2C), 128.50 (2C), 128.55 (2C), 128.56 (2C), 128.75 (2C), 128.86 (2C), 129.11 (2C), 135.29 (=CH-6), 136.22, 136.81, 140.84, 141.86 (Ar), 167.49 (C=O). GC-MS (EI 70 eV) *m*/*z*: 461 (3) [M^+•^], 294 (97) [M^+•^-167 (benzhydryl radical)], 91 (100). HRMS (ESI-TOF) *m*/*z*: [M + H]^+^ calcd. for C_31_H_27_NOS, 462.1892; found, 462.1886.

*(6RS)-6-Benzhydryl-1-benzyl-5-(phenylthio)-3,6-dihydropyridin-2(1H)-one* (**3o**).

Yield of 17% (0.0324 g, Method B, using 0.155 g of **1o**). The crude product, purified by column chromatography (SiO_2_, hexane/ethyl acetate = 6:1), yielded a brown oil. ^1^H NMR (400 MHz, CDCl_3_) δ 1.47 (dt, *J* = 21.2, 2.2 Hz, 1H, CHH-3), 2.68 (ddd, *J* = 21.2, 5.8, 1.2 Hz, 1H, CHH-3), 3.12 (d, *J* = 14.9 Hz, 1H, NCHH), 4.49–4.63 (m, 1H, CH-6), 4.84 (d, *J* = 2.1 Hz, 1H, 6-CH), 5.49 (d, *J* = 14.9 Hz, 1H, NCHH), 5.98 (dd, *J* = 5.8, 2.2 Hz, 1H, =CH-4), 6.81 (dd, *J* = 7.3, 1.7 Hz, 2H, ArH), 6.98–7.10 (m, 6H, ArH), 7.12–7.41 (m, 12H, ArH). ^13^C{H} NMR (101 MHz, CDCl_3_) δ 33.60 (CH_2_-3), 47.66 (NCH_2_), 53.17 (6-CH), 64.28 (CH-6), 126.67, 127.06, 127.37, 127.68, 128.01 (2C), 128.06 (2C), 128.29 (2C), 128.41 (2C), 128.56 (2C), 129.24 (2C), 130.20 (3C), (ArH, =CH-4), 130.91 (Ar), 131.32 (2C, ArH), 132.75, 135.99, 136.74 (Ar), 140.46 (=C-5), 169.11 (C=O). GC-MS (EI 70 eV) *m*/*z*: 461 (3) [M^+•^], 294 (97) [M^+•^-167 (benzhydryl radical)], 91 (100). HRMS (ESI-TOF) *m*/*z*: [M + H]^+^ calcd. for C_31_H_27_NOS, 462.1892; found, 462.1886.

*(4RS)-1,4-Dibenzhydryl-5-(phenylthio)-3,4-dihydropyridin-2(1H)-one* (**2p**).

Yield of 89% (0.387 g, Method B, using 0.3 g of **1p**). The crude product, purified by column chromatography (SiO_2_, hexane/ethyl acetate = 8:1), yielded a white solid, m.p. 63–65 °C. ^1^H NMR (400 MHz, CDCl_3_) δ 2.67 (dd, *J* = 16.2, 2.0 Hz, 1H, CHH-3), 2.78 (dd, *J* = 16.2, 6.8 Hz, 1H, CHH-3), 3.16 (ddd, *J* = 10.0, 6.8, 2.0 Hz, 1H, CH-4), 3.89 (d, *J* = 10.0 Hz, 1H, 4-CH), 6.44 (s, 1H, =CH-6), 6.87–7.02 (m, 2H, ArH), 7.06–7.53 (m, 24H, ArH, NCH). ^13^C{H} NMR (101 MHz, CDCl_3_) δ 36.52 (CH_2_-3), 40.32 (CH-4), 53.10 (4-CH), 59.23 (NCH), 115.32 (=C-5), 126.40, 126.51, 126.89, 127.72, 128.02 (3C), 128.18 (2C), 128.50 (2C), 128.58 (2C), 128.70 (6C), 128.81 (2C), 128.91 (2C), 129.07 (2C), 132.86 (=CH-6), 136.13, 138.22, 139.17, 141.02, 141.93 (Ar), 167.62 (C=O). GC-MS (EI 70 eV): decomposition. HRMS (ESI-TOF) *m*/*z*: [M + H]^+^ calcd. for C_37_H_31_NOS, 538.2205; found, 538.2199.

*(6RS)-2-Benzhydryl-1-benzyl-N,N-dimethyl-6-oxo-1,2,5,6-tetrahydropyridine-3-carboxamide* (**3q**).

Yield of 50% (0.085 g, Method B, using 0.103 g of **1q**). The crude product, purified by column chromatography (SiO_2_, hexane/ethyl acetate), yielded a white solid, m.p. 204–208 °C. ^1^H NMR (400 MHz, CDCl_3_) δ 2.30 (br. s, 3H, NCH_3_), 2.67 (br. s, 3H, NCH_3_), 2.86 (d, *J* = 15.4 Hz, 1H, NCHH), 2.96 (ddd, *J* = 20.6, 2.1, 1.6 Hz, 1H, CHH-5), 3.10 (dd, *J* = 20.6, 6.2 Hz, 1H, CHH-5), 4.23 (d, *J* = 9.1 Hz, 1H, 2-CH), 5.08 (d, *J* = 15.4 Hz, 1H, NCHH), 5.17 (dd, *J* = 9.1, 1.6 Hz, 1H, CH-2), 5.92 (dd, *J* = 6.2, 2.1 Hz, 1H, =CH-4), 7.01–7.11 (m, 2H, ArH), 7.18–7.37 (m, 13H, ArH). ^13^C{H} NMR (101 MHz, CDCl_3_) δ 33.03 (CH_2_-5), 34.90 br. (NCH_3_), 38.08 br. (NCH_3_), 49.33 (NCH_2_), 57.29 (2-CH), 62.57 (CH-2), 126.30 (=CH-4), 127.35, 127.38, 127.46, 127.75 (2C), 128.48 (2C), 128.53 (2C), 128.77 (2C), 128.81 (2C), 129.12 (2C), 135.93, 136.63, 139.59, 139.79 (Ar, =C-3), 168.63 (C=O), 168.69 (C=O). GC-MS (EI 70 eV) *m*/*z*: 424 (<1) [M^+•^], 257 [M^+•^-167] (82), 207 (44), 167 (32), 91 (100). HRMS (ESI-TOF) *m*/*z*: [M + H]^+^ calcd. for C_28_H_28_N_2_O_2_, 425.2229; found, 425.2224.

*(4RS)-1,4-Dibenzhydryl-N,N-dimethyl-6-oxo-1,4,5,6-tetrahydropyridine-3-carboxamide* (**2r**).

Yield of 23% (Method B, using 0.165 g of **1r**). White solid, m.p. = 212–213 °C. ^1^H NMR (400 MHz, CDCl_3_) δ 1.75 (br s, 3H, CH_3_), 2.52 (dd, *J* = 16.3, 1.7 Hz, 1H, CHH-3), 2.32–2.73 (br s, 3H, CH_3_), 2.84 (dd, *J* = 16.3, 6.5 Hz, 1H, CHH-3), 3.75 (d, *J* = 12.3 Hz, 1H, 4-CH), 3.91 (ddd, *J* = 12.3, 6.5, 1.7 Hz, 1H, CH-4), 6.08 (s, 1H, =CH-6), 7.04–7.20 (m, 8H, ArH, NCH), 7.24–7.45 (m, 13H, ArH). ^13^C{H} NMR (101 MHz, CDCl_3_)* δ 35.79 (CH_2_-3), 35.94 (CH-4), 54.54 (4-CH), 58.94 (NCH), 117.44 (=C-5), 126.79, 126.92 (2C), 127.83 (2C), 127.94, 128.05 (2C), 128.18 (2C), 128.30, 128.80 (2C), 128.83 (2C), 128.89 (2C), 128.95 (2C), 129.35 (ArH), 137.53, 140.34, 141.85, 142.35 (Ar), 168.42 (C=O), 169.83 (C=O). (*Note: Signals for N(CH_3_)_2_ groups are invisible). GC-MS (EI 70 eV): decomposition. HRMS (ESI-TOF) *m*/*z*: [M + H]^+^ calcd. for C_34_H_33_N_2_O_2_, 501.2542; found, 501.2537.

*(6RS)-6-Benzhydryl-1-benzyl-5-(phenylsulfonyl)-3,6-dihydropyridin-2(1H)-one* (**3s**).

Yield of 29% (0.0654 g, Method B, using 0.149 g of **1s**). The crude product, purified by column chromatography (SiO_2_, hexane/ethyl acetate, 2:1), yielded a white solid, m.p. 167–176 °C. ^1^H NMR (400 MHz, CDCl_3_) δ 1.26 (dt, *J* = 21.7, 2.0, 1.5 Hz, 1H, CHH-3), 2.67 (dd, *J* = 21.7, 6.3 Hz, 1H, CHH-3), 3.20 (d, *J* = 15.0 Hz, 1H, NCHH), 4.88 (dd, *J* = 1.5, 1.2 Hz, 1H, CH-6 or 6-CH), 4.98 (d, *J* = 1.2 Hz, 1H, CH-6 or 6-CH), 5.42 (d, *J* = 15.0 Hz, 1H, NCHH), 6.46 (d, *J* = 7.6 Hz, 2H, ArH), 6.93 (dd, *J* = 6.3, 2.0 Hz, 1H, =CH-4), 6.94–7.00 (m, 2H, ArH), 7.10 (t, *J* = 7.3 Hz, 1H, ArH), 7.28–7.48 (m, 12H, ArH), 7.55–7.61 (m, 1H, ArH), 7.70–7.77 (m, 2H, ArH). ^13^C{H} NMR (101 MHz, CDCl_3_) δ 32.89 (CH_2_-3), 48.01 (NCH_2_), 54.49, 61.14 (CH-6, 6-CH), 126.97, 127.27, 127.35 (2C), 127.58 (2C), 127.73 (2C), 128.11, 128.40 (2C), 128.59 (2C), 128.86 (2C), 129.48 (2C), 132.37 (2C), 133.80 (ArH), 134.63, 135.76 (Ar), 138.56 (=CH-4), 138.86, 139.45, 140.07 (Ar, =C-5), 167.86 (C=O). GC-MS (EI 70 eV) *m*/*z*: 493 (<1) [M^+•^], 325 (32) [M^+•^-167], 91 (100). HRMS (ESI-TOF) *m*/*z*: [M + H]^+^ calcd. for C_31_H_27_NO_3_S, 494.1790; found, 494.1784.

*(5SR,6RS)-6-Benzhydryl-1-benzyl-5-(phenylsulfonyl)-5,6-dihydropyridin-2(1H)-one* (**6s**).

Yield of 59% (0.1338 g, Method B). The crude product, purified by column chromatography (SiO_2_, hexane/ethyl acetate, 2:1), yielded a white solid, m.p. 180–182 °C. ^1^H NMR (400 MHz, CDCl_3_) δ 2.46 (d, *J* = 14.8 Hz, 1H, NCHH), 3.82 (d, *J* = 6.0 Hz, 1H, CH-5), 4.22 (d, *J* = 10.0 Hz, 1H, 6-CH), 4.65 (dd, *J* = 10.0, 1.5 Hz, 1H, CH-6), 5.02 (d, *J* = 14.8 Hz, 1H, NCHH), 6.20 (ddd, *J* = 9.8, 6.0, 1.5 Hz, 1H, =CH-4), 6.40 (dd, *J* = 9.8, 0.9 Hz, 1H, =CH-3), 6.86–6.93 (m, 2H, ArH), 7.07–7.14 (m, 2H, ArH), 7.15–7.41 (m, 15H), 7.57–7.67 (m, 1H, ArH). ^13^C{H} NMR (101 MHz, CDCl_3_) δ 48.93 (NCH_2_), 55.12 (6-CH), 56.97 (CH-6), 62.35 (CH-5), 127.36 (=CH-4), 127.55, 127.67, 127.90, 128.09 (2C), 128.36 (2C), 128.92 (2C), 128.94 (2C), 129.03 (2C), 129.27 (2C), 129.34 (2C), 130.57 (2C), (ArH), 131.53 (=CH-3), 134.06 (ArH), 135.86, 137.00, 139.59, 139.82 (Ar), 161.43 (C=O). GC-MS (EI 70 eV) *m*/*z*: 493 (<1) [M^+•^], 325 (48) [M^+•^-167], 91 (100). HRMS (ESI-TOF) *m*/*z*: [M + H]^+^ calcd. for C_31_H_27_NO_3_S, 494.1790; found, 494.1784.

*(4RS)-1,4-Dibenzhydryl-5-(phenylsulfonyl)-3,4-dihydropyridin-2(1H)-one* (**2t**).

Yield of 73% (0.0942 g, Method B, using 0.091 g of **1t**). The crude product, purified by column chromatography (SiO_2_, hexane/ethyl acetate, 2:1), yielded a white solid, m.p. 154–156 °C. ^1^H NMR (400 MHz, CDCl_3_) δ 2.68 (dd, *J* = 17.2, 9.3 Hz, 1H, CHH-3), 3.02 (dd, *J* = 17.2, 1.1 Hz, 1H, CHH-3), 3.73 (dud, *J* = 9.3, 4.3, 1.1 Hz, 1H, CH-4), 4.41 (d, *J* = 4.3 Hz, 1H, 4-CH), 6.58–6.65 (m, 2H, ArH), 6.67 (s, 1H,NCH), 6.88–6.98 (m, 2H, ArH), 7.01–7.13 (m, 4H, ArH), 7.13–7.39 (m, 13H, ArH, =CH-6), 7.49 (t, *J* = 7.8 Hz, 2H, ArH), 7.55–7.67 (m, 3H, ArH). ^13^C{H} NMR (101 MHz, CDCl_3_) δ 34.01 (CH_2_-3), 35.49 (CH-4), 51.59 (4-CH), 60.14 (NCH), 118.45 (=C-5), 126.56, 127.43, 127.45 (2C), 127.64 (2C), 128.10 (2C), 128.15 (2C), 128.34 (2C), 128.46 (2C), 128.81 (2C), 128.90 (2C), 129.00 (2C), 129.31 (2C), 130.22 (2C), 133.05 (ArH), 137.67, 138.15, 139.15 (Ar), 139.56 (=CH-6), 141.37, 141.54 (Ar), 167.43 (C=O). GC-MS (EI 70 eV) *m*/*z*: 569 (<1), (M^+•^), 535 (12) [569 (M^+•^)-H_2_S], 207 (11), 168 (17), 167 (100), 165 (33), 152 (19). HRMS (ESI-TOF) *m*/*z*: [M + H]^+^ calcd. for C_37_H_31_NO_3_S, 570.2103; found, 570.2097.

*(4RS,5SR)-1,4-Dibenzhydryl-6-oxo-5-phenyl-1,4,5,6-tetrahydropyridine-3-sulfinic acid* (**6t**).

Yield of 20% (0.026 g, Method B, using 0.091 g of **1t**). The crude product, purified by column chromatography (SiO_2_, hexane/ethyl acetate, 2:1), yielded a white solid, m.p. 208–210 °C. ^1^H NMR (400 MHz, CDCl_3_) δ 2.41–2.44 (m, 1H, OH), 3.58 (dd, *J* = 4.2, 1.3 Hz, 1H, CH-4), 4.59 (dd, *J* = 3.7, 1.3 Hz, 1H, 4-CH), 4.66 (d, *J* = 4.1 Hz, 1H, CH-5), 6.54 (s, 1H, NCH), 6.69–6.76 (m, 2H, ArH), 6.92–7.00 (m, 2H, ArH), 7.00–7.06 (m, 2H, ArH), 7.10–7.40 (m, 15H, ArH, =CH-2), 7.46–7.54 (m, 2H, ArH), 7.58–7.64 (m, 1H, ArH), 7.69–7.75 (m, 2H, ArH). ^13^C{H} NMR (101 MHz, CDCl_3_) δ 43.55 (CH-4), 49.45 (CH-5), 60.31 (NCH), 69.60 (4-CH), 117.01 (=C-3), 126.62, 127.40 (2C), 127.48, 127.57 (2C), 127.99 (2C), 128.02, 128.34 (2C), 128.40, 128.51 (2C), 128.85 (2C), 128.92 (2C), 129.18 (2C), 129.28 (2C), 129.89 (2C), 133.10 (ArH), 137.18 (Ar), 137.59 (=CH-2), 137.90, 139.27, 140.46, 141.17 (Ar), 167.32 (C=O). GC-MS (EI 70 eV): decomposition. HRMS (ESI-TOF) *m*/*z*: [M + H]^+^ calcd. for C_37_H_31_NO_3_S, 570.2103; found, 570.2097.

*(4RS)-4-Benzhydryl-5-(benzofuran-5-yl)-1-phenyl-3,4-dihydropyridin-2(1H)-one* (**2u**).

Yield of 59% (0.15 g, Method B, using 0.160 g of **1u**). The crude product, purified by column chromatography (SiO_2_, hexane/ethyl acetate, 4:1), yielded a white solid, m.p. 148–149 °C. ^1^H NMR (400 MHz, CDCl_3_) δ 2.96 (dd, *J* = 16.3, 1.6 Hz, 1H, CHH-3), 3.09 (dd, *J* = 16.3, 7.3 Hz, 1H, CHH-3), 3.99 (ddd, *J* = 7.9, 7.3, 1.6 Hz, 1H, CH-4), 4.15 (d, *J* = 7.9 Hz, 1H, 4-CH), 6.48 (s, 1H, =CH-6), 6.67 (dd, *J* = 2.2, 1.0 Hz, 1H, ArH), 6.94–7.09 (m, 4H, ArH), 7.11–7.19 (m, 4H, ArH), 7.19–7.35 (m, 8H, ArH), 7.37–7.45 (m, 2H, ArH), 7.59 (d, *J* = 2.2 Hz, 1H, ArH). ^13^C{H} NMR (101 MHz, CDCl_3_) δ 35.93 (CH_2_-3), 39.36 (CH-4), 53.15 (4-CH), 106.54, 111.17, 118.66, 122.88 (ArH), 123.29 (=C-5), 126.08 (2C), 126.26, 126.98, 127.12 (ArH), 127.57 (Ar), 127.96 (3C, ArH, =CH-6), 128.47 (2C), 128.51 (2C), 129.08 (2C), 129.13 (2C), (ArH), 133.53, 140.36, 141.10, 142.07 (Ar), 145.46 (ArH), 153.96 (Ar), 167.55 (C=O). GC-MS (EI 70 eV) *m*/*z*: 455 (<1) [M^+•^], 287 (36) [M^+•^-168], 167 (100), 77 (28). HRMS (ESI-TOF) *m*/*z*: [M + H]^+^ calcd. for C_32_H_25_NO_2_, 456.1964; found, 456.1958.

*(4RS)-4-Benzhydryl-5-(benzo[b]thiophen-5-yl)-1-phenyl-3,4-dihydropyridin-2(1H)-one* (**2v**).

Yield of 70% (0.13 g, Method B, using 0.119 g of **1v**). The crude product, purified by column chromatography (SiO_2_, hexane/ethyl acetate, 4:1), yielded a solid, m.p. 90–92 °C. ^1^H NMR (400 MHz, CDCl_3_) δ 2.86 (dd, *J* = 16.0, 1.7 Hz, 1H, CHH-3), 3.16 (dd, *J* = 16.1, 6.8 Hz, 1H, CHH-3), 3.96 (ddd, *J* = 9.7, 6.8, 1.7 Hz, 1H, CH-4), 4.12 (d, *J* = 9.7 Hz, 1H, 4-CH), 6.46 (s, 1H, =CH-6), 6.76–6.88 (m, 4H, ArH), 6.98 (dd, *J* = 6.5, 3.1 Hz, 2H, ArH), 7.10 (t, *J* = 7.7 Hz, 1H, ArH), 7.16–7.34 (m, 9H, ArH), 7.37 (d, *J* = 5.6 Hz, 1H, ArH), 7.45 (t, *J* = 7.8 Hz, 2H, ArH), 7.66 (d, *J* = 8.0 Hz, 1H, ArH). ^13^C{H} NMR (101 MHz, CDCl_3_) δ 36.44 (CH_2_-3), 40.63 (CH-4), 54.41 (4-CH), 121.02, 122.64 (ArH), 123.33 (=C-5), 123.70, 123.97, 125.98 (3C), 126.15, 126.92, 127.21, 127.79 (2C), 128.12 (2C), 128.48 (2C), 128.70 (2C), 129.21 (2C), (ArH), 129.85 (=CH-6), 135.10, 137.27, 140.06, 140.19, 141.60, 141.78 (Ar), 167.75 (C=O). GC-MS *m*/*z*: 470 (<1), (M^+•^) (304 (6), (M^+•^-167), 303 (49), 275 (12), 171 (26), 168 (39), 167 (100), 165 (33), 152 (13), 77 (17). HRMS (ESI-TOF) *m*/*z*: [M + H]^+^ calcd. for C_32_H_26_NOS, 472.1735; found, 472.1729.

### 3.3. Synthesis of Compounds ***7***

A 25 mL Schlenk flask was charged with 16 mL of anhydrous THF and placed in an ice bath at 0 °C, 0.815 g of diphenylmethane (1.5 equiv., 4.858 mmol) was added, and subsequently, 2.04 mL of *n*-BuLi (2.5 M in hexanes, 1.575 equiv., 5.102 mmol) were carefully added dropwise from a syringe and stirred for 25 min at 0 °C. The orange-red solution was then transferred with a syringe to a second 50 mL flask placed in a −80 °C bath in which 2-pyridone **1d** (3.239 mmol) had been previously dissolved in 16 mL of anhydrous tetrahydrofuran. The reaction was carried out for 70 min at −80 °C, after which a 1.2-fold molar excess of the appropriate alkyl bromide was added and the reaction was continued for an additional 2 h. Then, saturated ammonium chloride solution (ca. 5 mL) was added, the solution was warmed to rt and extracted with ethyl acetate (3 × 80 mL), and the organic layer was dried over anhydrous magnesium sulfate, concentrated, and purified by column chromatography using silica gel as the stationary phase.

*(3SR,4RS)-4-Bnzhydryl-3-benzyl-1-phenyl-3,4-dihydropyridin-2(1H)-one* (**7a**).

Yield of 72% (1.08 g, using 0.6 g of **1d**). The crude product, purified by column chromatography (SiO_2_, hexane/ethyl acetate, 7:1), yielded a white solid, m.p. 109–110 °C. ^1^H NMR (400 MHz, CDCl_3_) δ 2.82 (dddd, *J* = 11.5, 3.4, 1.6, 1.1 Hz, 1H, CH-3), 2.89 (dd, *J* = 12.4, 11.6 Hz, 1H, 3-CHH), 2.96 (ddd, *J* = 11.4, 6.3, 1.1 Hz, 1H, CHH), 3.11 (dd, *J* = 12.5, 3.4 Hz, 1H, CH-4), 3.81 (d, *J* = 11.4 Hz, 1H, 4-CH), 4.97 (td, *J* = 7.8, 6.3, 1.6 Hz, 1H, =CH-5), 6.29 (d, *J* = 7.8 Hz, 1H, =CH-6), 6.80–6.89 (m, 2H, ArH), 7.05–7.17 (m, 8H, ArH), 7.18–7.38 (m, 8H, ArH), 7.38–7.47 (m, 2H, ArH). ^13^C{H} NMR (101 MHz, CDCl_3_) *δ* 36.27 (3-CH_2_), 38.30 (CH-4), 46.89 (CH-3), 55.13 (4-CH), 107.70 (=CH-5), 125.91 (2C), 126.41, 126.58, 126.73, 127.03, 127.96 (2C), 128.02 (2C), 128.46 (2C), 128.48 (2C), 128.61 (2C), 129.07 (2C), 129.50 (2C), (ArH), 129.55 (=CH-5), 138.02, 140.43, 141.49, 142.94 (Ar), 170.41 (C=O). GC-MS (EI 70 eV) *m*/*z*: 429 (<1) [M^+•^], 262 (64) [M^+•^-167 (benzhydryl radical)], 165 (10). HRMS (ESI-TOF) *m*/*z*: [M + H]^+^ calcd. for C_31_H_28_NO, 430.2171; found, 430.2166.

*(3SR,4RS)-4-Benzhydryl-3-(benzo[d][1,3]dioxol-5-ylmethyl)-1-phenyl-3,4-dihydropyridin-2(1H)-one* (**7b**).

Yield of 74% (0.61 g, using 0.3 g of **1d**). The crude product, purified by column chromatography (SiO_2_, hexane/ethyl acetate, 5:1), yielded a white solid, m.p. 218–220 °C. ^1^H NMR (400 MHz, CDCl_3_) δ 2.62–2.86 (m, 2H, CH-3, 3-CHH), 2.98 (dd, *J* = 11.3, 6.3 Hz, 1H, CH-4), 3.02 (dd, *J* = 11.3, 2.4 Hz, 1H, 3-CHH), 3.82 (d, *J* = 11.3 Hz, 1H, 4-CH), 4.96 (ddd, *J* = 7.8, 6.3, 1.4 Hz, 1H, =CH-5), 6.01 (dd, *J* = 3.8, 1.4 Hz, 2H, OCH_2_O), 6.28 (d, *J* = 7.8 Hz, 1H, =CH-6), 6.54–6.62 (m, 2H, ArH), 6.80 (d, *J* = 8.3 Hz, 1H, ArH), 6.90–6.99 (m, 2H, ArH), 7.06–7.33 (m, 11H, ArH), 7.37–7.48 (m, 2H, ArH). ^13^C{H} NMR (101 MHz, CDCl_3_) δ 36.09 (3-CH_2_), 38.40 (CH-4), 47.02 (CH-3), 55.17 (4-CH), 101.00 (OCH_2_O), 107.65 (=CH-5), 108.17, 109.77, 122.48, 125.91 (2C), 126.46, 126.68, 127.04, 128.01 (2C), 128.09 (2C), 128.49 (2C), 128.63 (2C), 129.08 (2C), (ArH), 129.56 (=CH-6), 131.76, 140.41, 141.54, 142.90, 146.33, 147.73 (Ar), 170.32 (C=O). GC-MS (EI 70 eV): decomposition. HRMS (ESI-TOF) *m*/*z*: [M + H]^+^ calcd. for C_32_H_28_NO_3_, 474.2069; found, 474.2064.

*(3SR,4RS)-4-benzhydryl-1-phenyl-3-(3,4,5-trimethoxybenzyl)-3,4-dihydropyridin-2(1H)-one* (**7c**).

Yield of 65% (0.59 g, using 0.3 g of **1d**). The crude product, purified by column chromatography (SiO_2_, hexane/ethyl acetate, 5:1), yielded a white solid, m.p. 91–93 °C. ^1^H NMR (400 MHz, CDCl_3_) δ 2.74–2.86 (m, 2H, 3-CHH, CH-3), 2.96 (dd, *J* = 11.5, 6.3 Hz, 1H, CH-4), 3.07 (d, *J* = 9.0 Hz, 1H, 3-CHH), 3.78–3.85 (m, 7H, 2 × OCH_3_, 4-CH), 3.91 (s, 3H, OCH_3_), 4.97 (td, *J* = 7.8, 6.3, 1.3 Hz, 1H, =CH-5), 6.29 (d, *J* = 7.8 Hz, 1H, =CH-6), 6.33 (s, 2H, ArH), 6.87–6.93 (m, 2H, ArH), 7.07–7.18 (m, 6H, ArH), 7.20–7.27 (m, 2H, ArH), 7.27–7.36 (m, 3H, ArH), 7.40–7.49 (m, 2H, ArH). ^13^C{H} NMR (101 MHz, CDCl_3_) δ 36.60 (3-CH_2_), 38.23 (CH-4), 47.02 (CH-3), 55.19 (4-CH), 56.18 (2C, 2 × OCH_3_), 60.99 (OCH_3_), 106.25 (2C, ArH), 107.68 (=CH-5), 125.94 (2C), 126.47, 126.62, 127.17, 127.94 (2C), 128.15 (2C), 128.52 (2C), 128.57 (2C), 129.15 (2C), (ArH), 129.59 (=CH-6), 133.75, 136.68, 140.37, 141.55, 142.84 (Ar), 153.21 (2C, Ar), 170.46. GC-MS (EI 70 eV): decomposition. HRMS (ESI-TOF) *m*/*z*: [M + H]^+^ calcd. for C_34_H_34_NO_4_, 520.2488; found, 520.2482.

### 3.4. Synthesis of Compounds ***8***, ***9***

Method A: In a round-bottomed flask fitted with a reflux condenser and an argon balloon, 3,4-dihydropyridin-2-one (0.295 mmol) and 6 mL (18.24 mmol) of 85% phosphoric(V) acid were placed, heated to 120 °C, and stirred for 2–3 h. After this time, the reaction mixture was cooled, the flask was placed in an ice bath, saturated sodium bicarbonate solution was carefully added and extracted with ethyl acetate (3 × 20 mL), the organic layer was dried over anhydrous magnesium sulfate and filtered, and the solvent was distilled off under reduced pressure. The crude product was purified by column chromatography (SiO_2_) using a mixture of hexane and ethyl acetate as an eluent.

Method B: The procedure was similar to method A, except that 0.295 mmol of 3,4-dihydropyridin-2-one was dissolved in 4 mL of anhydrous acetonitrile. Then, 0.21 mL of triflic acid (7.5-fold excess, 2.21 mmol) was slowly added dropwise, and the reaction was carried out for 24 h at room temperature. After this time, while cooling the flask, a saturated solution of NaHCO_3_ was slowly added. The rest of the procedure was the same as in Method A.

Method C: The procedure was similar to Method A, except that 0.587 mmol of 3,4-dihydropyridin-2-one was dissolved in 8 mL of anhydrous acetonitrile in the flask and a 2.5-fold excess of TIPSOTf (1.469 mmol) was added dropwise from a syringe. Then, the mixture was refluxed for 24 h (monitoring by ^1^H NMR). Then, the reaction mixture was cooled to room temperature, 10 mL of saturated NaHCO_3_ solution were added, and the aqueous layer was extracted with ethyl acetate (3 × 40 mL). The rest of the procedure was the same as in Method A.

*(1SR,5SR,6RS)-2,6-Diphenyl-1,4,5,6-tetrahydro-1,5-methanobenzo[c]azocin-3(2H)-one* (**8a**).

Yield of 43% (Method A), 64% (Method B), 27% (Method C). White solid, m.p. 183–185 °C. ^1^H NMR (400 MHz, CDCl_3_) δ 2.28 (dt, *J* = 13.3, 3.2 Hz, 1H, CHH_ax_-11), 2.39 (dtd, *J* = 13.3, 3.2, 1.4 Hz, 1H, CHH_eq_-11), 2.60–2.67 (m, 1H, CH-5), 2.74 (dt, *J* = 18.8, 1.4 Hz, 1H, CHH_eq_-4), 3.11 (dd, *J* = 18.8, 8.3 Hz, 1H, CHH_ax_-4), 4.26 (s, 1H, CH_α_-6), 4.74 (td, *J* = 3.2, 1.4 Hz, 1H, CH-1), 6.59 (dd, *J* = 7.7, 1.2 Hz, 1H, ArH), 6.90–6.96 (m, 2H, ArH), 7.01–7.06 (m, 4H, ArH), 7.16–7.33 (m, 5H, ArH), 7.37–7.44 (m, 2H, ArH). ^13^C{H} NMR (101 MHz, CDCl_3_) δ 25.57 (CH_2_-11), 35.06 (CH-5), 39.98 (CH_2_-4), 52.08 (CH-6), 59.24 (CH-1), 126.33, 126.41, 127.27, 128.02 (2C), 128.39 (2C), 128.43, 128.53 (2C), 128.58, 129.26 (2C), 132.05 (ArH), 134.87, 136.55, 142.09, 146.61 (Ar), 169.51 (C=O). GC-MS (EI 70 eV) *m*/*z*: 339 (100) [M^+•^], 204 (44), 178 (28), 141 (35), 135 (83). HRMS (ESI-TOF) *m*/*z*: [M + H]^+^ calcd. for C_24_H_22_NO, 340.1701; found, 340.1696.

*(1SR,5SR,6SR)-2,6-Diphenyl-1,4,5,6-tetrahydro-1,5-methanobenzo[c]azocin-3(2H)-one* (**9a**).

Yield of 22% (Method A), 20% (Method B), 12% (Method C). The crude product purified by column chromatography (SiO_2_, hexane/ethyl acetate, 2:1) gave a white solid, m.p. 218–220 °C. ^1^H NMR (400 MHz, CDCl_3_) δ 2.35–2.53 (m, 3H, CH_2_-4, CHH-11), 2.68–2.81 (m, 2H, CHH-11, CH-5), 4.63 (d, *J* = 6.1 Hz, 1H, CH_b_-6), 4.70–4.76 (m, 1H, CH-1), 6.49 (dd, *J* = 7.6, 1.4 Hz, 1H, ArH), 6.93–7.22 (m, 6H, ArH), 7.25–7.43 (m, 7H, ArH). ^13^C{H} NMR (101 MHz, CDCl_3_) δ 31.68 (CH_2_-11), 33.33 (CH-5), 33.83 (CH_2_-4), 50.67 (CH-6), 59.53 (CH-1), 125.85, 126.84, 127.22, 128.22 (4C), 128.47, 128.72 (2C), 129.23 (2C), 129.83 (br), 130.63 (ArH), 136.23, 137.08, 142.08, 143.16 (Ar), 169.48 (C=O). GC-MS (EI 70 eV) *m*/*z*: 339 (100) [M^+•^], 204 (45), 178 (27), 135 (93). HRMS (ESI-TOF) *m*/*z*: [M + H]^+^ calcd. for C_24_H_22_NO, 340.1701; found, 340.1696.

*(1SR,5SR,6RS)-2-Benzyl-6-phenyl-1,4,5,6-tetrahydro-1,5-methanobenzo[c]azocin-3(2H)-one* (**8b**).

Yield of 58% (Method B), 33% (Method C). The crude product, purified by column chromatography (SiO_2_, hexane/ethyl acetate, 3:1), yielded a white solid, m.p. 149–151 °C. ^1^H NMR (400 MHz, CDCl_3_) δ 1.90 (dt, *J* = 13.3, 3.4 Hz, 1H, CHH_ax_-11), 2.19 (dq, *J* = 13.0, 2.6 Hz, 1H, CHH_eq_-11), 2.46–2.59 (m, 1H, CH-5), 2.68 (d, *J* = 18.6 Hz, 1H, CHH_eq_-4), 3.06 (dd, *J* = 18.6, 8.0 Hz, 1H, CHH_ax_-4), 3.81 (d, *J* = 15.4 Hz, 1H, NCHH), 4.18 (s, 1H, CH_α_-6), 4.23 (td, *J* = 3.2, 1.3 Hz, 1H, CH-1), 5.56 (d, *J* = 15.4 Hz, 1H, NCHH), 6.83–6.92 (m, 2H, ArH), 6.96–7.04 (m, 1H, ArH), 7.14–7.27 (m, 6H, ArH), 7.30–7.43 (m, 5H, ArH). ^13^C{H} NMR (101 MHz, CDCl_3_) δ 25.60 (CH_2_-11), 35.09 (CH-5), 40.00 (CH_2_-4), 46.99 (NCH_2_), 52.00 (CH-6), 52.49 (CH-1), 126.36, 126.51, 127.46, 127.82, 128.15 (2C), 128.39 (4C), 128.50, 128.81 (2C), 132.22 (ArH), 135.82, 136.92, 137.19, 147.00 (Ar), 169.49 (C=O). GC-MS (EI 70 eV) *m*/*z*: 353 (78) [M^+•^], 206 (100), 148 (75), 106 (61), 91 (85). HRMS (ESI-TOF) *m*/*z*: [M + H]^+^ calcd. for C_25_H_24_NO, 354.1858; found, 354.1852.

*(1SR,5SR,6SR)-2-Benzyl-6-phenyl-1,4,5,6-tetrahydro-1,5-methanobenzo[c]azocin-3(2H)-one* (**9b**).

Yield of 15% (Method B), 18% (Method C). The crude product, purified by column chromatography (SiO_2_, *n*-hexane/ethyl acetate, 3:1), yielded a white solid, m.p. 150–152 °C. ^1^H NMR (400 MHz, CDCl_3_) δ 2.26–2.38 (m, 3H, CHH_eq_-4, CH_2_-11), 2.44 (dd, *J* = 19.0, 7.3 Hz, 1H, CHH_ax_-4), 2.59–2.76 (m, 1H, CH-5), 3.83 (d, *J* = 15.3 Hz, 1H, NCHH), 4.20 (td, *J* = 3.1, 1.3 Hz, 1H, CH-1), 4.57 (d, *J* = 6.3 Hz, 1H, CH_β_-6), 5.62 (d, *J* = 15.3 Hz, 1H, NCHH), 6.95–7.46 (m, 14H). ^13^C{H} NMR (101 MHz, CDCl_3_) δ 31.49 (CH_2_-11), 33.22 (CH-5), 33.51 (CH_2_-4), 46.77 (NCH_2_), 50.76 (CH-6), 52.80 (CH-1), 126.21, 126.84, 127.48, 128.01, 128.11 (2C), 128.28, 128.70 (2C), 128.84 (2C), 129.84 br (2C), 130.84 (ArH), 136.89, 137.22, 137.28, 143.14 (Ar), 169.86 (C=O). GC-MS (EI 70 eV) *m*/*z*: 353 (99) [M^+•·^], 204 (57), 148 (47), 128 (44), 106 (78), 91 (100). HRMS (ESI-TOF) *m*/*z*: [M + H]^+^ calcd. for C_25_H_24_NO, 354.1858; found, 354.1852.

*(1SR,5SR,6RS)-2-Methyl-6-phenyl-1,4,5,6-tetrahydro-1,5-methanobenzo[c]azocin-3(2H)-one* (**8c**).

Yield of 54% (Method B). The crude product, purified by column chromatography (SiO_2_, hexane/ethyl acetate, 2:1 then 1:1), yielded a white solid, m.p. 164–166 °C. ^1^H NMR (400 MHz, CDCl_3_) δ 2.06 (dt, *J* = 13.1, 3.3 Hz, 1H, CHH_ax_-11), 2.26 (dq, *J* = 13.1, 2.7 Hz, 1H, CHH_eq_-11), 2.48–2.55 (m, 1H, CH-5), 2.56 (d, *J* = 18.5 Hz, 1H, CHH_eq_-4), 2.92 (dd, *J* = 18.5, 7.9 Hz, 1H, CHH_ax_-4), 3.00 (s, 3H, NCH_3_), 4.14 (s, 1H, CH_α_-6), 4.25 (td, *J* = 3.3, 1.3 Hz, 1H, CH-1), 6.87–6.94 (m, 2H, ArH), 6.96–7.02 (m, 1H, ArH), 7.17–7.30 (m, 6H, ArH). ^13^C{H} NMR (101 MHz, CDCl_3_) δ 25.33 (CH_2_-11), 33.66 (NCH_3_), 35.37 (CH-5), 39.83 (CH_2_-4), 51.92 (CH-6), 57.27 (CH-1), 126.36, 126.51, 127.69, 128.41 (4C), 128.49, 132.25 (ArH), 135.48, 136.83, 147.05 (Ar), 169.61 (C=O). GC-MS (EI 70 eV) *m*/*z:* 277 (23) [M^+•^], 204 (48), 73 (100). HRMS (ESI-TOF) *m*/*z*: [M + H]^+^ calcd. for C_19_H_20_NO, 278.1545; found, 278.1539.

*(1SR,5SR,6SR)-2-Methyl-6-phenyl-1,4,5,6-tetrahydro-1,5-methanobenzo[c]azocin-3(2H)-one* (**9c**).

Yield of 11% (Method B). The crude product, purified by column chromatography (SiO_2_, hexane/ethyl acetate, 2:1 then 1:1), yielded a white solid, m.p. 192–193 °C. ^1^H NMR (400 MHz, CDCl_3_) δ 2.19 (dt, *J* = 18.5, 1.7 Hz, 1H, CHH_eq_-11), 2.30 (dd, *J* = 18.5, 7.3 Hz, 1H, CHH_ax_-11), 2.36 (dq, *J* = 13.0, 2.8 Hz, 1H, CHH_eq_-4), 2.50 (dt, *J* = 13.0, 3.5 Hz, 1H, CHH_ax_-4), 2.61–2.70 (m, 1H, CH-5), 3.03 (s, 3H, NCH_3_), 4.23 (td, *J* = 3.2, 1.3 Hz, 1H, CH-1), 4.57 (d, *J* = 6.4 Hz, 1H, CH_β_-6), 7.02–7.28 (m, 7H, ArH), 7.32 (t, *J* = 7.4 Hz, 2H, ArH). ^13^C{H} NMR (101 MHz, CDCl_3_) δ 31.26 (CH_2_-11), 33.46 (2C), (CH_3_, CH_2_-4), 33.50 (CH-5), 50.72 (CH-6), 57.55 (CH-1), 126.15, 126.77, 127.81, 128.22, 128.63 (2C), 129.80 br, (2C), 130.82 (ArH), 136.61, 137.25, 143.16 (Ar), 169.92 (C=O). GC-MS (EI 70 eV) *m*/*z*: 277 (23) [M^+•^], 204 (48), 73 (100). HRMS (ESI-TOF) *m*/*z*: [M + H]^+^ calcd. for C_19_H_20_NO, 278.1545; found, 278.1539.

*(1SR,5SR,6RS,11SR)-11-Methyl-2,6-diphenyl-1,4,5,6-tetrahydro-1,5-methanobenzo[c]azocin-3(2H)-one* (**8d**).

Yield of 63% (Method C). The crude product, purified by column chromatography (SiO_2_, hexane/ethyl acetate, 3:1), yielded a white solid, m.p. 170–172 °C. ^1^H NMR (400 MHz, CDCl_3_) δ 1.33 (d, *J* = 6.9 Hz, 3H, 11-CH_3_), 2.37 (ddd, *J* = 8.6, 2.7, 1.7 Hz, 1H, CH-5), 2.53 (qt, *J* = 6.9, 2.7 Hz, 1H, CH-11), 2.60 (d, *J* = 19.1 Hz, 1H, CHH_eq_-4), 3.08 (dd, *J* = 19.1, 8.6 Hz, 1H, CHH_ax_-4), 4.32 (s, 1H, CH_a_-6), 4.46 (dd, *J* = 2.7, 1.7 Hz, 1H, CH-1), 6.63 (dd, *J* = 7.6, 1.4 Hz, 1H, ArH), 6.88–6.95 (m, 2H, ArH), 7.02–7.13 (m, 4H, ArH), 7.17–7.35 (m, 5H), 7.36–7.47 (m, 2H). ^13^C{H} NMR (101 MHz, CDCl_3_) δ 17.06 (11-CH_3_), 27.28 (CH-11), 36.65 (CH_2_-4), 41.02 (CH-5), 53.92 (CH_a_-6), 64.89 (CH-1), 126.40 (2C), 127.13, 128.06 (2C), 128.31 (2C), 128.42, 128.56, 128.70 (2C), 129.25 (2C), 132.09 (ArH), 134.31, 137.85, 142.31, 146.44 (Ar), 169.07 (C=O). GC-MS (EI 70 eV) *m*/*z*: 353 (86) [M^+•^], 338 (39) [M^+•^-Me], 218 (100), 203 (46), 178 (33), 135 (77), 92 (35), 77 (23). HRMS (ESI-TOF) *m*/*z*: [M + H]^+^ calcd. for C_25_H_24_NO, 354.1858; found, 354.1852.

*(1SR,5SR,6SR,11SR)-11-Methyl-2,6-diphenyl-1,4,5,6-tetrahydro-1,5-methanobenzo[c]azocin-3(2H)-one* (**9d**).

Yield of 20% (Method C). The crude product, purified by column chromatography (SiO_2_, hexane/ethyl acetate, 3:1), yielded a white solid, m.p. 220–222 °C. ^1^H NMR (400 MHz, CDCl_3_) δ 1.52 (d, *J* = 6.9 Hz, 3H, 11-CH_3_), 2.35–2.41 (m, 2H, CH_2_-4), 2.43–2.51 (m, 1H, CH-5), 2.60 (qt, *J* = 6.9, 2.4 Hz, 1H, CH-11), 4.45 (t, *J* = 2.2 Hz, 1H, CH-1), 4.62 (d, *J* = 5.6 Hz, 1H, CH_b_-6), 6.55 (dd, *J* = 7.6, 1.4 Hz, 1H, ArH), 7.00–7.23 (m, 6H), 7.24–7.46 (m, 7H). ^13^C{H} NMR (101 MHz, CDCl_3_) δ 17.37 (11-Me), 29.45 (CH_2_-4), 34.45 (CH-11), 39.44 (CH-5), 52.47 (CH-6), 65.26 (CH-1), 126.00, 126.85, 127.15, 128.17, 128.21 (3C), 128.52, 128.66 (2C), 129.25 (2C), 130.02, 130.61 (ArH), 135.62, 138.12, 142.32, 142.86 (Ar), 169.21 (C=O). GC-MS (EI 70 eV) *m*/*z*: 353 (100) [M^+•^], 218 (38), 178 (23), 133 (32), 91 (24); HRMS (ESI-TOF) *m*/*z*: [M + H]^+^ calcd. for C_25_H_24_NO, 354.1858; found, 354.1852.

*(1SR,5SR,6RS,11SR)-2-Benzyl-11-methyl-6-phenyl-1,4,5,6-tetrahydro-1,5-methano-benzo[c]azocin-3(2H)-one* (**8e**).

Yield of 34% (Method C). The crude product, purified by column chromatography (SiO_2_, hexane/ethyl acetate, 3:1), yielded a colorless oil. ^1^H NMR (400 MHz, CDCl_3_) *δ* 0.85 (d, *J* = 6.9 Hz, 3H, 11-CH_3_), 2.22 (dd, *J* = 8.2, 2.3 Hz, 1H, CH-5), 2.27–2.35 (m, 1H, CH-11), 2.54 (d, *J* = 18.9 Hz, 1H, CHH_eq_-4), 3.03 (dd, *J* = 18.9, 8.2 Hz, 1H, CHH_ax_-4), 3.75 (d, *J* = 14.8 Hz, 1H, NCHH), 3.96 (t, *J* = 2.3 Hz, 1H, CH-1), 4.24 (s, 1H, CH_α_-6), 5.58 (d, *J* = 14.9 Hz, 1H, NCHH), 6.80–6.90 (m, 2H, ArH), 6.98–7.04 (m, 1H, ArH), 7.11–7.26 (m, 6H, ArH), 7.28–7.41 (m, 5H, ArH). ^13^C{H} NMR (101 MHz, CDCl_3_) δ 16.42 (11-CH_3_), 27.24 (CH-11), 36.47 (CH_2_-4), 40.84 (CH-5), 46.98 (NCH_2_), 53.84 (CH-6), 57.61 (CH-1), 126.30, 126.46, 127.58, 127.76, 128.28 (2C), 128.43, 128.52 (2C), 128.61 (2C), 129.16 (2C), 132.28, (ArH), 135.26, 136.91, 138.19, 146.91 (Ar), 169.12 (C=O). GC-MS *m*/*z*: 367 (82) [M^+•^], 220 (100), 205 (78), 146 (23), 25 (78), 146 (23), 106 (25), 91 (55). HRMS (ESI-TOF) *m*/*z*: [M + H]^+^ calcd. for C_26_H_26_NO, 368.2014; found, 368.2009.

*(1SR,5SR,6SR,11SR)-2-Benzyl-11-methyl-6-phenyl-1,4,5,6-tetrahydro-1,5-methanobenzo[c]azocin-3(2H)-one* (**9e**).

Yield of 17% (Method C). The crude product, purified by column chromatography (SiO_2_, hexane/ethyl acetate, 3:1), yielded a white solid, m.p. = 220–221 °C. ^1^H NMR (400 MHz, CDCl_3_) δ 1.07 (d, *J* = 6.8 Hz, 3H, 11-CH_3_), 2.25 (d, *J* = 17.6 Hz, 1H, CHH-4), 2.31–2.47 (m, 3H, CHH-4, CH-5, CH-11), 3.77 (d, *J* = 14.8 Hz, 1H, NCHH), 3.93 (t, *J* = 2.1 Hz, 1H, CH-1), 4.56 (d, *J* = 5.6 Hz, 1H, CH-6_b_), 5.65 (d, *J* = 14.9 Hz, 1H, NCHH), 7.05–7.45 (m, 14H, ArH). ^13^C{H} NMR (101 MHz, CDCl_3_) *δ* 16.86 (11-CH_3_), 29.25 (CH_2_-4), 34.21 (CH-11), 39.16 (CH-5), 46.72 (NCH_2_), 52.51 (CH-6), 57.88 (CH-1), 126.18, 126.80, 127.57, 127.98, 128.15, 128.65 (4C), 129.05 (2C), 130.01 (2C, br), 130.81 (ArH), 136.40, 137.05, 138.41, 142.95 (Ar), 169.41 (C=O). ^1^H NMR (400 MHz, Toluene-d_8_) δ 0.74 (d, *J* = 6.9 Hz, 3H, 11-CH_3_), 1.74–1.83 (m, 1H, CH-11), 1.83–1.90 (m, 1H, CH-5), 2.18 (dd, *J* = 19.0, 7.3 Hz, 1H, CHH_ax_-4), 2.28 (d, *J* = 19.0 Hz, 1H, CHH_eq_-4), 3.58 (d, *J* = 14.8 Hz, 1H, NCHH), 3.65 (t, *J* = 2.2 Hz, 1H, CH-1), 4.14 (d, *J* = 6.2 Hz, 1H, CH-6_b_), 5.83 (d, *J* = 14.8 Hz, 1H, NCHH), 6.87–7.32 (m, 14H, ArH). GC-MS (EI 70 eV): *m*/*z*= 367 (100) [M^+•^], 220 (37), 205 (34), 179 (19), 143 (21), 106 (43), 91 (71). HRMS (ESI-TOF) *m*/*z*: [M + H]^+^ calcd. for C_26_H_26_NO, 368.2014; found, 368.2009.

*(1SR,5SR,6RS,11SR)-11-Benzylo-6-fenylo-1,4,5,6-tetrahydro-1,5-metanobenzo[c]azocyn-3(2H)-on* (**8f**).

Yield of 57% (Method B). The crude product, purified by column chromatography (SiO_2_, hexane/ethyl acetate, 2:1), yielded a white semi-solid. ^1^H NMR (400 MHz, CDCl_3_) δ 2.36 (d, *J* = 8.2 Hz, 1H, CH-5), 2.47 (d, *J* = 19.1 Hz, 1H, CHH-4), 2.56–2.62 (m, 1H, CH-11), 2.66 (dd, *J* = 13.7, 6.3 Hz, 1H, 11-CHH), 2.88 (dd, *J* = 13.7, 9.6 Hz, 1H, 11-CHH), 2.95 (dd, *J* = 19.1, 8.3 Hz, 1H, CHH-4), 4.02 (dt, *J* = 4.6, 2.1 Hz, 1H, CH-1), 4.24 (s, 1H, CH-6), 6.45 (d, *J* = 4.6 Hz, 1H, NH), 6.83–6.92 (m, 2H, ArH), 6.96–7.05 (m, 3H, ArH), 7.05–7.10 (m, 1H, ArH), 7.17–7.30 (m, 8H, ArH). ^13^C{H} NMR (101 MHz, CDCl_3_) *δ* 33.56 (CH-11), 36.07 (CH_2_-4), 36.51 (11-CH_2_), 39.18 (CH-5), 52.66 (CH-6), 53.84 (CH-1), 126.42, 126.45, 127.35, 127.99, 128.34 (2C), 128.44, 128.56 (2C), 128.59 (2C), 128.73 (2C), 132.10 (ArH), 134.34, 138.91, 140.14, 146.35 (Ar), 172.13 (C=O). GC-MS (EI 70 eV) *m*/*z*: 353 (2) [M^+•^-benzhydryl radical), 294 (100), 217 (31), 91 (37). HRMS (ESI-TOF) *m*/*z*: [M + H]^+^ calcd. for C_25_H_24_NO, 354.1858; found, 354.1852.

*(1SR,5SR,6SR,11SR)-11-Benzylo-6-fenylo-1,4,5,6-tetrahydro-1,5-metanobenzo[c]azocyn-3(2H)-on* (**9f**).

Yield of 10% (Method B). The crude product, purified by column chromatography (SiO_2_, hexane/ethyl acetate, 2:1), yielded a white semi-solid. ^1^H NMR (400 MHz, CDCl_3_) δ 2.18 (d, *J* = 19.2 Hz, 1H, CHH), 2.34 (dd, *J* = 19.2, 7.5 Hz, 1H, CHH-4), 2.45 (dd, *J* = 9.1, 6.8 Hz, 1H, CH-11), 2.57–2.66 (m, 1H, CH-5), 2.92 (dd, *J* = 13.7, 6.8 Hz, 1H, 11-CHH), 3.02 (dd, *J* = 13.7, 9.1 Hz, 1H. 11-CHH), 4.08 (dt, *J* = 4.3, 2.0 Hz, 1H, CH-1), 4.57 (d, *J* = 6.1 Hz, 1H, CH-6), 6.33 (d, *J* = 4.3 Hz, 1H, NH), 6.97–7.08 (m, 3H, ArH), 7.07–7.18 (m, 3H, ArH), 7.22–7.30 (m, 5H, ArH), 7.31–7.38 (m, 3H, ArH). ^13^C{H} NMR (101 MHz, CDCl_3_) δ 29.08 (CH_2_-4), 37.18 (CH-11), 37.28 (11-CH_2_), 40.88 (CH-5), 52.40 (CH-6), 53.59 (CH-1), 126.63, 126.91 (2C), 127.67, 128.16, 128.66 (2C), 128.78 (2C), 128.98 (2C), 129.99 br (2C), 130.75 (ArH), 135.62, 139.06, 140.42, 142.75 (Ar), 172.35 (C=O). GC-MS (EI 70 eV) *m*/*z*: 353 (4) [M^+•^- benzhydryl radical], 294 (100), 217 (20), 91 (42). HRMS (ESI-TOF) *m*/*z*: [M + H]^+^ calcd. for C_25_H_24_NO, 354.1858; found, 354.1852.

*(1SR,4RS,5RS,6RS)-4-Benzyl-2,6-diphenyl-1,4,5,6-tetrahydro-1,5-methanobenzo[c]azocin-3(2H)-one* (**14a**). Major isomer.

Total yield of **14a** and **15a** (80:20) of 66% (Method B). The crude product, purified by column chromatography (SiO_2_, hexane/ethyl acetate, 5:1), yielded a white semi, m.p. = 187–190 °C. Spectral data for **14a** refined from spectra of mixture **14a**:**15a** (80:20). ^1^H NMR (400 MHz, CDCl_3_) δ 2.04 (dt, *J* = 13.4, 3.6 Hz, 1H, CHH-11), 2.15 (dt, *J* = 13.4, 3.0 Hz, 1H, CHH-11), 2.36–2.43 (m, 1H, CH-5), 2.79–2.92 (m, 2H, CH-4, 4-CHH), 3.59 (d, *J* = 10.9 Hz, 1H, 4-CHH), 3.90 (s, 1H, CH-6), 4.70 (q, *J* = 2.6 Hz, 1H, CH-1), 6.43 (dd, *J* = 6.6, 2.9 Hz, 2H, ArH), 6.64 (dd, *J* = 7.7, 1.4 Hz, 1H, ArH), 6.96 (d, *J* = 7.5 Hz, 1H, ArH), 7.03–7.45 (m, 15H, ArH). ^13^C{H} NMR (101 MHz, CDCl_3_) δ 23.70 (CH_2_-11), 38.66 (CH-5), 39.44 (4-CH_2_), 50.60 (CH-4), 52.58 (CH-6), 59.25 (CH-1), 126.15, 126.48, 126.51, 127.16, 127.92 (2C), 128.11 (2C), 128.49 (2C), 128.53, 128.61 (3C), 129.26 (2C), 129.52 (2C), 132.06 (ArH), 134.56, 136.66, 140.04, 142.57, 146.03 (Ar), 171.74 (C=O). HRMS (ESI-TOF) *m*/*z*: [M + H]^+^ calcd. for C_31_H_28_NO, 430.2171; found, 430.2166.

*(1SR,4RS,5RS,6SR)-4-Benzyl-2,6-diphenyl-1,4,5,6-tetrahydro-1,5-methanobenzo[c]azocin-3(2H)-one* (**15a**). Minor isomer.

Spectral data for **15a** refined from spectra of mixture **14a**:**15a** (80:20). ^1^H NMR (400 MHz, CDCl3) δ 2.26 (dt, *J* = 13.1, 3.0 Hz, 1H, CHH-11), 2.43–2.50 (m, 1H, CH-5), 2.56 (dt, *J* = 13.1, 3.6 Hz, 1H, CHH-11), 2.72 (dd, *J* = 10.3, 4.0 Hz, 1H), 2.79–2.92 (m, 1H, CH-4,), 3.05 (dd, *J* = 13.7, 4.0 Hz, 1H), 4.49 (d, *J* = 6.3 Hz, 1H, CH-6), 4.66 (t, *J* = 3.3 Hz, 1H, CH-1), 6.45–6.50 (m, 1H, ArH), 6.75–6.85 (m, 1H, ArH), 6.98–7.02 (m, 1H, ArH), 7.03–7.45 (m, 16H). ^13^C{H} NMR (101 MHz, CDCl_3_) δ 28.65, 35.53, 39.68, 42.55, 50.70, 59.62, 125.82, 126.03, 126.62, 128.17, 128.26 (3C), 128.53 (4C), 128.61 (2C), 128.99 (2C), 129.19 (2C), 129.26, 130.45, 136.00, 137.16, 139.25, 142.24, 142.43, 172.11.

*(5RS,7RS,8RS)-7-Benzhydryl-12-phenyl-6,7,8,9-tetrahydro-5H-5,8-(epiminomethano)cyclohepta [4,5]benzo [1,2-d][1,3]dioxol-11-one* (**16a**).

Yield of 70% (Method B). The crude product, purified by column chromatography (SiO_2_, hexane/ethyl acetate, 4:1), yielded a white semi, 151–153 °C. ^1^H NMR (400 MHz, CDCl_3_) δ 1.95 (ddd, *J* = 14.0, 8.3, 4.6 Hz, 1H, CHH_β_-6), 2.25 (ddd, *J* = 14.0, 9.1, 2.5 Hz, 1H, CHH_α_-6), 2.92 (dd, *J* = 5.1, 2.6 Hz, 1H, CH-8), 2.97 (dd, *J* = 17.4, 5.1 Hz, 1H, CHH_β_-9), 3.12 (ddd, *J* = 12.0, 9.1, 8.3 Hz, 1H, CH-7), 3.31 (dd, *J* = 17.4, 2.6 Hz, 1H, CHH_α_-9), 3.87 (d, *J* = 12.0 Hz, 1H, 7-CH), 4.43 (dd, *J* = 4.6, 2.5 Hz, 1H, CH_b_-5), 5.94 (d, *J* = 1.1 Hz, 1H, OCHHO), 5.94 (d, *J* = 1.1 Hz, 1H, OCHHO), 6.51 (d, *J* = 1.0 Hz, 1H, CH-4), 6.66 (d, *J* = 1.1 Hz, 1H, CH-1), 7.02–7.46 (m, 15H, ArH). ^13^C{H} NMR (101 MHz, CDCl_3_) *δ* 37.40 (CH_2_-9), 39.48 (CH_2_-6), 39.52 (CH-7), 44.42 (CH-8), 59.14 (7-CH), 64.19 (CH-5), 101.18 (OCH_2_O), 107.98 (CH-4), 110.86 (CH-1), 124.82 (2C), 126.30, 126.55, 126.71, 127.88 (2C), 128.07 (2C), 128.65 (2C), (ArH), 128.89 (Ar), 128.95 (4C), 131.58, 142.19, 142.94, 143.45, 145.78, 147.57 (Ar), 172.29 (C=O). GC-MS (EI 70 eV): decomposition. HRMS (ESI-TOF) *m*/*z*: [M + H]^+^ calcd. for C_32_H_28_NO_3_, 474.2069; found, 474.2064.

*(5RS,7RS,8RS)-7-Benzhydryl-2,3,4-trimethoxy-11-phenyl-6,7,8,9-tetrahydro-5H-5,8-(epiminomethano)benzo [7]annulen-10-one* (**16b**).

Yield of 87% (Method B). The crude product, purified by column chromatography (SiO_2_, hexane/ethyl acetate, 4:1), yielded a white semi, 205–207 °C. ^1^H NMR (400 MHz, CDCl_3_) δ 2.00 (ddd, *J* = 14.0, 8.3, 4.8 Hz, 1H, CHH_β_-6), 2.16 (ddd, *J* = 14.0, 9.1, 2.6 Hz, 1H, CHH_α_-6), 2.92 (dd, *J* = 4.8, 3.0 Hz, 1H, CH-8), 3.03 (dd, *J* = 17.9, 4.9 Hz, 1H, CHH_β_-9), 3.13 (ddd, *J* = 12.0, 9.1, 8.3 Hz, 1H, CH-7), 3.36 (ddd, *J* = 17.9, 3.0, 0.8 Hz, 1H, CHH_α_-9), 3.61 (s, 3H, OCH_3_), 3.85 (s, 3H, OCH_3_), 3.85 (s, 3H, OCH_3_), 3.88 (d, *J* = 12.0 Hz, 1H, 7-CH), 5.29 (dd, *J* = 4.7, 2.6 Hz, 1H, CH-5), 6.49 (s, 1H, CH-1), 7.09–7.46 (m, 15H, ArH). ^13^C{H} NMR (101 MHz, CDCl_3_) δ 37.54 (CH_2_-9), 39.20 (CH_2_-6), 39.86 (CH-7), 44.51 (CH-8), 54.66 (CH-5), 55.89 (OCH_3_), 59.18 (7-CH), 60.85 (OCH_3_), 61.44 (OCH_3_), 109.41 (CH-1), 124.62 (Ar), 124.82 (2C), 126.17, 126.50, 126.65, 127.89 (2C), 128.07 (2C), 128.63 (2C), 128.86 (2C), 128.91 (2C), (ArH), 131.44, 139.97, 142.11, 143.00, 143.57, 149.78, 152.83 (Ar), 172.72 (C=O). GC-MS (EI 70 eV): decomposition. HRMS (ESI-TOF) *m*/*z*: [M + H]^+^ calcd. for C_34_H_34_NO_4_, 520.2488; found, 520.2482.

## 4. Conclusions

To sum up, we have demonstrated that the 1,4 addition of diphenylmethyllithium (**BzhLi**) or benzhydrylmagnesiate reagents to *N*1/C5-functionalized 2-pyridones provided reliable access to racemic, benzhydryl-functionalized 3,4-dihydropyridin-2-ones with good yields and high regioselectivity. Both methods can be used complementarily based on specific differences. The influence of substituents on regioselectivity has been noted, allowing for control over the regioselectivity of the addition. The treatment of δ-enelactams with TfOH and/or TIPSOTf results in the partially stereoselective synthesis of 6-phenyl-functionalized 7,8-benzomorphanones, indicating its potential in pharmaceutical research. The described cyclization protocol can be extended for the synthesis of novel C3–C6-bridged δ-lactams by using a methoxy-substituted benzyl group at C3 in the substrate.

## Figures and Tables

**Table 1:** Addition of benzhydrylmagnesiates **A**–**D** (Scheme 3) to 2-pyridone **1e**.

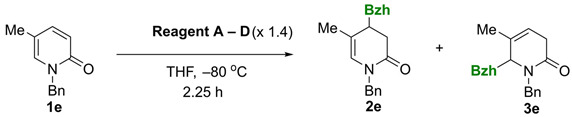						
	Reagent	Conversion ^a,b^ of 1e [%]	2e Yield ^c^ [%]	3e Yield ^c^ [%]	2e, 3e Total Yield ^c^ [%]	2e:3e
1	**A-1**	99	39	19	58	67:33 ^c^
2	**A-2**	88	-	-	-	63:37 ^b^
3	**B-1**	95	59	22	81	73:27 ^c^
4	**B-1**	99	57	24	81 ^d^	70:30 ^c^
5	**B-1**	**99**	**67**	**22**	**89** ^e^	**75:25** ^c^
6	**B-2**	28	-	-	-	67:33 ^b^
7	**C-1**	99	51	31	82	62:38 ^c^
8	**D-1**	99	45	31	76	59:41 ^c^

^a^ A concentration of 0.08 mol/dm^3^ was applied unless otherwise specified; ^b^ assigned by ^1^H NMR; ^c^ yields of isolated products; ^d^ a concentration of 0.06 mol/dm^3^ was applied; ^e^ a concentration of 0.034 mol/dm^3^ was applied.

## Data Availability

Data are contained within the article and the Supplementary Materials.

## Supplementary Materials

The following supporting information can be downloaded at: https://www.mdpi.com/article/10.3390/molecules29225274/s1, Spectroscopic data and procedures for the synthesis of compounds **1c** [33], **1e**, and **1g** (Scheme S1: Synthesis of compounds **1c**, **1e**, and **1g**); compounds **1d** [34], **1f** [35], **1h** [34], **1u**, and **1v** (Scheme S2: Synthesis of **1d**, **1f**, **1h**, **1u**, and **1v**); compounds **1i**–**1k** [36], **1l** [37], **1m** [17], **1n** [38], and **1o**–**1t** [36] (Scheme S3: Synthesis of **1i**–**1t**); 5-benzyl-2-methoxypyridine [39] and 2-methoxy-5-(phenylsulfonyl)pyridine [40]; compounds **4a** and **5a** (Scheme S4: Synthesis of **4a** and **5a**); and compounds **12** and **13**. Structural analysis of bridged δ-lactams **8**, **9**, and **16**. Figure S1: *J*^3^_H,H_ refined from ^1^H NMR spectra and calculated based on the Haasnoot equation [28] (in round brackets) using structures optimized by the PM3 method [29] for all possible isomers **8**–**11**, together with the Overhouser effects derived from ^1^H,^1^H NOESY spectra for **8** and **9**; Figure S2: ^1^H,^1^H NOESY spectra of compound **8b** (CDCl_3_); Figure S3: ^1^H,^1^H NOESY spectra of compound **9b** (CDCl_3_); Figure S4: ^1^H,^1^H NOESY spectra of compound **9b** (Toluene-d_8_); Figure S5: *J*^3^_H,H_ refined from ^1^H NMR spectra and calculated based on the Haasnoot equation [30] (in round brackets) using structures optimized by the PM3 method for compounds **16a** and **16b**; Figure S6. ^1^H,^1^H NOESY spectra of compound **16a** (CDCl_3_); Figure S7. ^1^H,^1^H NOESY spectra of compound **16b** (CDCl_3_). Figure S8. ^13^C,^1^H HETCOR spectra of compound **6s** (CDCl_3_); Figure S9. ^1^H,^1^H NOESY spectra of compound **6s** (CDCl_3_); Figure S10. ^1^H,^1^H DQFCOSY spectra of compound **6t** (CDCl_3_); Figure S11. ^13^C,^1^H HETCOR spectra of compound **6t** (CDCl_3_); Figure S12. ^1^H,^1^H NOESY spectra of compound **6t** (CDCl_3_); Copies of the ^1^H NMR and ^13^C NMR spectra for all new compounds **1**–**16**.
