# Supplementary material for: Direct Synthesis of Benzhydryl-Functionalized 3,4-Dihydropyridin-2-ones from 2-Pyridones and Their Use in the Formation of Bridged δ-Lactams
Source: Molecules. 2024 Nov 7;29(22):5274. doi: 10.3390/molecules29225274 (PMC11597090; doi:10.3390/molecules29225274)
Supplement: Supplementary file 1 [file molecules-29-05274-s001.zip › molecules-3271608-supplementary.pdf]

Direct Synthesis of Benzhydryl-Functionalized 3,4-Dihydropyridin-2-ones from 2-Pyridones and Their Use in the Formation of Bridged δ-Lactams

Zofia M. Myk[1], Jacek G. Sośnicki[1*], Łukasz Struk[1,2]

[1] Department of Organic and Physical Chemistry, Faculty of Chemical Technology and Engineering, West Pomeranian University of Technology, Szczecin, Al. Piastów 42, 71-065 Szczecin, Poland

[2] Center for Advanced Materials and Manufacturing Process Engineering (CAMMPE), Al. Piastów 42, 71-065 Szczecin, Poland

Table of Contents

1. Synthesis of Substrates **1**.. S2
2. Synthesis of Compounds **4a** and **5a**.. S6
3. Experimental and Spectral Data for Compounds **12** and **13**.................................. S7
4. Structural Analysis of Bridged δ-Lactams **8**, **9**, and **16**................................... S8
5. 2D Spectra for Compounds **6s** and **6t**.. S12
6. ^{1}H and ^{13}C Spectra for Compounds **1–16**..................................... S15

1. Synthesis of Substrate **1**

1.1. Synthesis of Substrates **1c**, **1e** and **1g**

Substrates **1c**, **1e**, and **1g** were obtained according to the procedure described earlier, with some modifications [16].

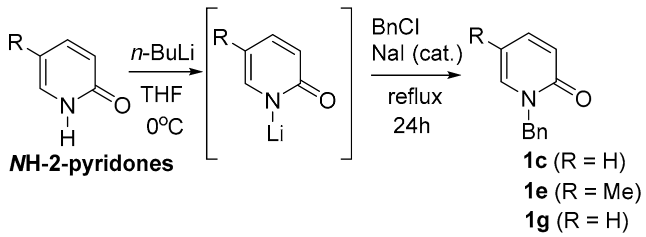

Scheme S1. Synthesis of compounds **1c**, **1e**, and **1g**

Representative procedure for the synthesis of 1-benzylpyridin-2(1*H*)-one (**1c**):

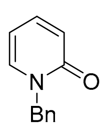
1c

To a cooled (0° C) and stirred solution of *N*H pyridin-2(1*H*)-one (2 g, 21.3 mmol) in anhydrous THF (97 mL) in a 250 mL Schlenk flask equipped with septum and argon balloon, *n*-BuLi (2.1 mL, 11.0 M in hexanes, 23.1 mmol, 1.08 equiv.) was added via syringe over a few minutes, and the mixture was stirred for 5 min. To a cooled (0° C) solution, 3.15 mL of benzyl chloride (1.3 equiv.) and 0.63 g of NaI (4.2 mmol, 0.2 equiv.) were added. The resulting solution was then stirred under gentle reflux for 24 h under argon (a condenser was capped with an argon balloon). After this time, the mixture was cooled to rt, and aqueous saturated NH_4Cl (30 mL) was added. The aqueous layer was extracted with ethyl acetate (3 x 80 mL), and the combined organic layers were dried with $MgSO_4$. The mixture was filtered, and the solvents were evaporated under reduced pressure. The crude product, purified by column chromatography (SiO_2, ethyl acetate, 1:1), yielded 3.65g of **1c** as a white solid (90% yield). (Spectral data for this product matched those reported earlier [33].)

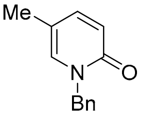
1e

1-Benzyl-5-methylpyridin-2(1*H*)-one (**1e**).

Yield of 93% (3.4 g). 5-Methylpyridin-2(1*H*)-one (2 g, 18.3 mmol), *n*-BuLi (2.5 M in hexanes, 7.7 mL, 19.3 mmol, 1.05 equiv.), BnCl (2.74 mL), and 0.55 g of NaI were used. The crude product, purified by column chromatography (SiO_2, ethyl acetate), yielded 3.4 g of **1e** as a white product. (Spectral data for this product matched those reported earlier [15].)

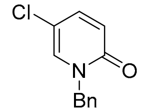
1g

1-Benzyl-5-chloropyridin-2(1*H*)-one (**1g**).

Yield of 85% (1.44 g). 5-Chloropyridin-2(1*H*)-one (1 g, 7.72 mmol), *n*-BuLi (0.74 mL, 11.0 M in hexanes, 8.1 mmol, 1.05 equiv.), BnCl (1.15 mL), and NaI (0.23 g) were used. The crude product, purified by column chromatography (SiO_2, *n*-hexane : ethyl acetate, 3:2), yielded 1.44 g of **1g** as a colorless solid. (Spectral data for this product matched those reported earlier [14].)

1.2. Synthesis of Substrates **1d**, **1f**, **1h**, **1u**, and **1v**

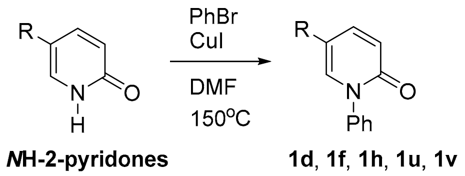

Scheme S2. Synthesis of **1d**, **1f**, **1h**, **1u** and **1v**

General procedure:
In a 250 mL round-bottom flask equipped with a magnetic dipole and a reflux condenser with a tube filled with calcium chloride at the end, the appropriate 2-pyridone (52.58 mmol), bromobenzene (2 equiv., 105.16 mmol, 11.1 mL) was placed, with anhydrous DMF (83 mL) and anhydrous potassium carbonate (1.1 equiv., 57.83 mmol, 7.99 g). The content of the flask was stirred for 5 minutes at room temperature, after which copper iodide (0.1 equiv., 5.23 mmol, 1g for **1d**, **1u**, **1v**; or 0.25 equiv., 13.07 mmol, 2.5 g for **1f**, **1h**) was added. The reaction was then conducted at 150 °C for 22 hours (**1d**, **1f**, **1h**) or 18 hours (**1u**, **1v**). After this, the reaction mixture was cooled, and aqueous ammonia (25%, 60 mL) was added and extracted with ethyl acetate (4 x 80 mL). The organic layer was dried by shaking with anhydrous magnesium sulfate and filtered through a Celite® pad on a glass funnel, and DMF was distilled off under reduced pressure (≈5 mbar, 70 °C). The crude product was purified by crystallization from AcOEt and hexane (the filtered crystals were washed with a mixture of AcOEt and hexane) or by column liquid chromatography (details below).

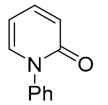

1d

1-Phenylpyridin-2(1*H*)-one (**1d**).
Yield of 80%. The crude product was purified by crystallization from AcOEt and *n*-hexane. (Spectral data for this product matched those reported earlier [34].)

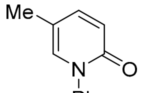

1f

5-Methyl-1-phenylpyridin-2(1*H*)-one (**1f**).
Yield of 62%. The crude product was purified by crystallization from AcOEt and *n*-hexane. (Spectral data for this product matched those reported earlier [35].)

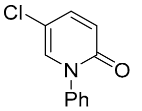

1h

5-Chloro-1-phenylpyridin-2(1*H*)-one (**1h**).
Yield of 66%. The crude product was purified by column liquid chromatography (SiO_2, *n*-hexane : ethyl acetate, 3:2). (Spectral data for this product matched those reported earlier [34]).

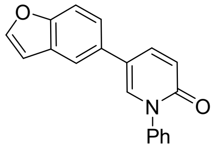

1u

5-(Benzofuran-5-yl)-1-phenylpyridin-2(1*H*)-one (**1u**).
Yield of 32%. The crude product purified by column liquid chromatography (SiO_2, *n*-hexane : ethyl acetate, 2:1) and yielded yellow oil. ^{1}H NMR (400 MHz, $CDCl_3$) δ 6.69 – 6.82 (m, 2H, ArH, CH-3), 7.31 (dd, *J* = 8.5, 2.0 Hz, 1H, ArH), 7.37 – 7.55 (m, 7H, ArH, CH-6), 7.62 (dd, *J* = 5.3, 2.1 Hz, 2H, ArH), 7.70 (dd, *J* = 9.5, 2.7 Hz, 1H, ArH, CH-4). ^{13}C{H} NMR (101 MHz, $CDCl_3$) δ 106.59, 111.84, 118.48 (ArH), 120.55 (Ar), 121.65 (CH-3), 122.55, 126.59 (2C), (ArH), 128.16 (Ar), 128.56, 129.37 (2C), (ArH), 131.27 (Ar), 135.14 (ArH), 140.33 (CH-4), 140.98 (Ar), 145.93 (CH-6), 154.35 (Ar) , 161.61 (C=O). GC-MS (EI= 70EV): m/z= 287 (100), 259 (40), 155 (19), 128 (22), 77 (41). HRMS (ESI-TOF) m/z: $[M + H]^+$ calcd. for $C_{19}H_{14}N0_2$, 288.1025; found, 288.1019.

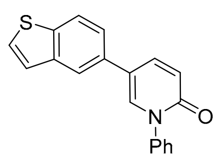

1v

5-(Benzo[b]thiophen-5-yl)-1-phenylpyridin-2(1*H*)-one (**1v**).
Yield of 72%. The crude product, purified by column liquid chromatography (SiO_2, *n*-hexane : ethyl acetate, 1:2), yielded a yellow solid, m.p. = 153–154 °C. ^{1}H NMR (400 MHz, $CDCl_3$) δ 6.79 (dd, *J* = 9.4, 0.7 Hz, 1H, CH-3), 7.28 (dd, *J* = 7.3, 1.0 Hz, 1H, ArH), 7.38 (t, *J* = 7.7 Hz, 1H, ArH), 7.43 (dd, *J* = 5.5, 0.9 Hz, 1H, ArH), 7.45 – 7.55 (m, 6H, ArH), 7.56 (dd, *J* = 2.7, 0.7 Hz, 1H, CH-4), 7.69 (dd, *J* = 9.4, 2.6 Hz, 1H, CH-2), 7.88 (dt, *J* = 8.1, 1.0 Hz, 1H, ArH). ^{13}C{H} NMR (101 MHz, $CDCl_3$) δ 119.72 (Ar), 121.59, 122.15, 122.26, 124.04, 124.46, 126.57(2C), 127.19, 128.64, 129.46 (2C), (ArH), 132.37 (Ar), 136.83 (CH-4), 137.59, 140.76, 140.90 (Ar), 141.48 (CH-2), 161.67 (C=O). GC-MS (EI= 70EV): m/z= 303 (100) [$M^{+\bullet}$], 275 (33), 274 (17), 171 (34), 77 (16). HRMS (ESI-TOF) m/z: $[M + H]^+$ calcd. for $C_{19}H_{14}NOS$, 304.0796; found, 304.0791.

1.3. Synthesis of Substrates **1i–1t**

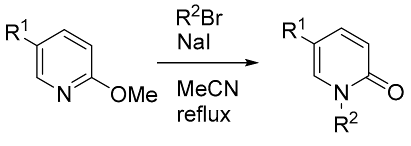

2-methoxypyridines **1i-1t**

Scheme S3. Synthesis of **1i–1t**

Compounds **1i**–**1t** were obtained according to Bowman's and Bridge's method, starting from derivatives of 2-methoxypyridine [36], with some modifications.

Representative procedure for the synthesis of 1,5-dibenzylpyridin-2(1*H*)-one (**1l**).

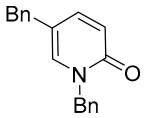

1l

A 50 mL round-bottom flask was charged with 5-benzyl-2-methoxypyridine (0.7 g, 3.51 mmol), benzyl bromide (0.62 mL, 5.27 mmol, 1.5 equiv.), and 19 mL of anhydrous acetonitrile. The mixture was then treated with anhydrous sodium iodide (0.78 g, 5.27 mmol, 1.5 equiv.), and a reflux condenser fitted with an argon balloon was attached to the flask. The reaction was refluxed for 24 hours, and then the reaction mixture was cooled, saturated with sodium chloride solution (20 mL), and extracted with ethyl acetate (3 x 70 mL). The organic layer was dried over anhydrous magnesium sulfate (VI) and filtered off, and the filtrate was concentrated under reduced pressure. The crude product, purified by column chromatography [SiO_2, *n*-hexane, AcOEt (1 : 1)], yielded 0.825 g (yield of 85%) of *N*,5-dibenzyl-2-pyridone (**1l**) as a transparent oil. Spectral data for this product matched those reported earlier [37].)

(In the syntheses of other compounds, 1.5 equiv. of other alkyl bromide and 1.5 equiv. of NaI, along with a proportional amount of solvent, were used.)

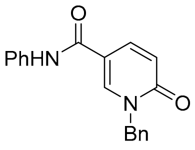

1i

1-Benzyl-6-oxo-*N*-phenyl-1,6-dihydropyridine-3-carboxamide (**1i**).

Yield of 85%. [6-Methoxy-*N*-phenylnicotinamide (0.24 g, 1.05 mmol) was used.] The crude product, purified by column chromatography [SiO_2, *n*-hexane, AcOEt (1:1), then AcOEt], yielded 0.273 g of a white solid, m.p. = 181-182 °C. ^{1}H NMR (400 MHz, $CDCl_3$) δ 5.13 (d, *J* = 2.4 Hz, 2H, NCH_2), 6.54 (d, *J* = 9.5 Hz, 1H, CH-3), 7.10 – 7.17 (m, 1H, ArH), 7.24 – 7.36 (m, 7H, ArH), 7.48 – 7.58 (m, 2H, ArH), 7.70 (dd, *J* = 9.5, 2.6 Hz, 1H, CH-4), 8.03 (br s, 1H, NH), 8.20 (d, *J* = 2.6 Hz, 1H, CH-6). ^{13}C{H} NMR (101 MHz, $CDCl_3$) δ 52.91 (NCH_2), 114.00 (C-5), 119.95 (CH-3), 120.54 (2C), ArH, 124.83, 128.19 (2C), 128.47, 129.07 (2C), 129.11 (2C), (ArH), 135.39 (Ar), 136.49 (CH-4), 137.60 (Ar), 141.07 (CH-6), 162.27 (C=O). GC-MS (EI= 70 eV): m/z= 304 (23) [$M^{+\bullet}$], 212 (55), 91 (100), 65 (12). HRMS (ESI-TOF) m/z: $[M + H]^+$ calcd. for $C_{16}H_{17}N_2O_2$, 305.1290; found, 305.1285.

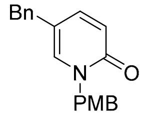

1j

5-Benzyl-1-(4-methoxybenzyl)pyridin-2(1*H*)-one (**1j**).

Yield of 91%. [5-Benzyl-2-methoxypyridine (0.3 g, 1.506 mmol) was used.] The crude product, purified by column chromatography [SiO_2, *n*-hexane, AcOEt (1:1)], yielded 0.418 g of a white solid, m.p. = 72–74 °C. ^{1}H NMR (400 MHz, $CDCl_3$) δ 3.64 (s, 2H, CH_2), 3.78 (s, 3H, OCH_3), 5.04 (s, 2H, NCH_2), 6.55 (d, *J* = 9.3 Hz, 1H, CH-3), 6.82 – 6.89 (m, 2H, ArH), 7.05 (d, *J* = 2.4 Hz, 1H, CH-6), 7.08 – 7.16 (m, 3H, ArH, CH-4), 7.18 – 7.33 (m, 5H, ArH). ^{13}C{H} NMR (101 MHz, $CDCl_3$) δ 37.60 (CH_2), 51.45 (NCH_2), 55.28 (OCH_3), 114.23 (2C), ArH), 118.94 (C-5), 121.08 (CH-3), 126.61 (ArH), 128.54 (Ar), 128.65 (2C), 128.69 (2C), 129.64 (2C), (ArH), 135.03 (CH-4), 139.36 (Ar), 141.23 (CH-6), 159.37 (C=O), 162.04 (Ar). GC-MS (EI= 70 eV): m/z= 305 (30) [$M^{+\bullet}$], 121 (100). HRMS (ESI-TOF) m/z: $[M + H]^+$ calcd. for $C_{20}H_{20}NO_2$, 306.1494; found, 306.1489.

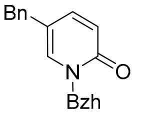

1k

1-Benzhydryl-5-benzylpyridin-2(1*H*)-one (**1k**).

Yield of 80%. [5-Benzyl-2-methoxypyridine (0.6 g, 3.01 mmol) was used.] The crude product, purified by column chromatography [SiO_2, *n*-hexane, AcOEt (3:1)], yielded 0.85 g of a white solid, m.p. = 111–112 °C. ^{1}H NMR (400 MHz, $CDCl_3$) δ 3.60 (s, 2H, 5-CH_2), 6.57 (d, *J* = 9.3 Hz, 1H, CH-3), 6.90 (d, *J* = 2.1 Hz, 1H, CH-6), 6.99 – 7.07 (m, 2H, ArH), 7.08 – 7.38 (m, 14H, ArH, CH-4), 7.51 (s, 1H, NCH). ^{13}C{H} NMR (101 MHz, $CDCl_3$) δ 37.81 (5-CH_2), 61.72 (NCH), 118.25 (Ar), 120.86 (CH-3), 126.53 (ArH), 127.95 (2), 128.54 (2C), 128.65(2C), 128.75 (4C), 128.78 (4C), 133.81 (CH-6), 138.89 (2C), 139.38 (Ar), 140.86 (CH-4), 161.89 (C=O). GC-MS (EI 70eV): m/z= 351 (42) [$M^{+\bullet}$], 167 (100);HRMS (ESI-TOF) m/z: $[M + H]^+$ calcd. for $C_{25}H_{22}NO$, 352.1701; found, 352.1697.

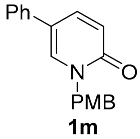

1m

1-(4-Methoxybenzyl)-5-phenylpyridin-2(1*H*)-one (**1m**).

Yield of 93%. [2-Methoxy-5-phenyl-pyridine (0.8 g, 1.08 mmol) was used.] The crude product, purified by column chromatography [SiO_2, *n*-hexane, AcOEt (2:1)], yielded 1.47 g of the product. (Spectral data for this product matched those reported earlier [17].)

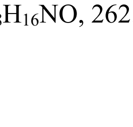

1n

1-Benzhydrylpyridin-2(1*H*)-one (**1n**).

Yield of 70%. [2-Methoxypyridine (1 g, 9.16 mmol) was used.] The crude product, purified by column chromatography [SiO_2, *n*-hexane, AcOEt (2:1)], yielded 1.77 g of a white solid, m.p. = 154–156 °C. (NMR data for this product matched those reported earlier [40].) GC-MS (EI= 70eV): m/z= 261 (21) [$M^{+\bullet}$], 167 (100), 152 (23). HRMS (ESI-TOF) m/z: $[M + H]^+$ calcd. for $C_{18}H_{16}NO$, 262.1232; found, 262.1226.

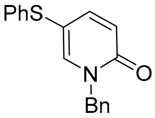

1o

1-Benzyl-5-(phenylthio)pyridin-2(1*H*)-one (**1o**).
Yield of 85%. [5-(Phenylthio)-2-methoxypyridnie (0.3 g, 1.38 mmol) was used.] The crude product, purified by column chromatography [SiO_2, *n*-hexane, AcOEt (3:1)], yielded 0.34 g of white solid, m.p. = 75-77 °C. ^{1}H NMR (400 MHz, $CDCl_3$) δ 5.14 (s, 2H, NCH_2), 6.62 (d, *J* = 9.5 Hz, 1H, =CH-3), 7.08 – 7.21 (m, 3H, ArH), 7.22 – 7.29 (m, 2H, ArH), 7.29 – 7.41 (m, 6H, ArH, =CH-4), 7.57 (d, *J* = 2.5 Hz, 1H, =CH-6). ^{13}C{H} NMR (101 MHz, $CDCl_3$) δ 52.18 (NCH_2), 109.88 (=C-5), 121.97 (=CH-3), 126.27, 127.48 (2C), 128.24 (2C), 128.31, 129.03 (2C), 129.19 (2C), (ArH), 135.79, 137.08, 142.46 (=CH-6), 145.13 (=CH-4), 161.71 (C=O). GC-MS (EI= 70EV): m/z= 293 (100) [$M^{+\bullet}$], 292 (36), 216 (15), 187 (18), 147 (11), 91 (89), 65 (17). HRMS (ESI-TOF) m/z: $[M + H]^+$ calcd. for $C_{18}H_{16}NOS$, 294.0953; found, 294.0947.

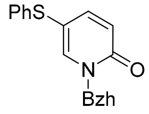

1p

1-Benzhydryl-5-(phenylthio)pyridin-2(1*H*)-one (**1p**).
Yield of 85%. [2-Methoxy-5-phenyl-thiopyridine (0.6 g, 2.76 mmol) was used.] The crude product, purified by column chromatography [SiO_2, *n*-hexane, AcOEt (4:1)], yielded 0.87 g of a white solid, m.p. = 107–108 °C. ^{1}H NMR (400 MHz, $CDCl_3$) δ 6.62 (d, *J* = 9.5 Hz, 1H =CH-3), 7.06 – 7.10 (m, 2H, ArH), 7.13 – 7.20 (m, 5H, =CH-4, ArH), 7.25 – 7.39 (m, 9H, ArH), 7.41 (d, *J* = 2.4 Hz, 1H, =CH-6), 7.47 (s, 1H NCH). ^{13}C{H} NMR (101 MHz, $CDCl_3$) δ 62.27 NCH, 109.47, (=C-5), 121.65 (=CH-3), 126.24, 127.49 (2C), 128.27 (2C), 128.71 (4C), 128.97 (4C), 129.17 (2C), (ArH) 137.14, 138.22 (2C), (Ar), 141.27 (=CH-6), 144.73 (=CH-4), 161.54 (C=O). GC-MS (EI 70eV): m/z= 369 (60) [$M^{+\bullet}$], 168 (29), 167 (100), 165 (54), 152 (31). HRMS (ESI-TOF) m/z: $[M + H]^+$ calcd. for $C_{24}H_{19}NOS$, 370.1266; found, 370.1260.

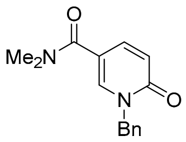

1q

1-Benzyl-*N*,*N*-dimethyl-6-oxo-1,6-dihydropyridine-3-carboxamide (**1q**).
Yield of 68%. [(6-Methoxy-*N*,*N*-dimethylnicotinamide (0.26 g, 1.427 mmol) was used.] The crude product, purified by column chromatography [SiO_2, *n*-hexane, AcOEt (1:1), then AcOEt], yielded 0.259 g of brown oil. ^{1}H NMR (400 MHz, $CDCl_3$) δ 3.01 (s, 6H, $(NCH_3)_2$), 5.15 (s, 2H, NCH_2), 6.58 (d, *J* = 9.4 Hz, 1H, CH-3), 7.28 – 7.38 (m, 4H, ArH), 7.44 (dd, *J* = 9.4, 2.5 Hz, 1H, CH-4), 7.51 – 7.61 (m, 1H, ArH), 7.66 (d, *J* = 2.5 Hz, 1H, CH-6). ^{13}C{H} NMR (101 MHz, $CDCl_3$) δ ~38.00 [v br., $N(CH_3)_2$], 52.34 (NCH_2), 114.77 (C-5), 119.90 (=CH-3), 128.37, 128.43 (2C), 129.06 (2C), (ArH), 135.68 (Ar), 138.47 (CH-4), 139.44 (CH-2), 161.99 (C=O), 167.74 (C=O). GC-MS: m/z= 256 (100) [$M^{+\bullet}$], 212 (37), 91 (100), 65 (13). HRMS (ESI-TOF) m/z: $[M + H]^+$ calcd. for $C_{15}H_{16}N_2O_2$, 257.1290; found, 257.1285.

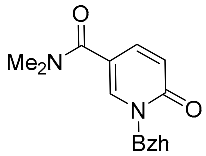

1r

1-Benzhydryl-*N*,*N*-dimethyl-6-oxo-1,6-dihydropyridine-3-carboxamide (**1r**).
Yield of 38%. [6-Methoxy-*N*,*N*-dimethylnicotinamide (0.3204 g, 1.78 mmol) was used.] The crude product, purified by column chromatography [Al_2O_3, AcOEt], yielded 0.224 g of a white solid, m.p. = 204–207 °C. ^{1}H NMR (400 MHz, $CDCl_3$) δ 2.89 [s, 6H, $N(CH_3)_2$], 6.62 (d, *J* = 9.4 Hz, 1H, =CH-3), 7.11 – 7.19 (m, 4H, ArH), 7.30 – 7.39 (m, 6H, ArH), 7.44 (d, *J* = 2.5 Hz, 1H, =CH-6), 7.48 (s, 1H, NCH), 7.51 (dd, *J* = 9.4, 2.5 Hz, 1H, =CH-4). ^{13}C{H} NMR (101 MHz, $CDCl_3$) δ 35.7 (2C), $(NCH_3)_2$, 62.40 (NCH), 113.85 (=C-5), 120.04 (=CH-3), 128.31 (2C), 128.70 (4C), 129.01 (4C), (ArH), 137.82 (=CH-6), 138.20 (2C, Ar), 138.61 (=CH-4), 161.79 (C=O), 167.81 (C=O). GC-MS (EI 70eV): m/z= 332 (20) [$M^{+\bullet}$], 168 (16), 167 (100), 165 (37), 152 (16). HRMS (ESI-TOF) m/z: $[M + H]^+$ calcd. for $C_{21}H_{21}N_2O_2$, 333.1603; found, 333.1598.

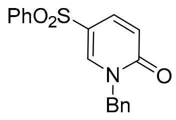

1s

1-Benzyl-5-(phenylsulfonyl)pyridin-2(1*H*)-one (**1s**).
Yield of 78%. [5-(Phenylsulfonyl)-2-methoxypyridine (0.25 g, 1.00 mmol) was used.] The crude product, purified by column chromatography [SiO_2, *n*-hexane, AcOEt (2:1)], yielded 0.254 g of a white solid, m.p. = 165-167 °C. ^{1}H NMR (400 MHz, $CDCl_3$) δ 5.15 (s, 2H, NCH_2), 6.57 (d, *J* = 9.7 Hz, 1H, CH-3), 7.28 – 7.41 (m, 5H, ArH), 7.48 – 7.64 (m, 4H, ArH, CH-4), 7.79 – 7.90 (m, 2H, ArH), 8.17 (d, *J* = 2.7 Hz, 1H, CH-6). ^{13}C{H} NMR (101 MHz, $CDCl_3$) δ 52.97(NCH_2), 120.49 (C-5), 121.45 (=CH-3), 127.30 (2C), 128.33 (2C), 128.69, 129.19 (2C), 129.55 (2C), 133.55 (ArH), 134.85 (Ar), 135.91 (CH-6), 141.00 (Ar), 141.19 (CH-4), 161.41 (C=O). GC-MS: m/z= 325 (46) [$M^{+\bullet}$], 324 (17), 219 (13), 183 (10), 91 (100), 65 (11). HRMS (ESI-TOF) m/z: $[M + H]^+$ calcd. for $C_{18}H_{15}NO_3S$, 326.0851; found, 326.0845.

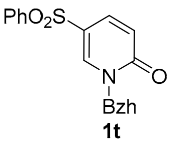

1t

1-Benzhydryl-5-(phenylsulfonyl)pyridin-2(1*H*)-one (**1t**).
Yield of 79%. [2-Methoxy-5-phenylsulfonylpyridine (0.25 g, 1.0 mmol) was used.] The crude product, purified by column chromatography [SiO_2, *n*-hexane, AcOEt (3:1)], yielded 0.521 g of a white solid, m.p. = 122–124 °C. ^{1}H NMR (400 MHz, $CDCl_3$) δ 6.59 (d, *J* = 9.6 Hz, 1H, =CH-3), 7.06 – 7.19 (m, 4H, ArH), 7.34 – 7.43 (m, 7H, =CH-4, ArH), 7.45 – 7.64 (m, 4H, ArH. NCH),

7.67 – 7.76 (m, 2H, ArH), 7.99 (d, *J* = 2.7 Hz, 1H, =CH-6); ^{13}C{H} NMR (101 MHz, $CDCl_3$) δ 63.06 (NCH), 120.28 (=C-5), 121.11 (=CH-3), 127.16 (2C), 128.59 (4C), 128.67 (2C), 129.19 (4C), 129.50 (2C), 133.43 (ArH), 135.48 (=CH-4), 137.39 (2C), (Ar), 140.16 (=CH-6), 141.19 (Ar), 161.22 (C=O). GC-MS (EI 70eV): m/z= 401 (16) [$M^{+\bullet}$], 168 (14), 167 (100), 165 (43), 152 (17). HRMS (ESI-TOF) m/z: $[M + H]^+$ calcd. for $C_{24}H_{20}NO_3S$, 402.1164; found, 402.1158.

1.3.1. Synthesis of 2-Methoxypyridines

Synthesis of **5-benzyl-2-methoxypyridine**.

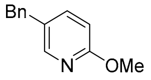

5-benzyl-2-methoxypyridine

To a solution prepared from 5.3 mL of isopropylmagnesium chloride (0.5 equiv., 7.98 mmol, 2 M solution in THF) and 42 mL of anhydrous tetrahydrofuran placed in a 100 mL Schlenk flask and cooled to 0 °C, 6.4 mL of *n*-BuLi (1.0 equiv., 15.96 mmol, 2.5 M solution in hexanes) were added dropwise under argon. The solution was stirred for 10 min at 0 °C, then 5-bromo-2-methoxypyridine (3.0 g, 15.96 mmol) and CuI (0.15 g, 0.8 mmol, 0.05 equiv.) were added. The halogen–magnesium exchange reaction was carried out for 45 min, after which $PhCH_2Br$ [1.0 equiv.] was added and stirred for 0.5 h at 0 °C and then stirred at room temperature for 3.5 h. After this time, a saturated ammonium chloride solution was added to the mixture and extracted with ethyl acetate (3 x 70 mL). The organic layer was dried by shaking over anhydrous magnesium sulfate, and the solvent was evaporated under reduced pressure. The crude product was purified by column chromatography (SiO_2, hexane : AcOEt, 12:1), yielding the product (2.64 g, 83% yield). (Spectral data for this product matched those reported earlier [39].)

Synthesis of **2-methoxy-5-(phenylsulfonyl)pyridine**.

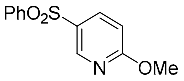

2-methoxy-5-(phenylsulfonyl)pyridine

To a stirred solution of 2-methoxy-5-phenylthiopyridine (1.33 g, 6.12 mmol) in a mixture of methanol (100 mL) and water (10 mL) in a flask cooled in an ice bath, Oxone® was added (2 equiv., 7.54 g) in 3 equal portions (at 0 °C) over 5 minutes. The flask was removed from the bath, and the reaction proceeded for 1 h at room temperature. Then, 200 mL of water were added, and the mixture was stirred overnight at rt. The precipitate that had formed at the top of the flask was filtered off and washed with water and then hexane. The precipitate was dissolved in AcOEt and filtered through a 2 cm pad of silica gel, and the filtrate was concentrated. A total of 1.105 g (72% yield) of the product was obtained as a white solid, for which the spectral data are consistent with the literature data [40].

2. Synthesis of Compounds **4a** and **5a**

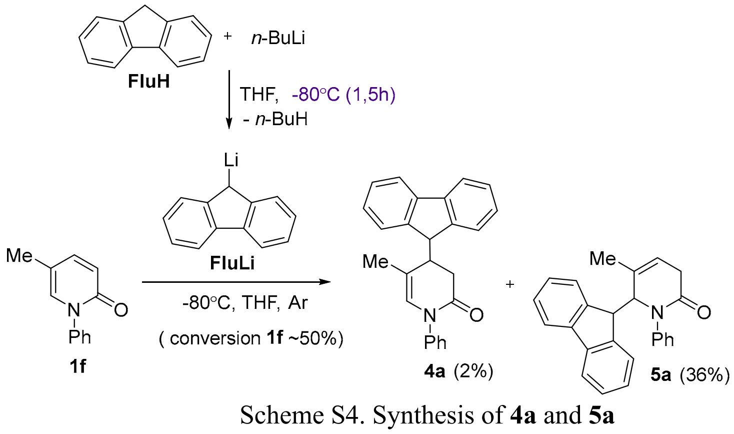

Scheme S4. Synthesis of **4a** and **5a**

In a 25 mL Schlenk flask, 0.437 g of fluorene (1.5 equiv., 2.63 mmol) was dissolved in anhydrous THF (11 mL) under argon. The flask and contents were cooled to –80 °C, and 1.1 mL of *n*-BuLi (1.58 equiv., 2.5 M in hexane) was slowly added dropwise and stirred for 1.5 h. After this time, the contents of the flask were transferred by syringe to a solution of *N*-phenylpyridin-2-one (0.3 g, 1.754 mmol, 1 equiv.) in 11 mL of THF placed in a 50 mL Schlenk flask at –80 °C under argon. The reaction was continued for 3.5 h and then saturated with ammonium chloride solution (5 mL), which was added and warmed to rt. It was extracted with ethyl acetate (3 x 40 mL), dried over anhydrous magnesium sulfate (VI), and filtered, and the solvent was distilled off under reduced pressure. The crude reaction mixture, purified by column chromatography [SiO_2, *n*-hexane : AcOEt (5:1, then 1:1)], yielded 0.0134 g of **4a** (2%) and 0.241 g of **5a** (36%).

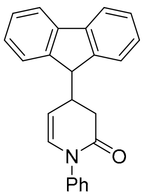
4a

(4*RS*)-4-(9*H*-Fluoren-9-yl)-1-phenyl-3,4-dihydropyridin-2(1*H*)-one (**4a**).

Pink solid, m.p. = 240-242 °C. ^{1}H NMR (400 MHz, $CDCl_3$) δ 2.29 (dd, *J* = 16.4, 9.6 Hz, 1H, CHH-3), 2.43 (ddd, *J* = 16.4, 7.4, 0.9 Hz, 1H, CHH-3), 3.61 (ddtd, *J* = 9.6, 7.4, 3.7, 2.1 Hz, 1H, CH-4), 4.16 (d, *J* = 3.7 Hz, 1H, 4-CH), 5.23 (dd, *J* = 7.9, 3.7 Hz, 1H, =CH-4), 6.27 (dd, *J* = 7.9, 2.1 Hz, 1H, =CH-6), 7.08 – 7.15 (m, 2H, ArH), 7.21 – 7.44 (m, 7H, ArH), 7.53 (dq, *J* = 7.4, 0.9 Hz, 1H, ArH), 7.65 (dq, *J* = 7.5, 0.9 Hz, 1H, ArH), 7.77 (ddt, *J* = 7.6, 2.1, 0.9 Hz, 2H, ArH). ^{13}C{H} NMR (101 MHz, $CDCl_3$) δ 33.97 (CH_2-3), 36.54 (CH-4), 50.57 (4-CH), 108.79, 119.99, 120.07, 124.44, 125.26, 126.00 (2C), 126.96, 127.00, 127.22, 127.60, 127.76, 129.00 (2C), 131.34 (ArH, =CH-5, =CH-6), 140.35, 141.62, 141.99, 143.88, 144.55 (Ar), 168.44 (C=O). GC-MS (EI 70 eV) *m/z*: 337 (<1), ($M^{+\bullet}$), 172 (100) [$M^{+\bullet}$- 165 (fluorenyl radical)], 165 (15). HRMS (ESI-TOF) m/z: [M + H]$^+$ calcd. for $C_{24}H_{19}NO$, 338.1545; found, 338.1539.

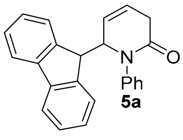

(6*RS*)-6-(9*H*-Fluoren-9-yl)-1-phenyl-3,6-dihydropyridin-2(1*H*)-one (**5a**).

White solid, m.p. = 199-200 °C. ^{1}H NMR (400 MHz, $CDCl_3$) δ 2.88 – 2.98 (m, 2H, CH_2-3), 4.20 (d, *J* = 3.9 Hz, 1H, 6-CH), 4.96 – 5.02 (m, 1H, =CH-4), 5.19 (pd, *J* = 3.9, 1.3 Hz, 1H, CH-6), 5.43 (dtd, *J* = 10.3, 3.9, 3.0, 1.3 Hz, 1H, =CH-5), 7.23 – 7.45 (m, 6H, ArH), 7.49 – 7.58 (m, 4H, ArH), 7.71 – 7.75 (m, 3H, ArH). ^{13}C{H} NMR (101 MHz, $CDCl_3$) δ 33.03 (CH_2-3), 49.83 (6-CH), 65.57 (CH-6), 120.08, 120.14 (ArH), 120.85 (=CH-4), 123.59 (ArH), 123.75 (=CH-5), 124.64, 127.14, 127.22, 127.77 (2C), 127.82, 127.95 (2C), 129.74 (2C), 140.92, 141.54, 142.00, 142.91, 143.24 (Ar), 168.43 (C=O). GC-MS (EI 70 eV) *m/z*: 337 (<1), ($M^{+\bullet}$), 172 (100) [$M^{+\bullet}$- 165 (fluorenyl radical)], 165 (16). HRMS (ESI-TOF) m/z: [M + H]$^+$ calcd. for $C_{24}H_{19}NO$, 338.1545; found, 338.1539.

3. Experimental and Spectral Data for Compounds **12** and **13**

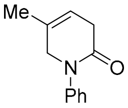
12d

5-Methyl-1-phenyl-3,6-dihydropyridin-2(1*H*)-one (**12d**).

Yield of 22% (Method B). The crude product, purified by column chromatography (SiO_2, hexane/ethyl acetate, 1:1), yielded a yellow oil. HRMS (ESI-TOF) *m/z*: [M + H]$^+$ calcd. for $C_{12}H_{14}NO$, 188.1075; found, 188.1070. GC-MS (EI 70eV): m/z= 187 (100) [$M^{+\bullet}$], 119 (90), 77 (50), 67 (41).

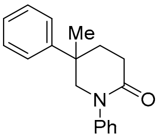
13d

(5*RS*)-5-Methyl-1,5-diphenylpiperidin-2-one (**13d**).

Yield of 27% (Method B). The crude product, purified by column chromatography (SiO_2, hexane/ethyl acetate, 1:1), yielded a yellow oil. ^{1}H NMR (400 MHz, $CDCl_3$) δ 1.41 (s, 3H, CH_3), 2.06 – 2.20 (m, 1H, CHH-4), 2.27 – 2.48 (m, 2H, CHH-3, CHH-4), 2.50 – 2.65 (m, 1H, CHH-3), 3.77 (d, *J* = 12.5 Hz, 1H, CHH-6), 3.97 (dd, *J* = 12.5, 1.8 Hz, 1H, CHH-6), 7.24 – 7.31 (m, 5H, ArH), 7.37 – 7.46 (m, 5H, ArH). ^{13}C{H} NMR (101 MHz, $CDCl_3$) δ 27.74 (CH_3), 29.98, 33.74 (CH_2-3, CH_2-4), 37.93 (C-5), 60.79 (NCH_2), 125.62 (2C), 126.01 (2C), 126.73, 126.89, 128.81 (2C), 129.29 (2C), (ArH), 143.31, 144.51 (Ar), 169.58 (C=O). HRMS (ESI-TOF) *m/z*: [M + H]$^+$ calcd. for $C_{18}H_{20}NO$, 266.1545; found, 266.1539. GC-MS (EI 70eV) *m/z*: 265 (82) [$M^{+\bullet}$], 118 (100), 106 (86), 77 (33).

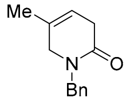
12e

1-Benzyl-5-methyl-3,6-dihydropyridin-2(1*H*)-one (**12e**).

Yield of 9% (Method B). The crude product, purified by column chromatography (SiO_2, hexane/ethyl acetate, 3:1), yielded a yellow oil. ^{1}H NMR (400 MHz, $CDCl_3$) δ 1.66 (s, 3H, 5-CH_3), 2.93 – 3.09 (m, 2H, CH_2-3), 3.62 – 3.79 (m, 2H, CH_2-6), 4.66 (s, 2H, NCH_2), 5.43 (tq, *J* = 3.4, 1.7 Hz, 1H, =CH-4), 7.16 – 7.40 (m, 5H, C_6H_5). ^{13}C{H} NMR (101 MHz, $CDCl_3$) δ 19.77 (5-CH_3), 32.24 (CH_2-3), 49.65 (NCH_2), 52.04 (CH_2-6), 117.23 (=CH-4), 127.51 (ArH), 128.05 (=C-5), 128.17 (2C), 128.66 (2C), (ArH), 136.78 (Ar), 167.51 (C=O). GC-MS (EI 70eV): m/z= 201 (71) [$M^{+\bullet}$], 132 (41), 91 (100), 68 (51). HRMS (ESI-TOF) *m/z*: [M + H]$^+$ calcd. for $C_{13}H_{16}NO$, 202.1232; found, 202.1226.

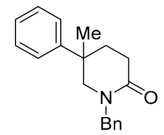

13e

1-Benzyl-5-methyl-5-phenylpiperidin-2-one (**13e**).

Yield of 18% (Method B). The crude product, purified by column chromatography (SiO_2, hexane/ethyl acetate, 3:1), yielded a white semi-solid. ^1H NMR (400 MHz, $CDCl_3$) δ 1.26 (s, 3H, 5-CH_3), 1.91 – 2.02 (m, 1H, C<u>H</u>H-4), 2.17 – 2.30 (m, 2H, CH<u>H</u>-4, C<u>H</u>H-3), 2.40 – 2.52 (m, 1H, CH<u>H</u>-3), 3.23 (d, J = 12.6 Hz, 1H, C<u>H</u>H-6), 3.56 (dd, J = 12.6, 1.7 Hz, 1H, CH<u>H</u>-6), 4.60 (d, J = 14.3 Hz, 1H, NC<u>H</u>H), 4.69 (d, J = 14.3 Hz, 1H, NCH<u>H</u>), 7.05 – 7.12 (m, 2H, ArH), 7.15 – 7.28 (m, 3H, ArH), 7.28 – 7.41 (m, 5H, ArH). $^{13}C\{H\}$ NMR (101 MHz, $CDCl_3$) δ 27.70 (C-5), 29.40 (CH_3), 33.54 (CH_2-3), 37.23 (CH_2-4), 50.37 (NCH_2), 56.60 (CH_2-6), 125.44 (2C), 126.51, 127.61, 128.59 (4C), 128.79 (2C), (ArH), 136.86, 144.56 (Ar), 169.42 (C=O). GC-MS (EI 70 eV) m/z: 279 (75) [$M^{+\bullet}$], 118 (100), 91 (96). HRMS (ESI-TOF) m/z: $[M + H]^+$ calcd. for $C_{19}H_{22}NO$, 280.1701; found, 280.1696.

4. Structural Analysis of Bridged δ-Lactams **8**, **9**, and **16**

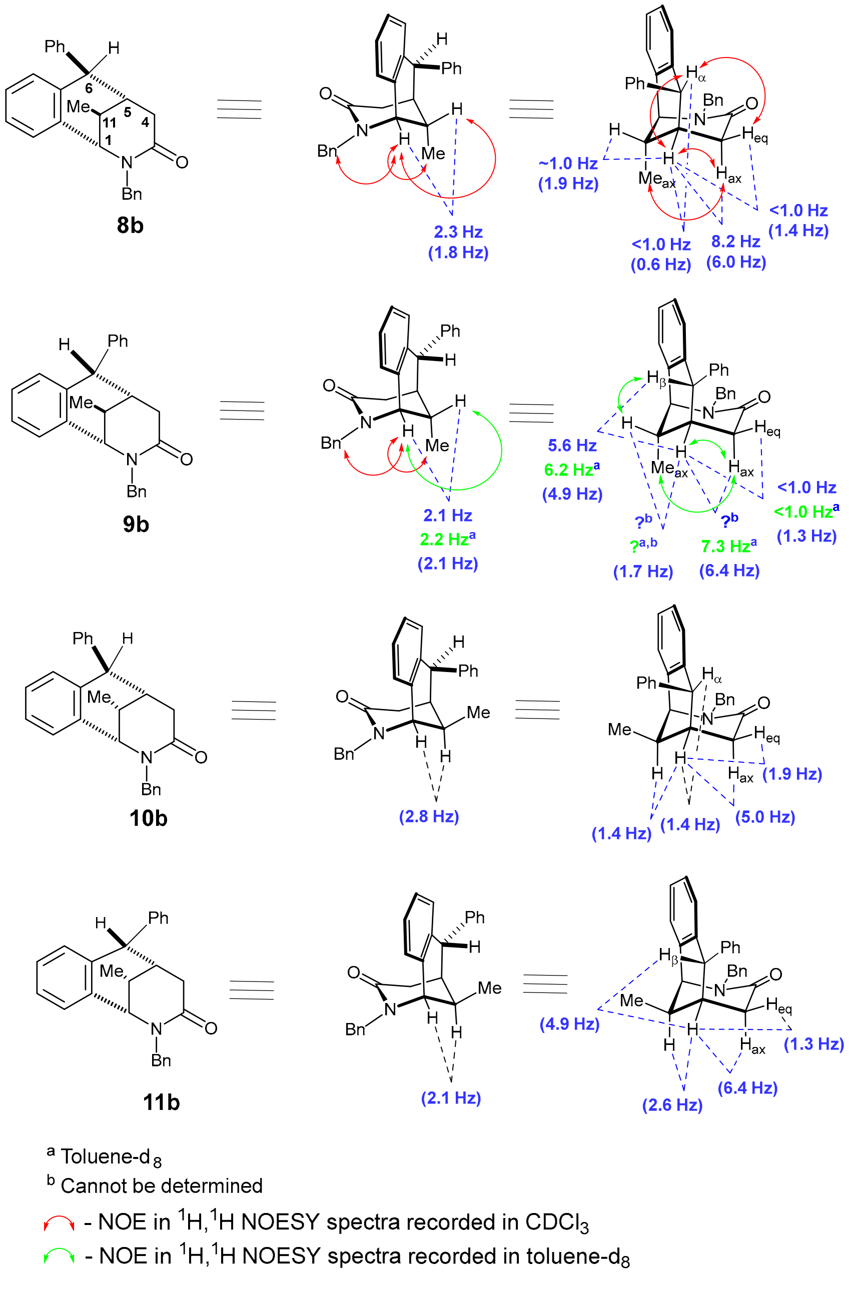

Figure S1. $J^3_{H,H}$ refined from ^1H NMR spectra and calculated based on the Haasnoot equation [28] (in round brackets) using structures optimized by the PM3 method for all possible isomers **8**–**11**, together with the

Overhouser effects derived from ^{1}H,^{1}H NOESY spectra for **8** and **9**. (Only one enantiomer is shown for simplicity).

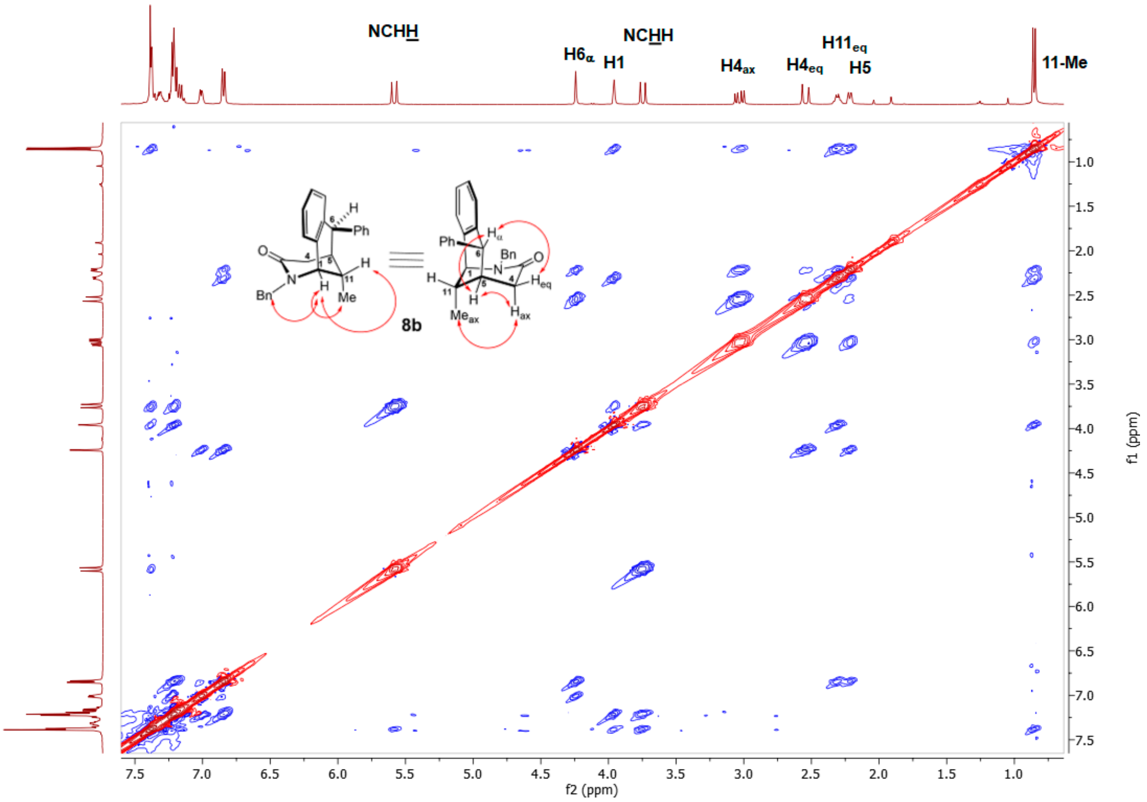

Figure S2. ^{1}H,^{1}H NOESY spectra of compound **8b** ($CDCl_3$).

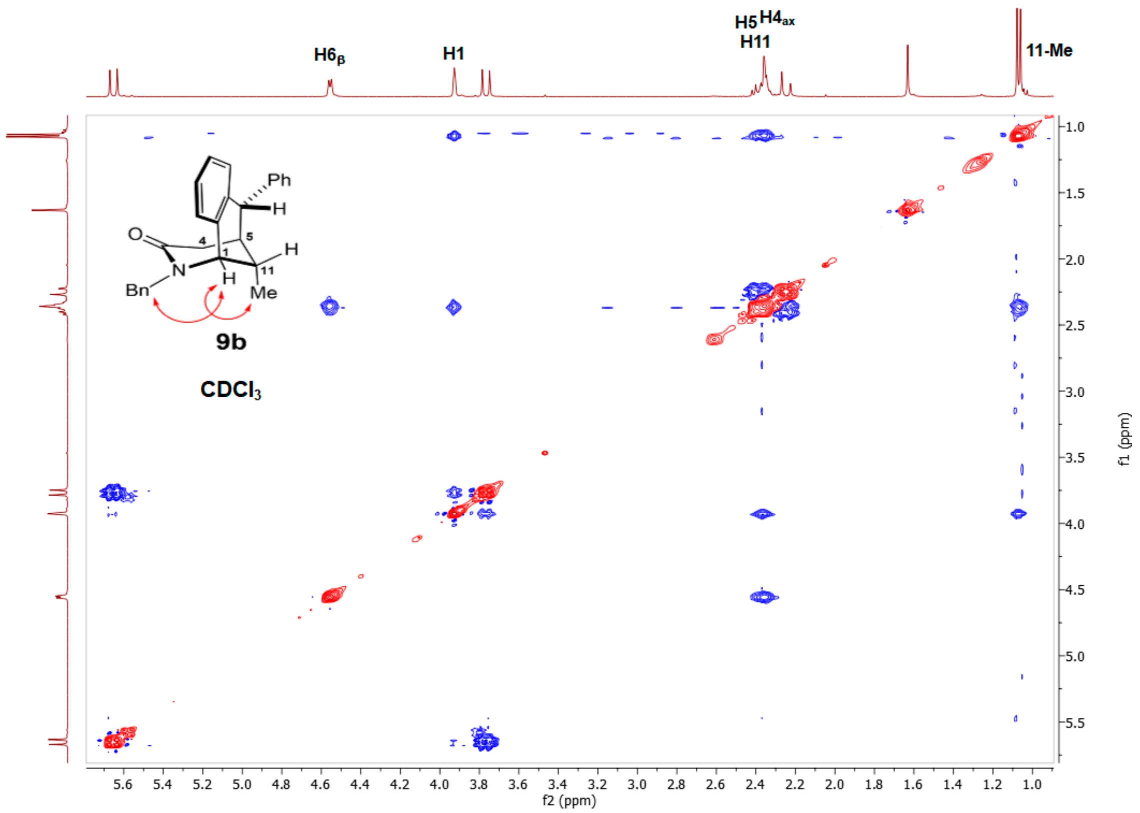

Figure S3. $^{1}H,^{1}H$ NOESY spectra of compound **9b** ($CDCl_3$).

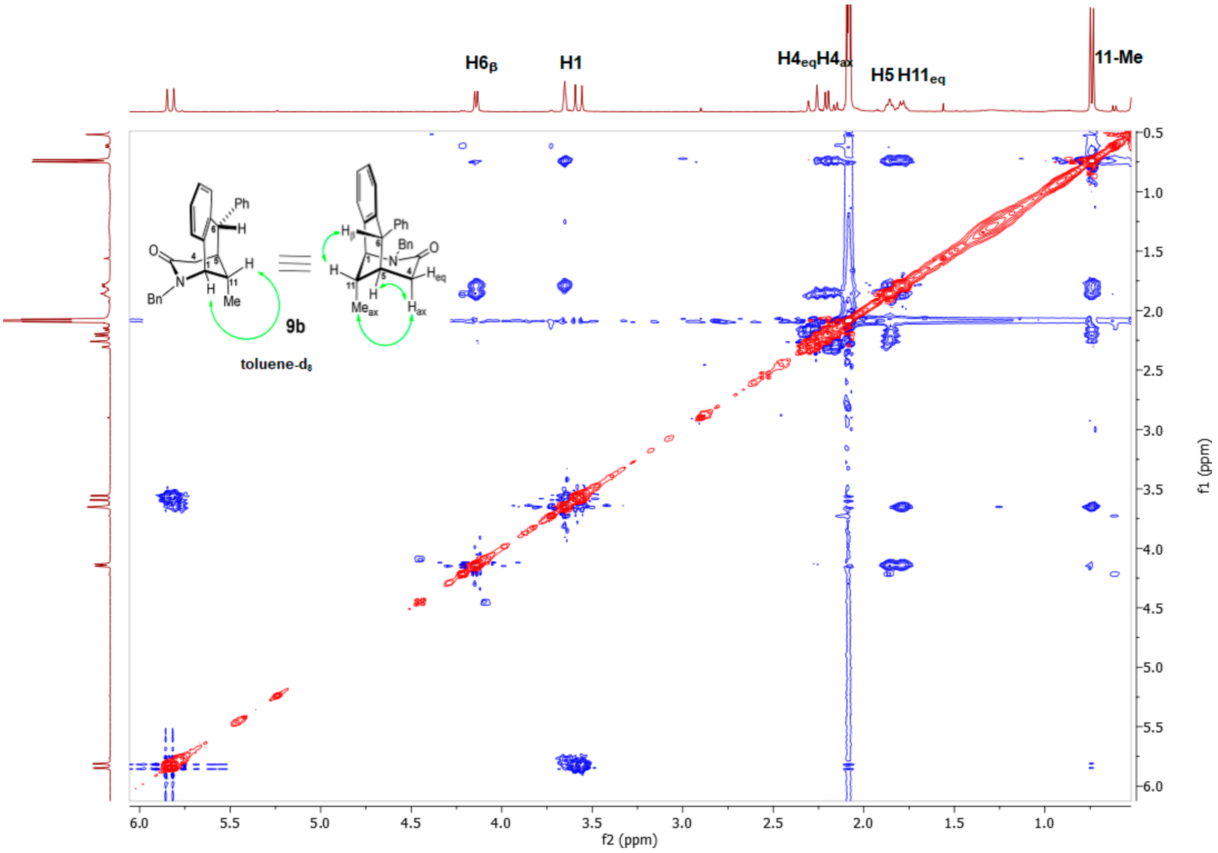

Figure S4. $^{1}H,^{1}H$ NOESY spectra of compound **9b** (toluene-d_8).

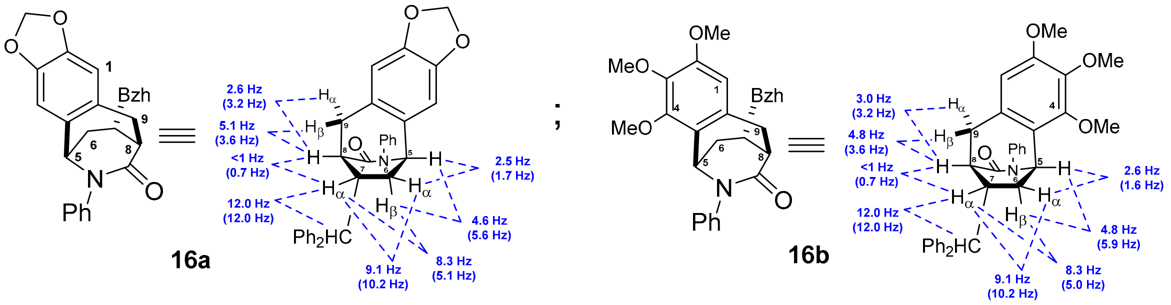

Figure S5. $J^3_{H,H}$ refined from ^1H NMR spectra and calculated based on the Haasnoot equation [28] (in round brackets) using structures optimized by the PM3 method for compounds **16a** and **16b**. (Only one enantiomer is shown for simplicity).

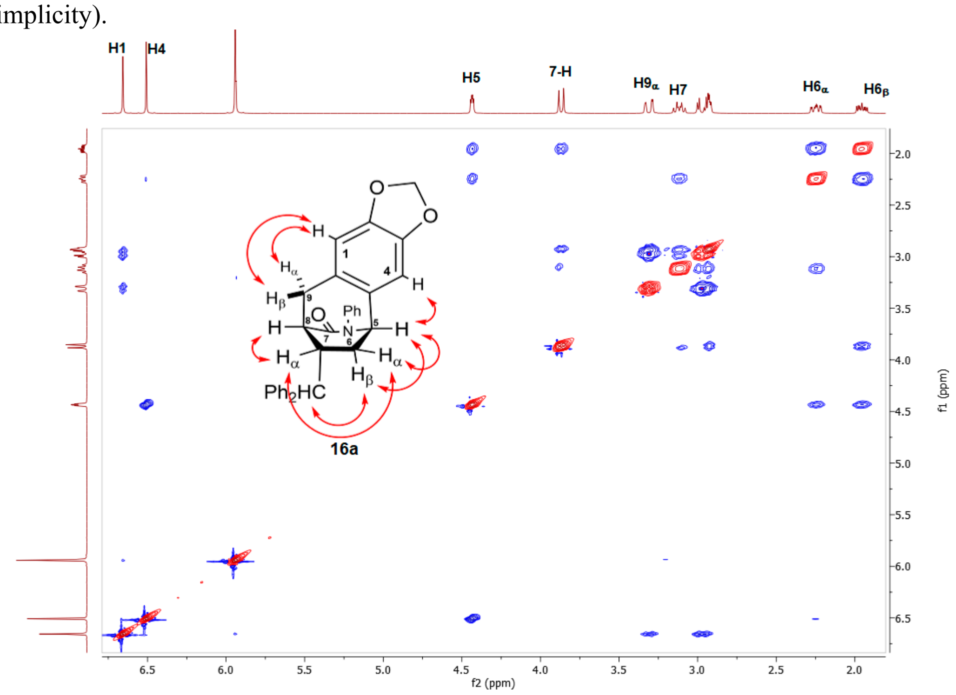

Figure S6. ^1H,^1H NOESY spectra of compound **16a** ($CDCl_3$).

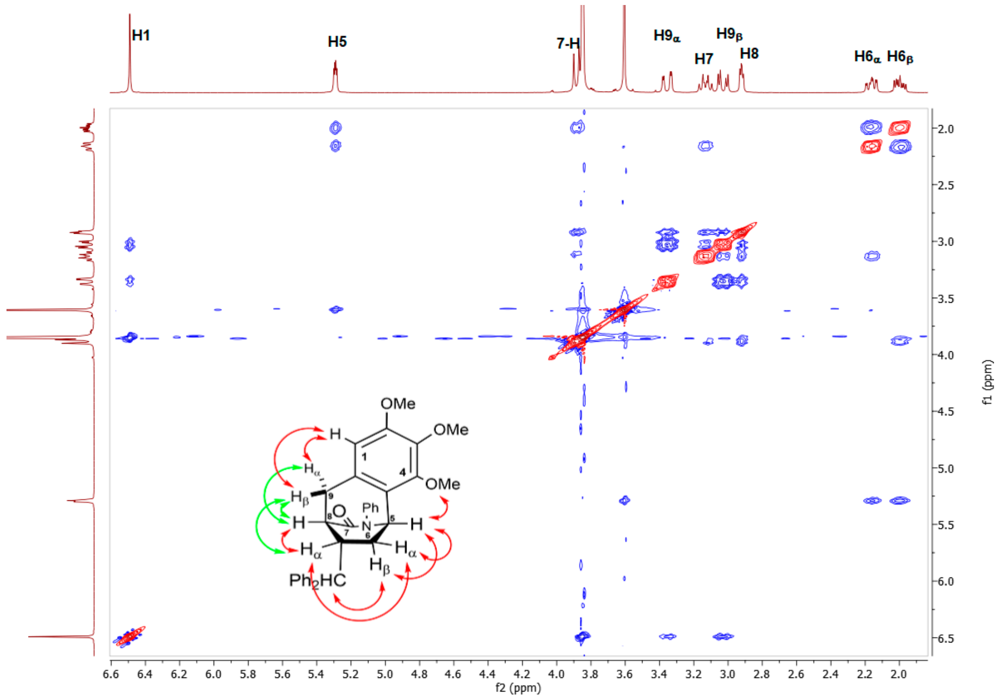

Figure S7. ^{1}H,^{1}H NOESY spectra of compound **16b** ($CDCl_3$).

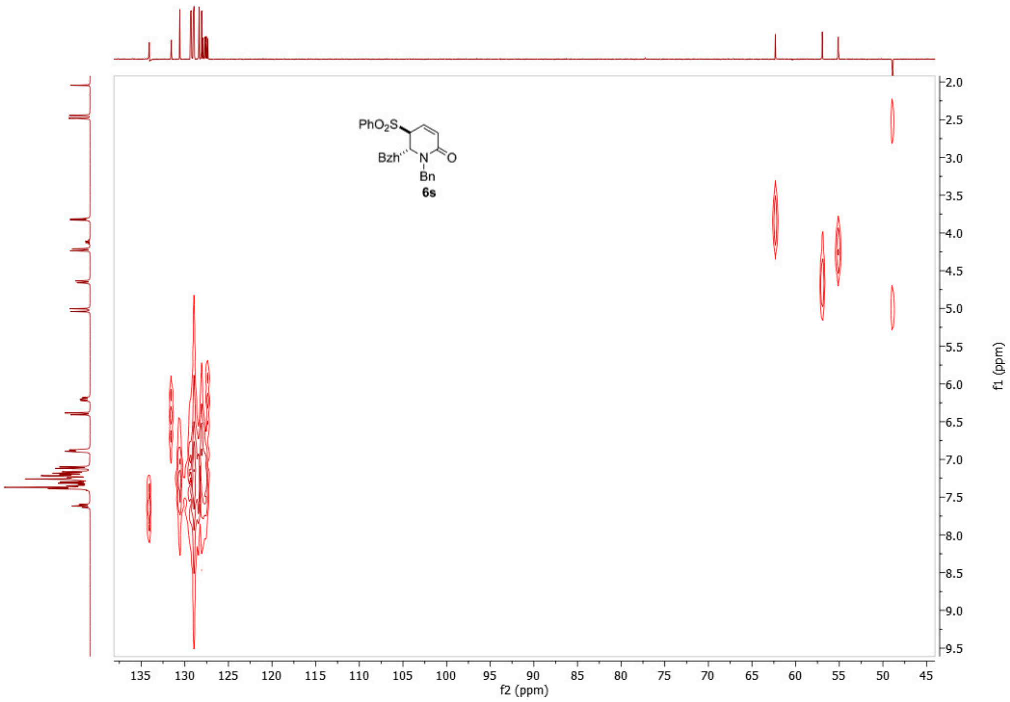

Figure S8. ^{13}C,^{1}H HETCOR spectra of compound **6s** ($CDCl_3$).

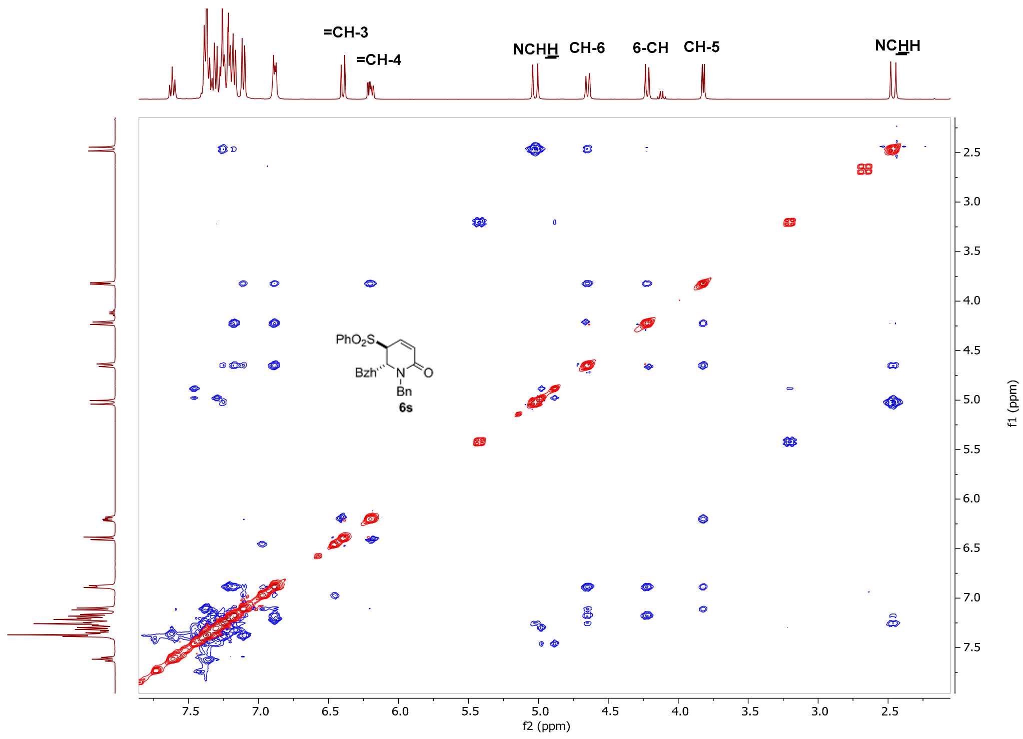

Figure S9. $^{1}H,^{1}H$ NOESY spectra of compound **6s** ($CDCl_3$).

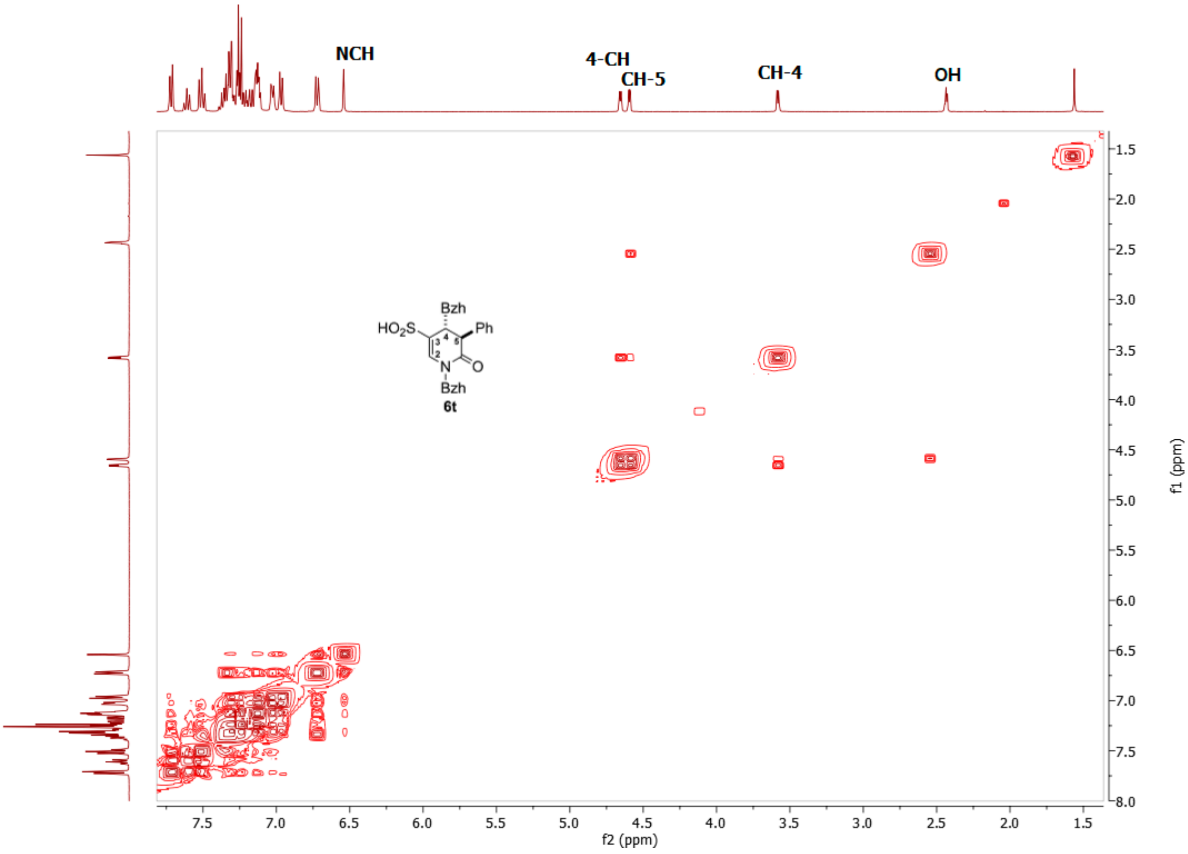

Figure S10. $^{1}H,^{1}H$ DQFCOSY spectra of compound **6t** ($CDCl_3$).

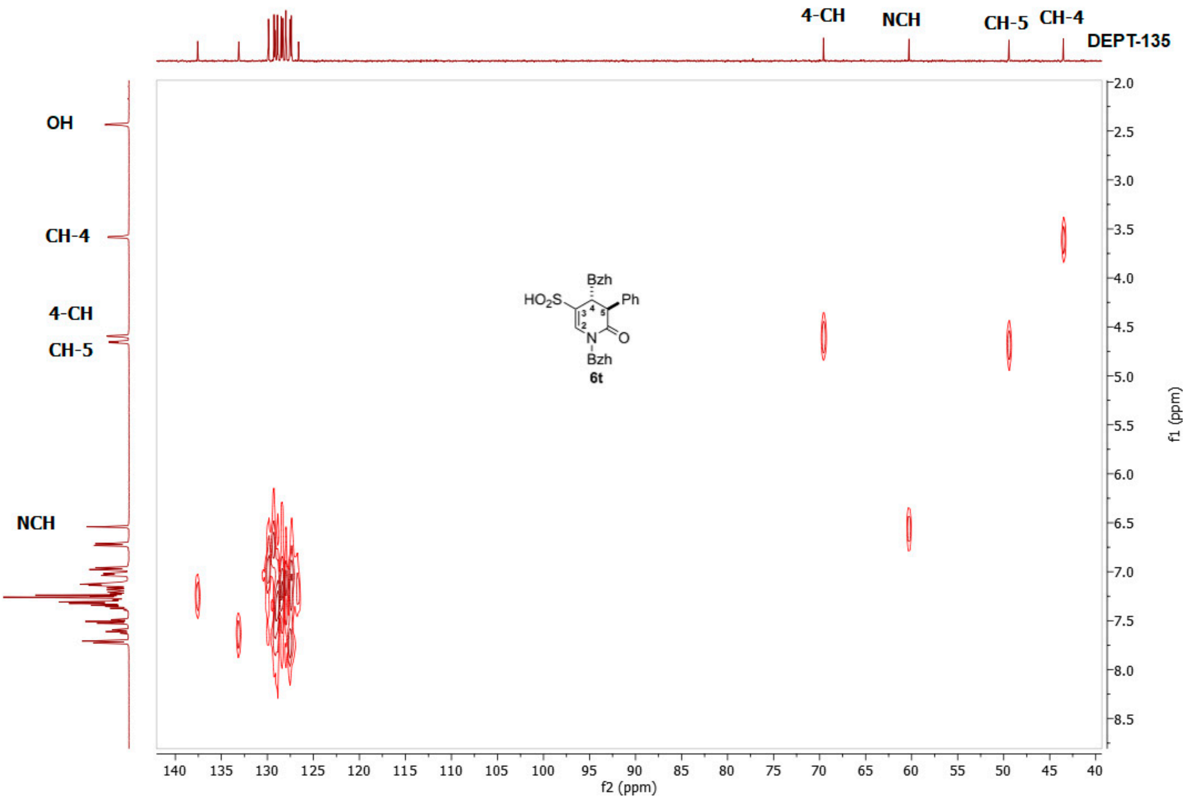

Figure S11. ^{13}C,^{1}H HETCOR spectra of compound **6t** ($CDCl_3$).

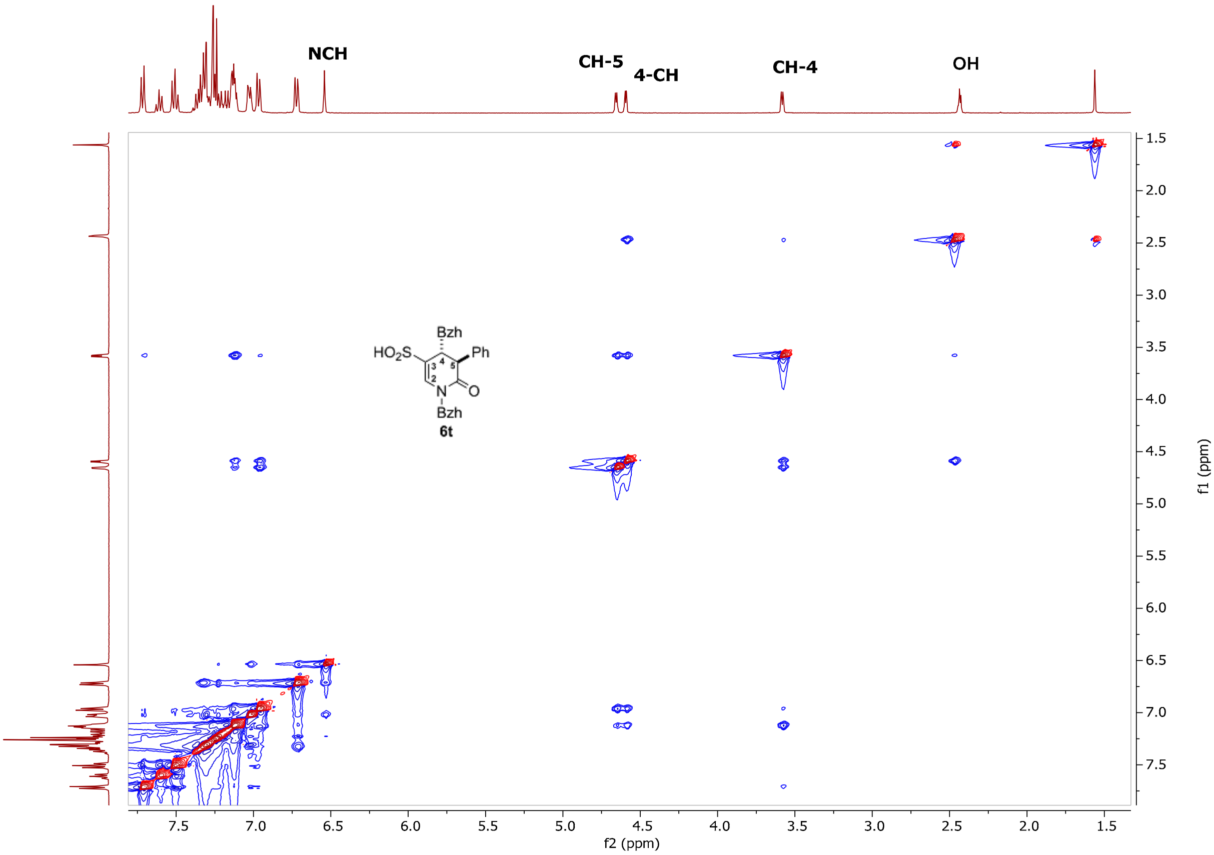

Figure S12. ^{1}H,^{1}H NOESY spectra of compound **6t** ($CDCl_3$).

5. ^{1}H and ^{13}C Spectra for Compounds **1–16**

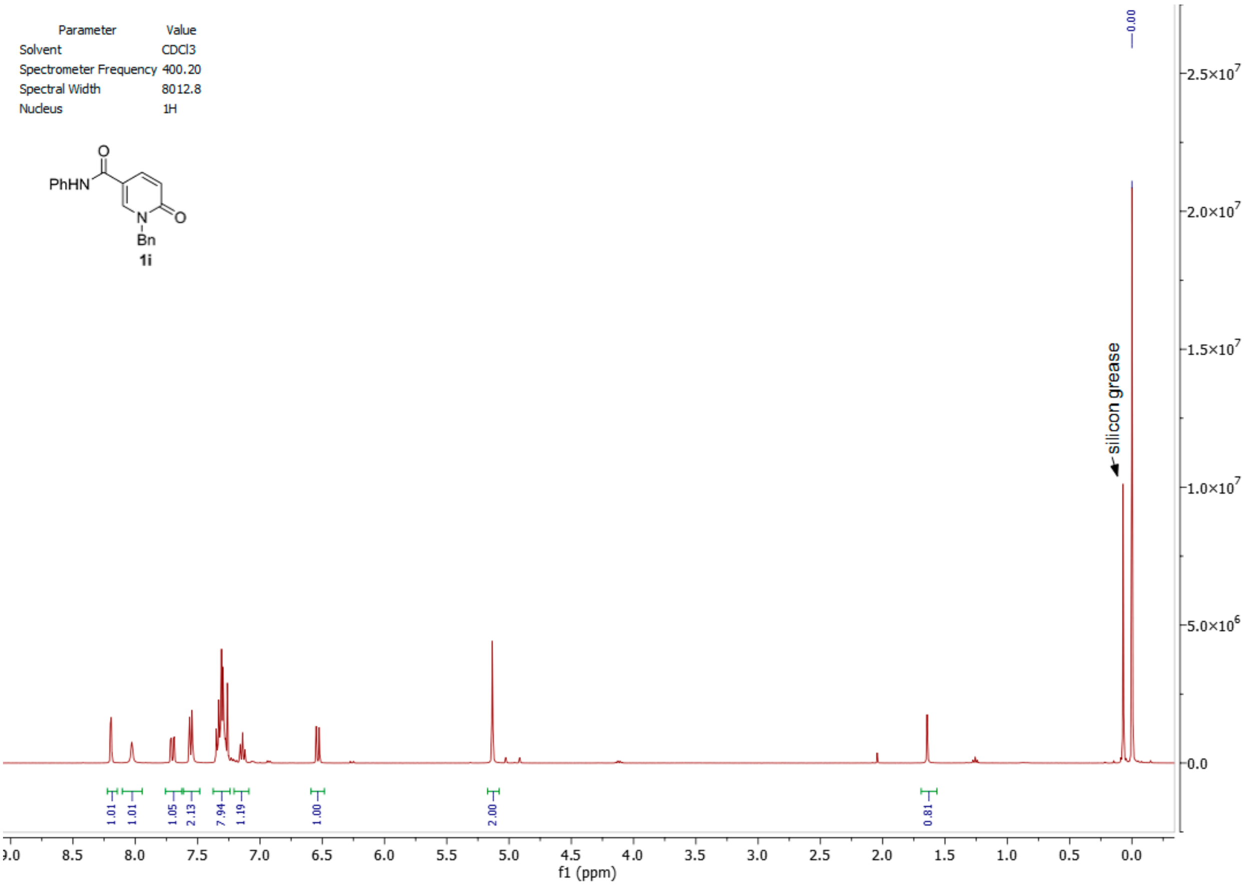

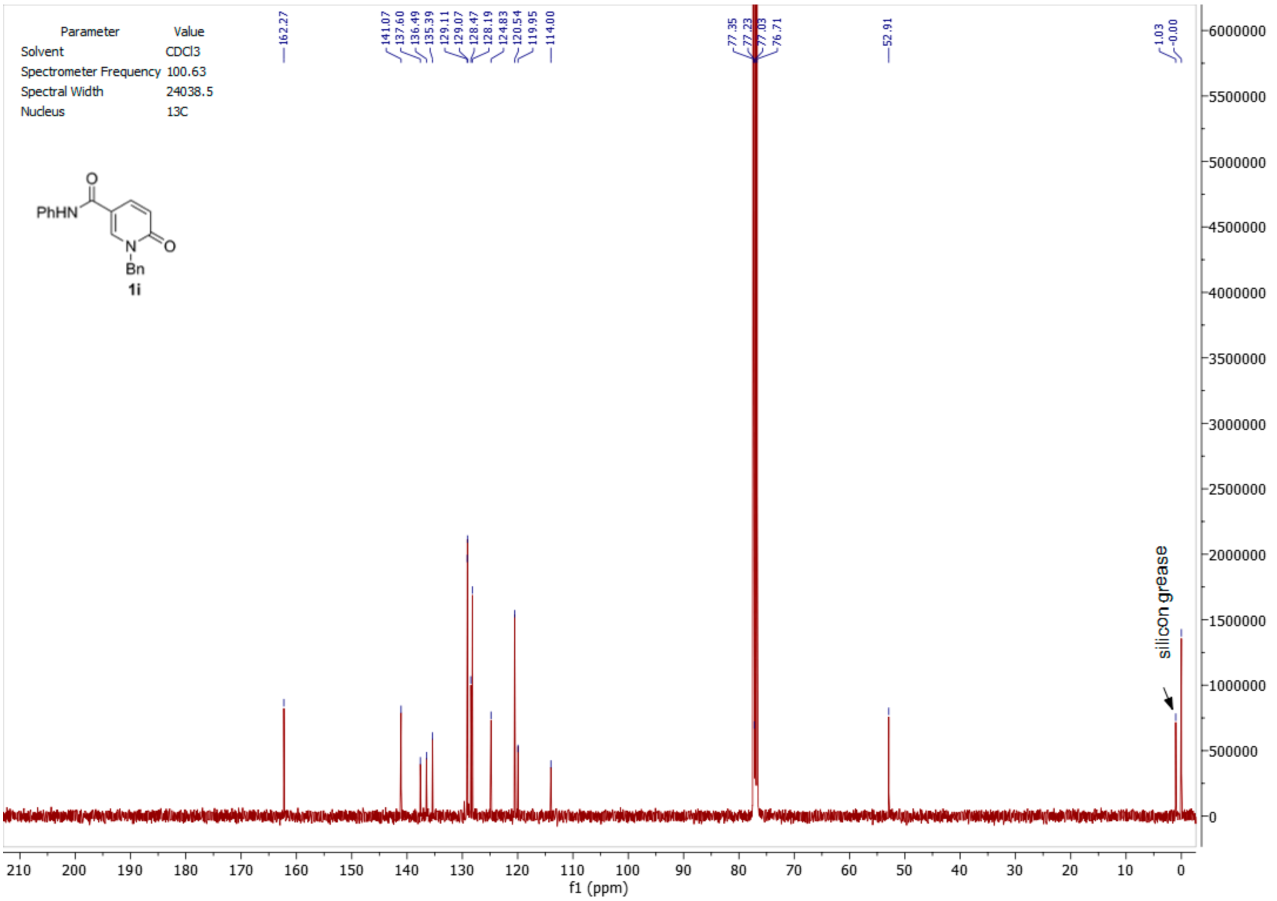

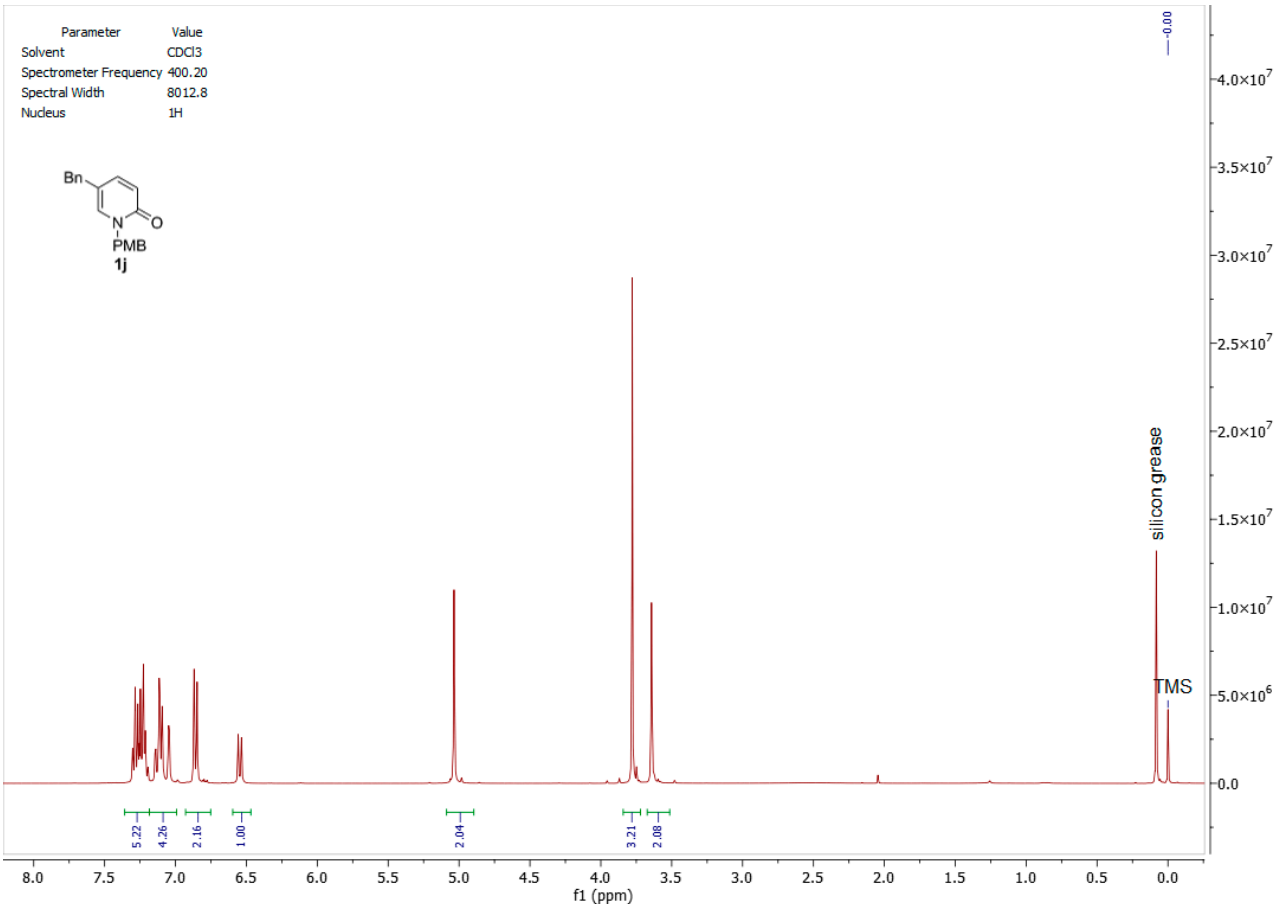

Parameter Value
Solvent CDCl3
Spectrometer Frequency 400.20
Spectral Width 8012.8
Nucleus 1H
Bn
N
O
PMB
1j
-0.00
silicon grease
TMS
5.22
4.26
2.16
1.00
2.04
3.21
2.08
f1 (ppm)

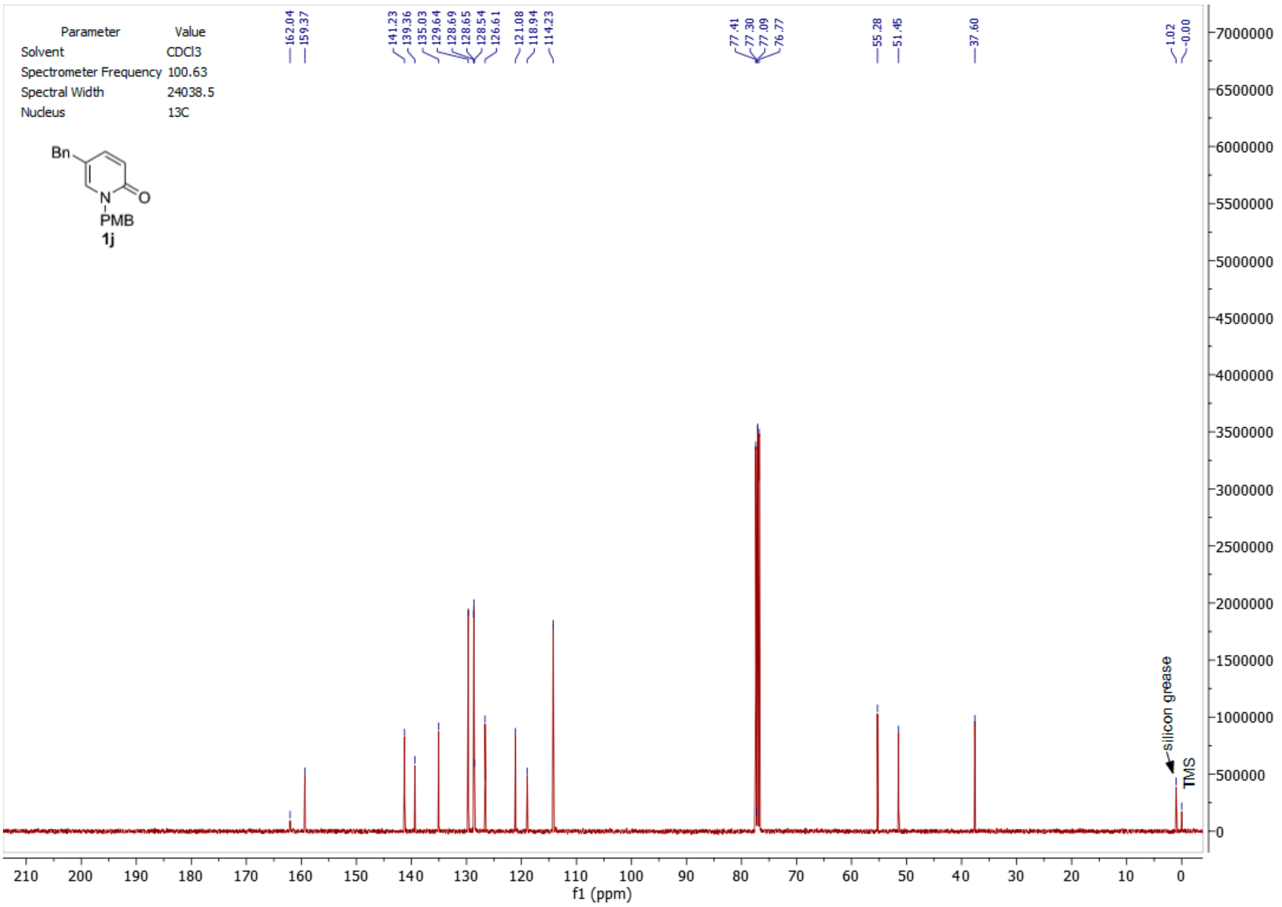

Parameter Value
Solvent CDCl3
Spectrometer Frequency 100.63
Spectral Width 24038.5
Nucleus 13C
Bn
N
O
PMB
1j
162.04
159.37
141.23
139.36
135.03
129.64
128.69
128.65
128.54
126.61
121.08
118.94
114.23
77.41
77.30
77.09
76.77
55.28
51.45
37.60
1.02
-0.00
silicon grease
TMS
f1 (ppm)

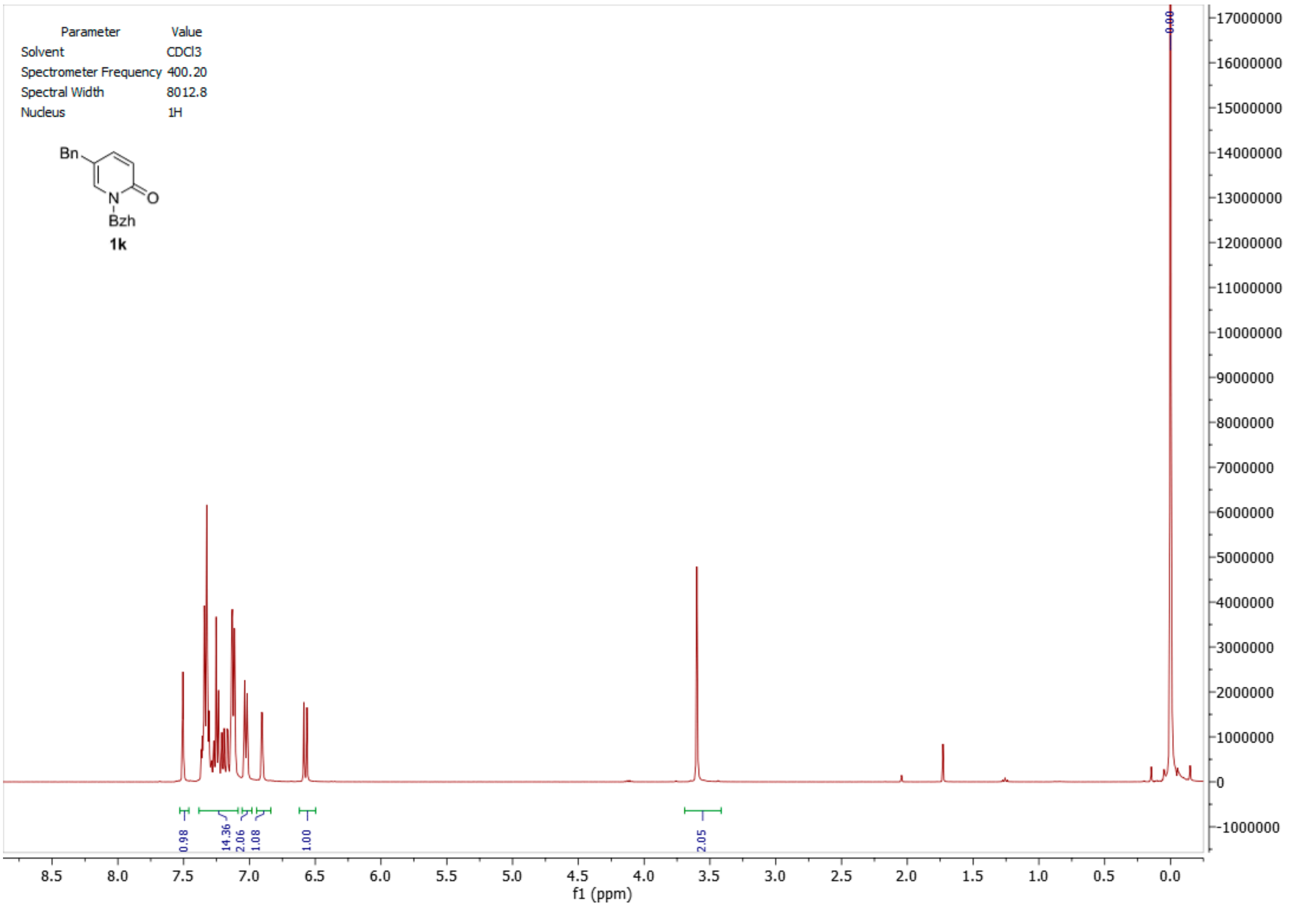

Parameter Value
Solvent CDCl3
Spectrometer Frequency 400.20
Spectral Width 8012.8
Nucleus 1H
Bn
N
O
Bzh
1k
0.98
14.36
2.06
1.08
1.00
2.05
f1 (ppm)

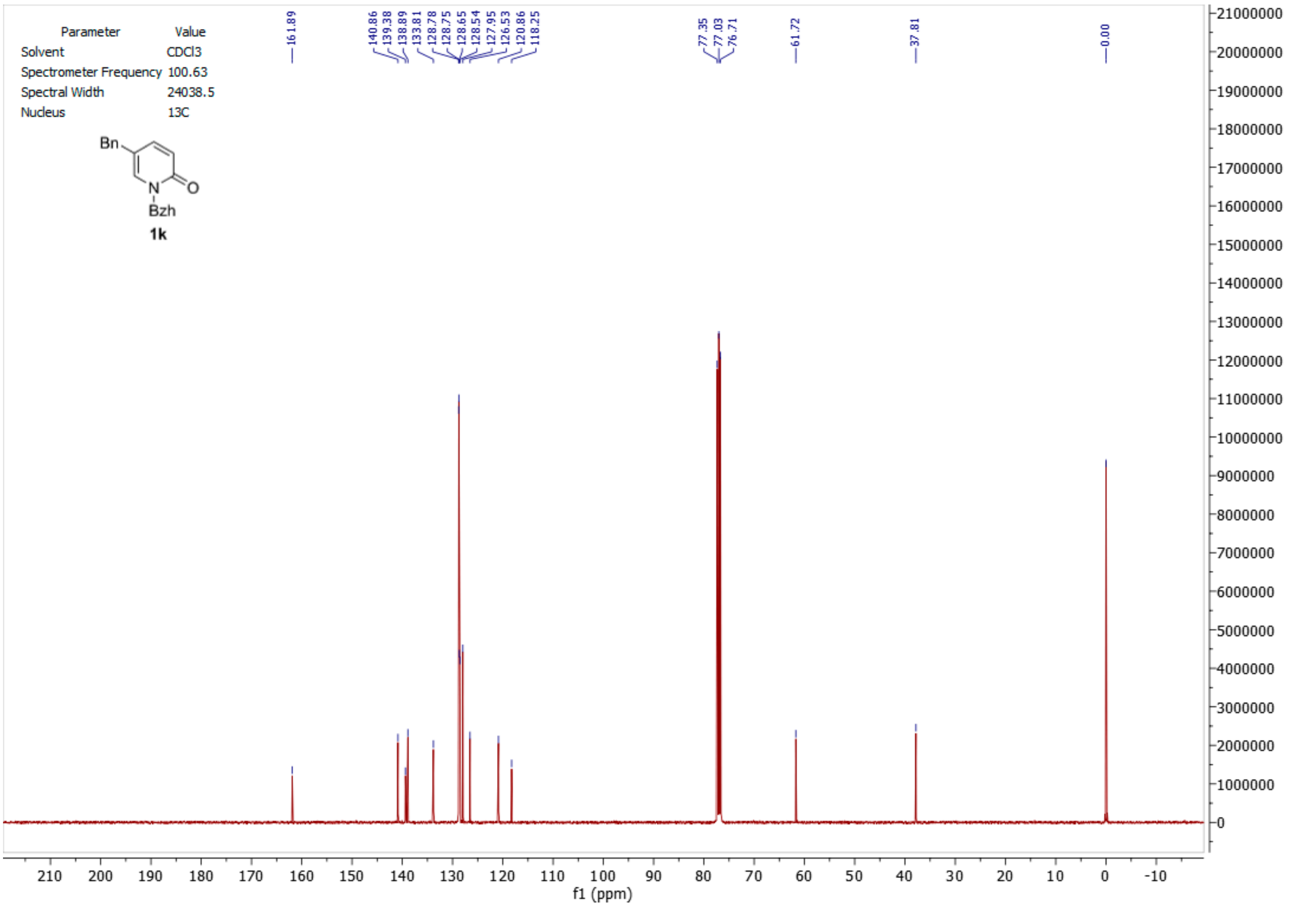

Parameter Value
Solvent CDCl3
Spectrometer Frequency 100.63
Spectral Width 24038.5
Nucleus 13C
Bn
N
O
Bzh
1k
161.89
140.86
139.38
138.89
133.81
128.78
128.75
128.65
128.54
127.95
126.53
120.86
118.25
77.35
77.03
76.71
61.72
37.81
0.00
f1 (ppm)

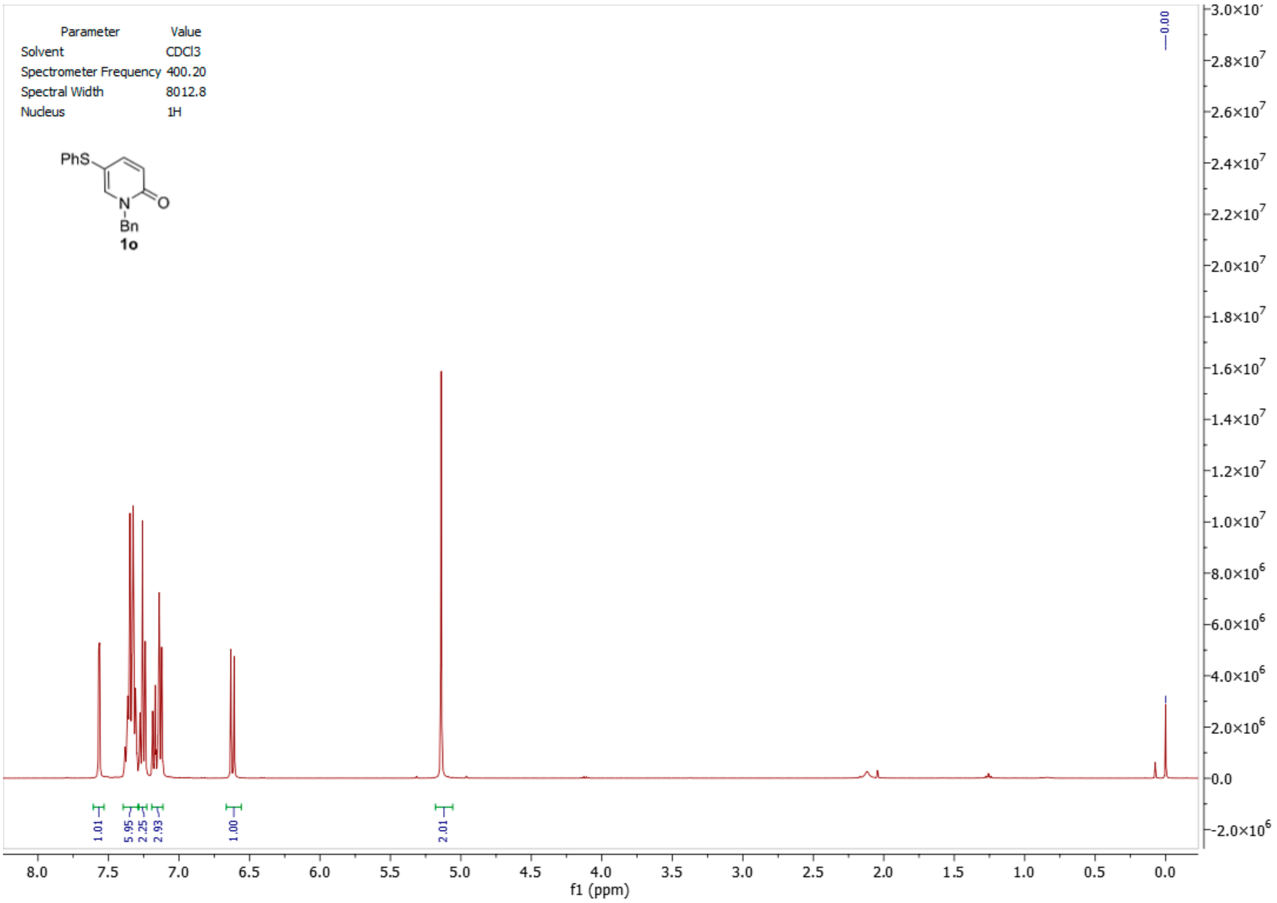
Parameter Value
Solvent CDCl3
Spectrometer Frequency 400.20
Spectral Width 8012.8
Nucleus 1H
PhS
N
O
Bn
1o
f1 (ppm)

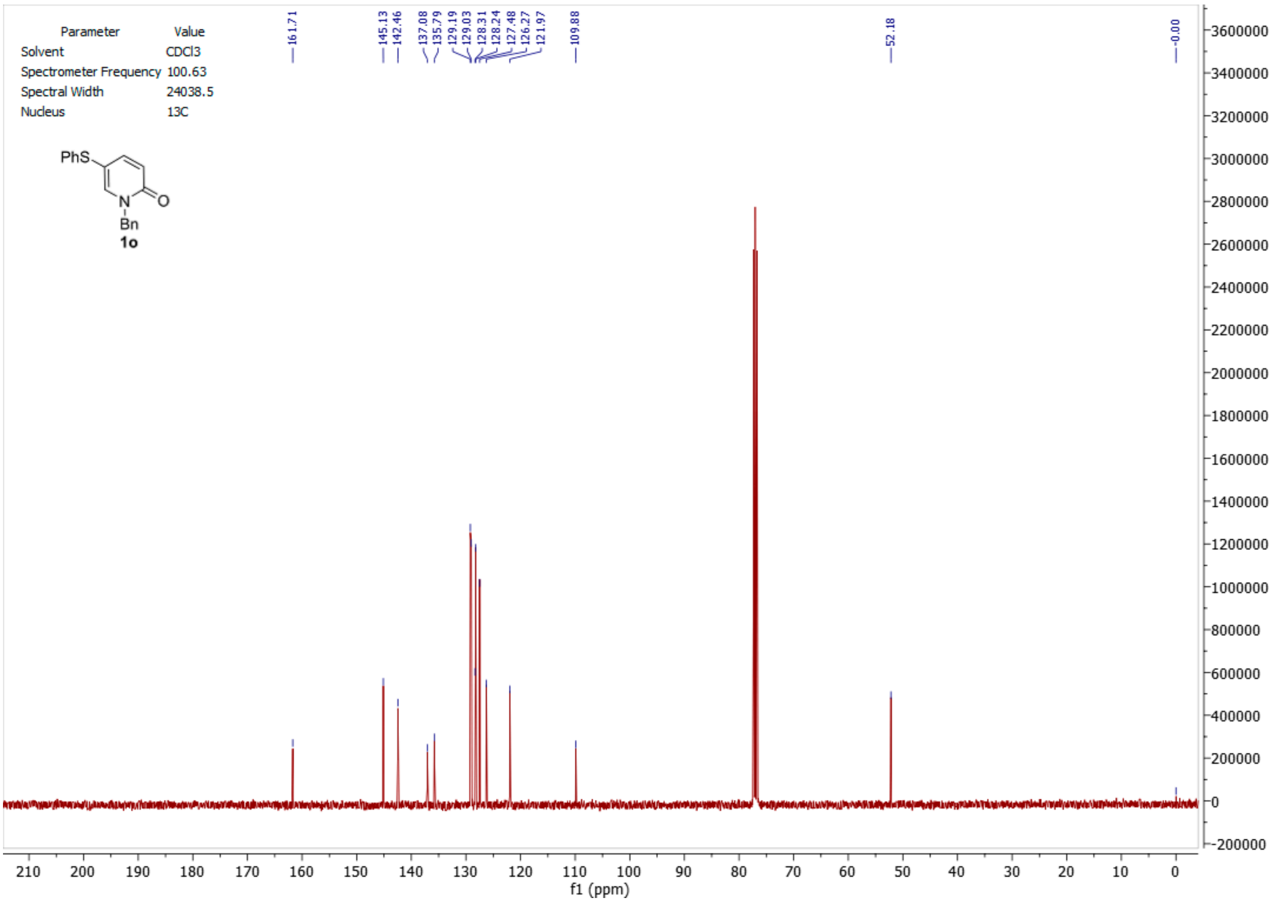
Parameter Value
Solvent CDCl3
Spectrometer Frequency 100.63
Spectral Width 24038.5
Nucleus 13C
PhS
N
O
Bn
1o
161.71
145.13
142.46
137.08
135.79
129.19
129.03
128.31
128.24
127.48
126.27
121.97
109.88
52.18
-0.00
f1 (ppm)

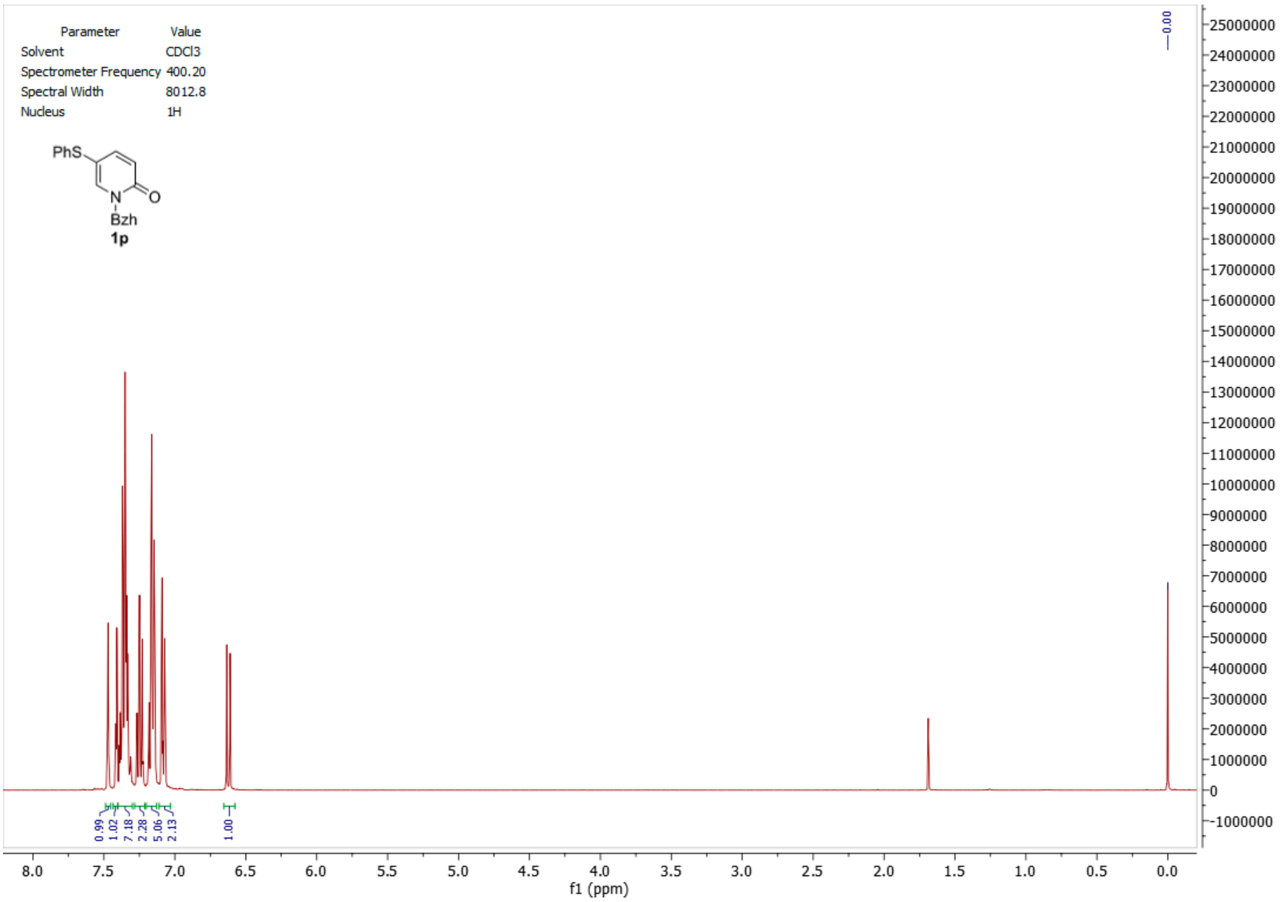

Parameter Value
Solvent CDCl3
Spectrometer Frequency 400.20
Spectral Width 8012.8
Nucleus 1H
PhS
N
O
Bzh
1p
f1 (ppm)

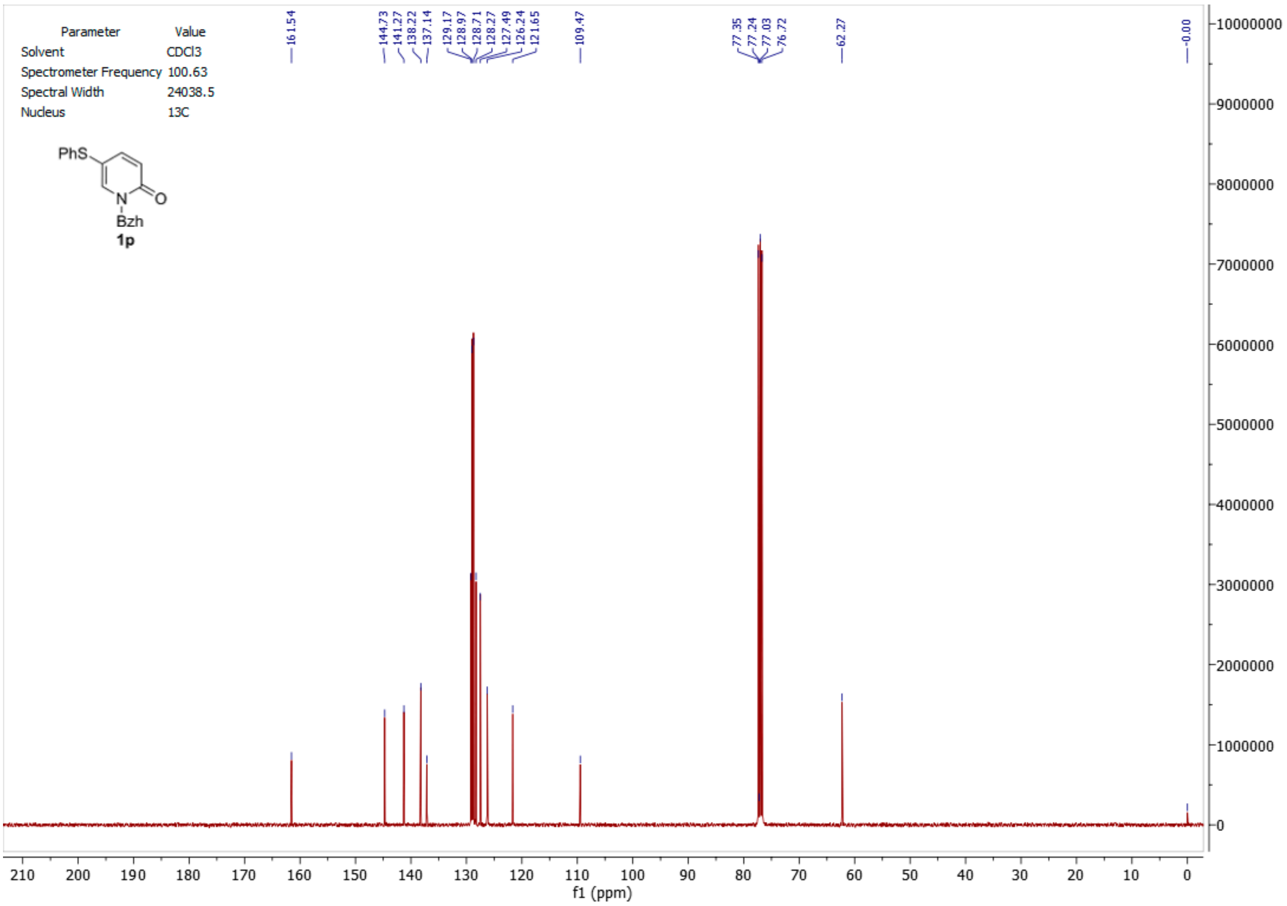

Parameter Value
Solvent CDCl3
Spectrometer Frequency 100.63
Spectral Width 24038.5
Nucleus 13C
PhS
N
O
Bzh
1p
161.54
144.73
141.27
138.22
137.14
129.17
128.97
128.71
128.27
127.49
126.24
121.65
109.47
77.35
77.24
77.03
76.72
62.27
0.00
f1 (ppm)

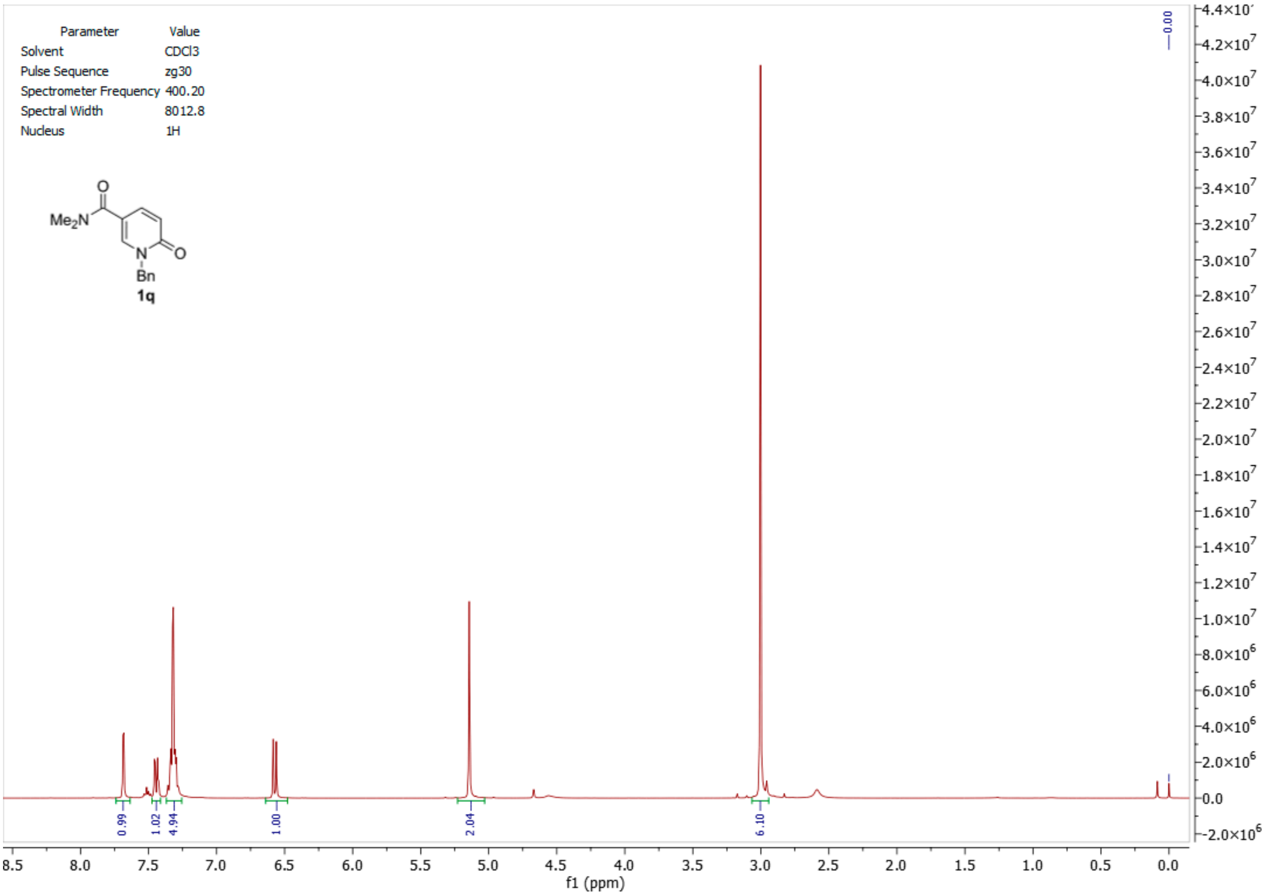

Parameter Value
Solvent CDCl3
Pulse Sequence zg30
Spectrometer Frequency 400.20
Spectral Width 8012.8
Nucleus 1H
Me2N
O
N
Bn
1q
0.99 1.02 4.94 1.00 2.04 6.10
0.00
f1 (ppm)

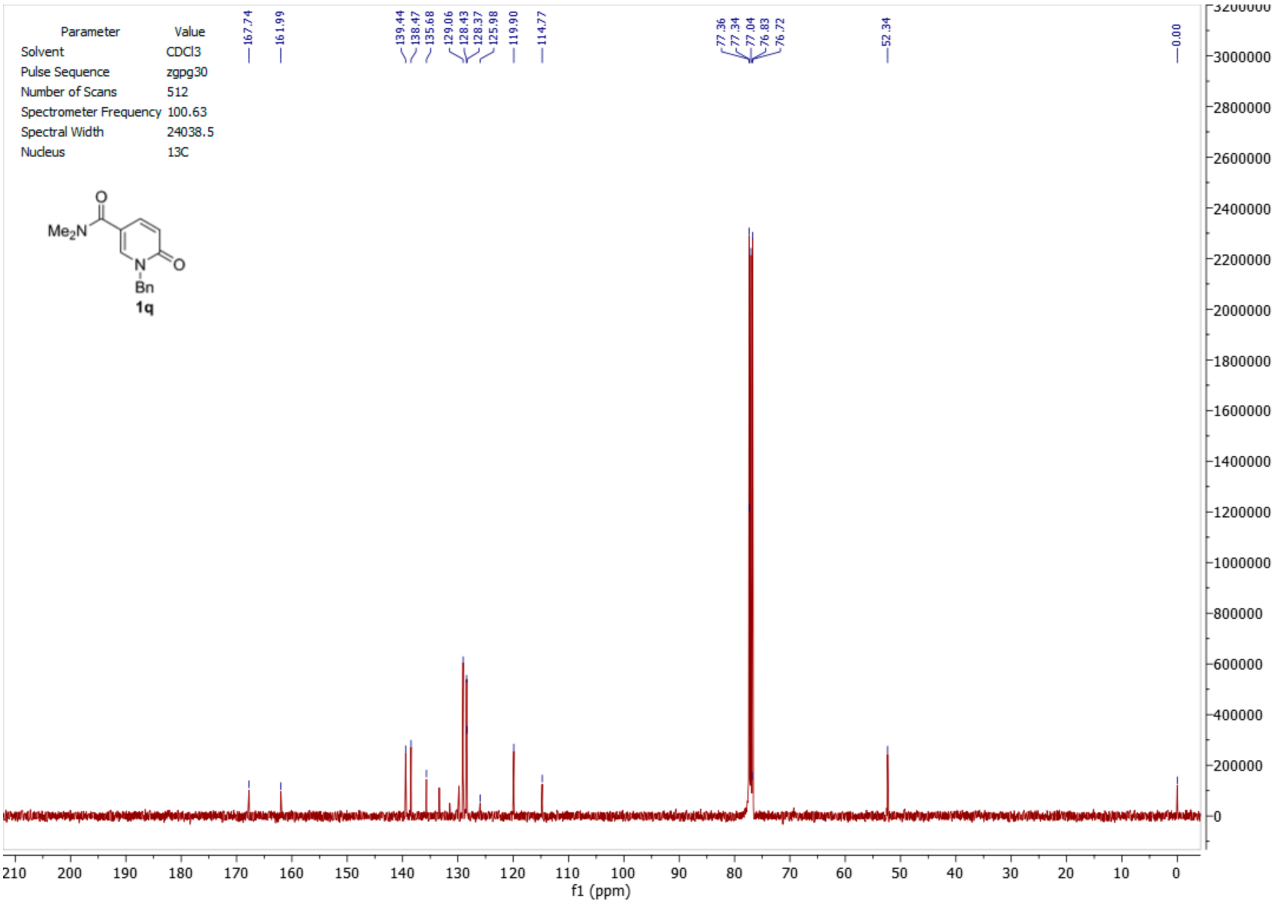

Parameter Value
Solvent CDCl3
Pulse Sequence zgpg30
Number of Scans 512
Spectrometer Frequency 100.63
Spectral Width 24038.5
Nucleus 13C
Me2N
O
N
Bn
1q
167.74
161.99
139.44
138.47
135.68
129.06
128.43
128.37
125.98
119.90
114.77
77.36
77.34
77.04
76.83
76.72
52.34
0.00
f1 (ppm)

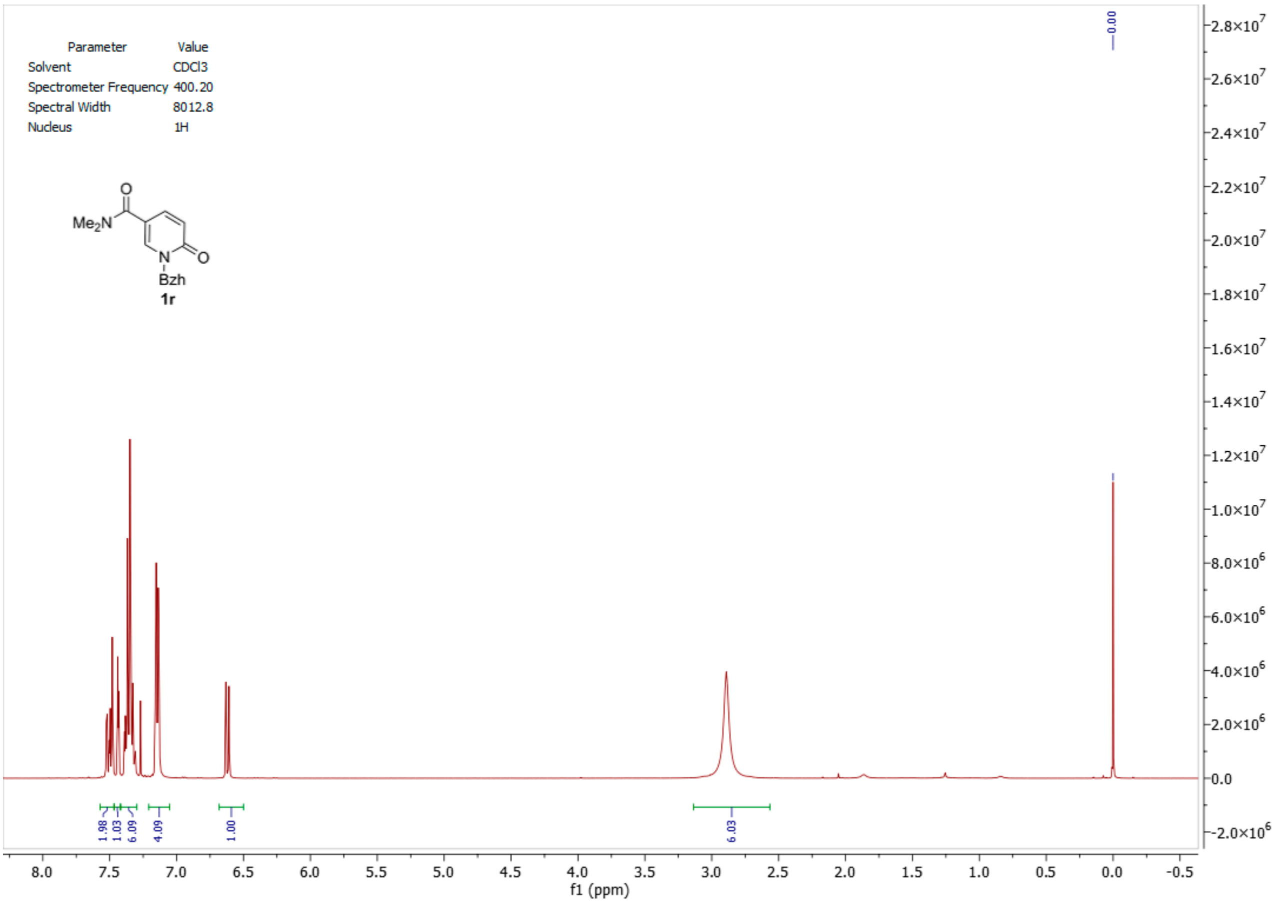
Parameter Value
Solvent CDCl3
Spectrometer Frequency 400.20
Spectral Width 8012.8
Nucleus 1H
Me2N
O
N
O
Bzh
1r
0.00
1.98
1.03
6.09
4.09
1.00
6.03
f1 (ppm)

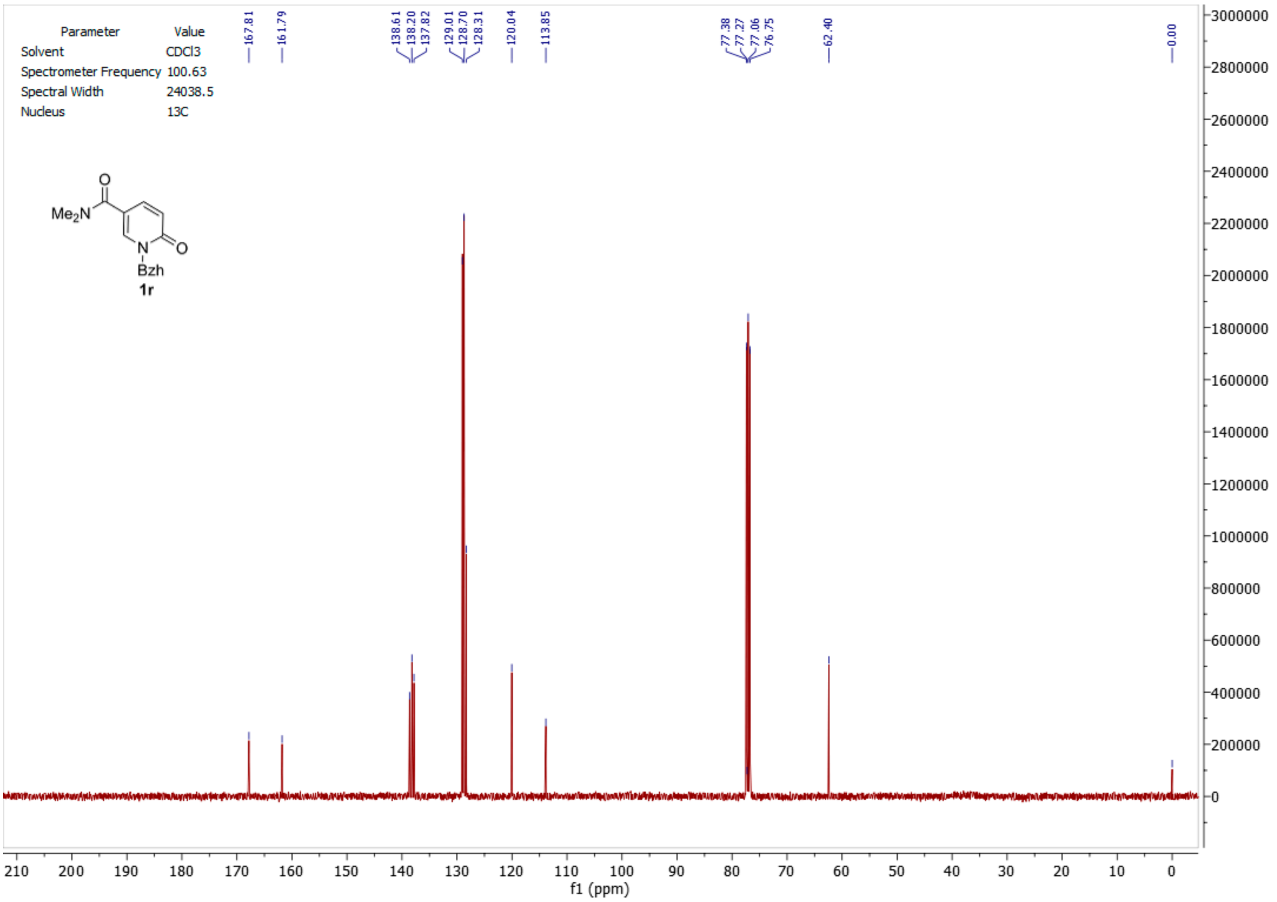
Parameter Value
Solvent CDCl3
Spectrometer Frequency 100.63
Spectral Width 24038.5
Nucleus 13C
Me2N
O
N
O
Bzh
1r
167.81
161.79
138.61
138.20
137.82
129.01
128.70
128.31
120.04
113.85
77.38
77.27
77.06
76.75
62.40
0.00
f1 (ppm)

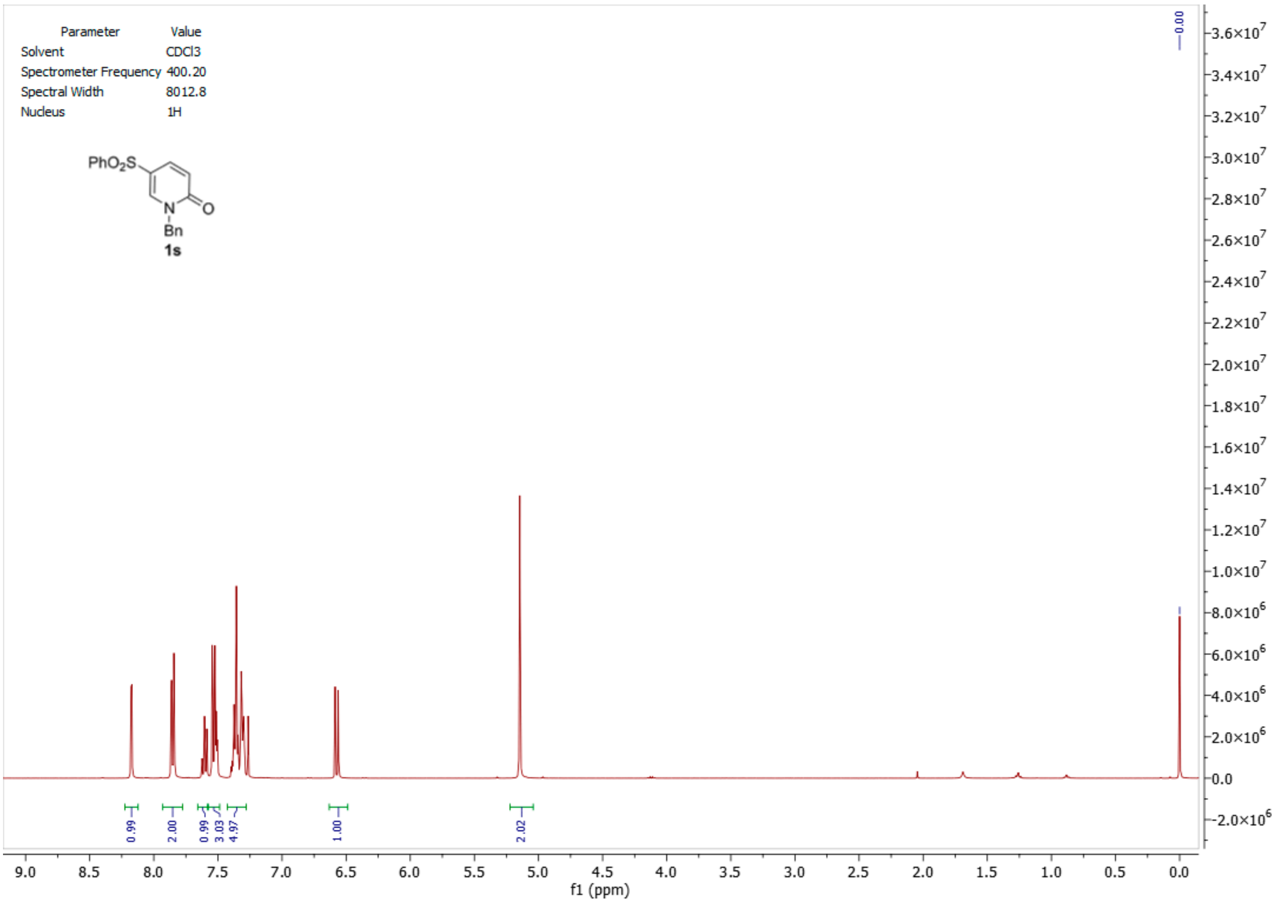

Parameter Value
Solvent CDCl3
Spectrometer Frequency 400.20
Spectral Width 8012.8
Nucleus 1H
PhO2S
N
O
Bn
1s
0.99
2.00
0.99
3.03
4.97
1.00
2.02
f1 (ppm)

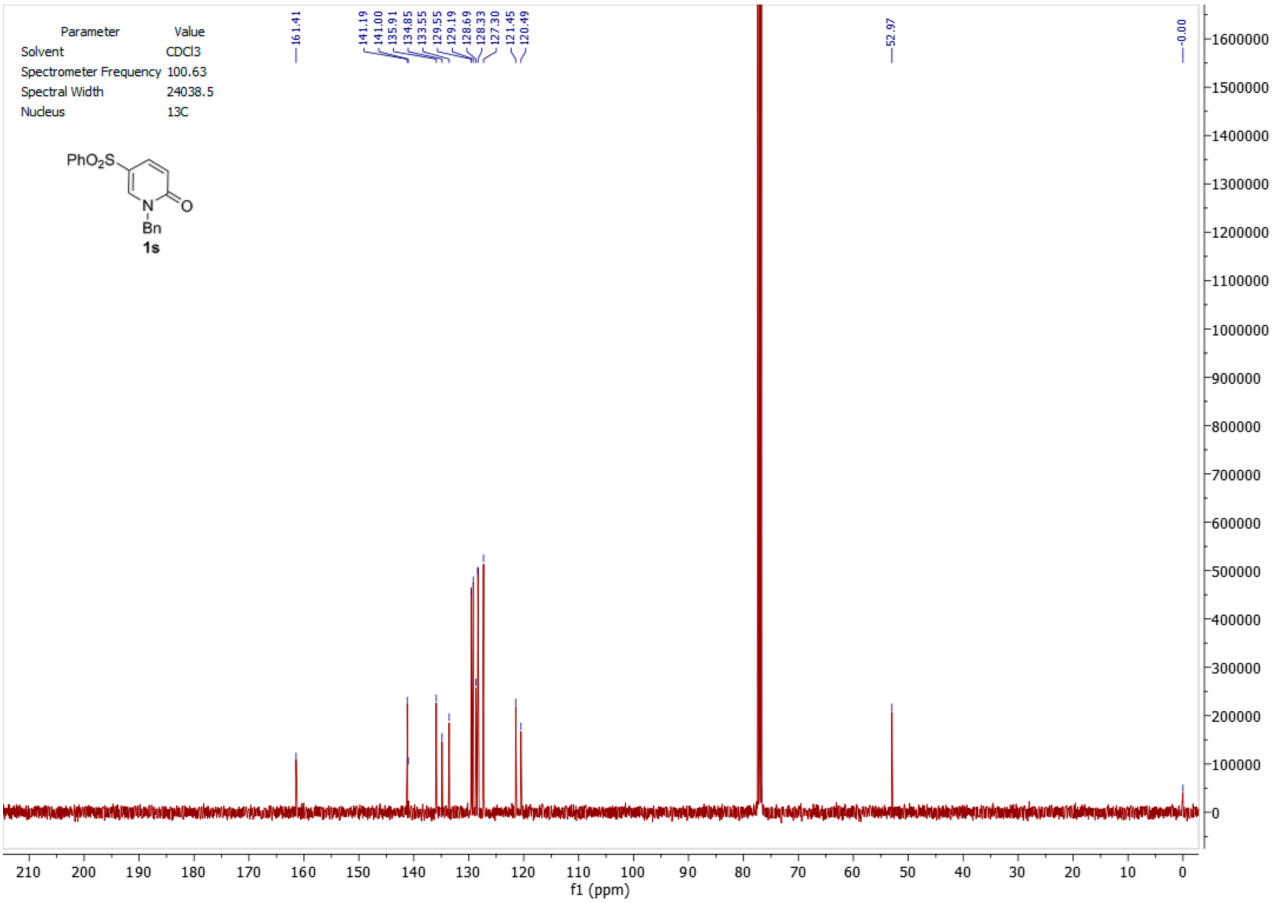

Parameter Value
Solvent CDCl3
Spectrometer Frequency 100.63
Spectral Width 24038.5
Nucleus 13C
PhO2S
N
O
Bn
1s
161.41
141.19
141.00
135.91
134.85
133.55
129.55
129.19
128.69
128.33
127.30
121.45
120.49
52.97
-0.00
f1 (ppm)

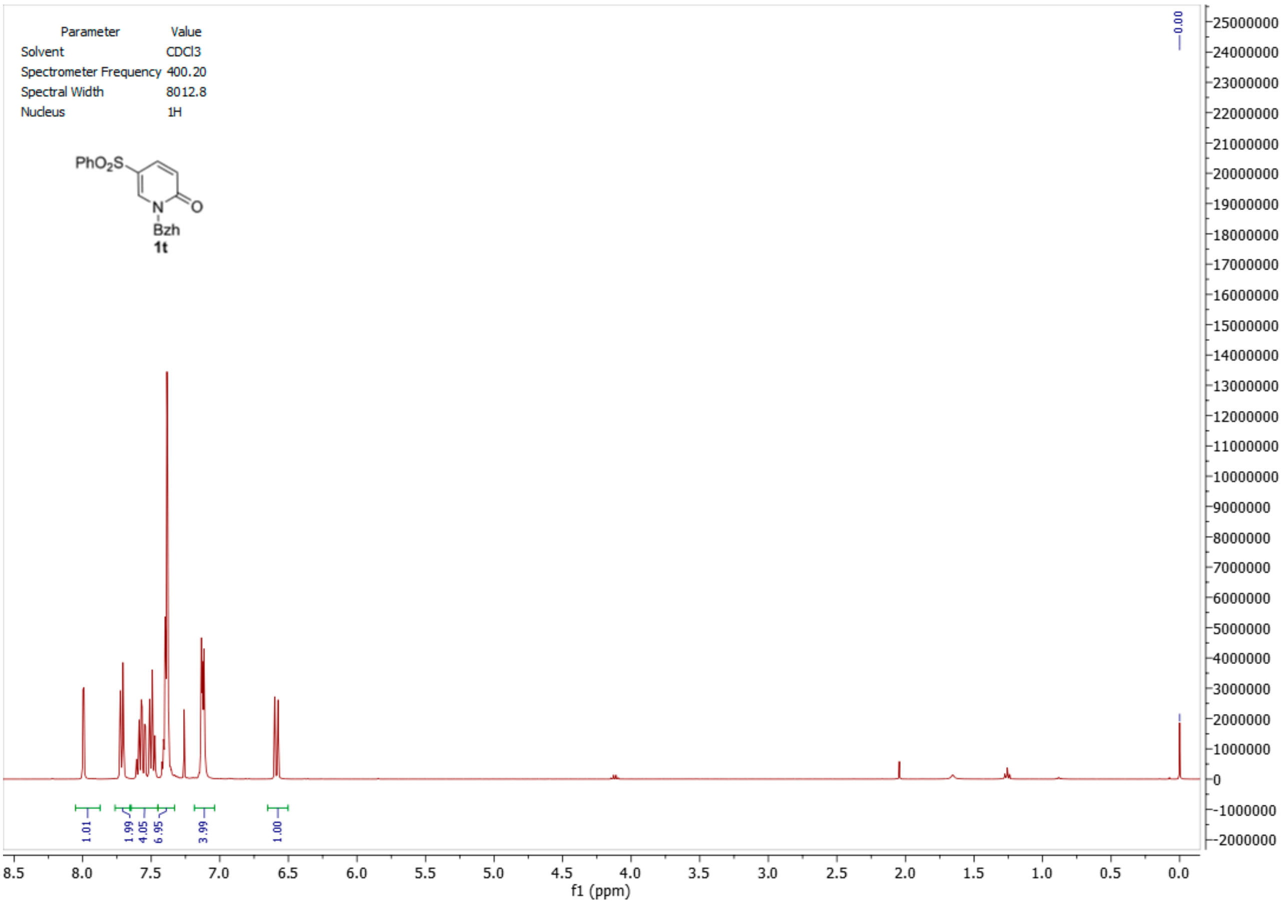

Parameter Value
Solvent CDCl3
Spectrometer Frequency 400.20
Spectral Width 8012.8
Nucleus 1H
PhO2S
N
O
Bzh
1t
f1 (ppm)

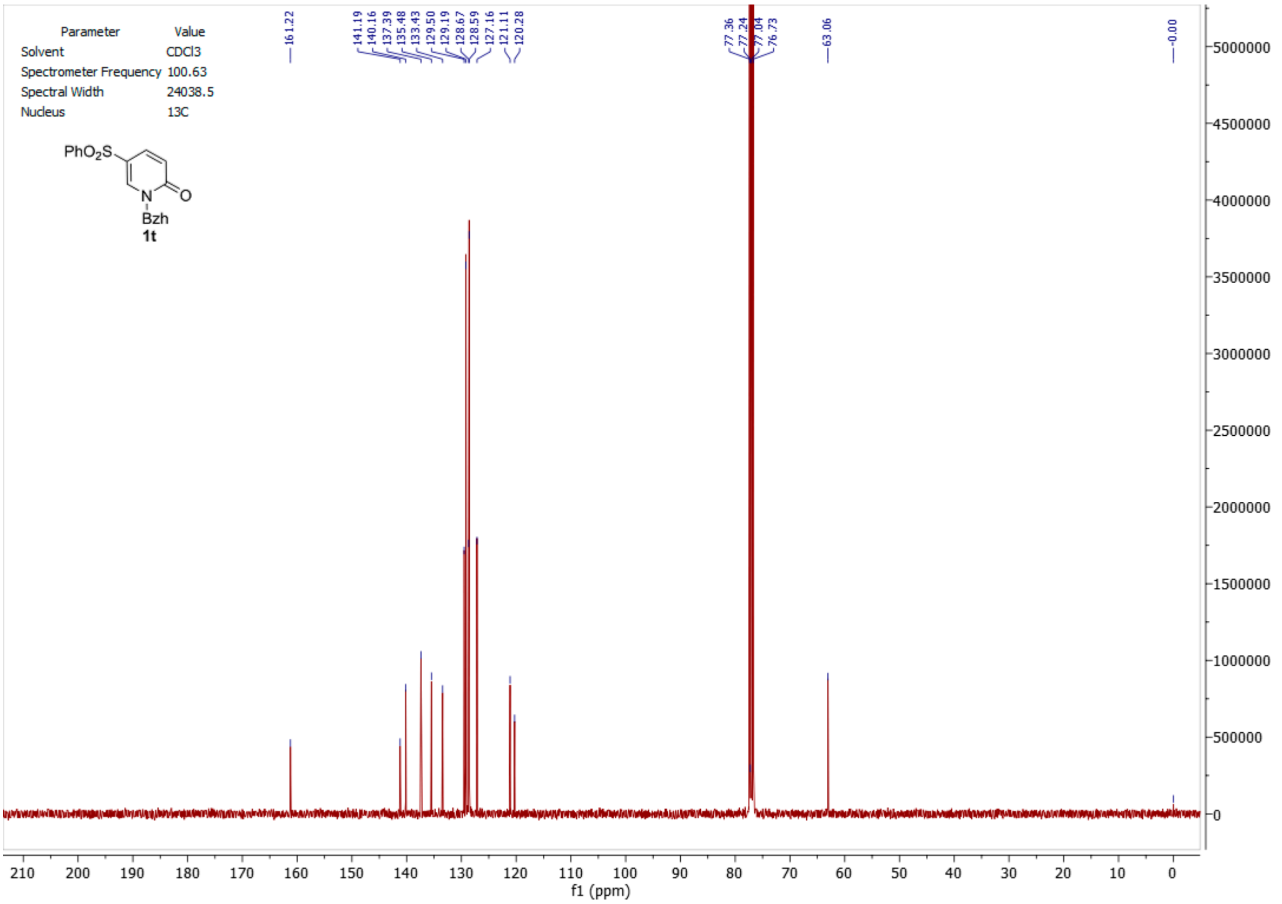

Parameter Value
Solvent CDCl3
Spectrometer Frequency 100.63
Spectral Width 24038.5
Nucleus 13C
PhO2S
N
O
Bzh
1t
161.22
141.19
140.16
137.39
135.48
133.43
129.50
129.19
128.67
128.59
127.16
121.11
120.28
77.36
77.24
77.04
76.73
63.06
-0.00
f1 (ppm)

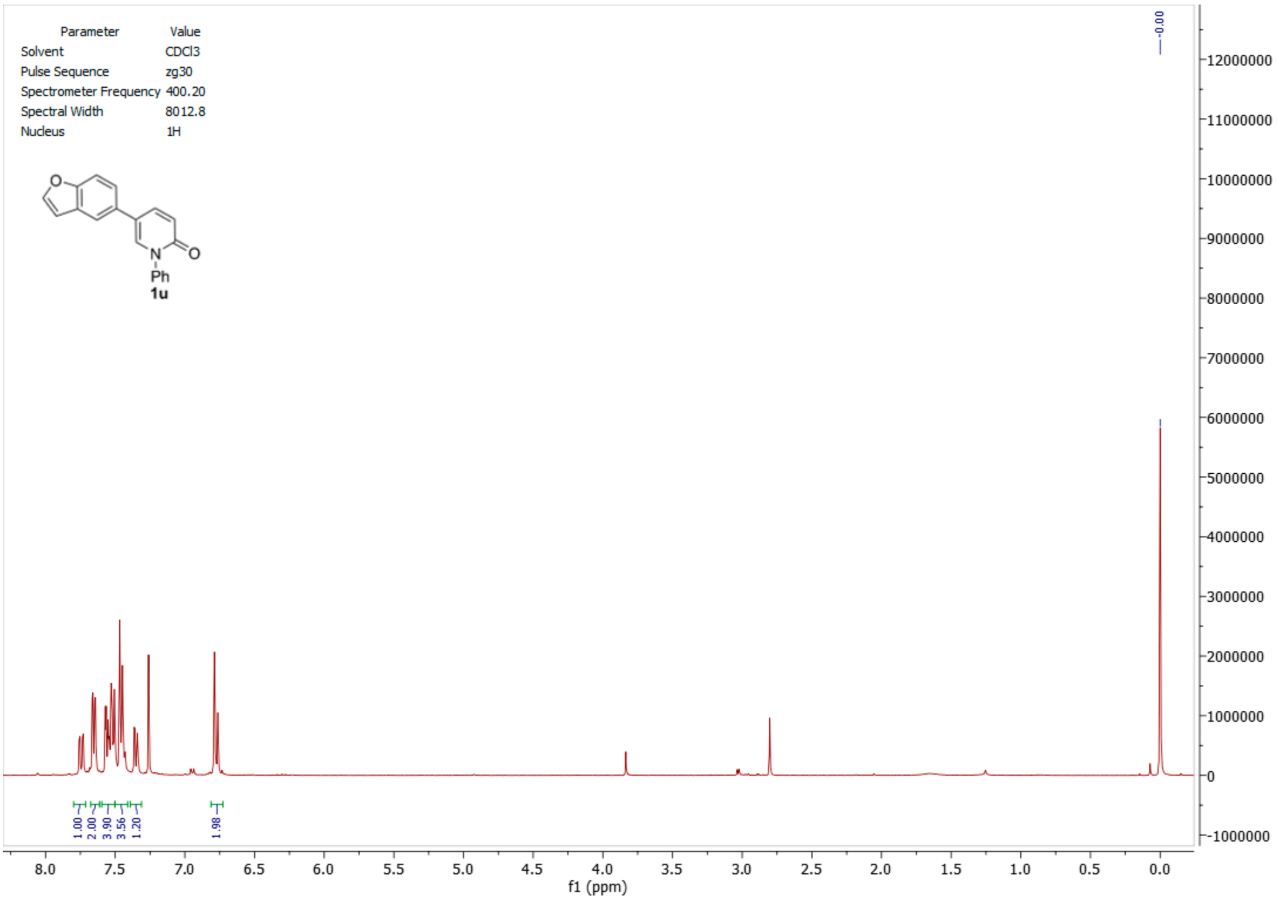
Parameter Value
Solvent CDCl3
Pulse Sequence zg30
Spectrometer Frequency 400.20
Spectral Width 8012.8
Nucleus 1H
1u
f1 (ppm)

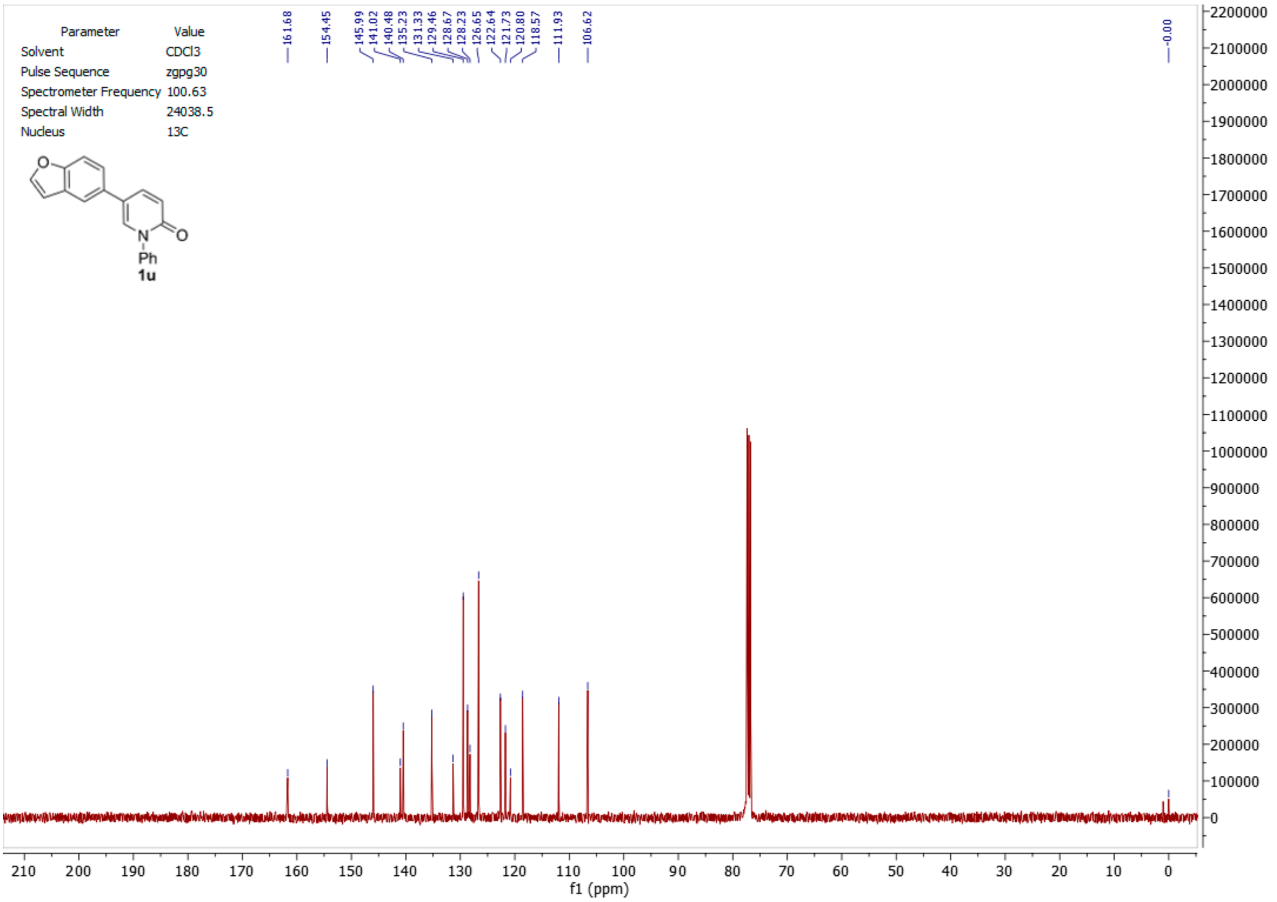
161.68
154.45
145.99
141.02
140.48
135.23
131.33
129.46
128.67
128.23
126.65
122.64
121.73
120.80
118.57
111.93
106.62
Parameter Value
Solvent CDCl3
Pulse Sequence zgpg30
Spectrometer Frequency 100.63
Spectral Width 24038.5
Nucleus 13C
1u
f1 (ppm)

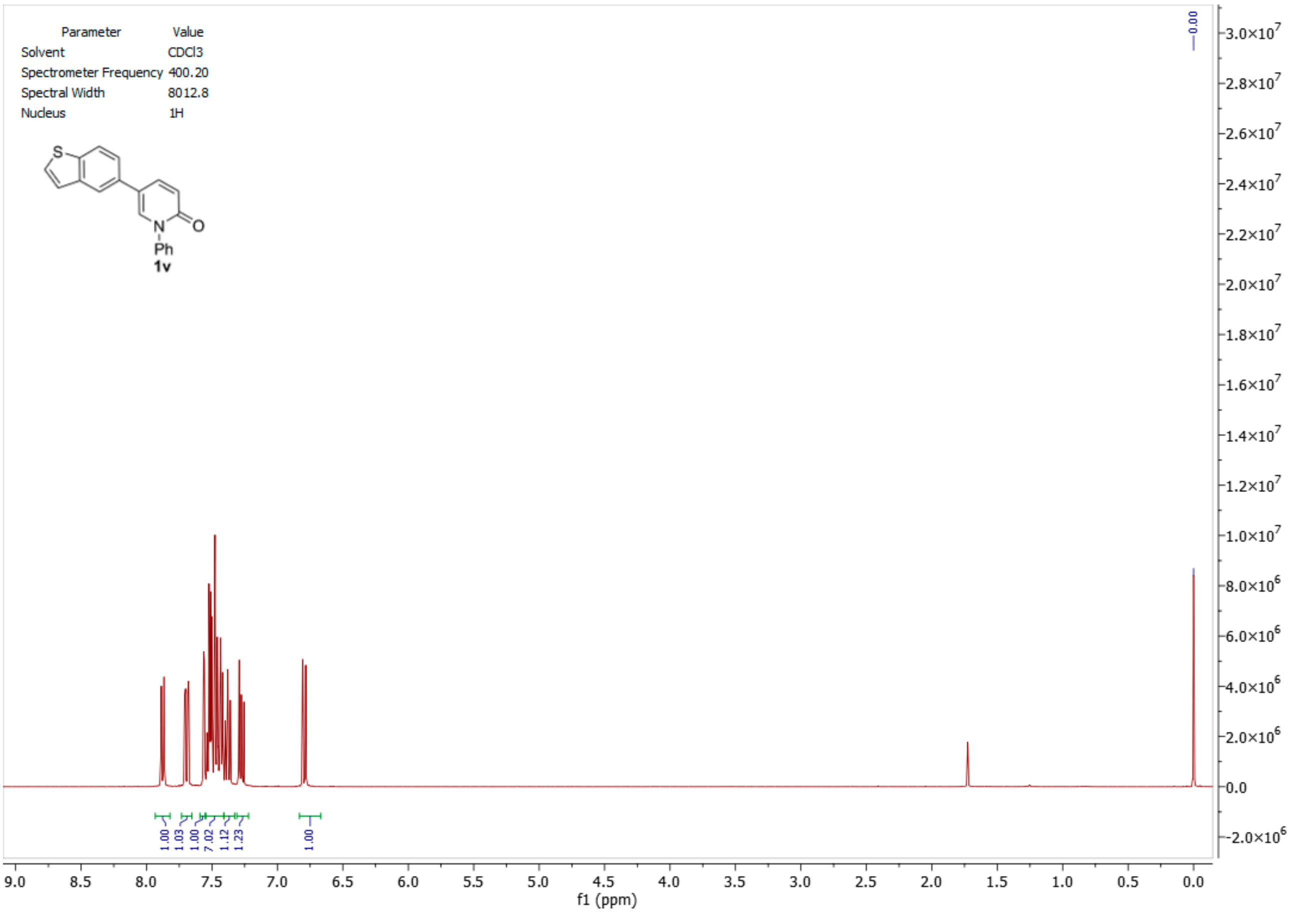
Parameter Value
Solvent CDCl3
Spectrometer Frequency 400.20
Spectral Width 8012.8
Nucleus 1H
S
N
O
Ph
1v
0.00
1.00 1.03 1.00 7.02 1.12 1.23 1.00
f1 (ppm)

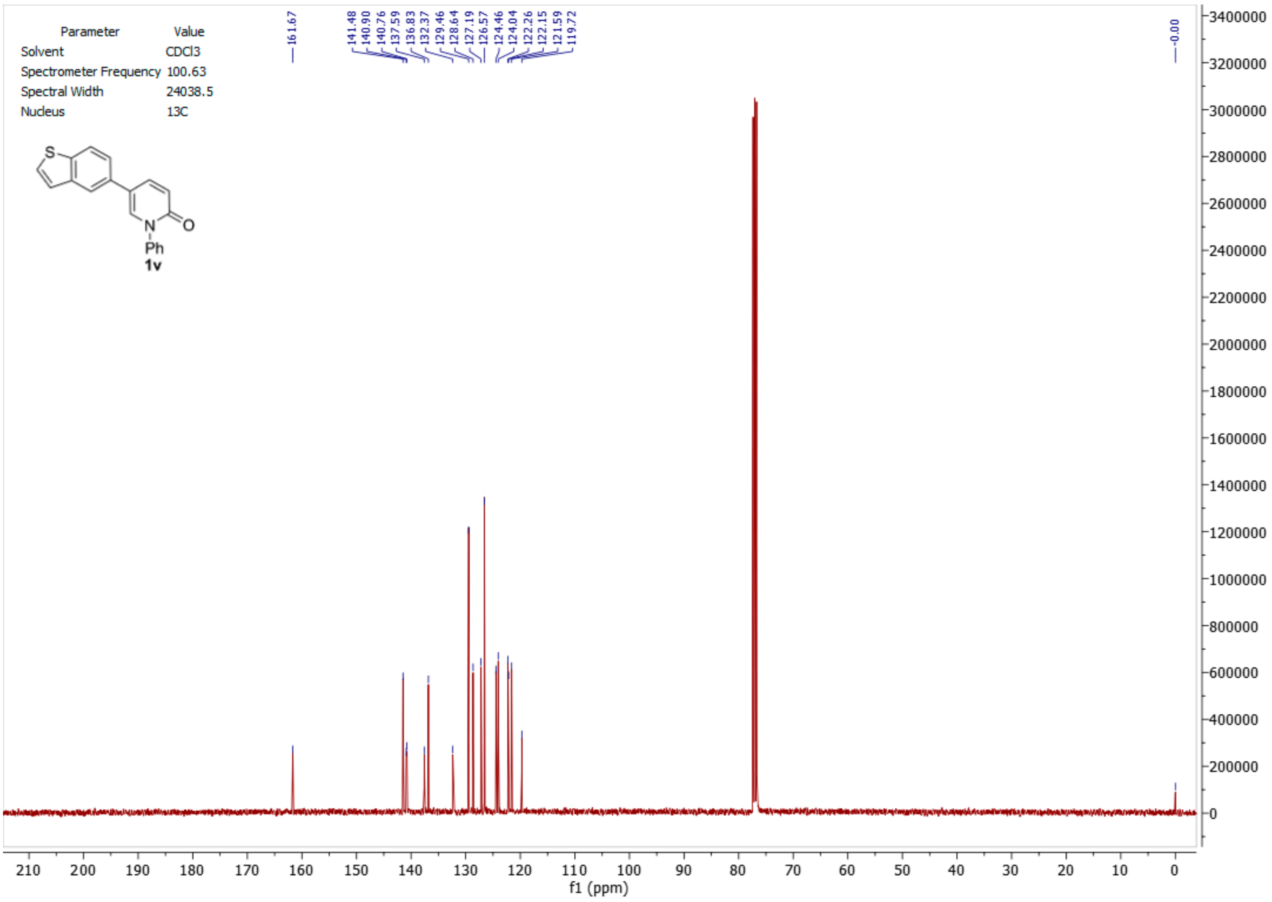
Parameter Value
Solvent CDCl3
Spectrometer Frequency 100.63
Spectral Width 24038.5
Nucleus 13C
S
N
O
Ph
1v
161.67
141.48 140.90 140.76 137.59 136.83 132.37 129.46 128.64 127.19 126.57 124.46 124.04 122.26 122.15 121.59 119.72
0.00
f1 (ppm)

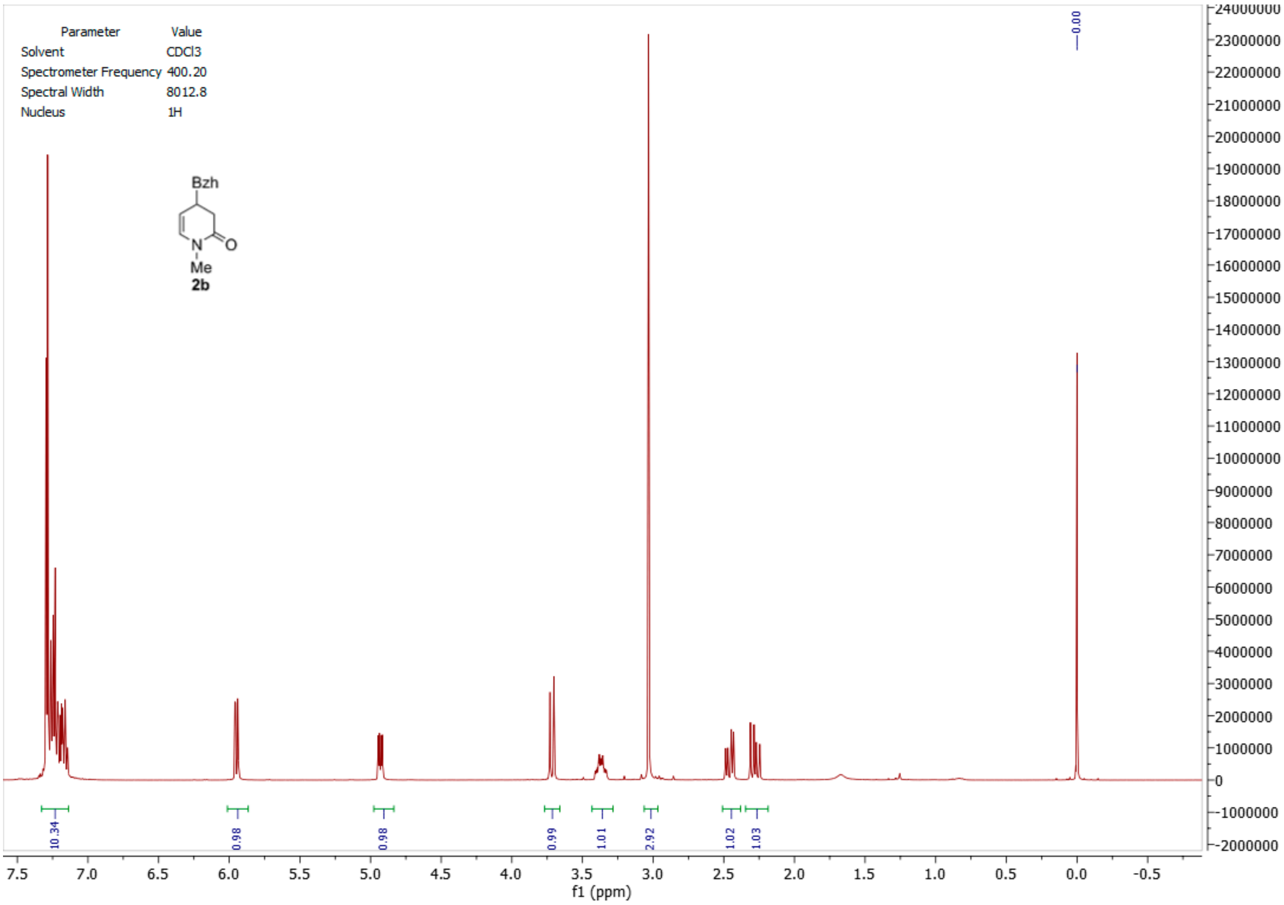

Parameter Value
Solvent CDCl3
Spectrometer Frequency 400.20
Spectral Width 8012.8
Nucleus 1H
Bzh
N
O
Me
2b
0.00
10.34
0.98
0.98
0.99
1.01
2.92
1.02
1.03
f1 (ppm)

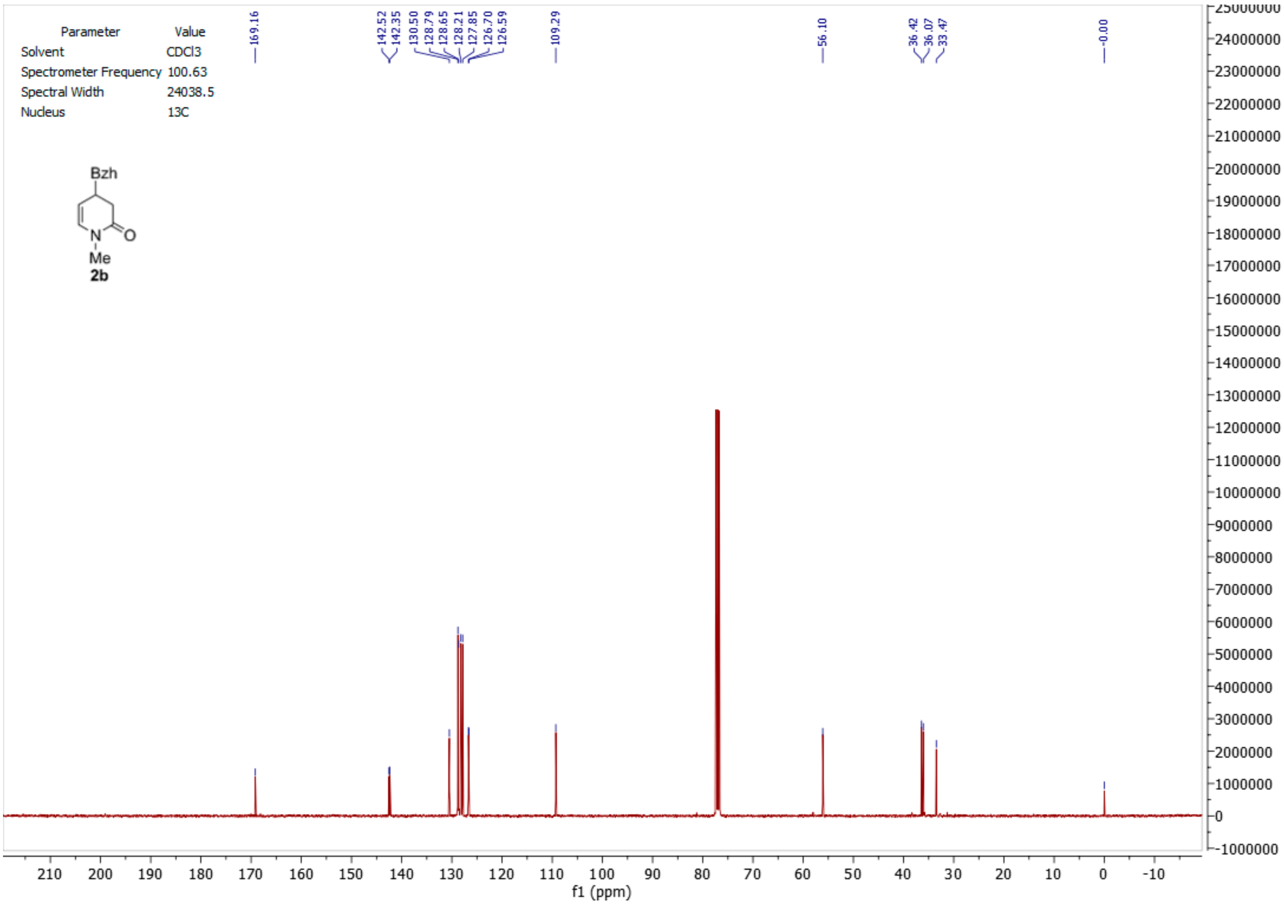

Parameter Value
Solvent CDCl3
Spectrometer Frequency 100.63
Spectral Width 24038.5
Nucleus 13C
Bzh
N
O
Me
2b
169.16
142.52
142.35
130.50
128.79
128.65
128.21
127.85
126.70
126.59
109.29
56.10
36.42
36.07
33.47
0.00
f1 (ppm)

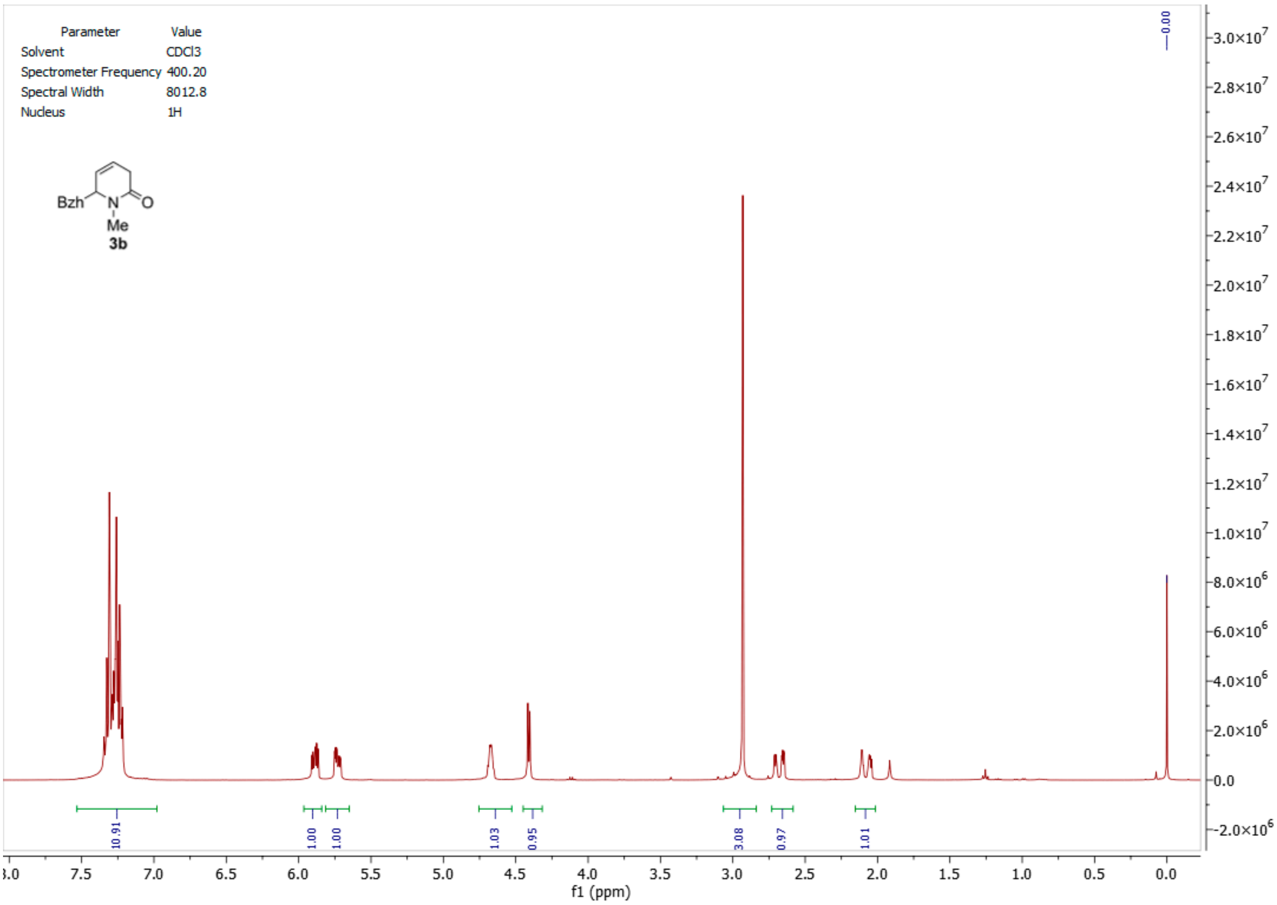
Parameter Value
Solvent CDCl3
Spectrometer Frequency 400.20
Spectral Width 8012.8
Nucleus 1H
Bzh
N
O
Me
3b
0.00
10.91
1.00
1.00
1.03
0.95
3.08
0.97
1.01
f1 (ppm)

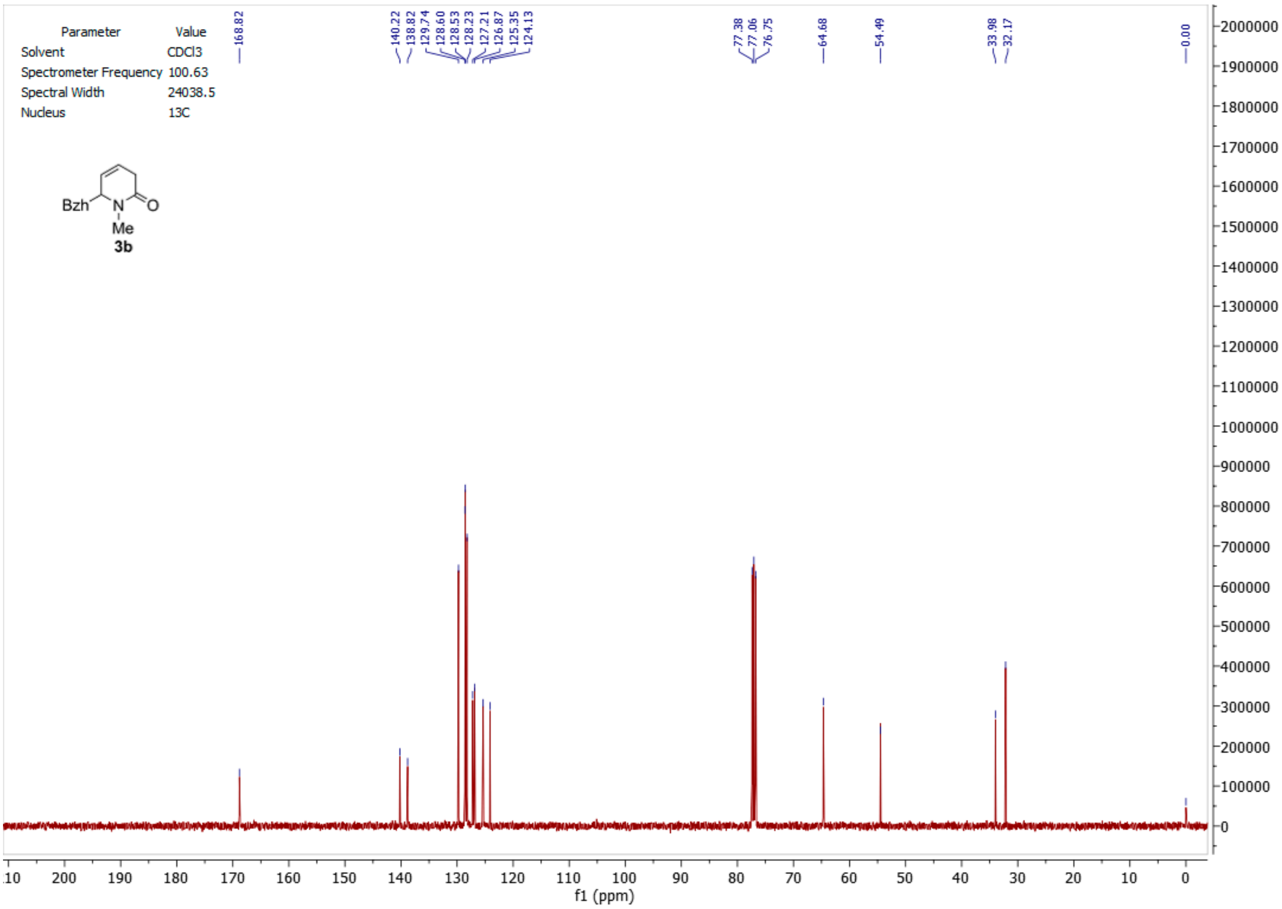
Parameter Value
Solvent CDCl3
Spectrometer Frequency 100.63
Spectral Width 24038.5
Nucleus 13C
Bzh
N
O
Me
3b
168.82
140.22
138.82
129.74
128.60
128.53
128.23
127.21
126.87
125.35
124.13
77.38
77.06
76.75
64.68
54.49
33.98
32.17
0.00
f1 (ppm)

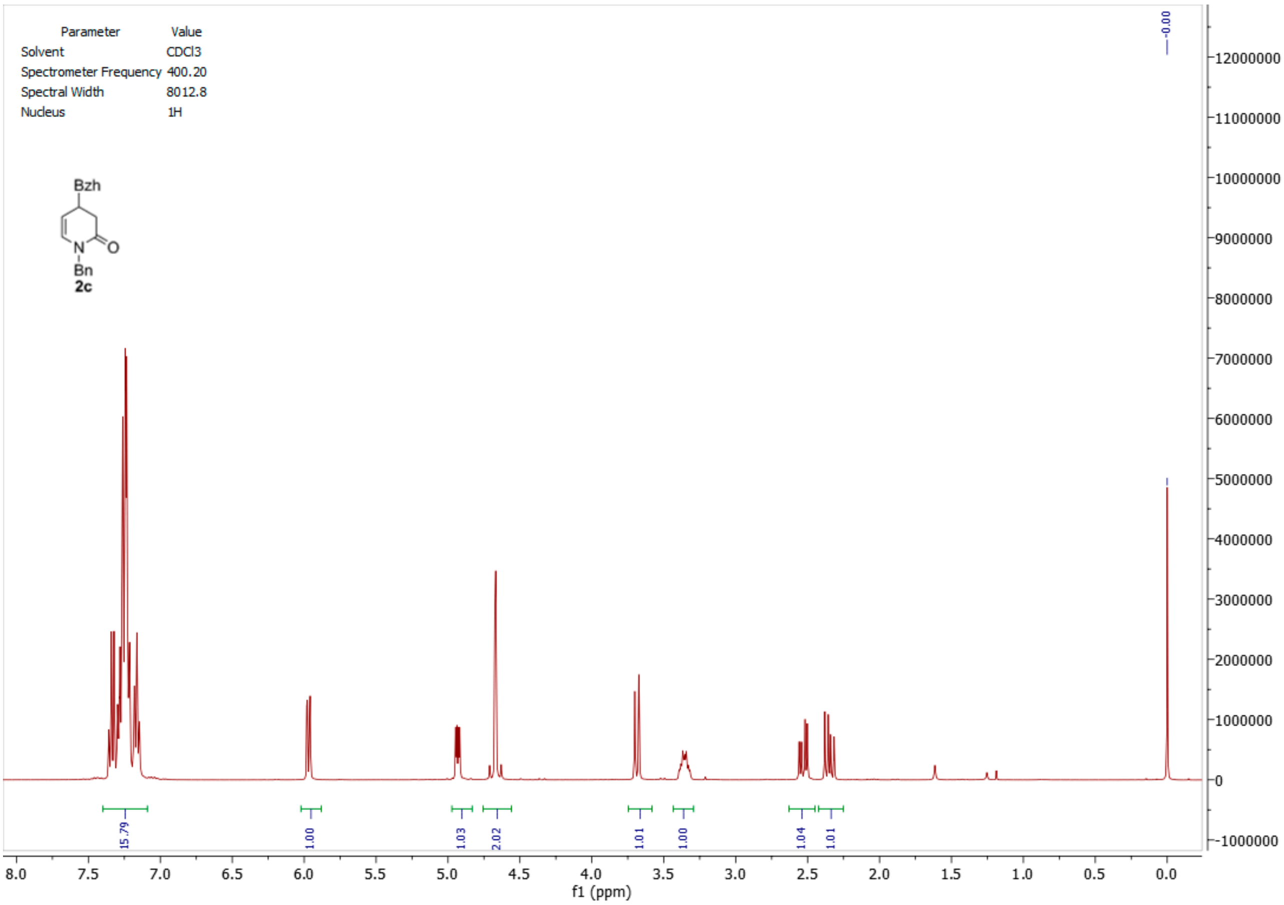
Parameter Value
Solvent CDCl3
Spectrometer Frequency 400.20
Spectral Width 8012.8
Nucleus 1H
Bzh
N
O
Bn
2c
15.79
1.00
1.03
2.02
1.01
1.00
1.04
1.01
f1 (ppm)

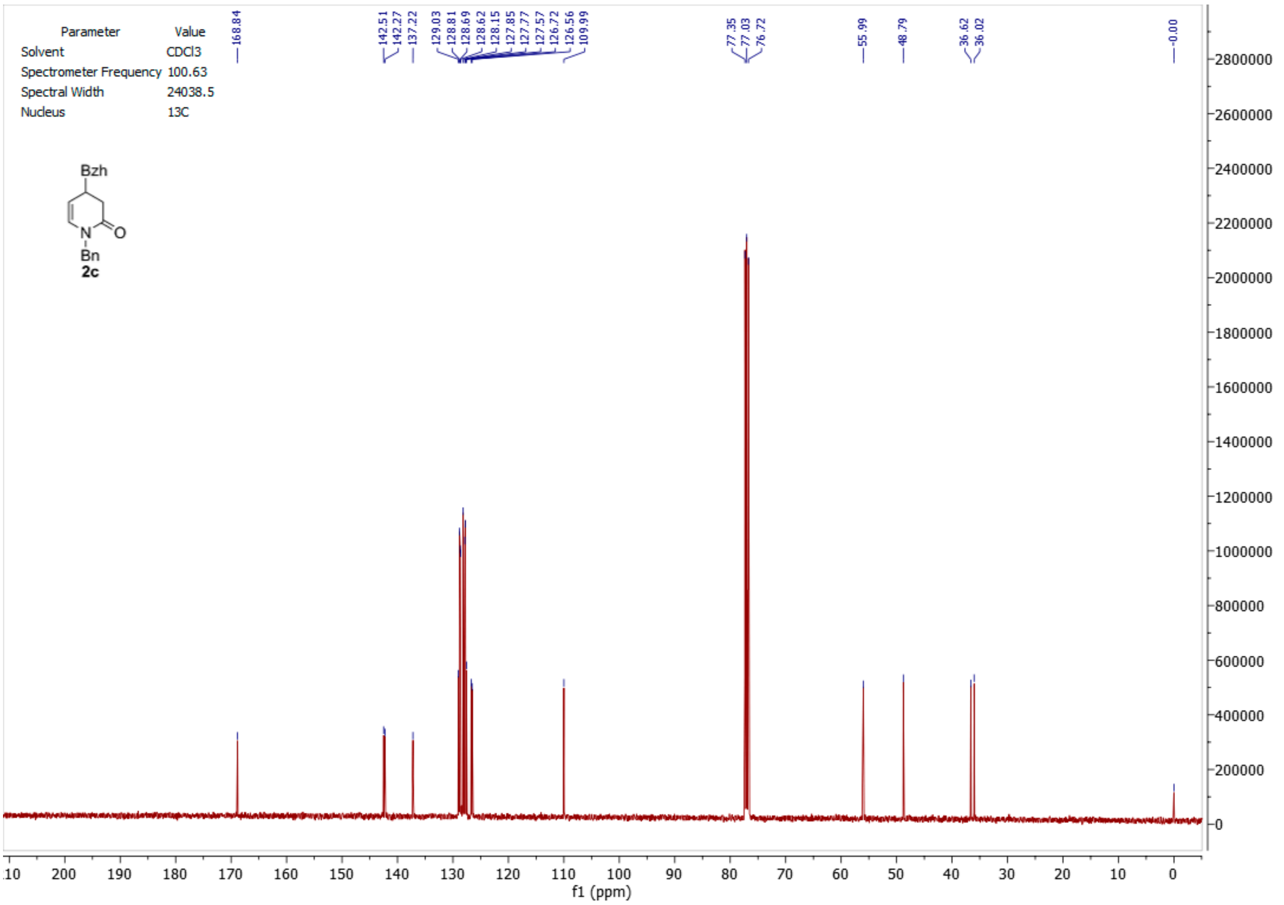
Parameter Value
Solvent CDCl3
Spectrometer Frequency 100.63
Spectral Width 24038.5
Nucleus 13C
Bzh
N
O
Bn
2c
168.84
142.51
142.27
137.22
129.03
128.81
128.69
128.62
128.15
127.85
127.77
127.57
126.72
126.56
109.99
77.35
77.03
76.72
55.99
48.79
36.62
36.02
f1 (ppm)

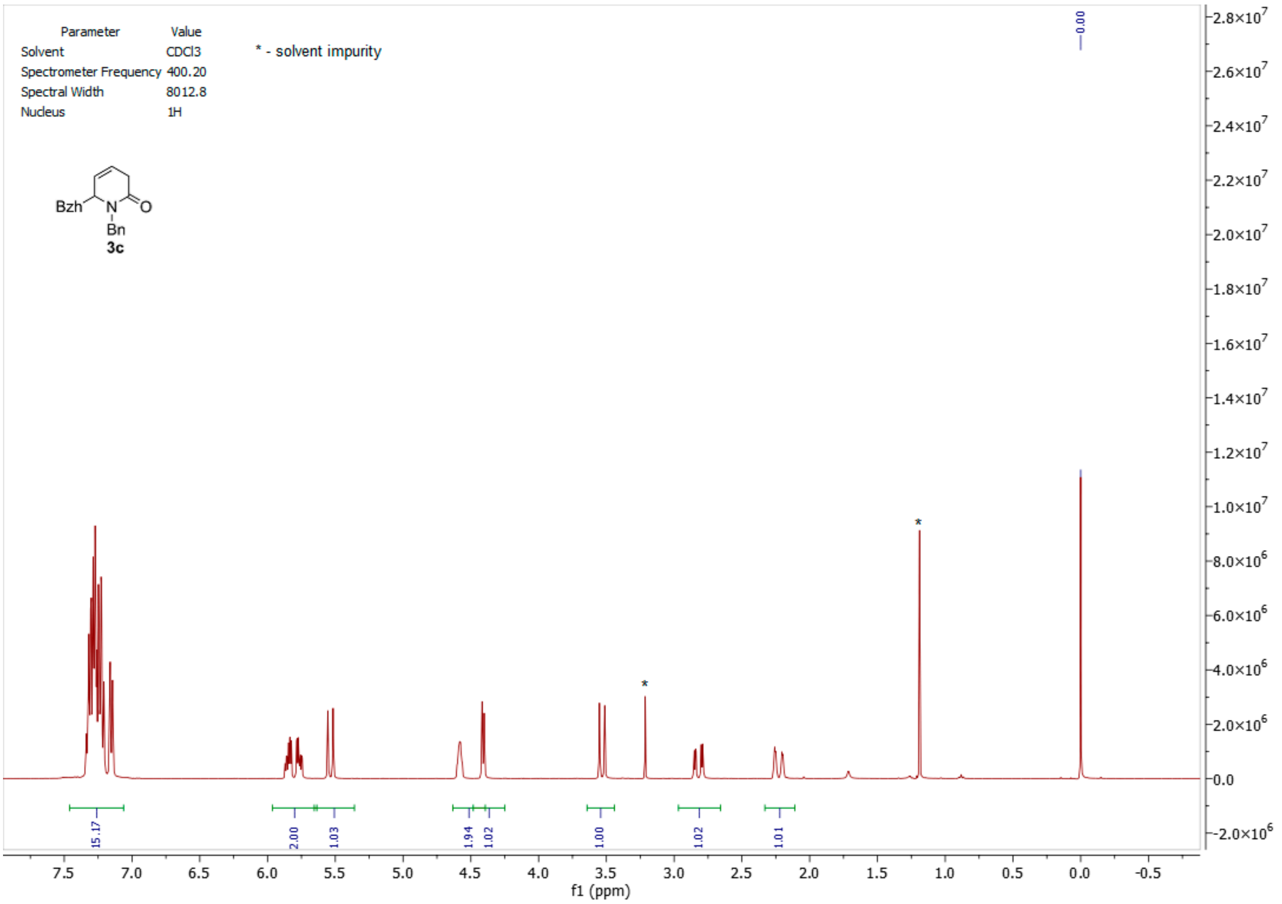
Parameter Value
Solvent CDCl3
Spectrometer Frequency 400.20
Spectral Width 8012.8
Nucleus 1H
* - solvent impurity
Bzh
N
O
Bn
3c
f1 (ppm)

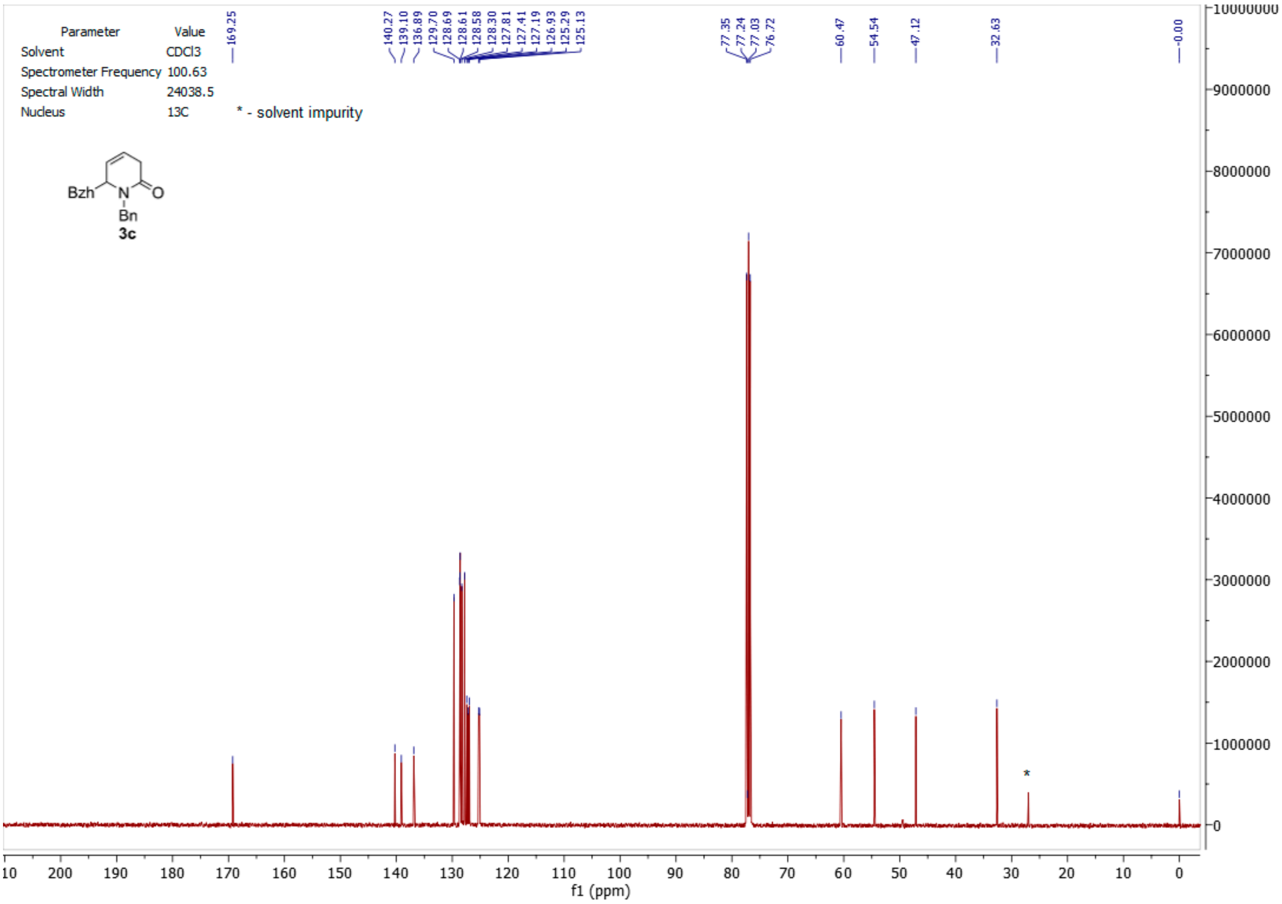
Parameter Value
Solvent CDCl3
Spectrometer Frequency 100.63
Spectral Width 24038.5
Nucleus 13C
* - solvent impurity
Bzh
N
O
Bn
3c
f1 (ppm)

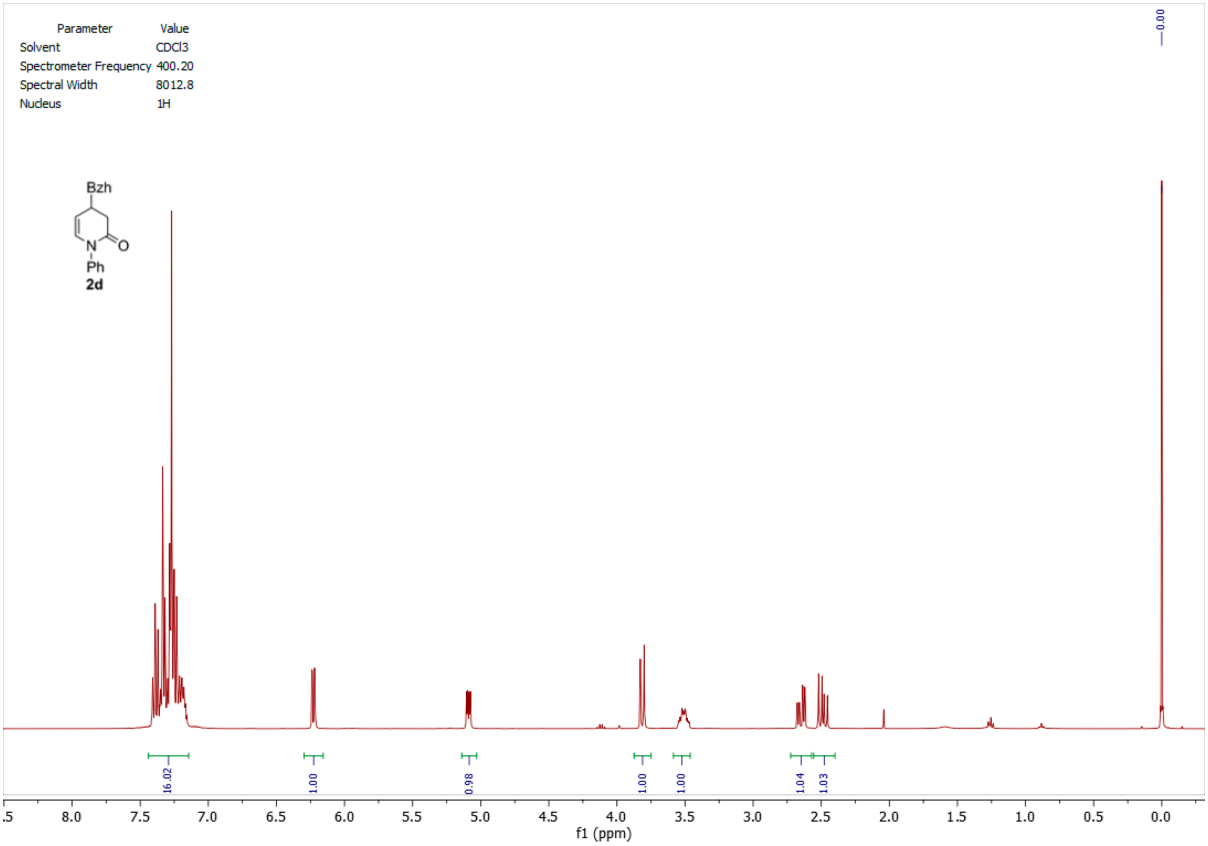

Parameter Value
Solvent CDCl3
Spectrometer Frequency 400.20
Spectral Width 8012.8
Nucleus 1H
Bzh
N
O
Ph
2d
16.02
1.00
0.98
1.00
1.00
1.04
1.03
0.00
f1 (ppm)

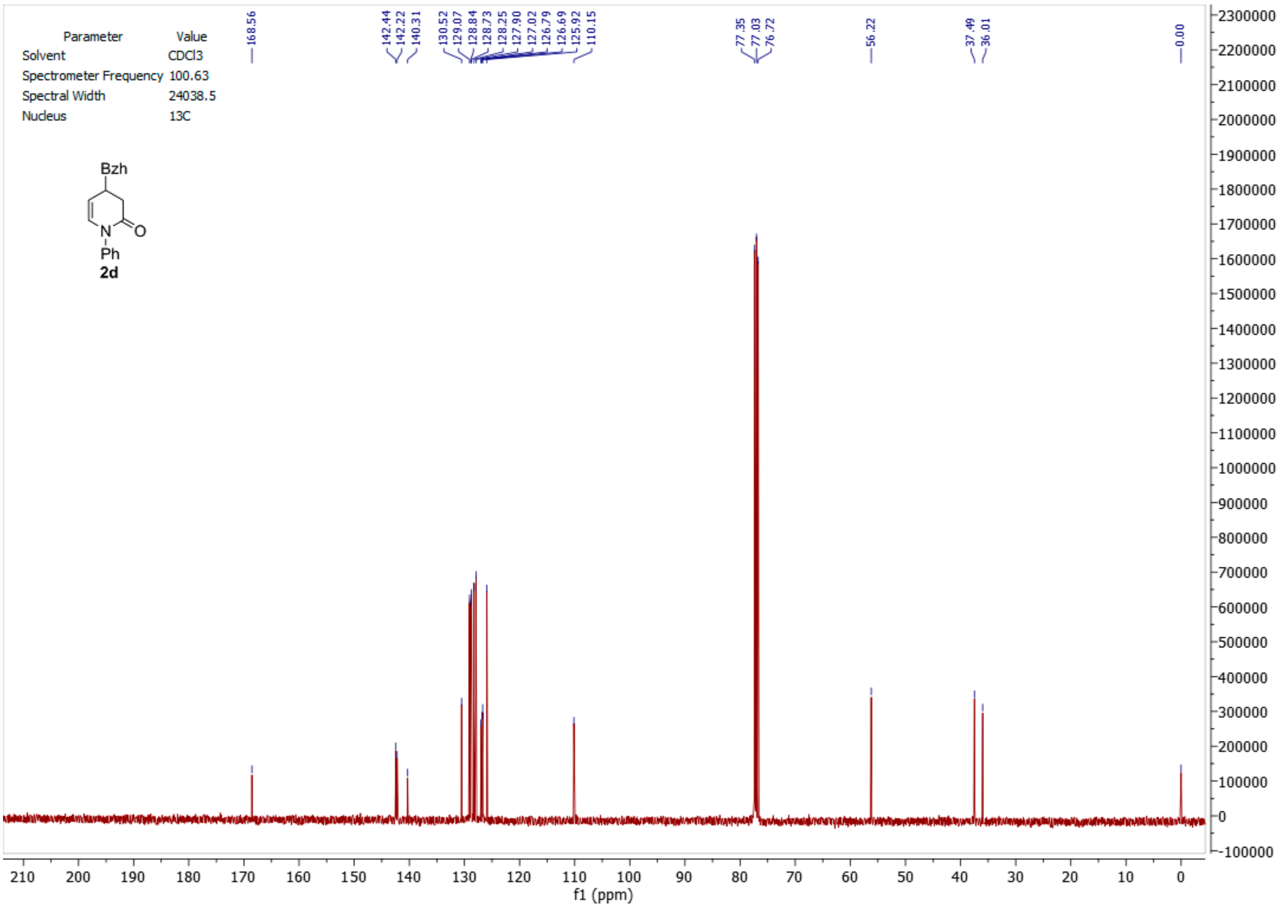

Parameter Value
Solvent CDCl3
Spectrometer Frequency 100.63
Spectral Width 24038.5
Nucleus 13C
Bzh
N
O
Ph
2d
168.56
142.44
142.22
140.31
130.52
129.07
128.84
128.73
128.25
127.90
127.02
126.79
126.69
125.92
110.15
77.35
77.03
76.72
56.22
37.49
36.01
0.00
f1 (ppm)

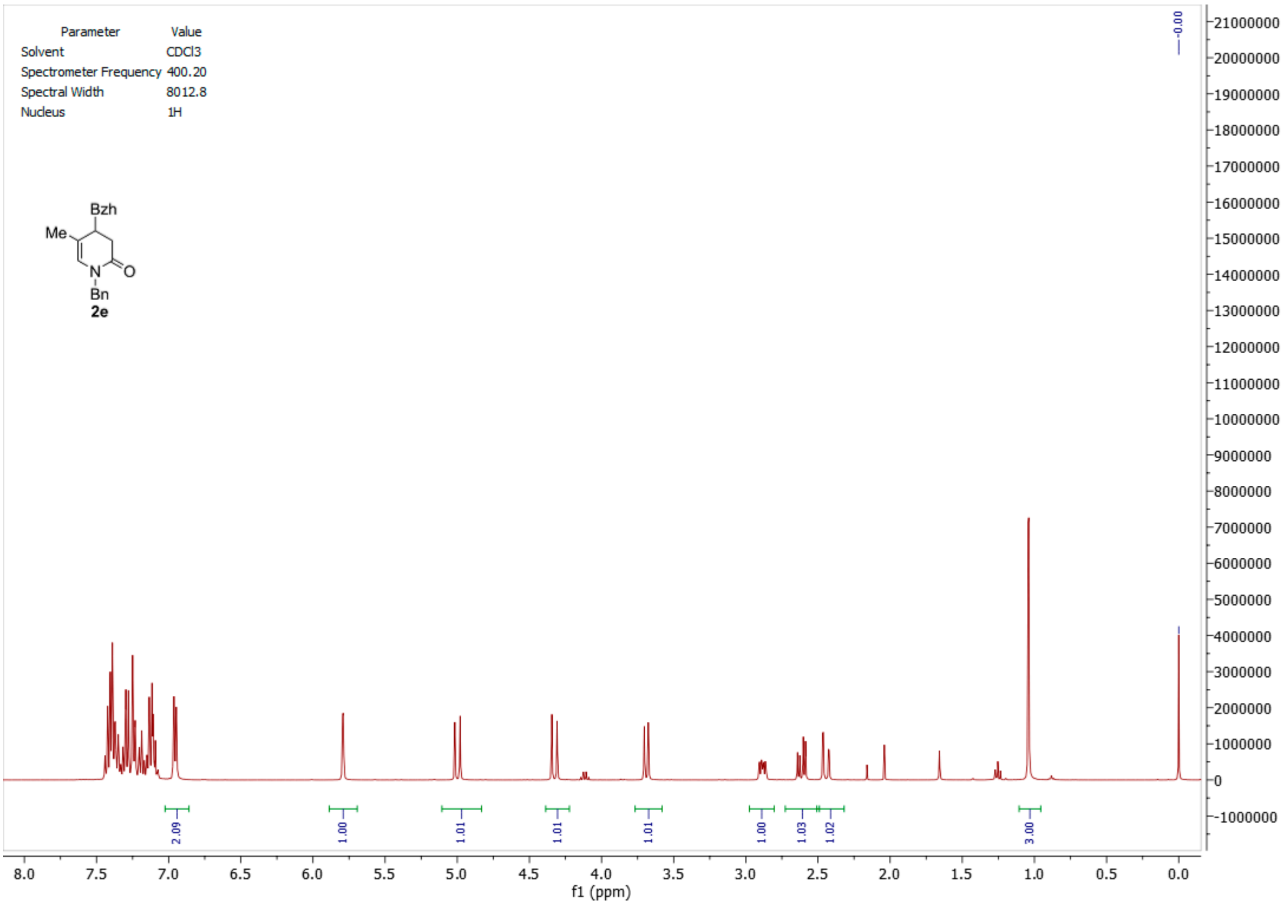
Parameter Value
Solvent CDCl3
Spectrometer Frequency 400.20
Spectral Width 8012.8
Nucleus 1H
Bzh
Me
N
O
Bn
2e
0.00
2.09
1.00
1.01
1.01
1.01
1.00
1.03
1.02
3.00
f1 (ppm)

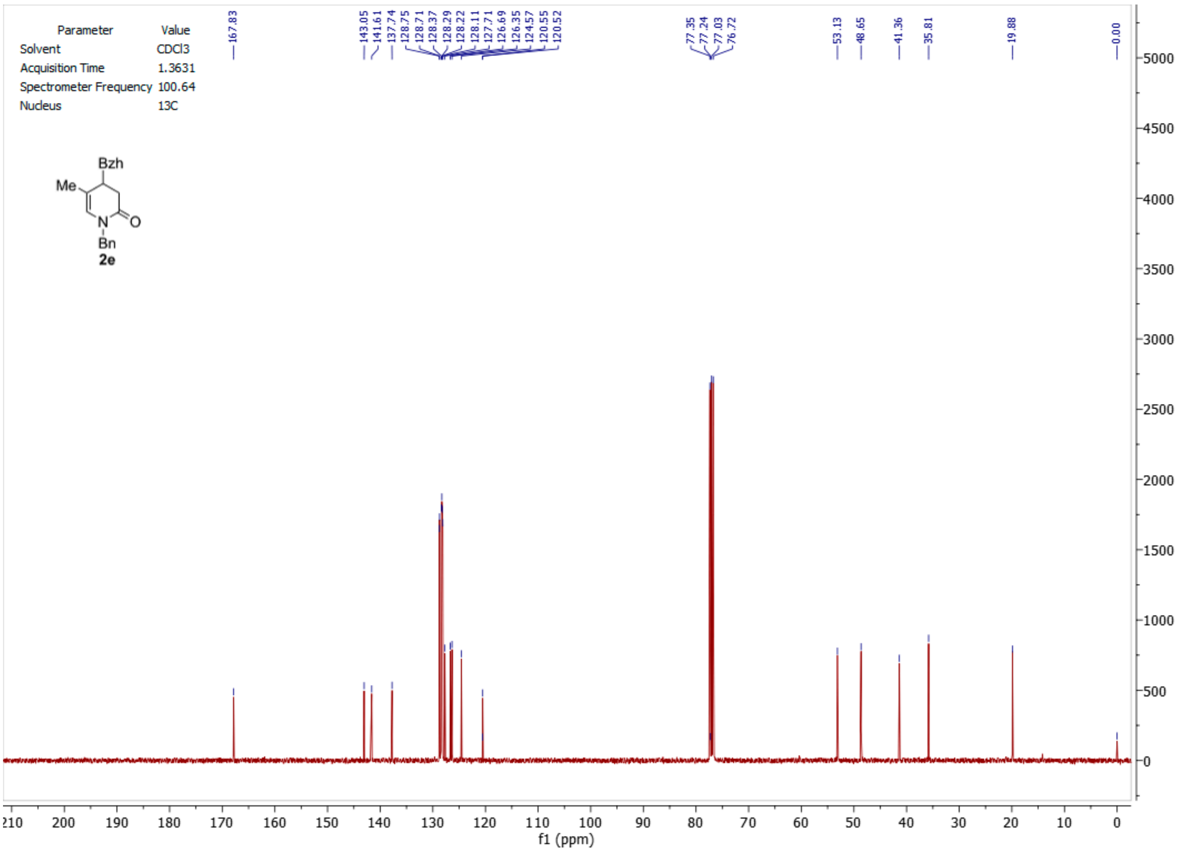
Parameter Value
Solvent CDCl3
Acquisition Time 1.3631
Spectrometer Frequency 100.64
Nucleus 13C
Bzh
Me
N
O
Bn
2e
167.83
143.05
141.61
137.74
128.75
128.71
128.37
128.29
128.22
128.11
127.71
126.69
126.35
124.57
120.55
120.52
77.35
77.24
77.03
76.72
53.13
48.65
41.36
35.81
19.88
0.00
f1 (ppm)

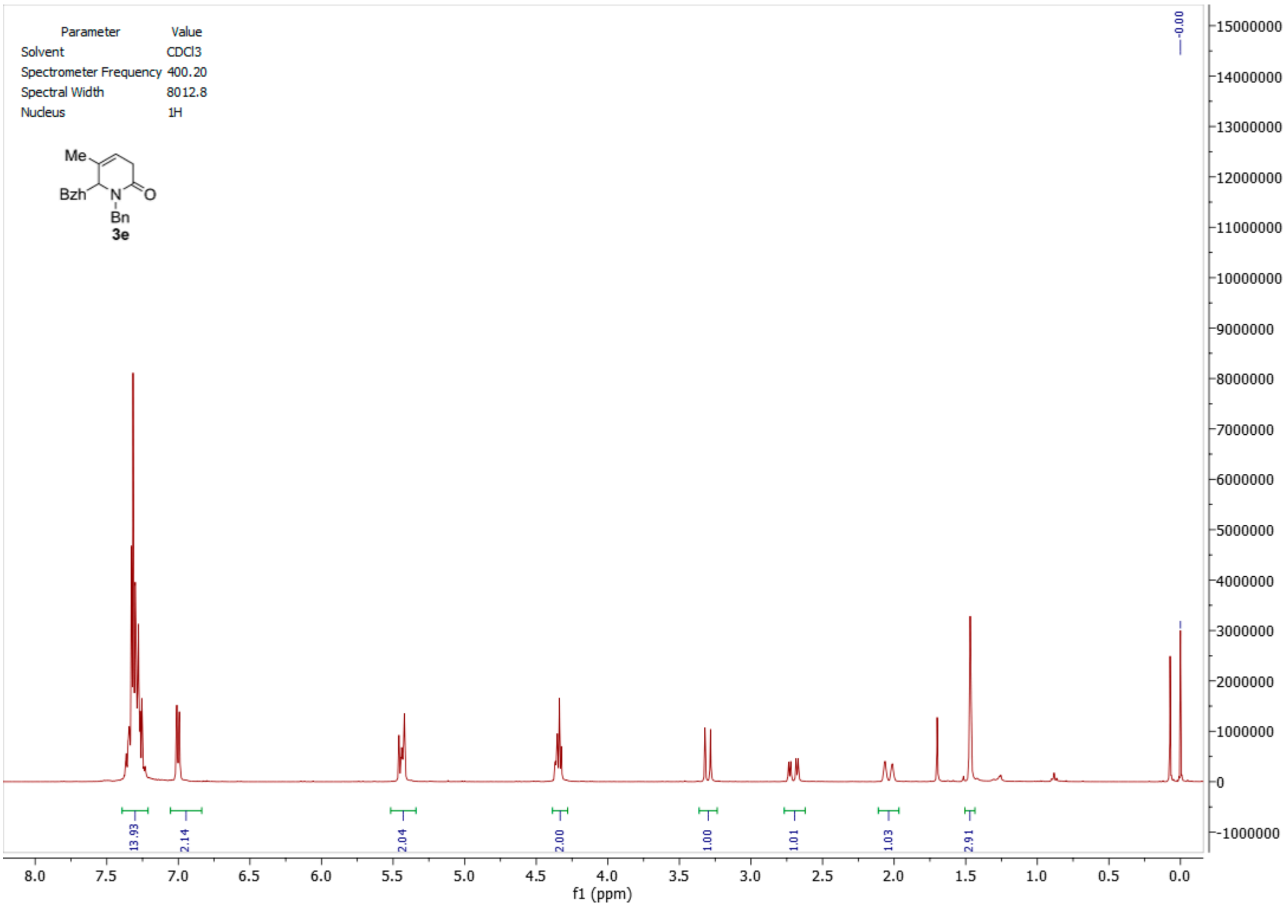
Parameter Value
Solvent CDCl3
Spectrometer Frequency 400.20
Spectral Width 8012.8
Nucleus 1H
Me
Bzh
N
O
Bn
3e
13.93
2.14
2.04
2.00
1.00
1.01
1.03
2.91
-0.00
f1 (ppm)

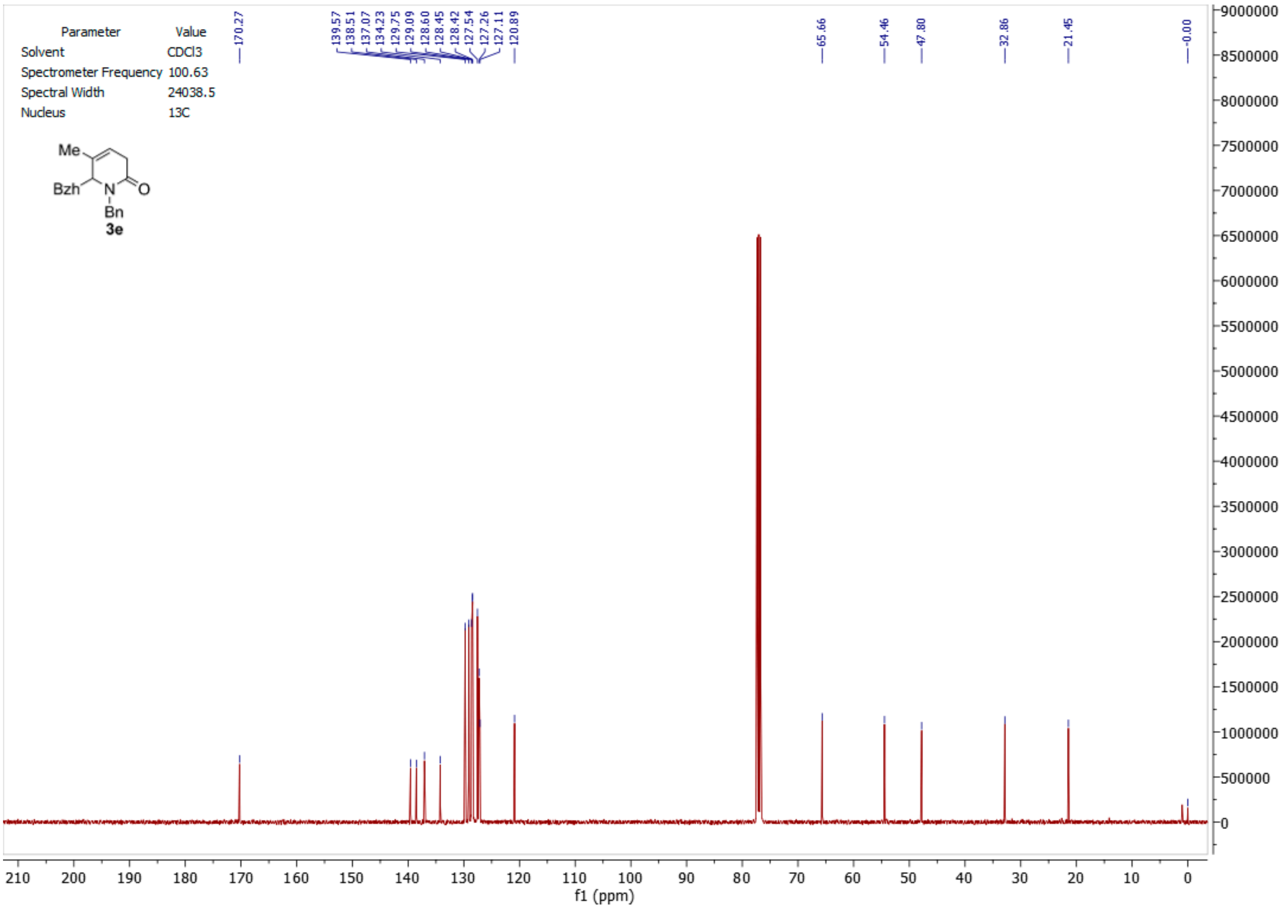
Parameter Value
Solvent CDCl3
Spectrometer Frequency 100.63
Spectral Width 24038.5
Nucleus 13C
Me
Bzh
N
O
Bn
3e
170.27
139.57
138.51
137.07
134.23
129.75
129.09
128.60
128.45
128.42
127.54
127.26
127.11
120.89
65.66
54.46
47.80
32.86
21.45
-0.00
f1 (ppm)

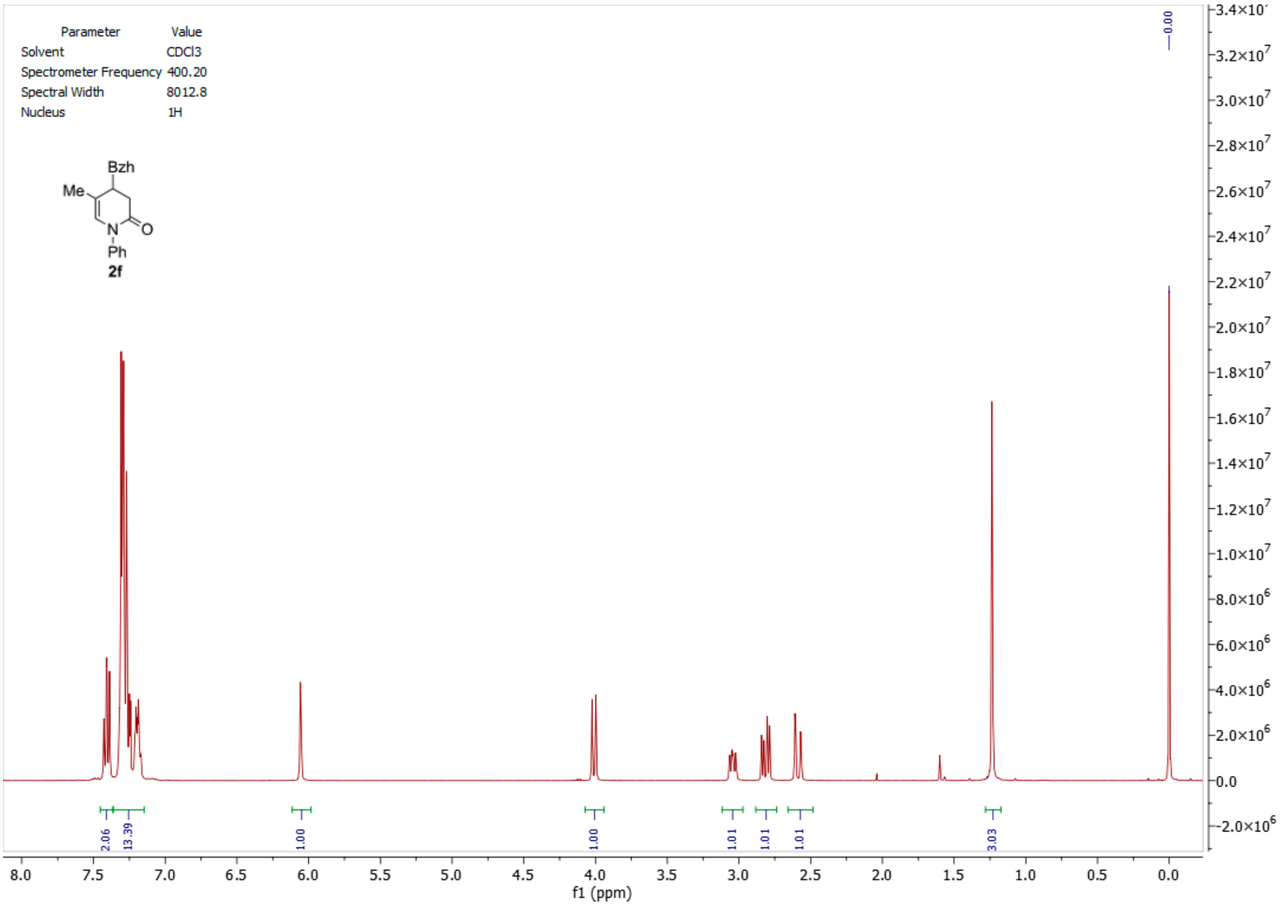
Parameter Value
Solvent CDCl3
Spectrometer Frequency 400.20
Spectral Width 8012.8
Nucleus 1H
Bzh
Me
N
O
Ph
2f
0.00
2.06
13.39
1.00
1.00
1.01
1.01
1.01
3.03
f1 (ppm)

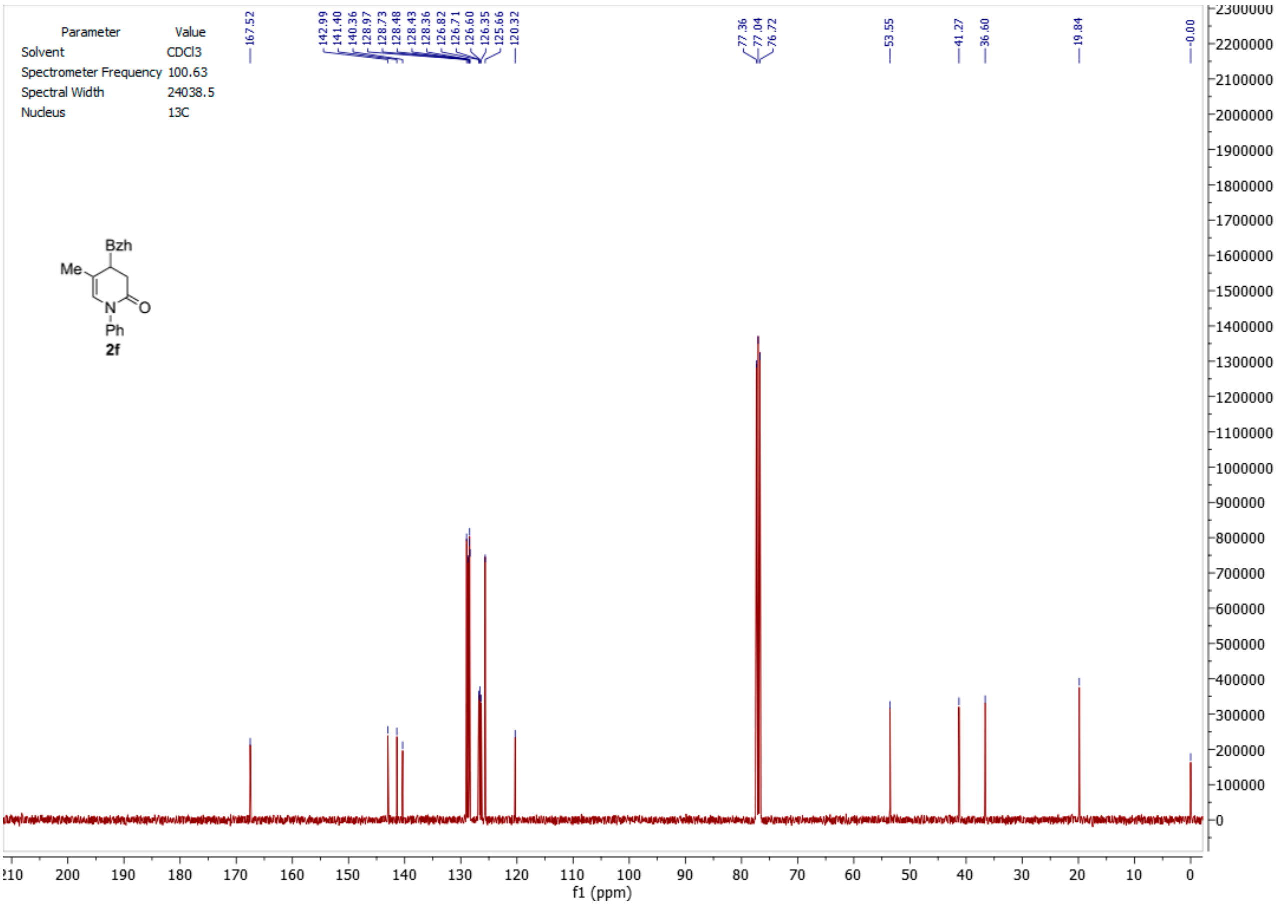
Parameter Value
Solvent CDCl3
Spectrometer Frequency 100.63
Spectral Width 24038.5
Nucleus 13C
Bzh
Me
N
O
Ph
2f
167.52
142.99
141.40
140.36
128.97
128.73
128.48
128.43
128.36
126.82
126.71
126.60
126.35
125.66
120.32
77.36
77.04
76.72
53.55
41.27
36.60
19.84
-0.00
f1 (ppm)

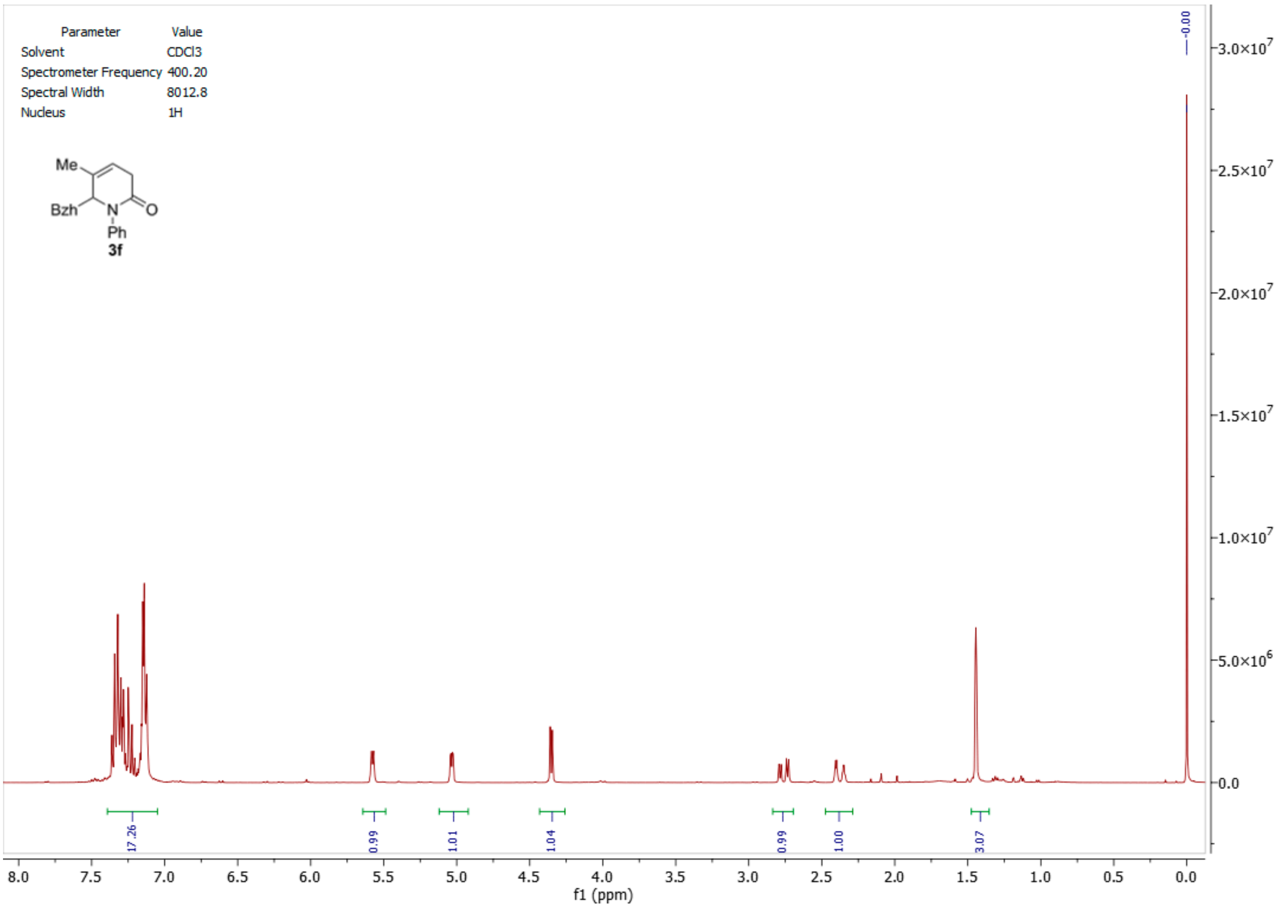

Parameter Value
Solvent CDCl3
Spectrometer Frequency 400.20
Spectral Width 8012.8
Nucleus 1H
Me
Bzh
N
O
Ph
3f
0.00
17.26
0.99
1.01
1.04
0.99
1.00
3.07
f1 (ppm)

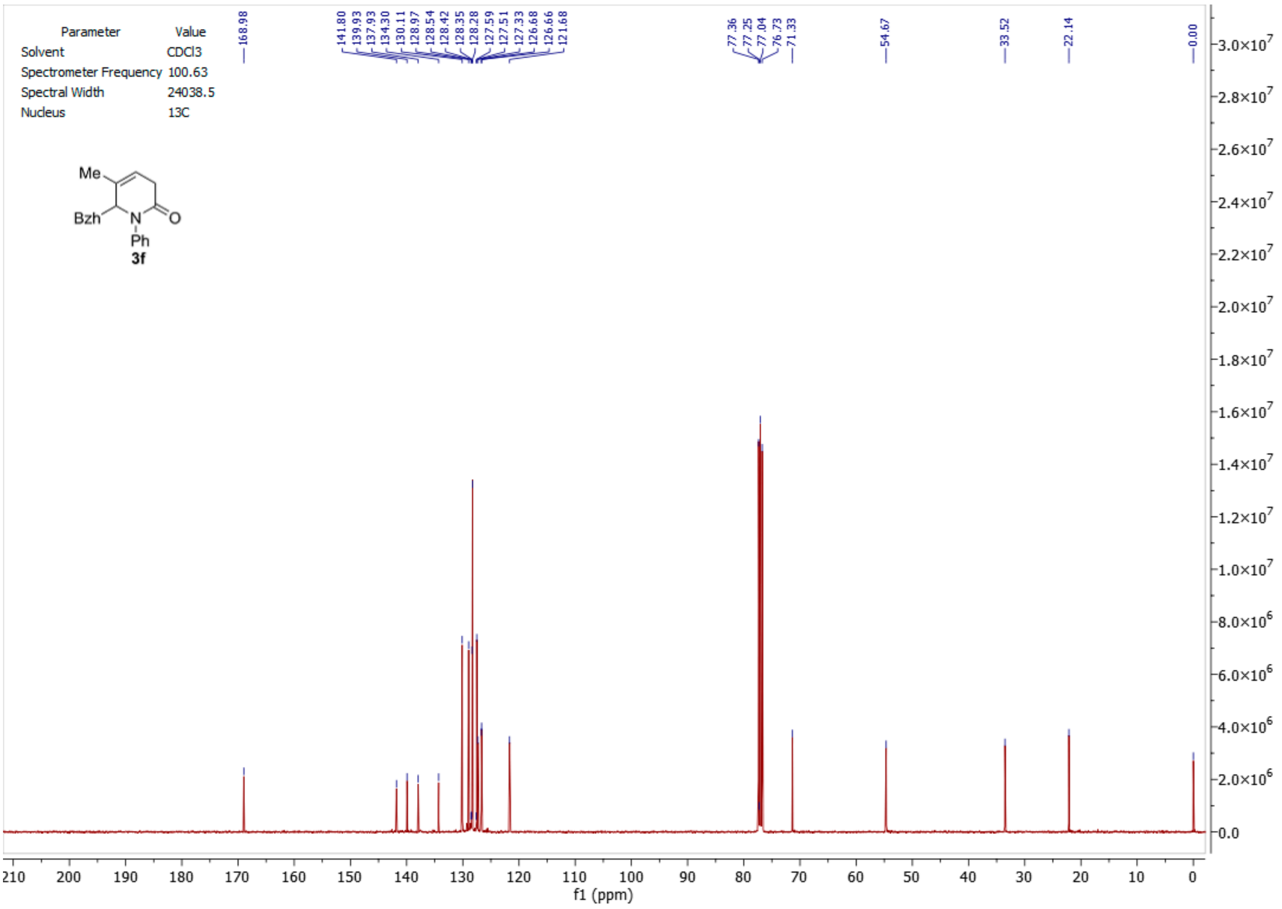

Parameter Value
Solvent CDCl3
Spectrometer Frequency 100.63
Spectral Width 24038.5
Nucleus 13C
Me
Bzh
N
O
Ph
3f
168.98
141.80
139.93
137.93
134.30
130.11
128.97
128.54
128.42
128.35
128.28
127.59
127.51
127.33
126.68
126.66
121.68
77.36
77.25
77.04
76.73
71.33
54.67
33.52
22.14
0.00
f1 (ppm)

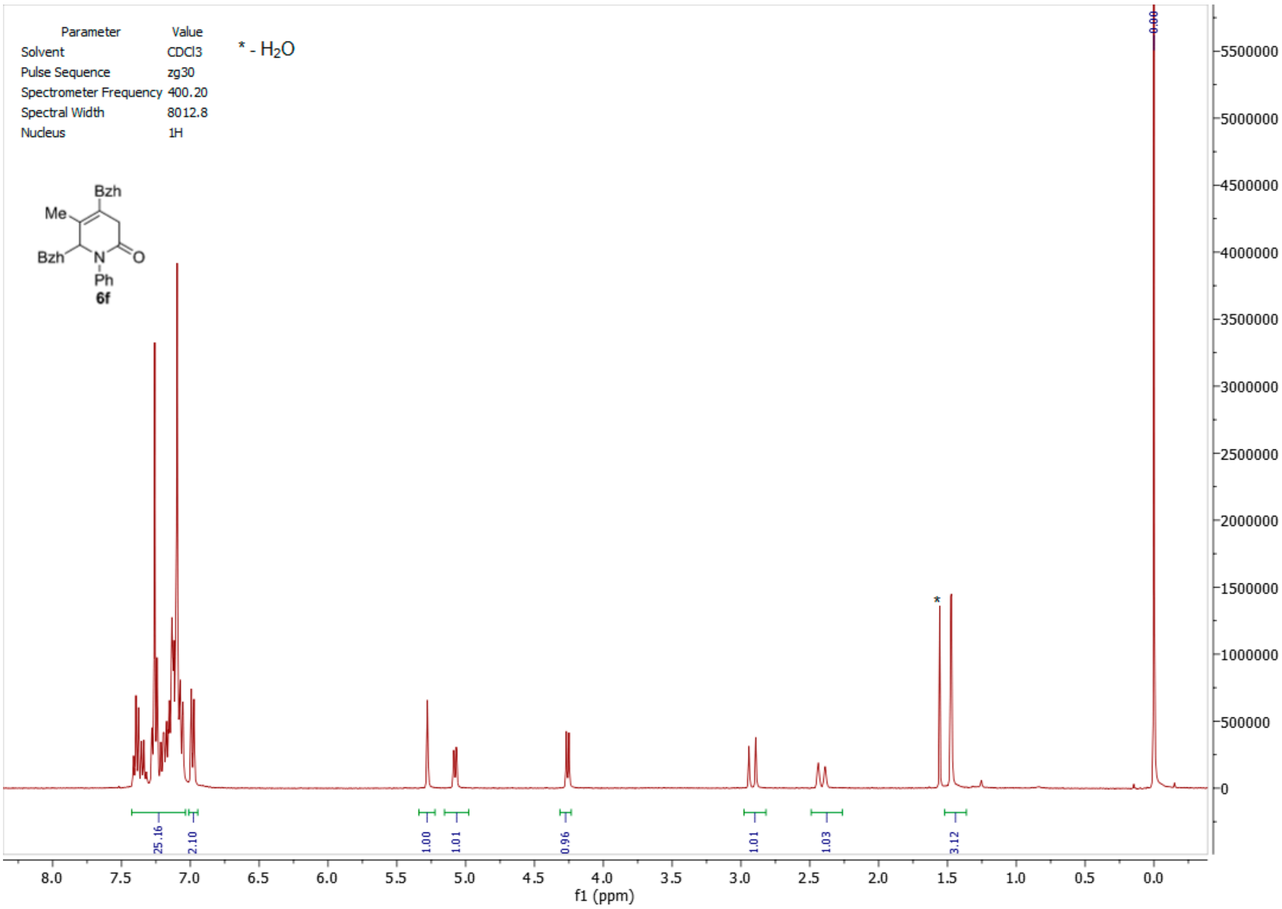
Parameter Value
Solvent CDCl3
Pulse Sequence zg30
Spectrometer Frequency 400.20
Spectral Width 8012.8
Nucleus 1H
* - H2O
Bzh
Me
Bzh
N
O
Ph
6f
f1 (ppm)

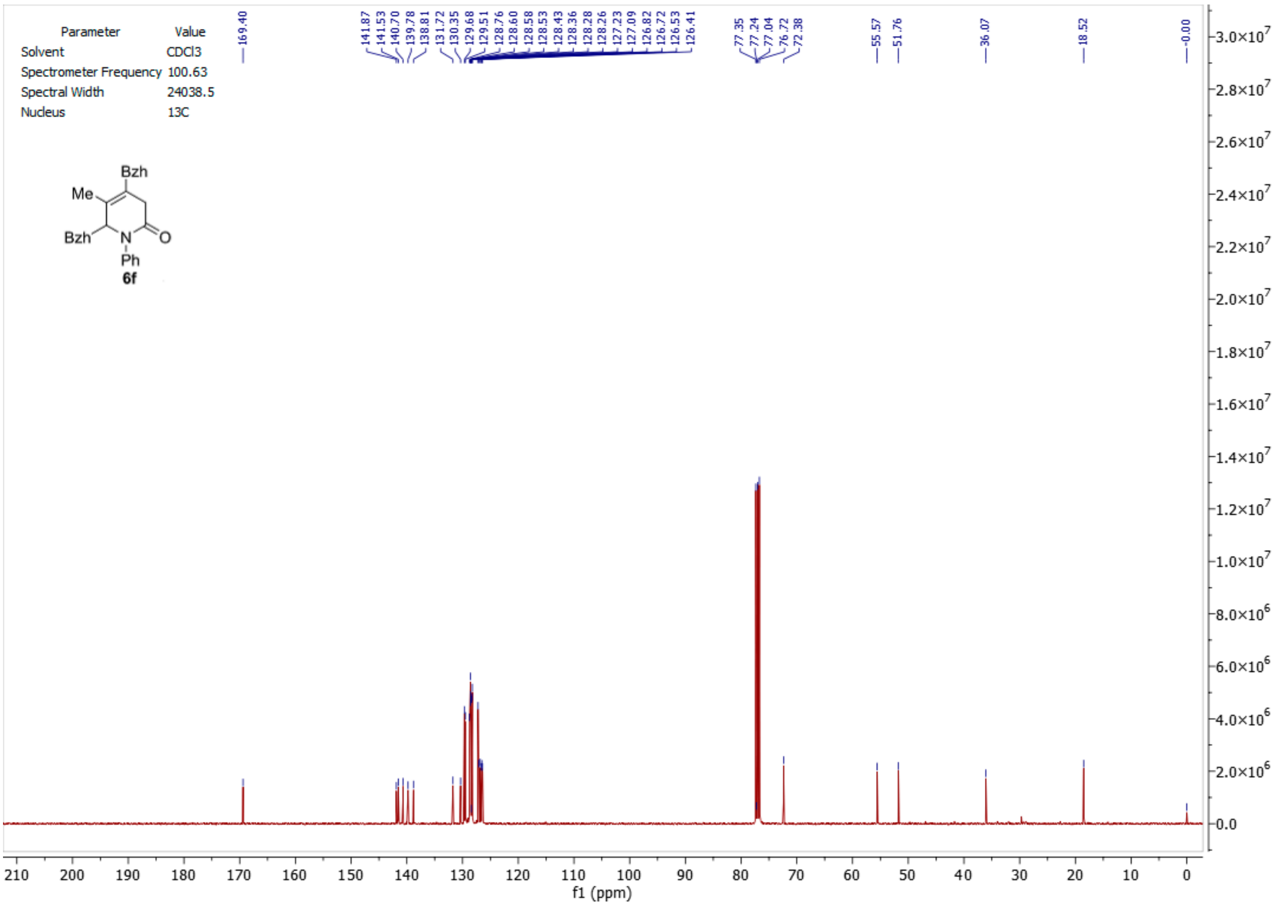
Parameter Value
Solvent CDCl3
Spectrometer Frequency 100.63
Spectral Width 24038.5
Nucleus 13C
Bzh
Me
Bzh
N
O
Ph
6f
f1 (ppm)

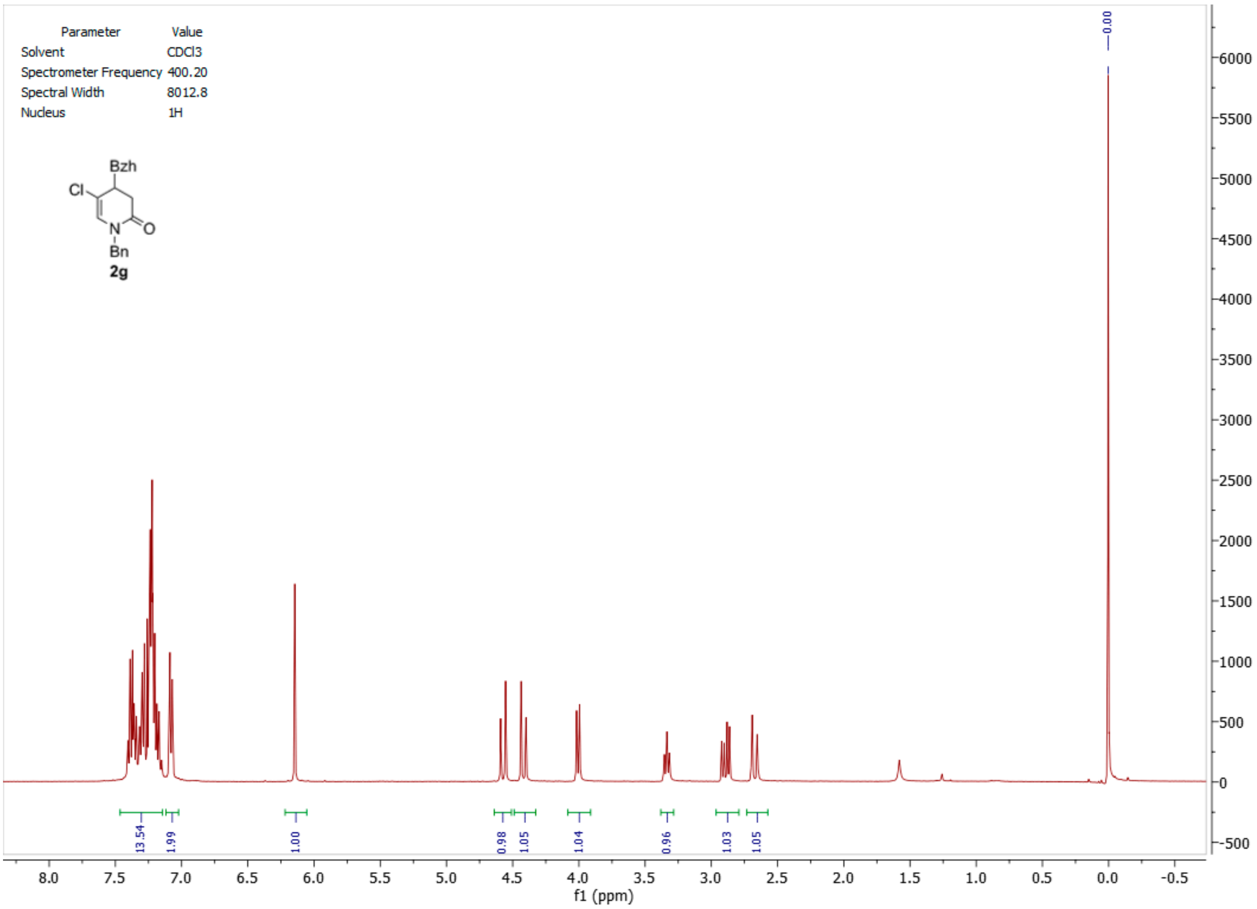

Parameter Value
Solvent CDCl3
Spectrometer Frequency 400.20
Spectral Width 8012.8
Nucleus 1H
Bzh
Cl
N
O
Bn
2g
f1 (ppm)

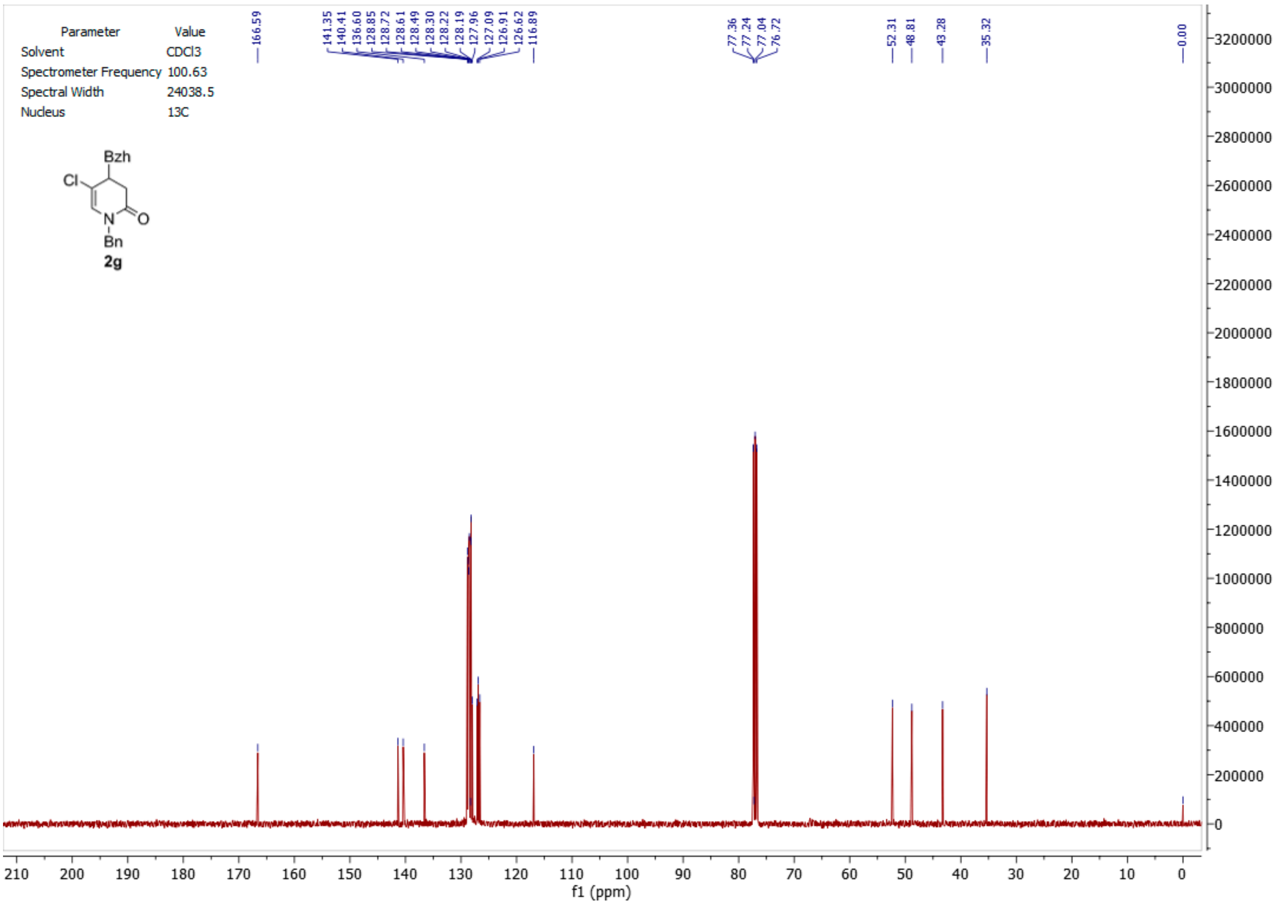

Parameter Value
Solvent CDCl3
Spectrometer Frequency 100.63
Spectral Width 24038.5
Nucleus 13C
Bzh
Cl
N
O
Bn
2g
166.59
141.35
140.41
136.60
128.85
128.72
128.61
128.49
128.30
128.22
128.19
127.96
127.09
126.91
126.62
116.89
77.36
77.24
77.04
76.72
52.31
48.81
43.28
35.32
0.00
f1 (ppm)

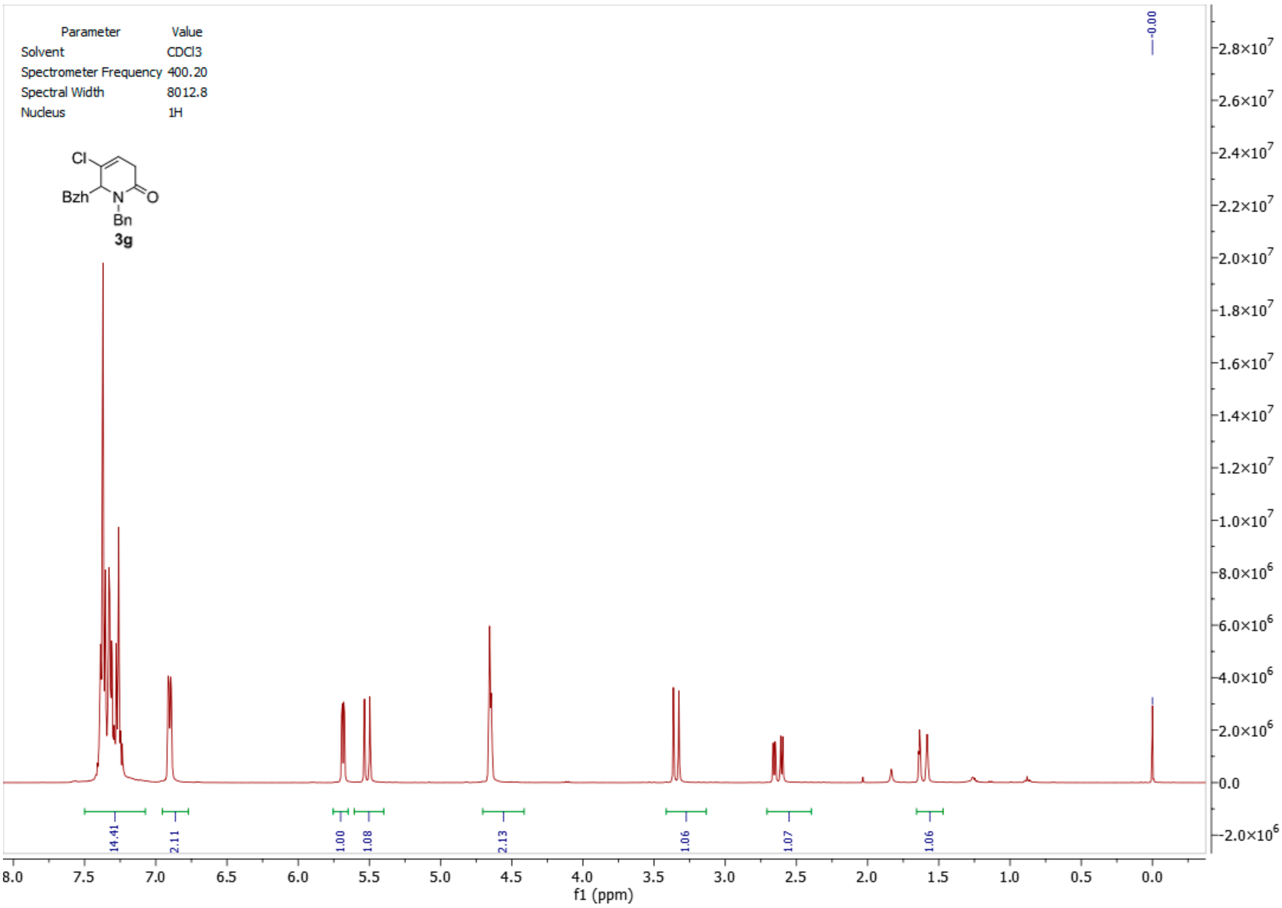

Parameter Value
Solvent CDCl3
Spectrometer Frequency 400.20
Spectral Width 8012.8
Nucleus 1H
Cl
Bzh
N
O
Bn
3g
14.41
2.11
1.00
1.08
2.13
1.06
1.07
1.06
0.00
f1 (ppm)

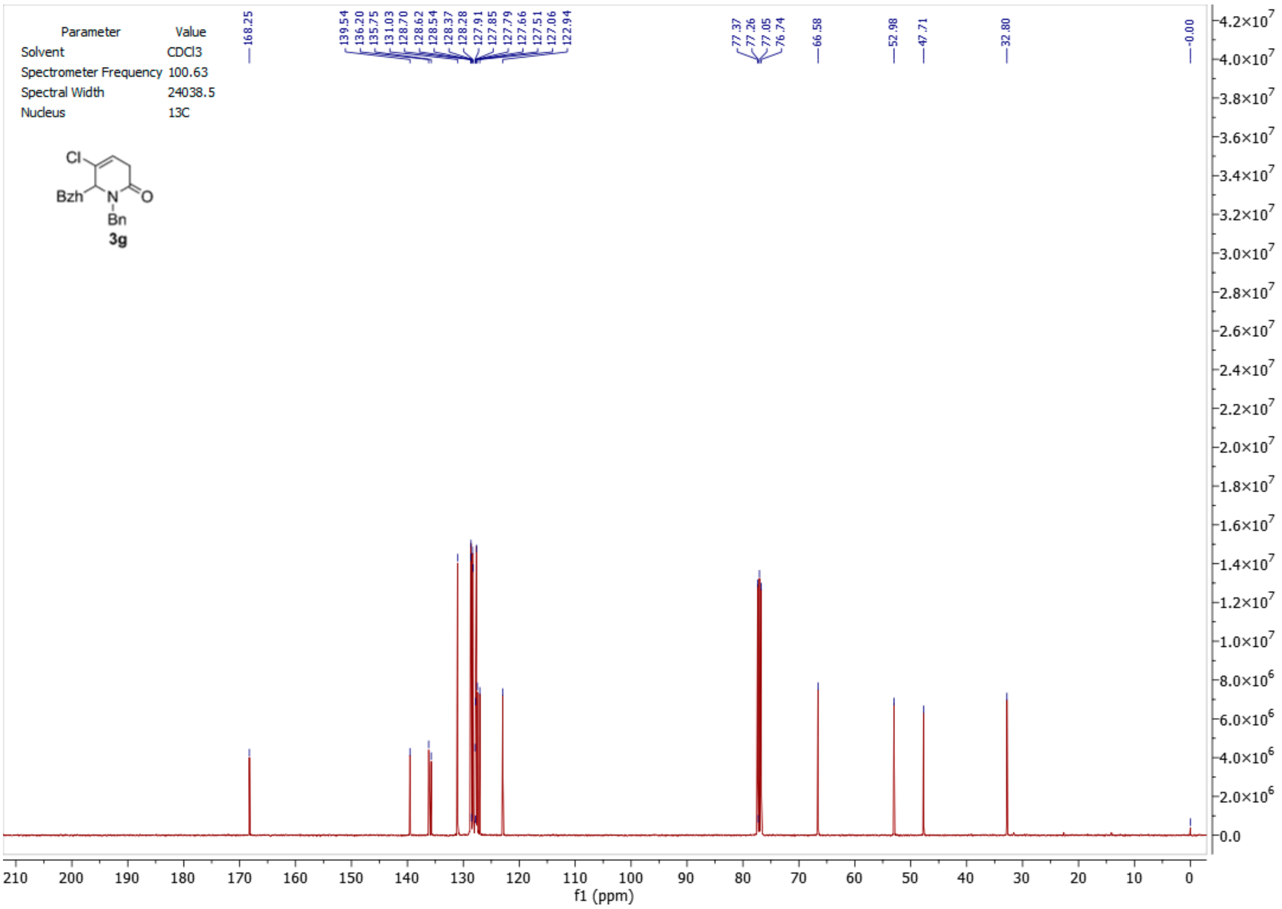

Parameter Value
Solvent CDCl3
Spectrometer Frequency 100.63
Spectral Width 24038.5
Nucleus 13C
Cl
Bzh
N
O
Bn
3g
168.25
139.54
136.20
135.75
131.03
128.70
128.62
128.54
128.37
128.28
127.91
127.85
127.79
127.66
127.51
127.06
122.94
77.37
77.26
77.05
76.74
66.58
52.98
47.71
32.80
0.00
f1 (ppm)

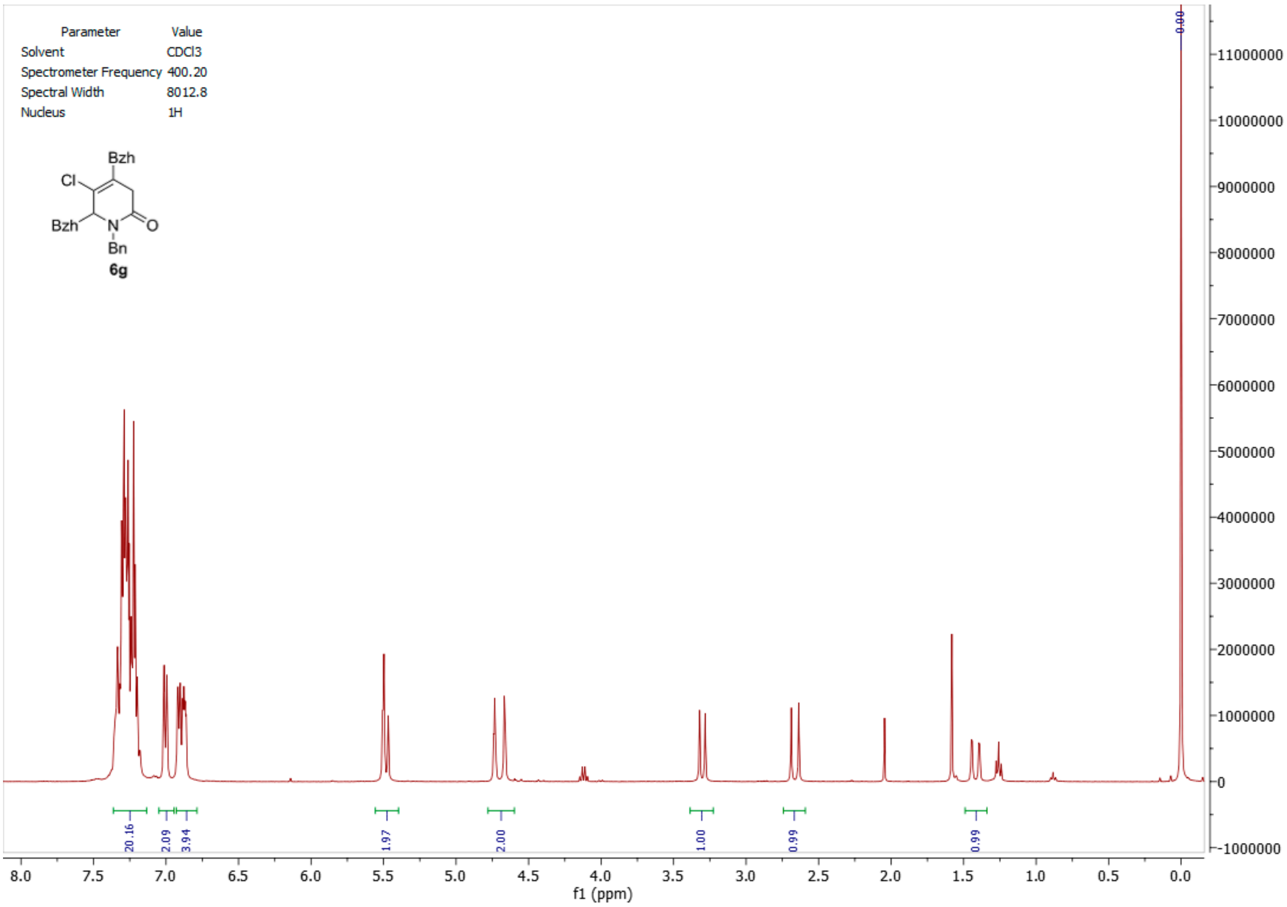

Parameter Value
Solvent CDCl3
Spectrometer Frequency 400.20
Spectral Width 8012.8
Nucleus 1H
Bzh
Cl
Bzh
N
O
Bn
6g
f1 (ppm)

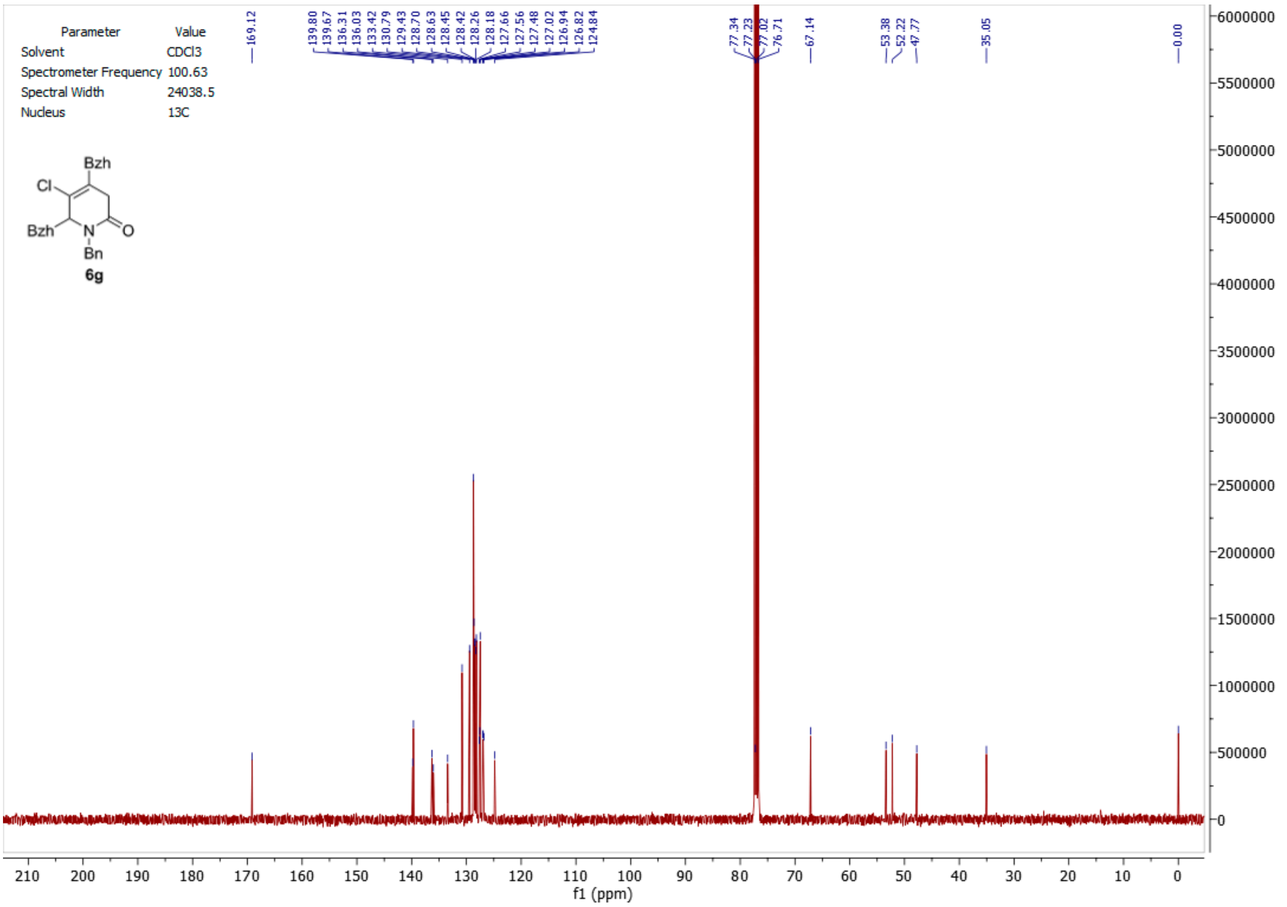

Parameter Value
Solvent CDCl3
Spectrometer Frequency 100.63
Spectral Width 24038.5
Nucleus 13C
Bzh
Cl
Bzh
N
O
Bn
6g
f1 (ppm)

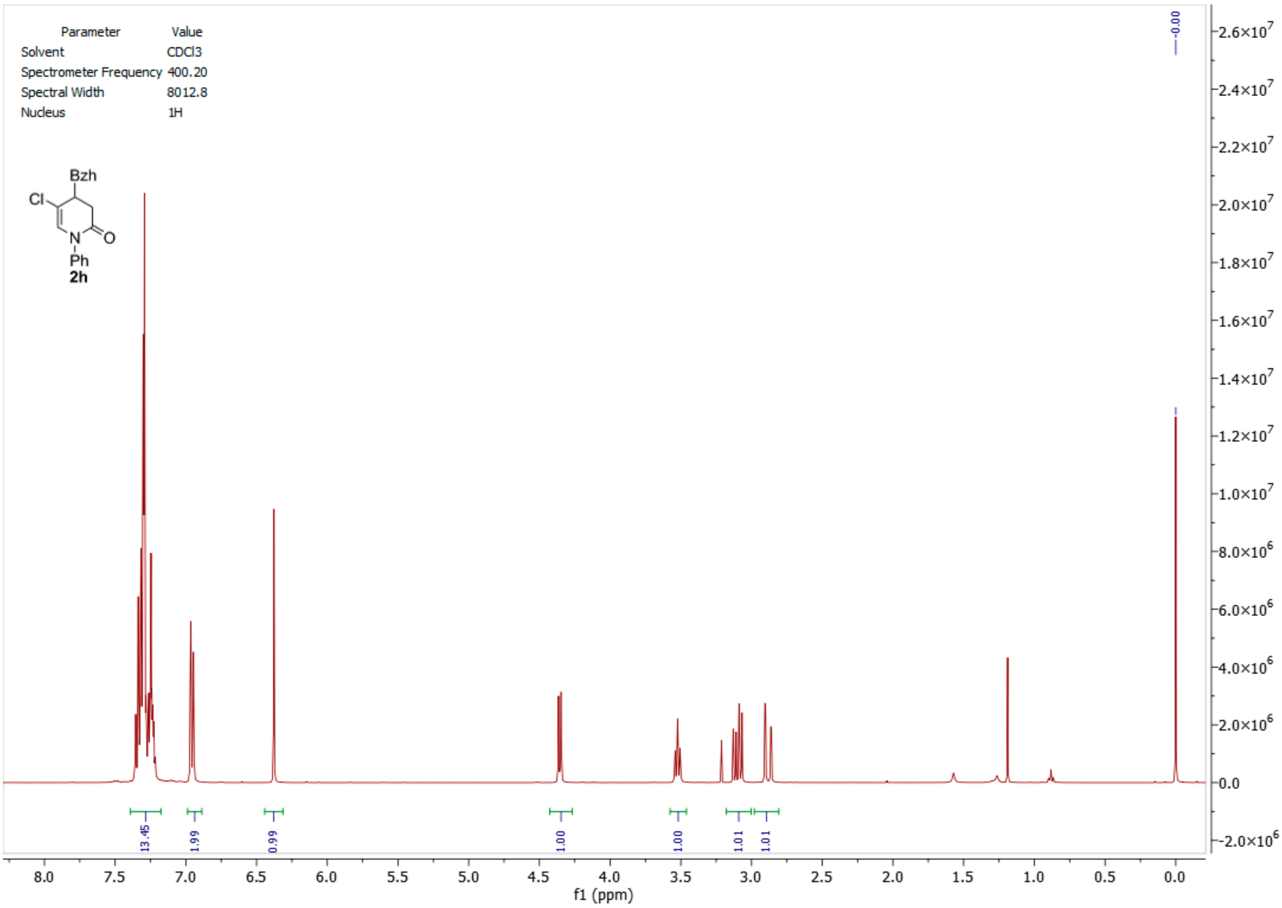
Parameter Value
Solvent CDCl3
Spectrometer Frequency 400.20
Spectral Width 8012.8
Nucleus 1H
Bzh
Cl
N
O
Ph
2h
f1 (ppm)

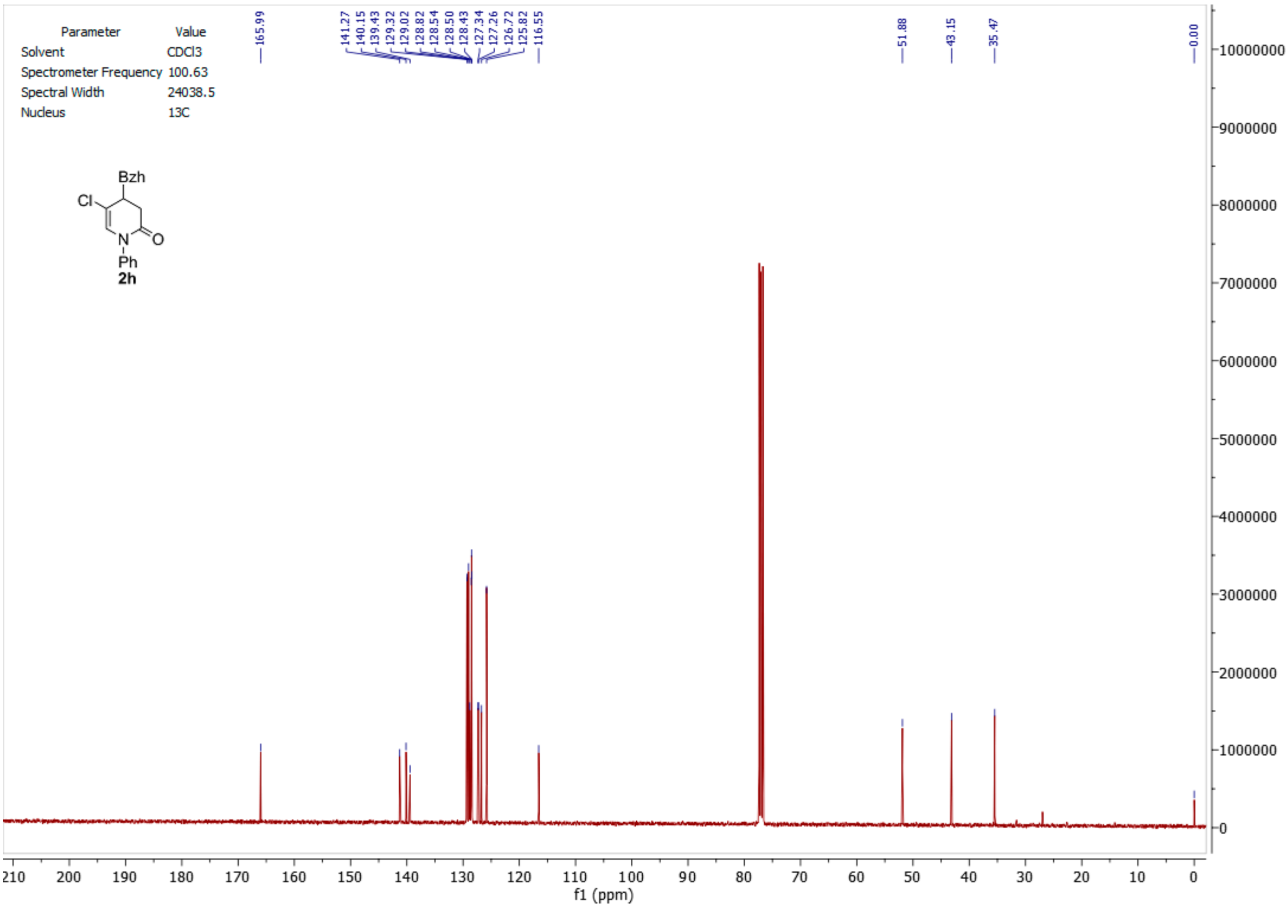
Parameter Value
Solvent CDCl3
Spectrometer Frequency 100.63
Spectral Width 24038.5
Nucleus 13C
Bzh
Cl
N
O
Ph
2h
165.99
141.27
140.15
139.43
129.32
129.02
128.82
128.54
128.50
128.43
127.34
127.26
126.72
125.82
116.55
51.88
43.15
35.47
0.00
f1 (ppm)

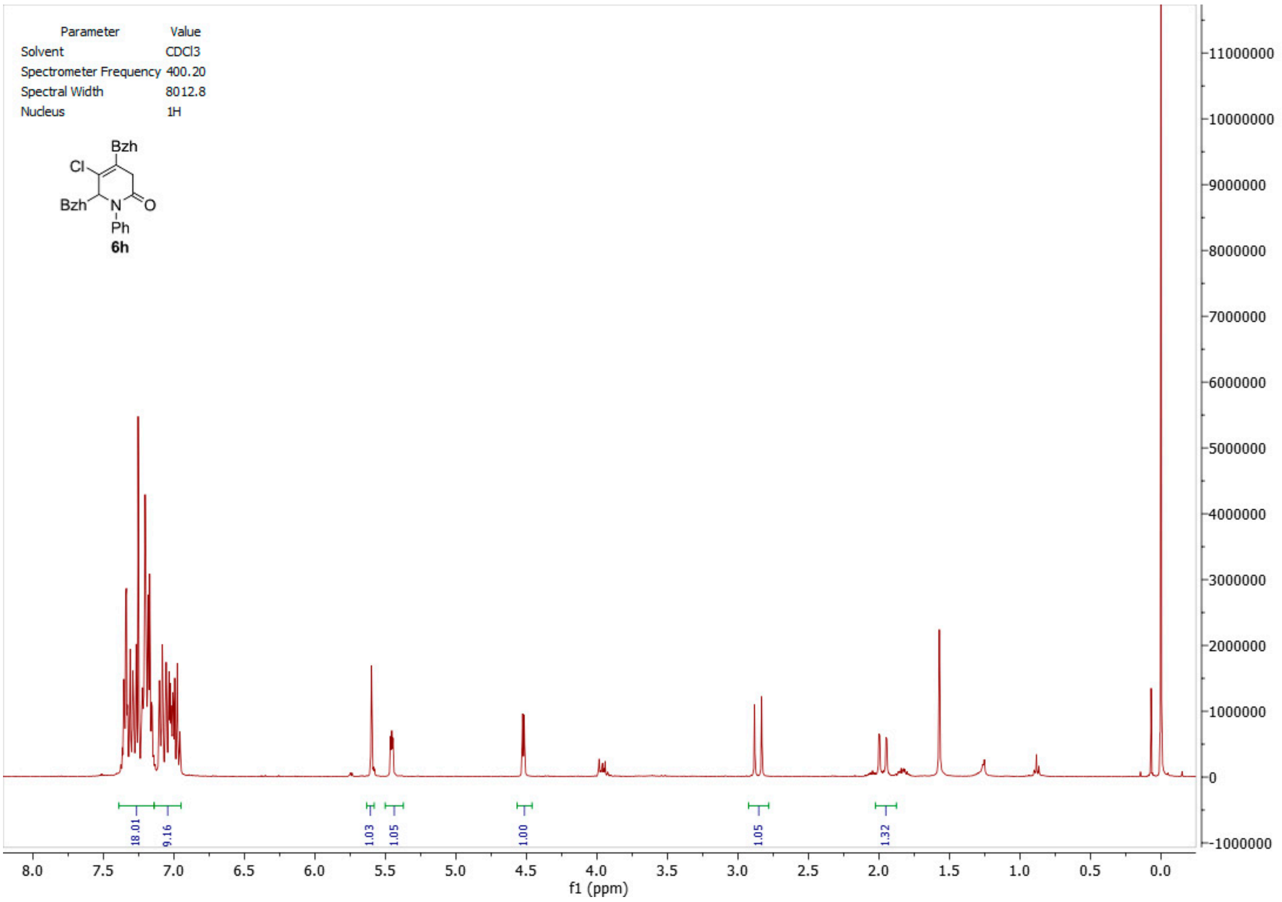

Parameter Value
Solvent CDCl3
Spectrometer Frequency 400.20
Spectral Width 8012.8
Nucleus 1H
Bzh
Cl
Bzh
N
O
Ph
6h
18.01
9.16
1.03
1.05
1.00
1.05
1.32
f1 (ppm)

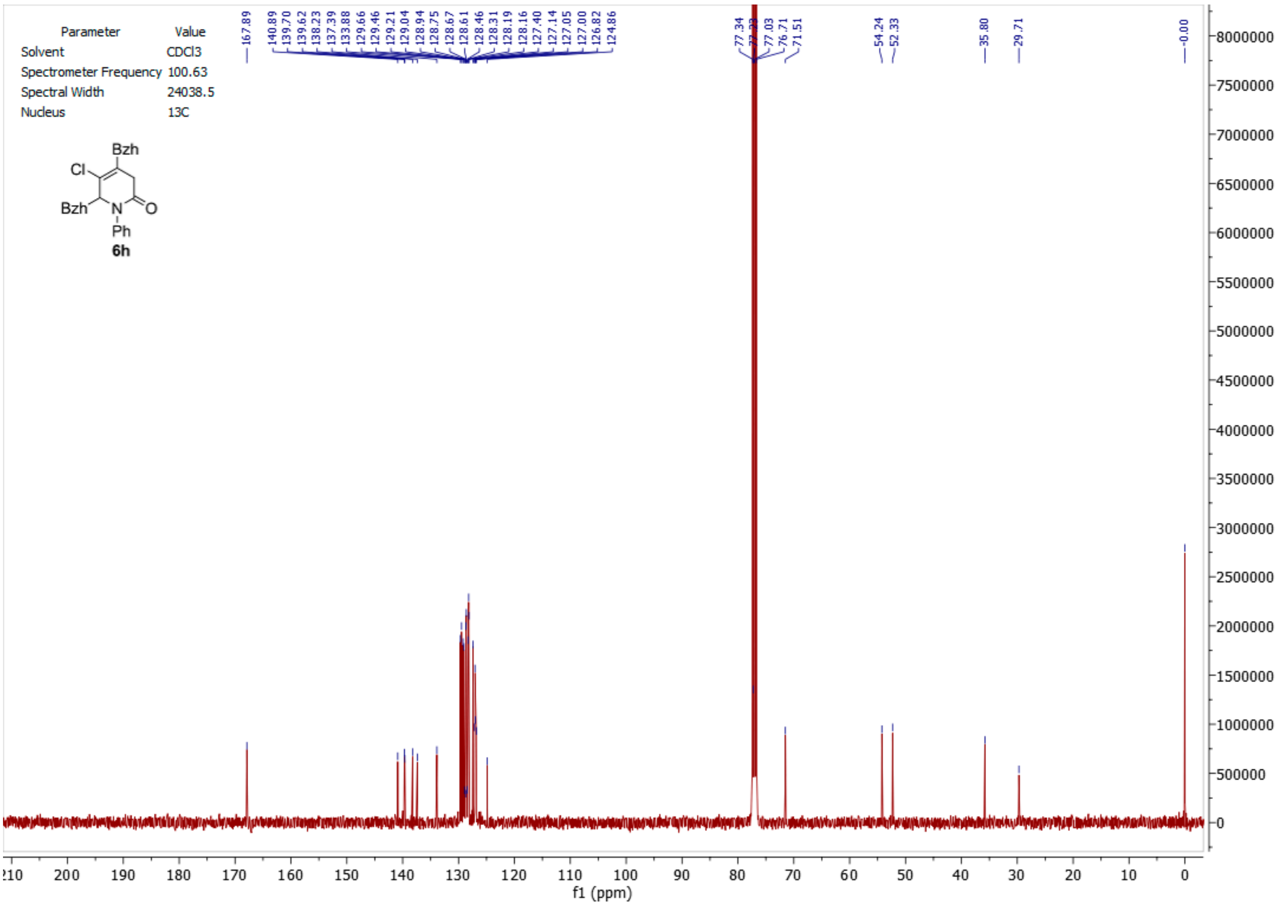

Parameter Value
Solvent CDCl3
Spectrometer Frequency 100.63
Spectral Width 24038.5
Nucleus 13C
Bzh
Cl
Bzh
N
O
Ph
6h
167.89
140.89
139.70
139.62
138.23
137.39
133.88
129.66
129.46
129.21
129.04
128.94
128.75
128.67
128.61
128.46
128.31
128.19
128.16
127.40
127.14
127.05
127.00
126.82
124.86
77.34
77.03
76.71
71.51
54.24
52.33
35.80
29.71
-0.00
f1 (ppm)

Parameter	Value
Solvent	CDCl3
Spectrometer Frequency	400.20
Spectral Width	8012.8
Nucleus	1H

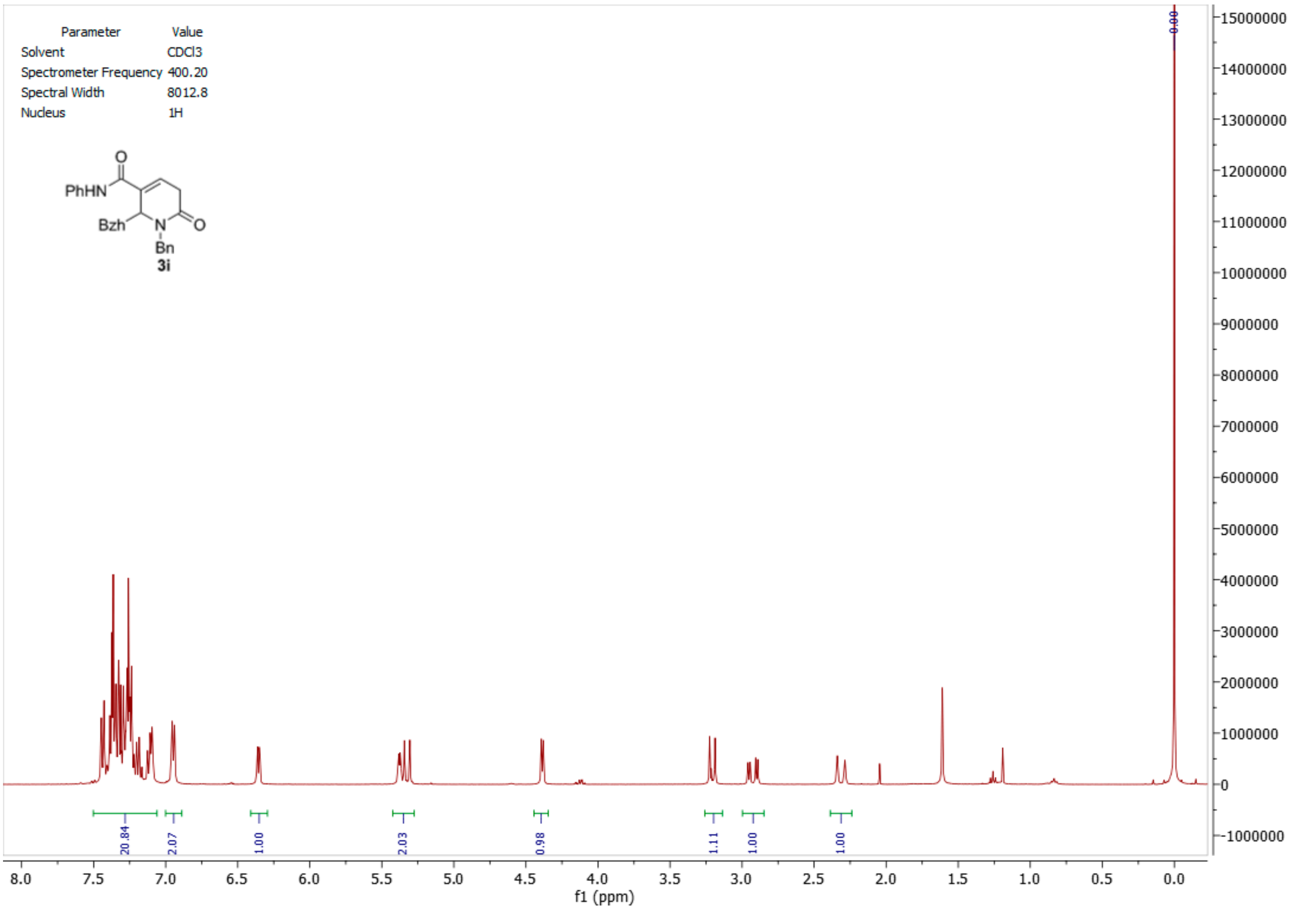

Parameter	Value
Solvent	CDCl3
Spectrometer Frequency	100.63
Spectral Width	24038.5
Nucleus	13C

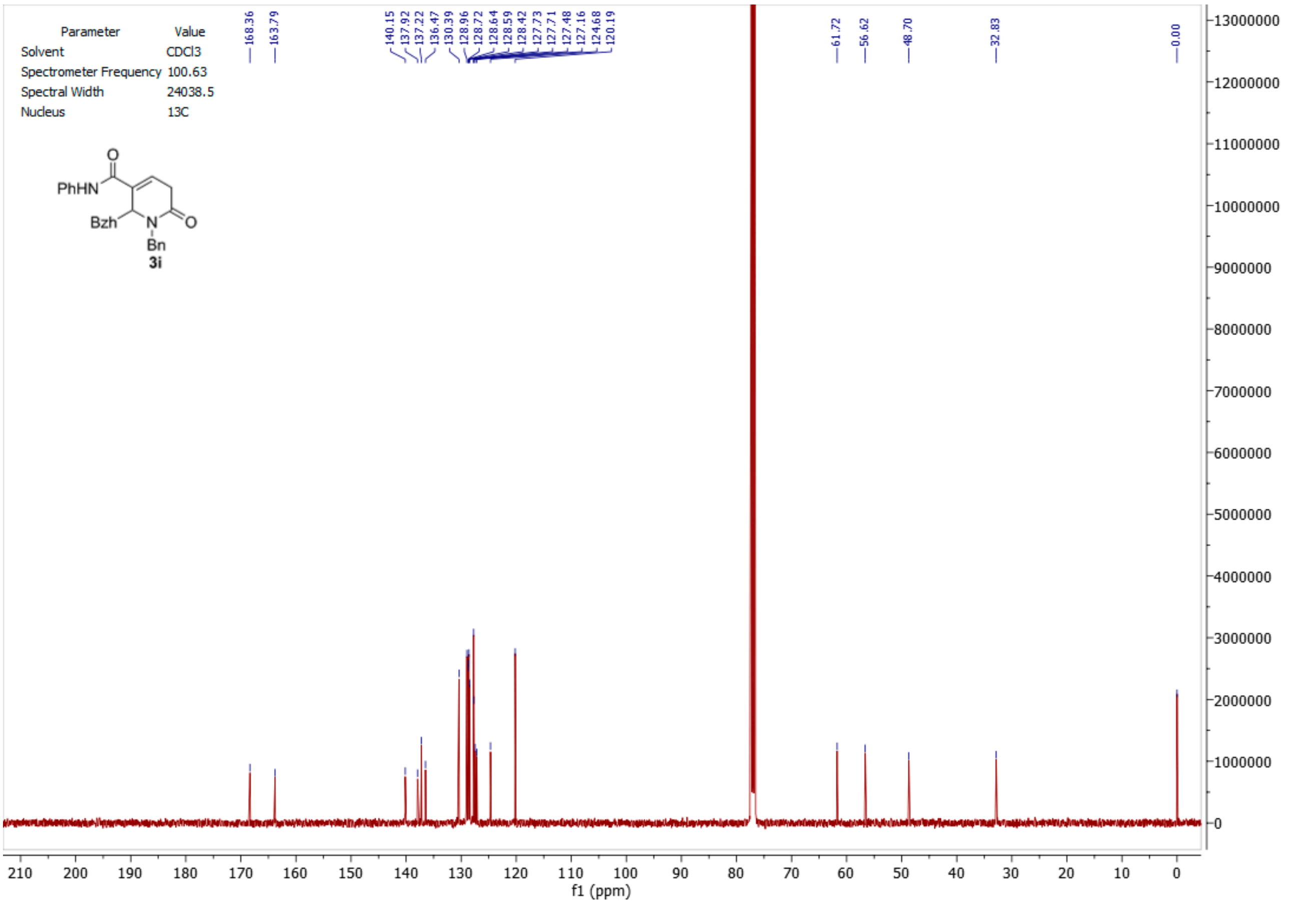

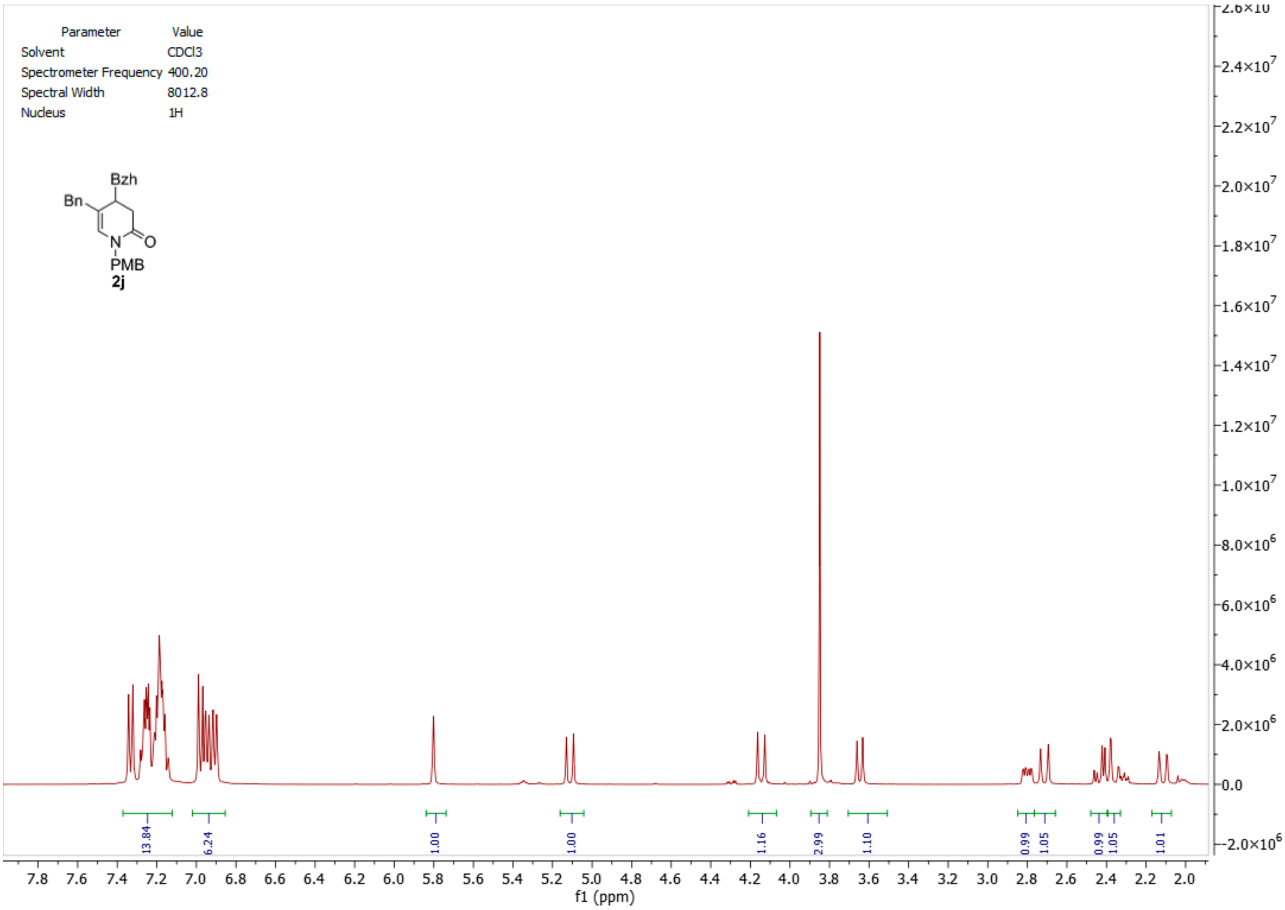
Parameter Value
Solvent CDCl3
Spectrometer Frequency 400.20
Spectral Width 8012.8
Nucleus 1H
Bzh
Bn
N
O
PMB
2j
13.84
6.24
1.00
1.00
1.16
2.99
1.10
0.99
1.05
0.99
1.05
1.01
f1 (ppm)

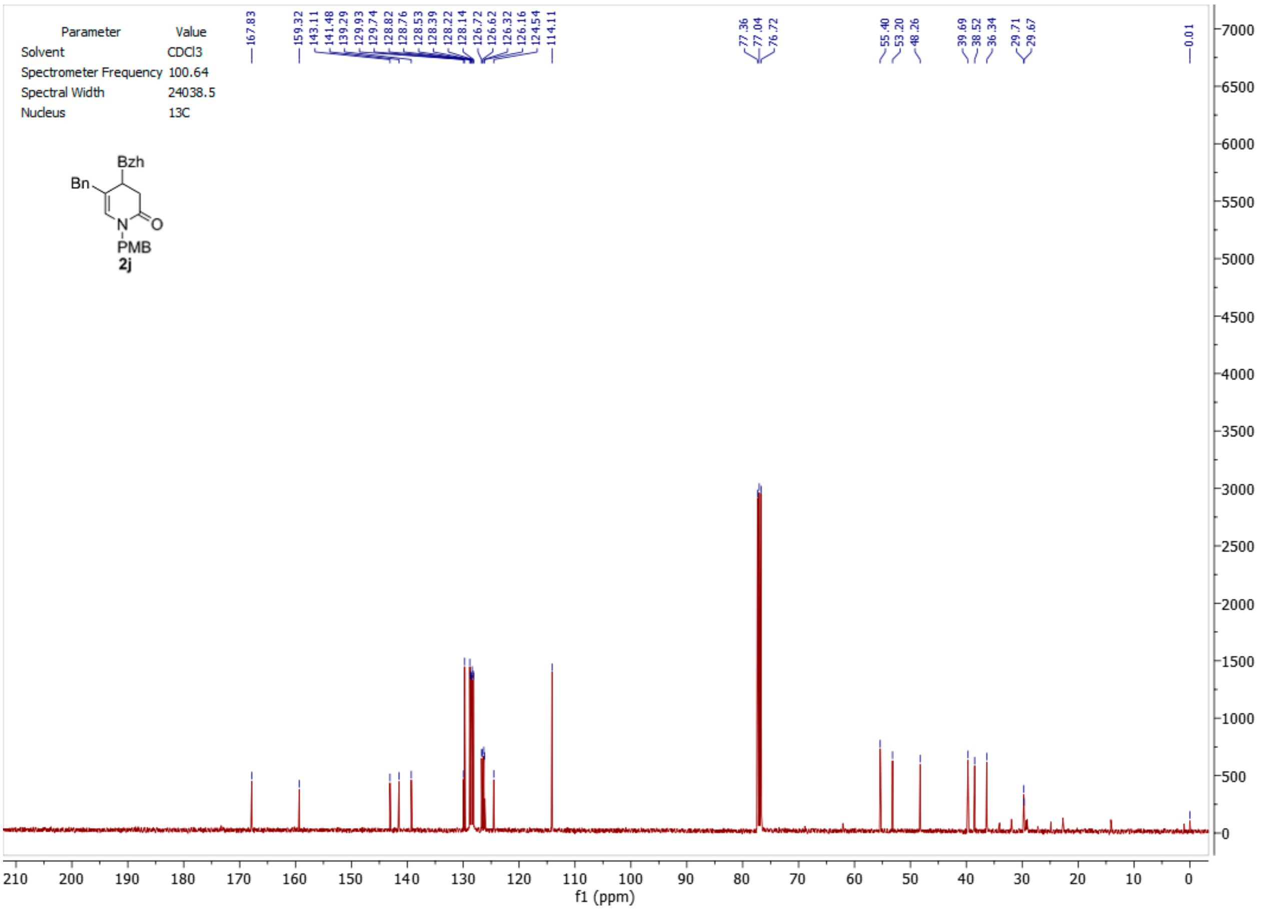
Parameter Value
Solvent CDCl3
Spectrometer Frequency 100.64
Spectral Width 24038.5
Nucleus 13C
Bzh
Bn
N
O
PMB
2j
167.83
159.32
143.11
141.48
139.29
129.93
129.74
128.82
128.76
128.53
128.39
128.22
128.14
126.72
126.62
126.32
126.16
124.54
114.11
77.36
77.04
76.72
55.40
53.20
48.26
39.69
38.52
36.34
29.71
29.67
0.01
f1 (ppm)

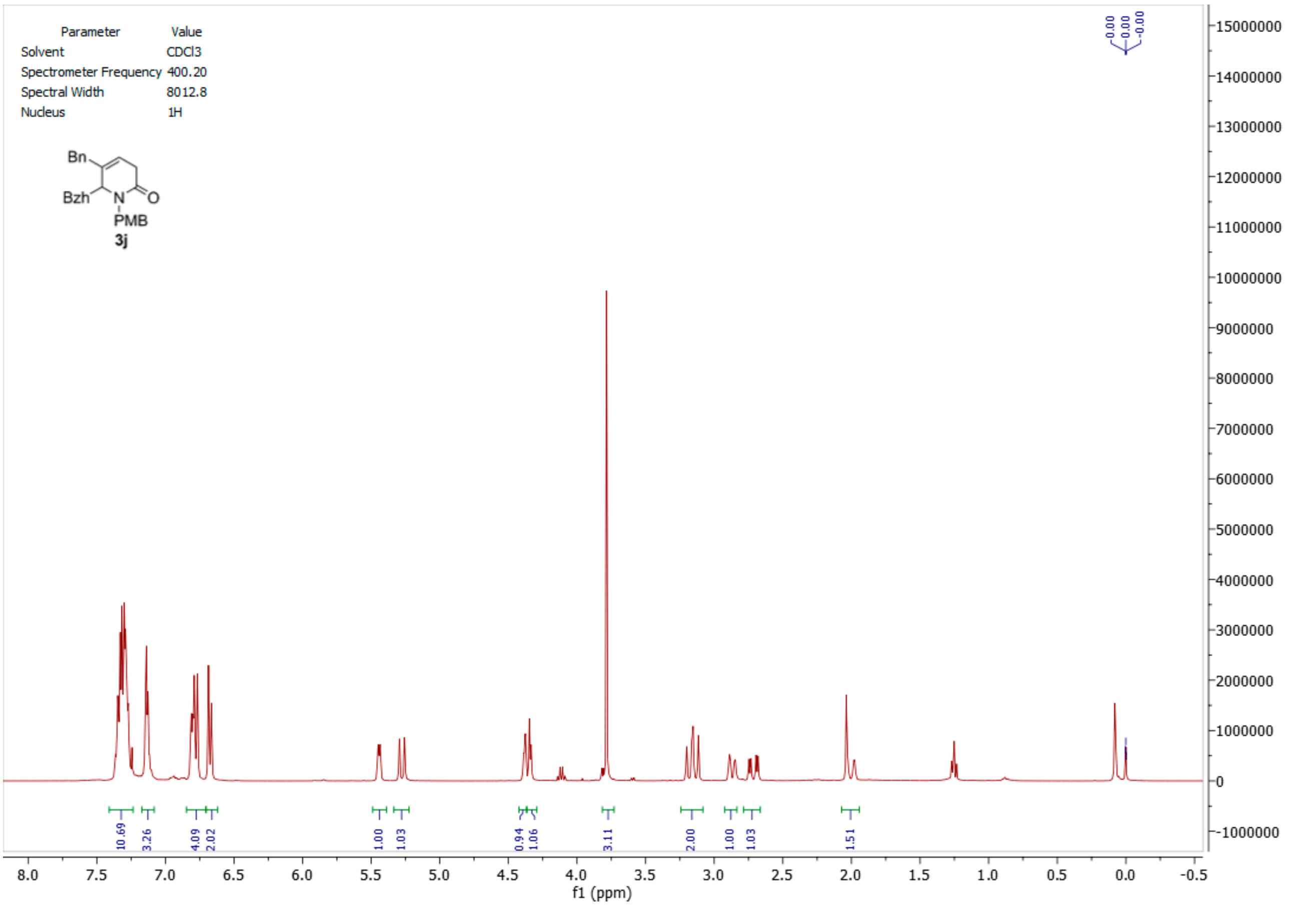
Parameter Value
Solvent CDCl3
Spectrometer Frequency 400.20
Spectral Width 8012.8
Nucleus 1H
Bn
Bzh
N
O
PMB
3j
0.00 0.00 -0.00
10.69 3.26 4.09 2.02 1.00 1.03 0.94 1.06 3.11 2.00 1.00 1.03 1.51
f1 (ppm)

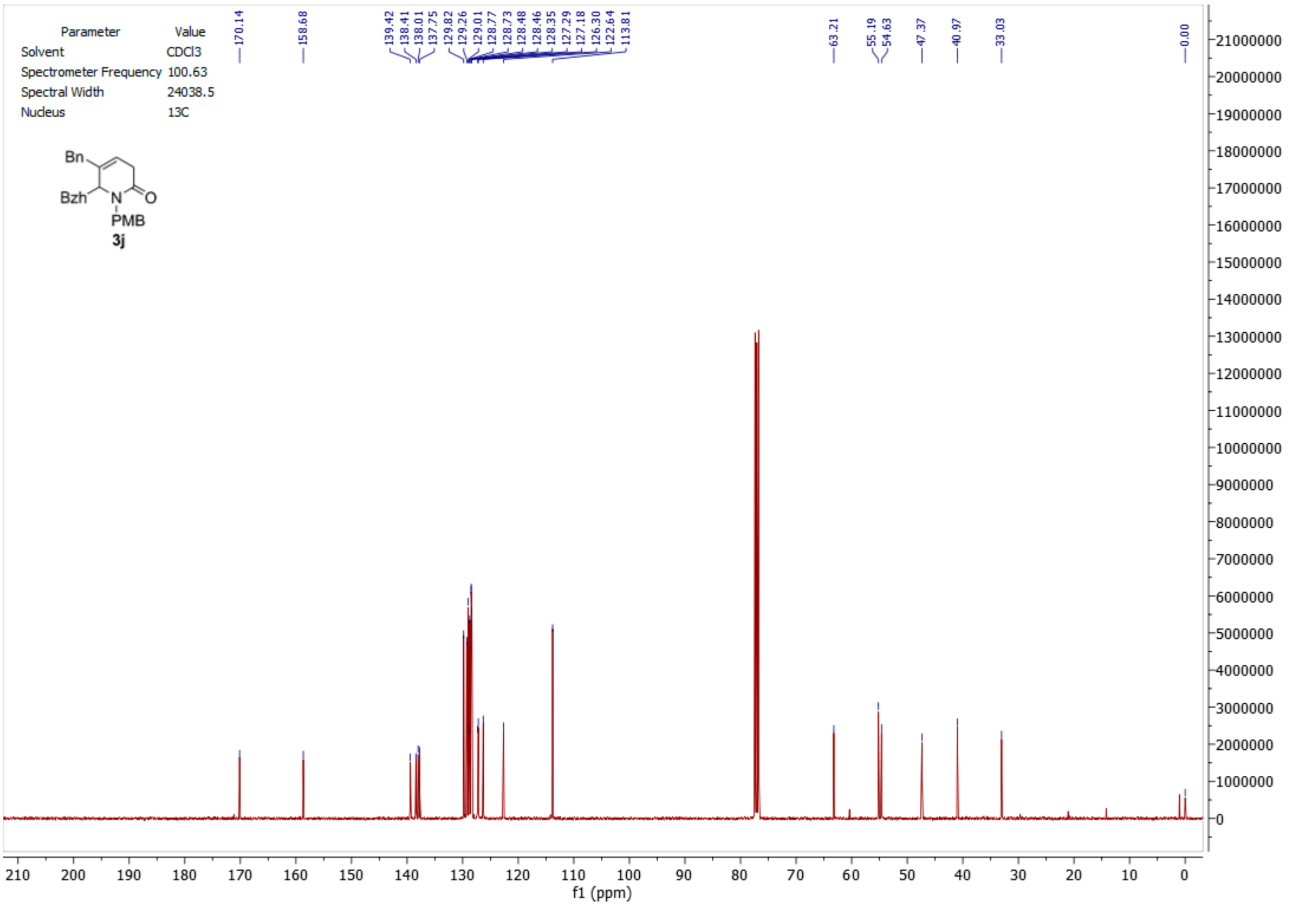
Parameter Value
Solvent CDCl3
Spectrometer Frequency 100.63
Spectral Width 24038.5
Nucleus 13C
Bn
Bzh
N
O
PMB
3j
170.14 158.68 139.42 138.41 138.01 137.75 129.82 129.26 129.01 128.77 128.73 128.48 128.46 128.35 127.29 127.18 126.30 122.64 113.81 63.21 55.19 54.63 47.37 40.97 33.03 0.00
f1 (ppm)

Parameter	Value
Solvent	CDCl3
Spectrometer Frequency	400.20
Spectral Width	8012.8
Nucleus	1H

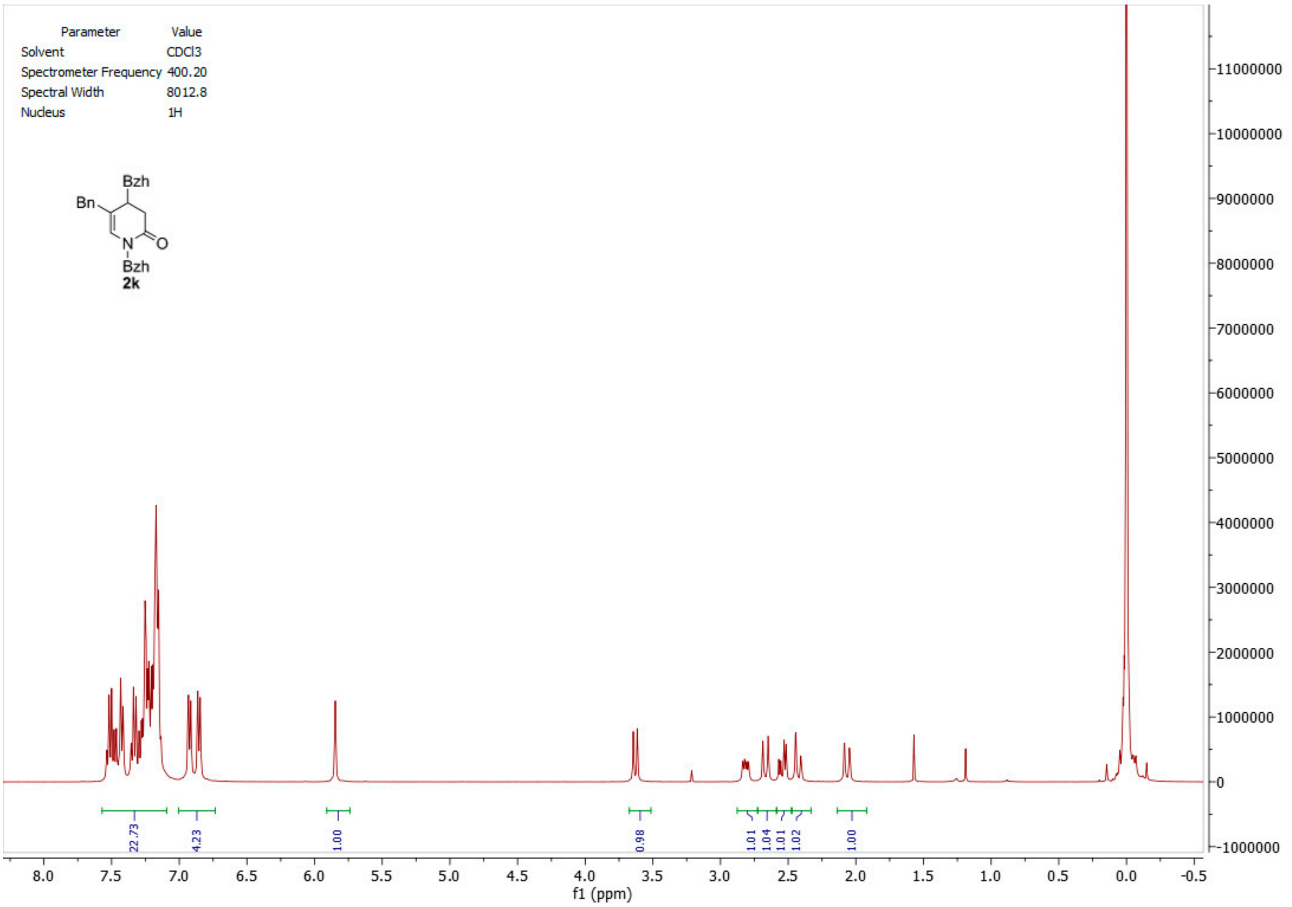

Parameter	Value
Solvent	CDCl3
Spectrometer Frequency	100.63
Spectral Width	24038.5
Nucleus	13C

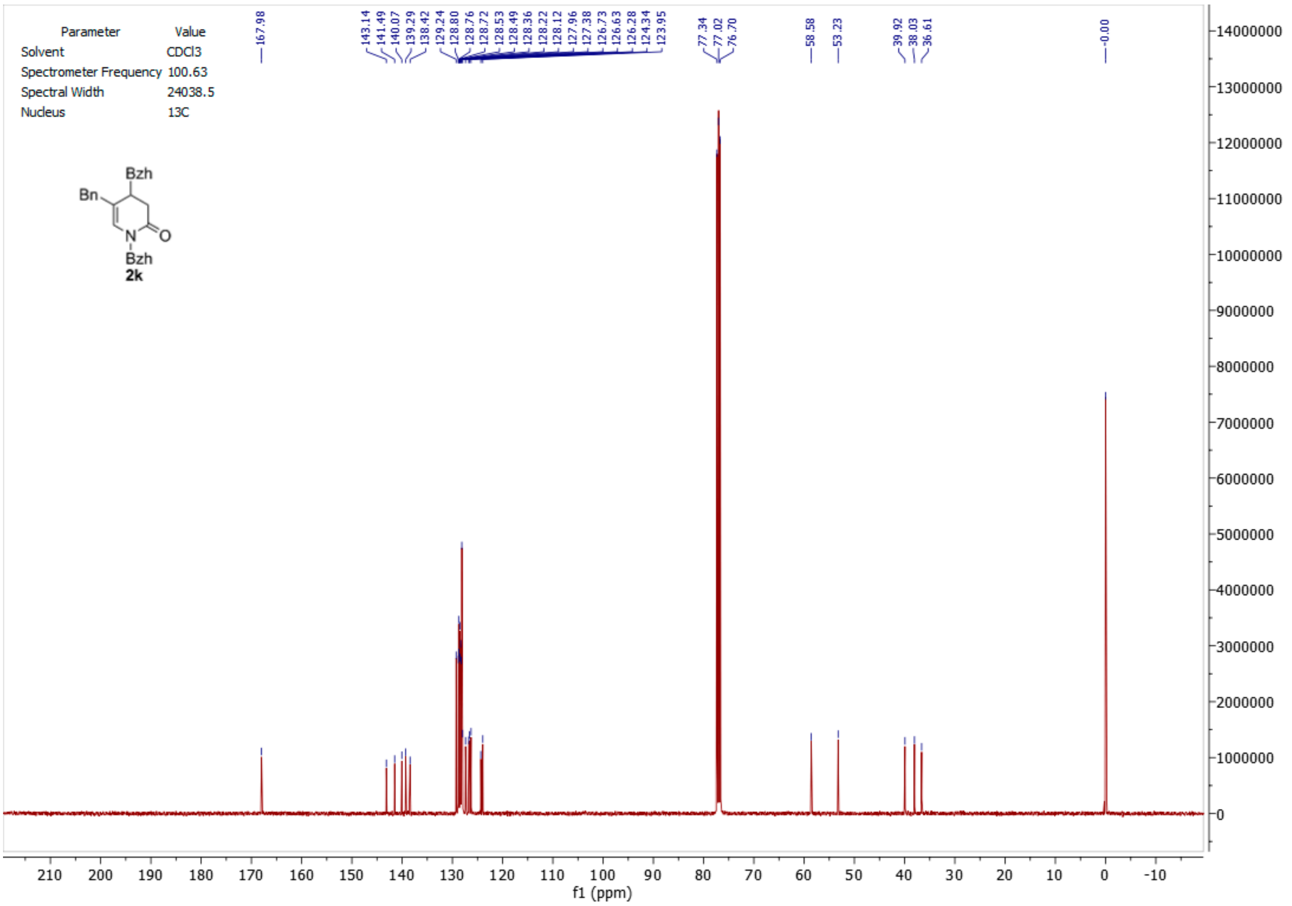

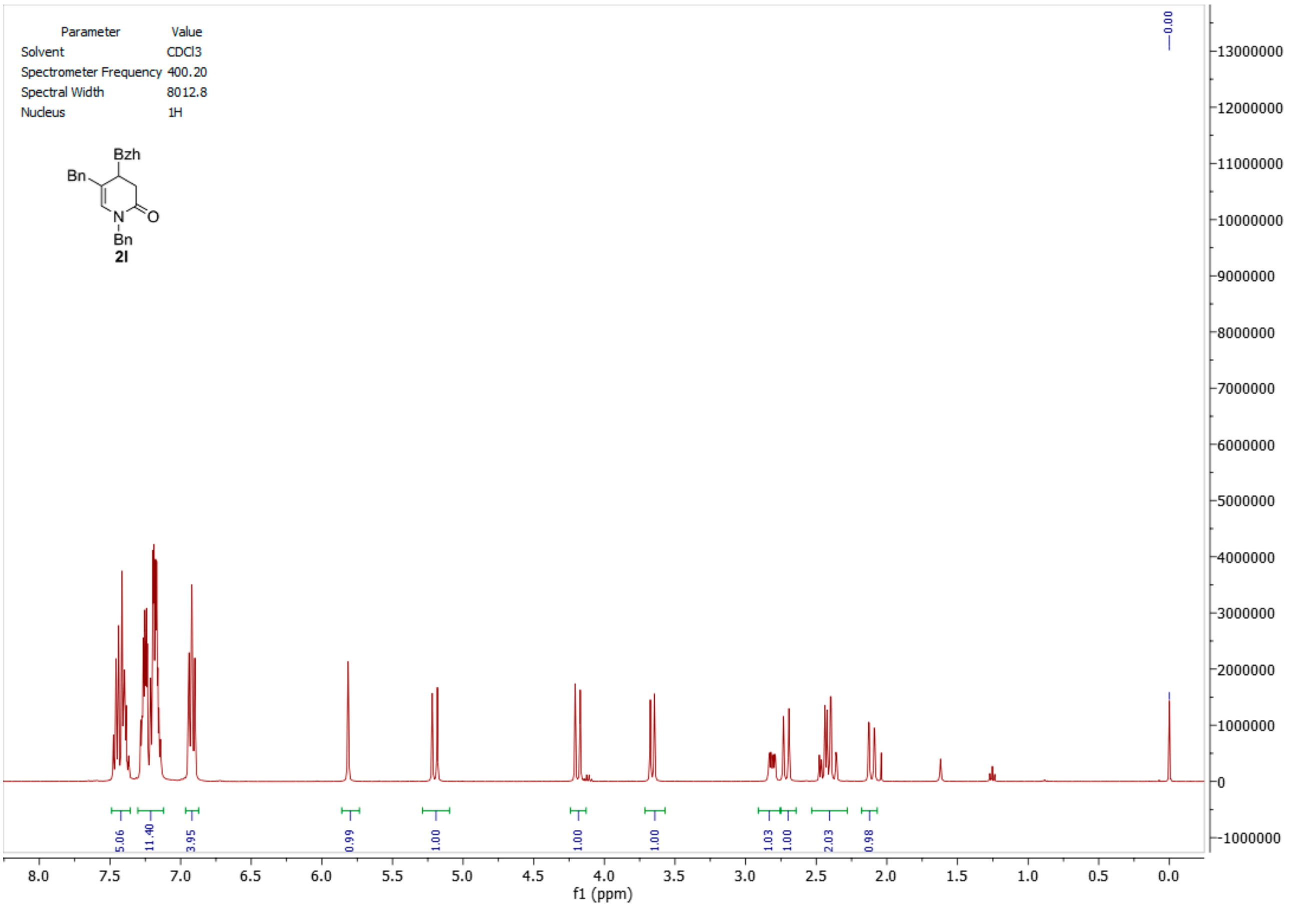

Parameter Value
Solvent CDCl3
Spectrometer Frequency 400.20
Spectral Width 8012.8
Nucleus 1H
Bzh
Bn
N
O
Bn
2l
0.00
5.06
11.40
3.95
0.99
1.00
1.00
1.00
1.03
1.00
2.03
0.98
f1 (ppm)

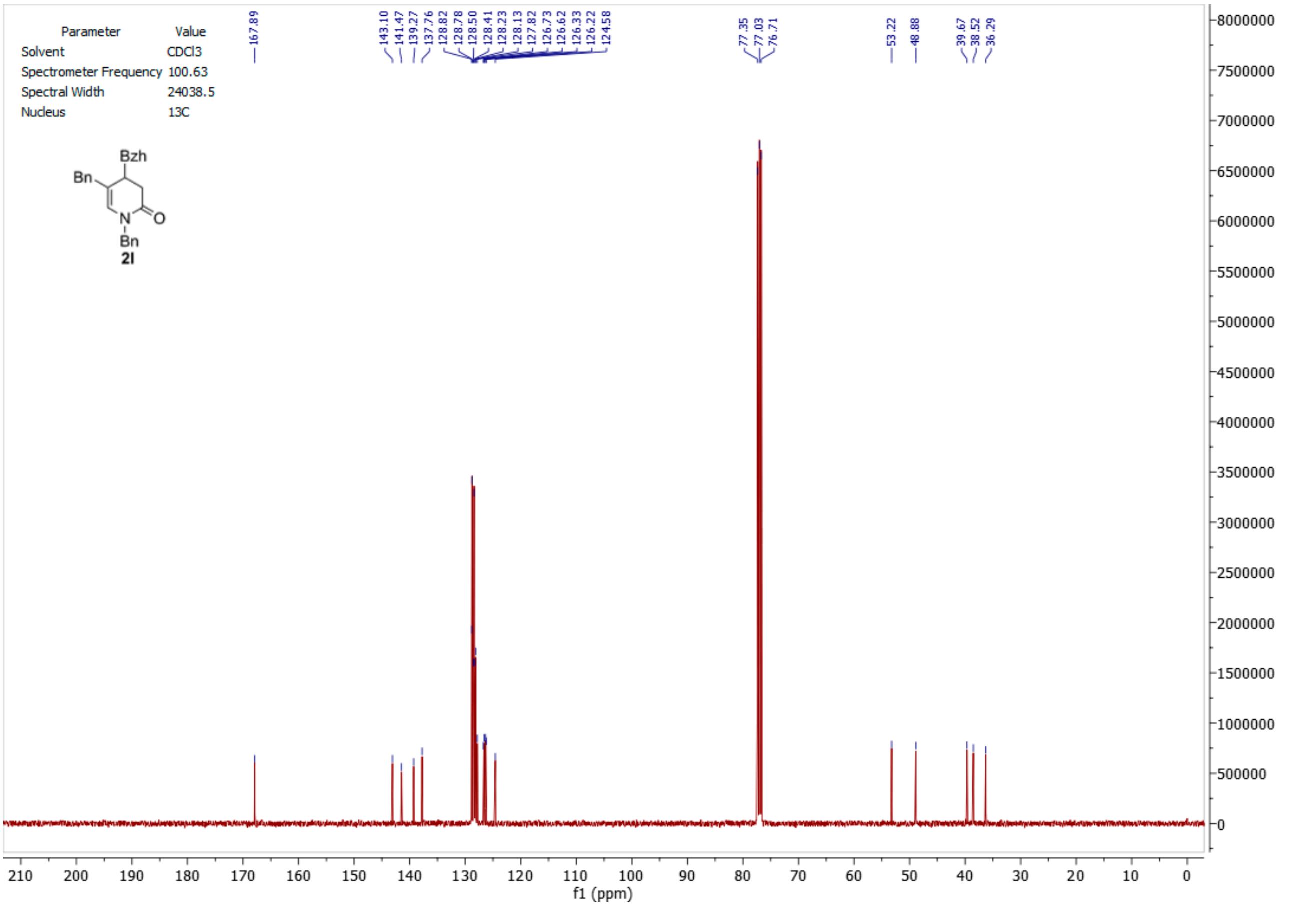

Parameter Value
Solvent CDCl3
Spectrometer Frequency 100.63
Spectral Width 24038.5
Nucleus 13C
Bzh
Bn
N
O
Bn
2l
167.89
143.10
141.47
139.27
137.76
128.82
128.78
128.50
128.41
128.23
128.13
127.82
126.73
126.62
126.33
126.22
124.58
77.35
77.03
76.71
53.22
48.88
39.67
38.52
36.29
f1 (ppm)

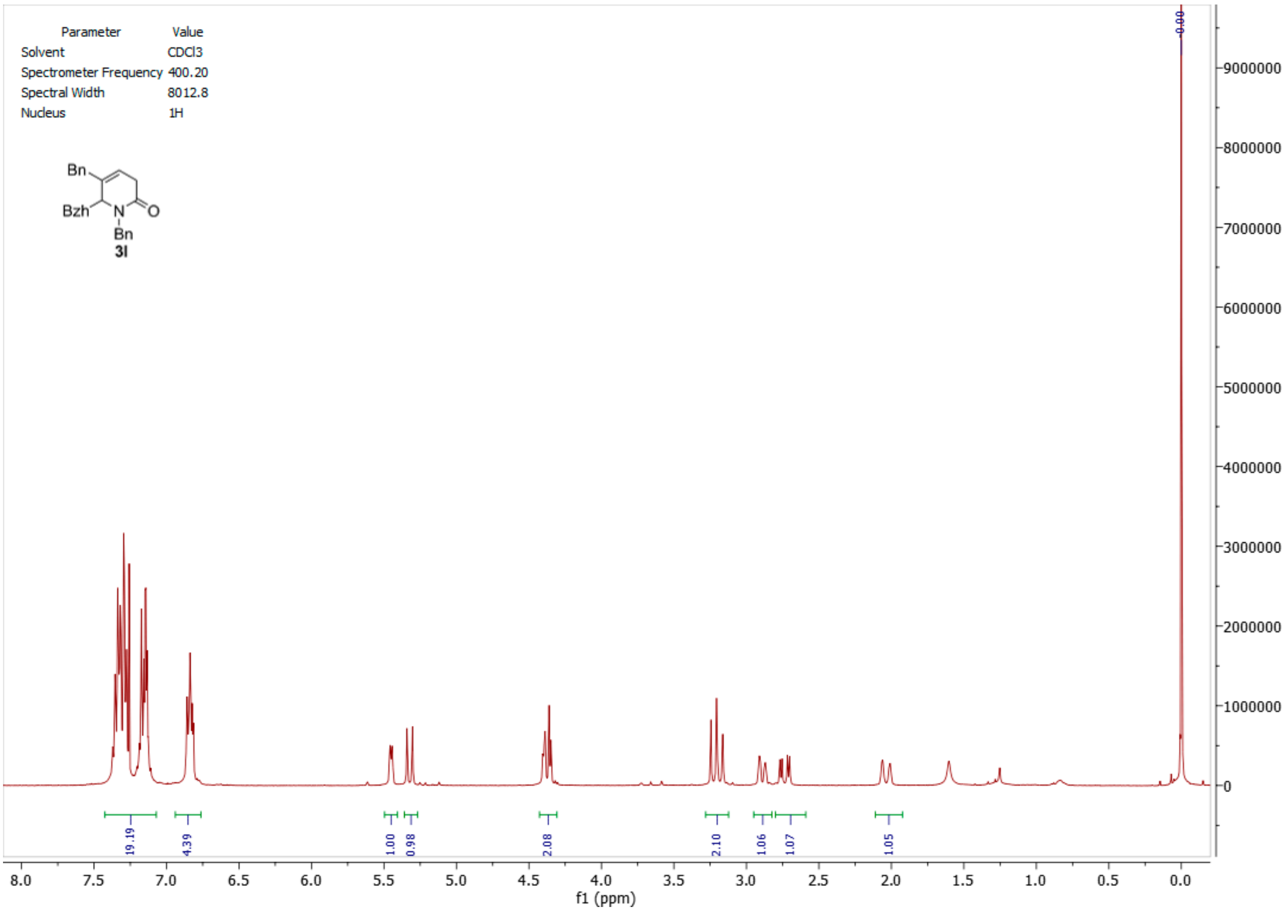

Parameter Value
Solvent CDCl3
Spectrometer Frequency 400.20
Spectral Width 8012.8
Nucleus 1H
Bn
Bzh
N
O
Bn
3l
f1 (ppm)

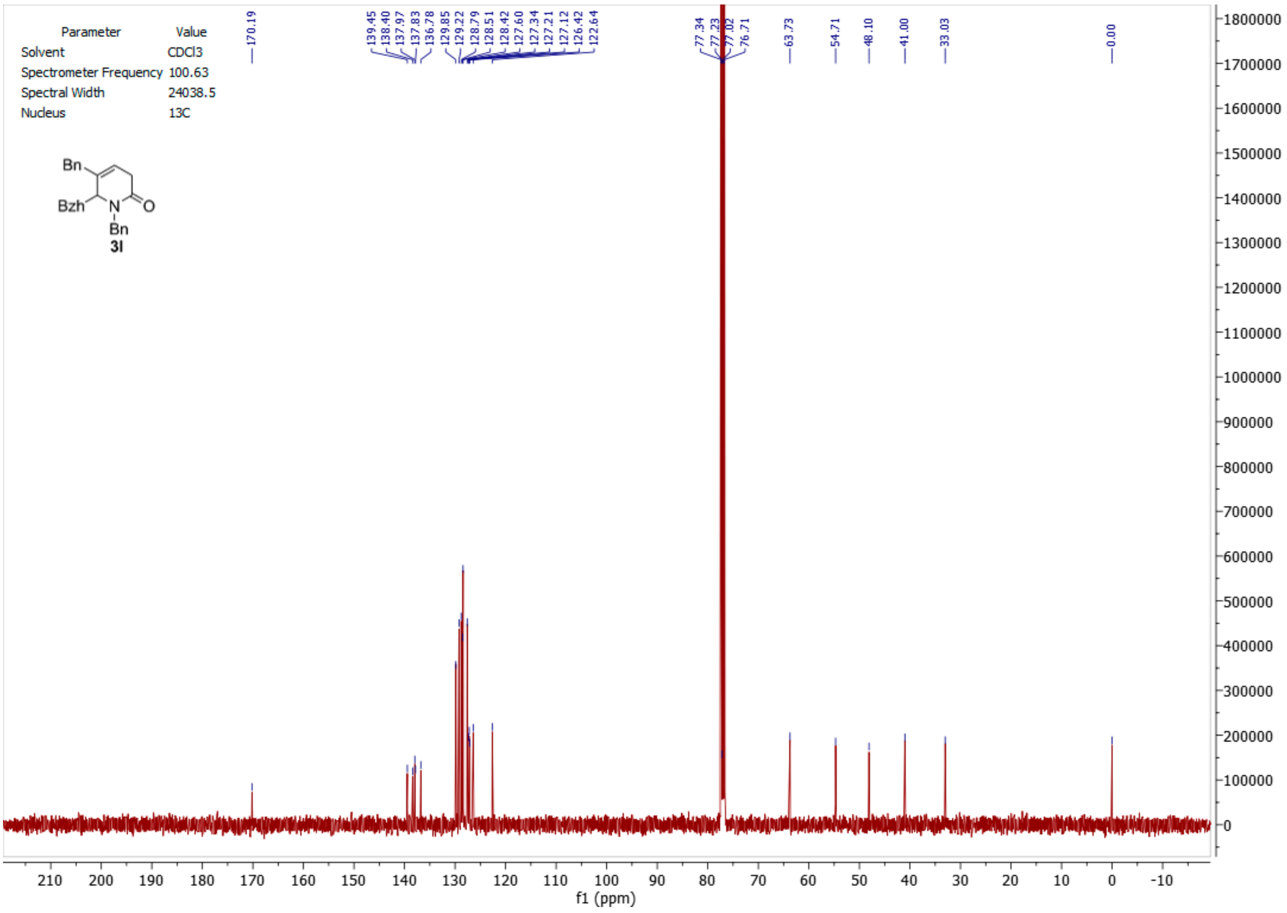

Parameter Value
Solvent CDCl3
Spectrometer Frequency 100.63
Spectral Width 24038.5
Nucleus 13C
Bn
Bzh
N
O
Bn
3l
f1 (ppm)

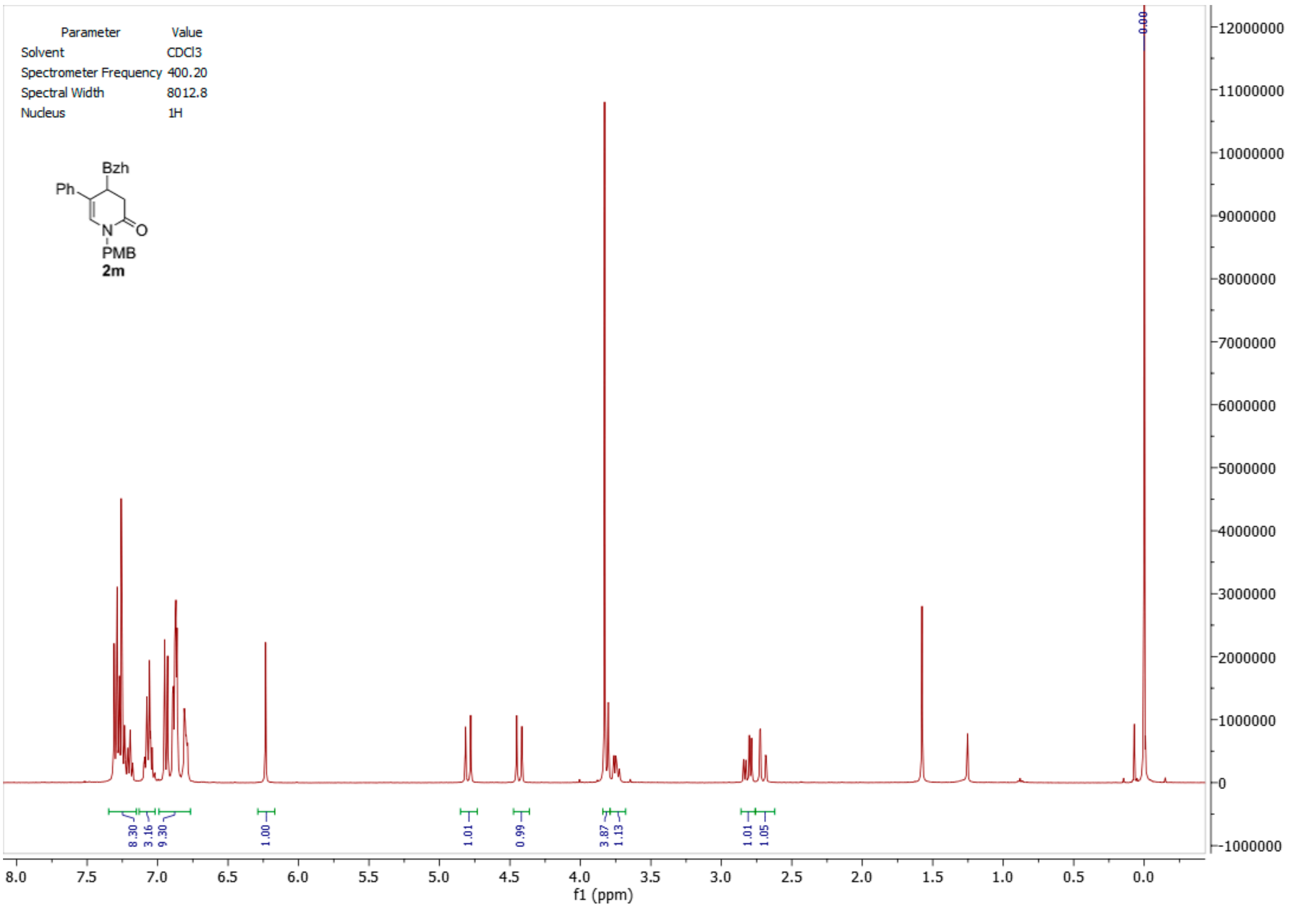
Parameter Value
Solvent CDCl3
Spectrometer Frequency 400.20
Spectral Width 8012.8
Nucleus 1H
Bzh
Ph
N
O
PMB
2m
8.30
3.16
9.30
1.00
1.01
0.99
3.87
1.13
1.01
1.05
0.00
f1 (ppm)

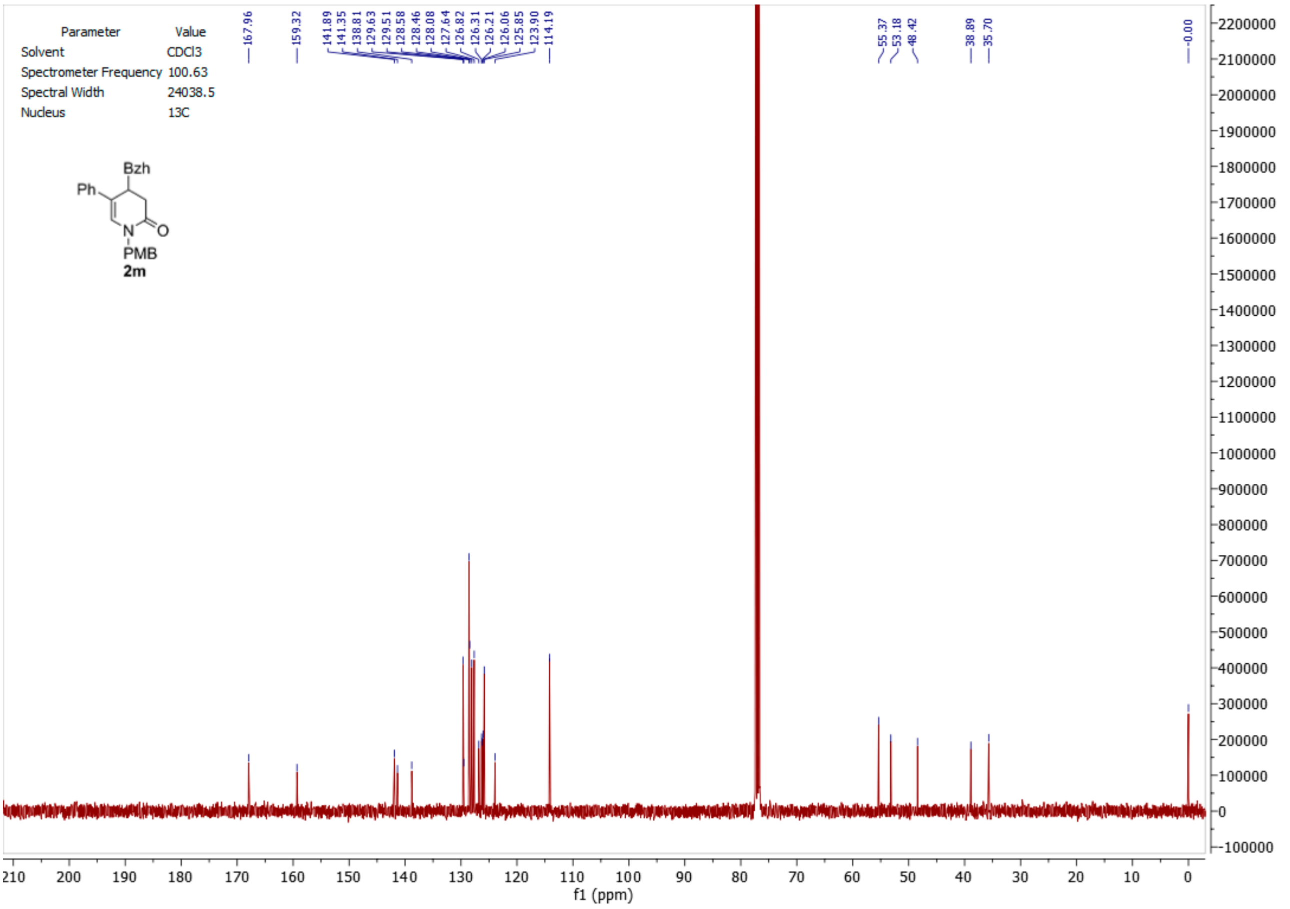
Parameter Value
Solvent CDCl3
Spectrometer Frequency 100.63
Spectral Width 24038.5
Nucleus 13C
Bzh
Ph
N
O
PMB
2m
167.96
159.32
141.89
141.35
138.81
129.63
129.51
128.58
128.46
128.08
127.64
126.82
126.31
126.21
126.06
125.85
123.90
114.19
55.37
53.18
48.42
38.89
35.70
-0.00
f1 (ppm)

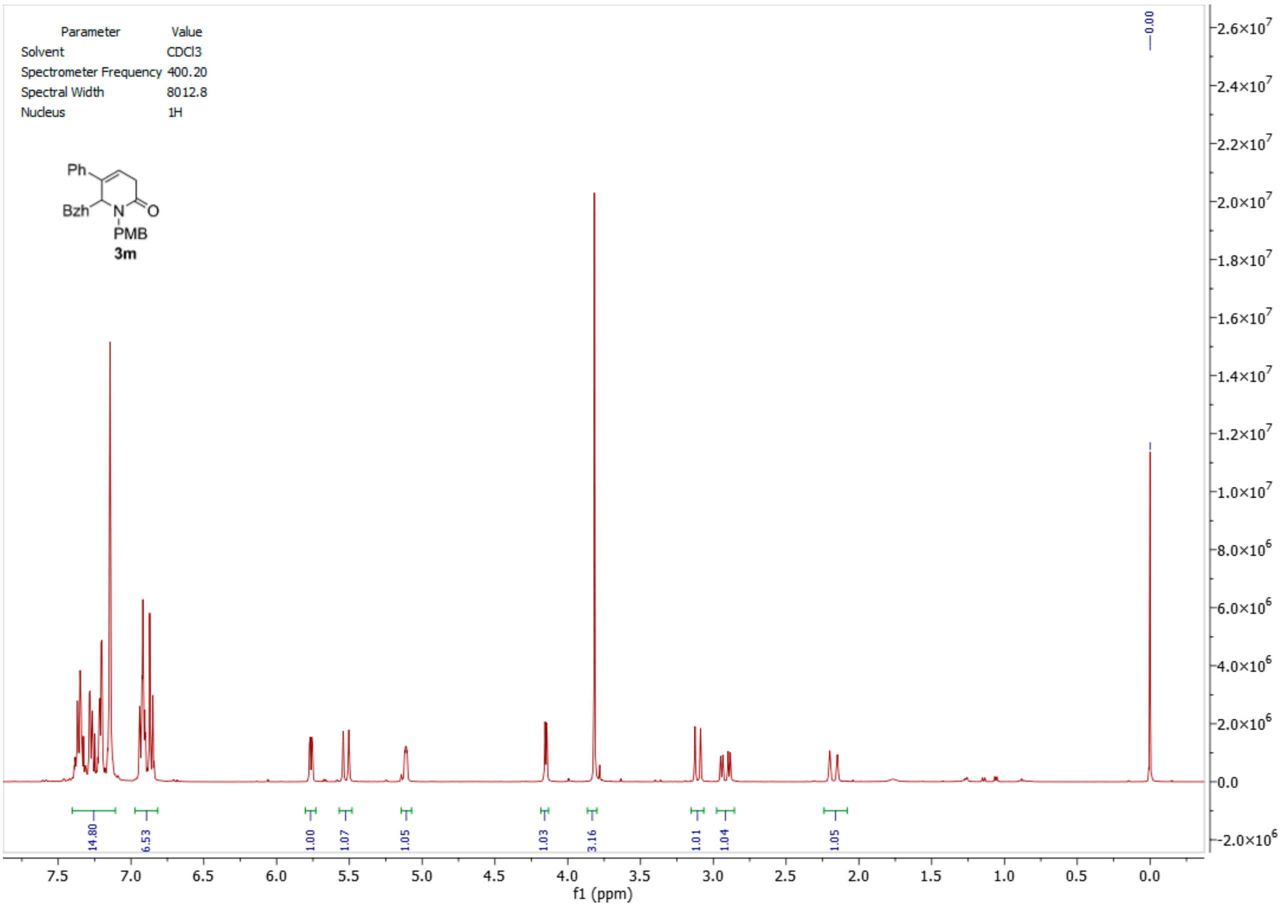
Parameter Value
Solvent CDCl3
Spectrometer Frequency 400.20
Spectral Width 8012.8
Nucleus 1H
Ph
Bzh
N
O
PMB
3m
f1 (ppm)

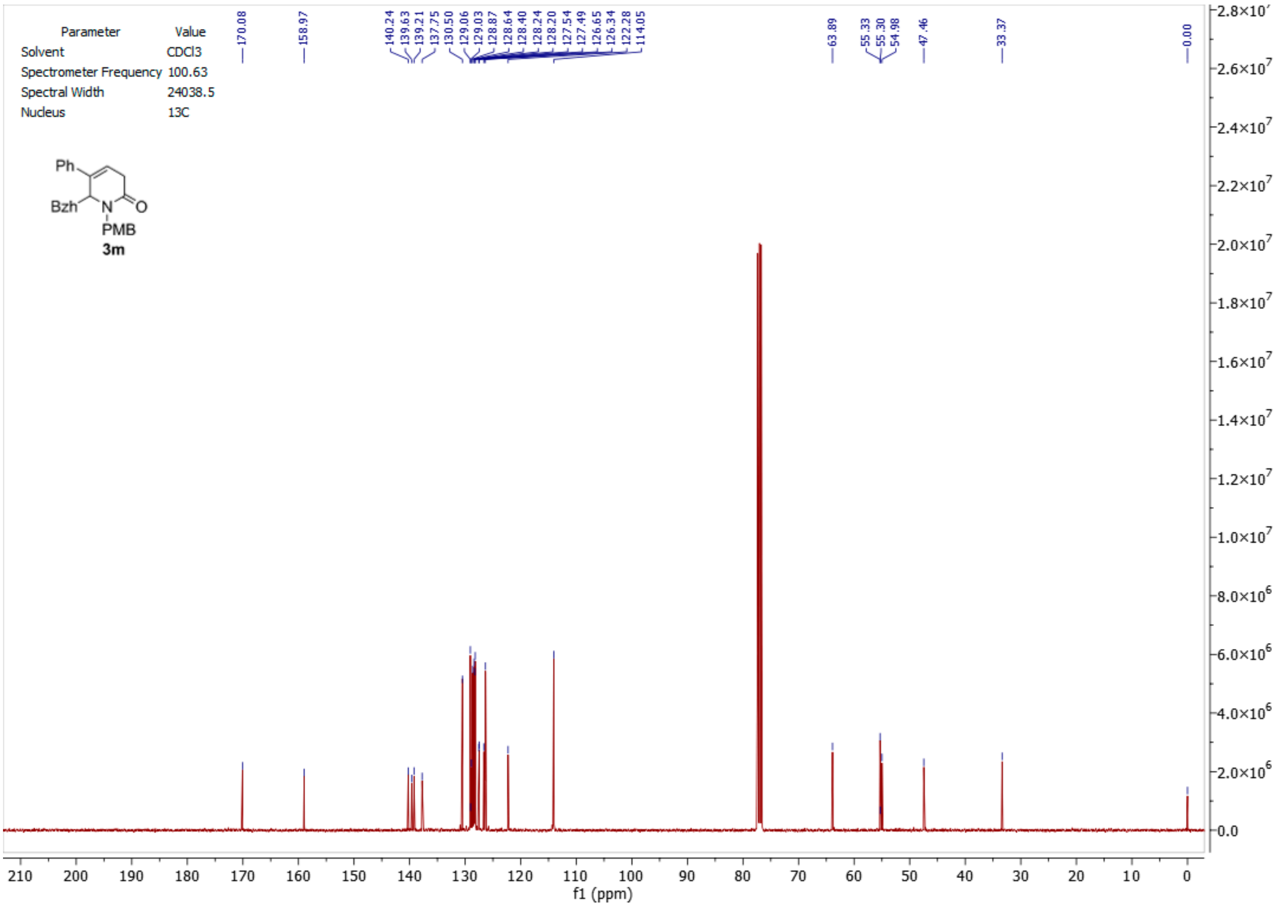
Parameter Value
Solvent CDCl3
Spectrometer Frequency 100.63
Spectral Width 24038.5
Nucleus 13C
Ph
Bzh
N
O
PMB
3m
170.08
158.97
140.24
139.63
139.21
137.75
130.50
129.06
129.03
128.87
128.64
128.40
128.24
128.20
127.54
127.49
126.65
126.34
122.28
114.05
63.89
55.33
55.30
54.98
47.46
33.37
0.00
f1 (ppm)

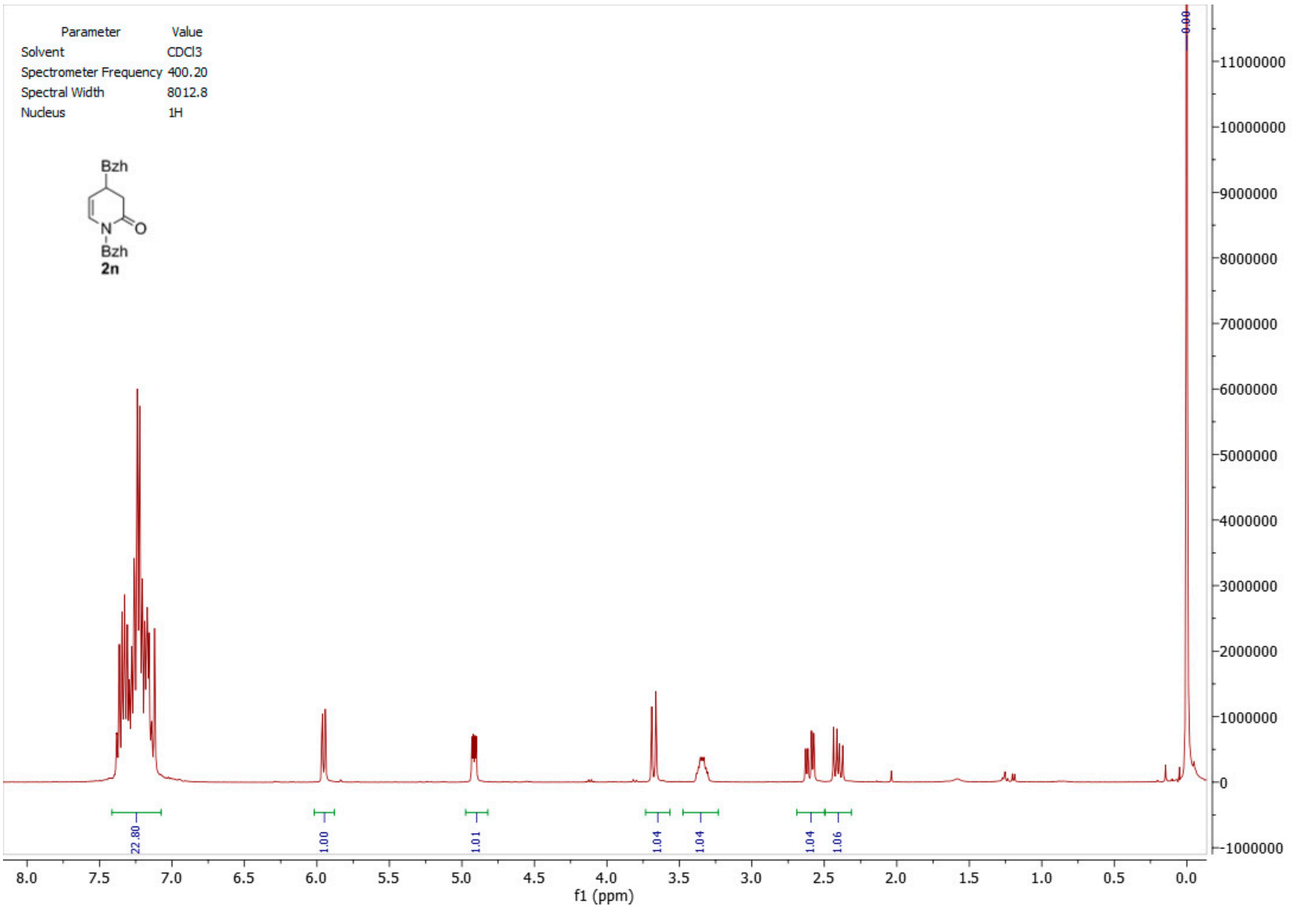
Parameter Value
Solvent CDCl3
Spectrometer Frequency 400.20
Spectral Width 8012.8
Nucleus 1H
Bzh
N
O
Bzh
2n
22.80
1.00
1.01
1.04
1.04
1.04
1.06
f1 (ppm)

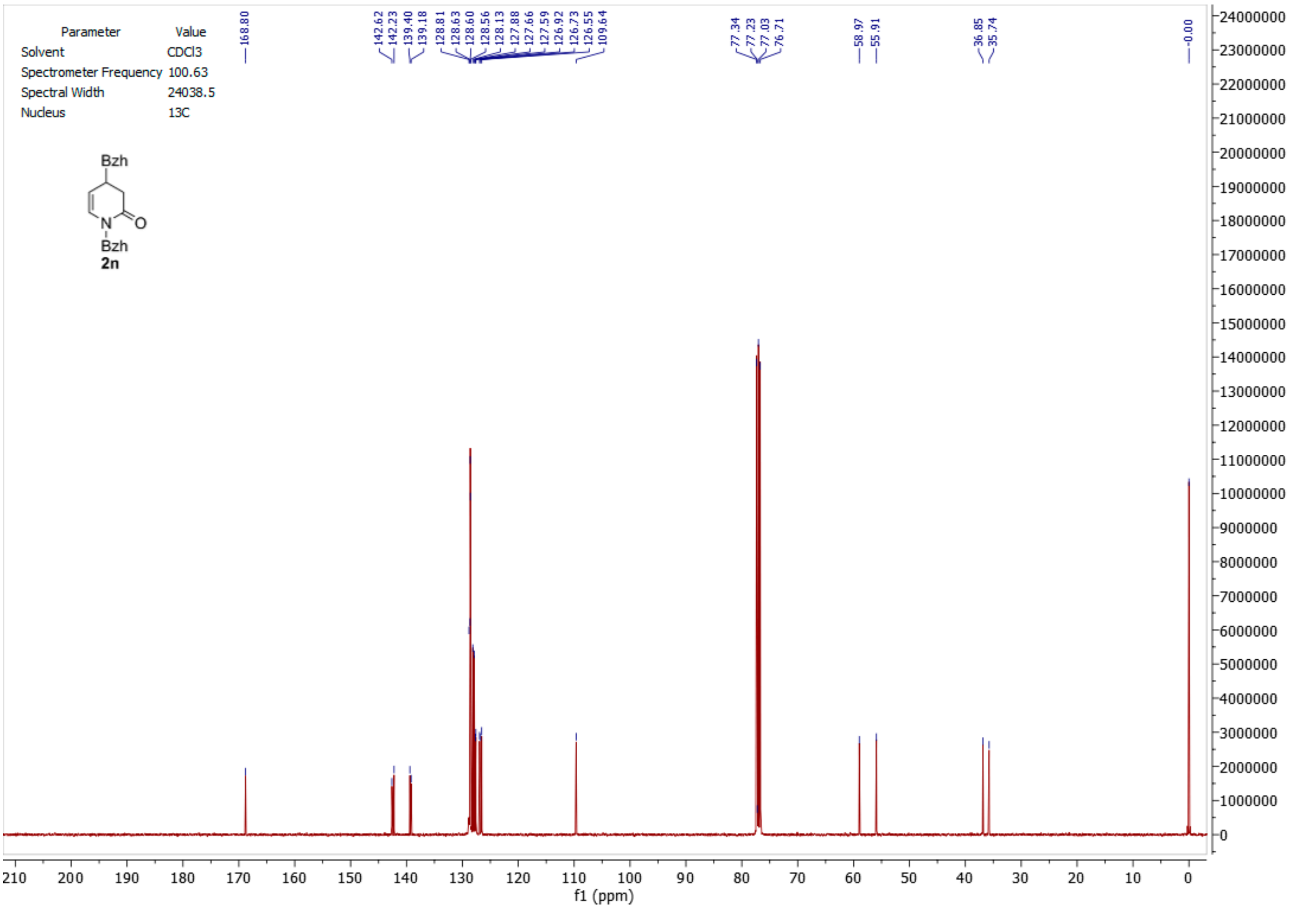
Parameter Value
Solvent CDCl3
Spectrometer Frequency 100.63
Spectral Width 24038.5
Nucleus 13C
Bzh
N
O
Bzh
2n
168.80
142.62
142.23
139.40
139.18
128.81
128.63
128.60
128.56
128.13
127.88
127.66
127.59
126.92
126.73
126.55
109.64
77.34
77.23
77.03
76.71
58.97
55.91
36.85
35.74
0.00
f1 (ppm)

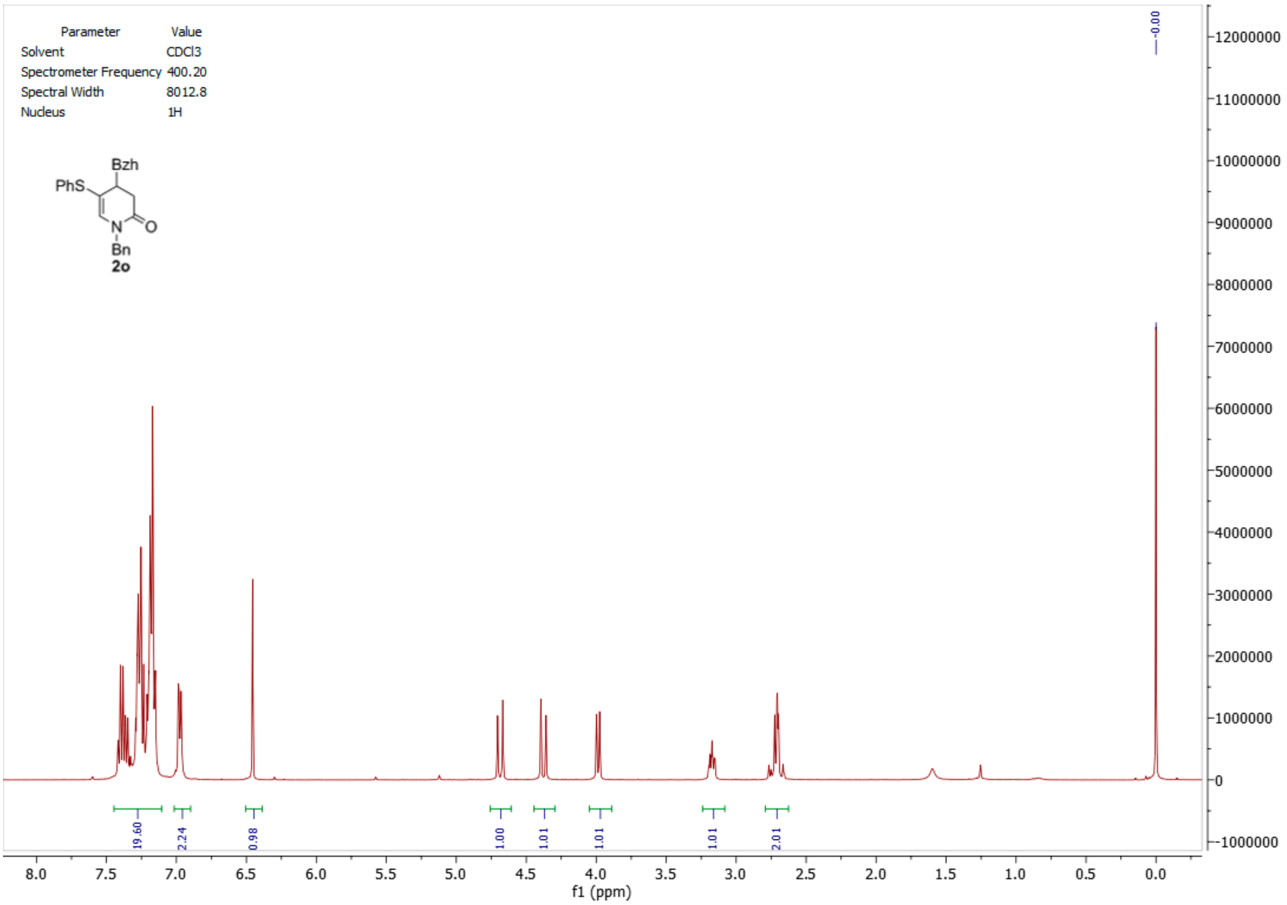

Parameter Value
Solvent CDCl3
Spectrometer Frequency 400.20
Spectral Width 8012.8
Nucleus 1H
Bzh
PhS
N
O
Bn
2o
f1 (ppm)

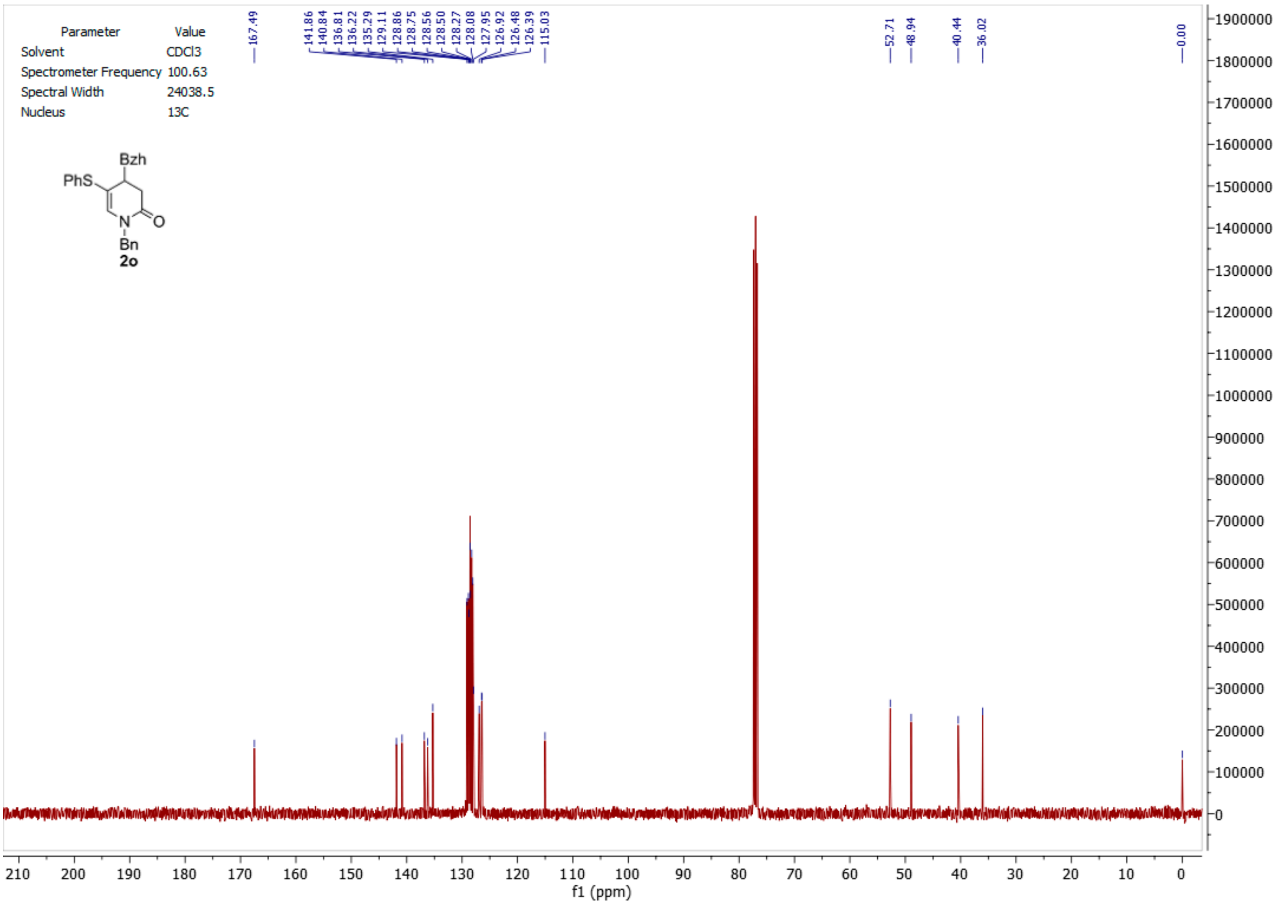

Parameter Value
Solvent CDCl3
Spectrometer Frequency 100.63
Spectral Width 24038.5
Nucleus 13C
Bzh
PhS
N
O
Bn
2o
167.49
141.86
140.84
136.81
136.22
135.29
129.11
128.86
128.75
128.56
128.50
128.27
128.08
127.95
126.92
126.48
126.39
115.03
52.71
48.94
40.44
36.02
0.00
f1 (ppm)

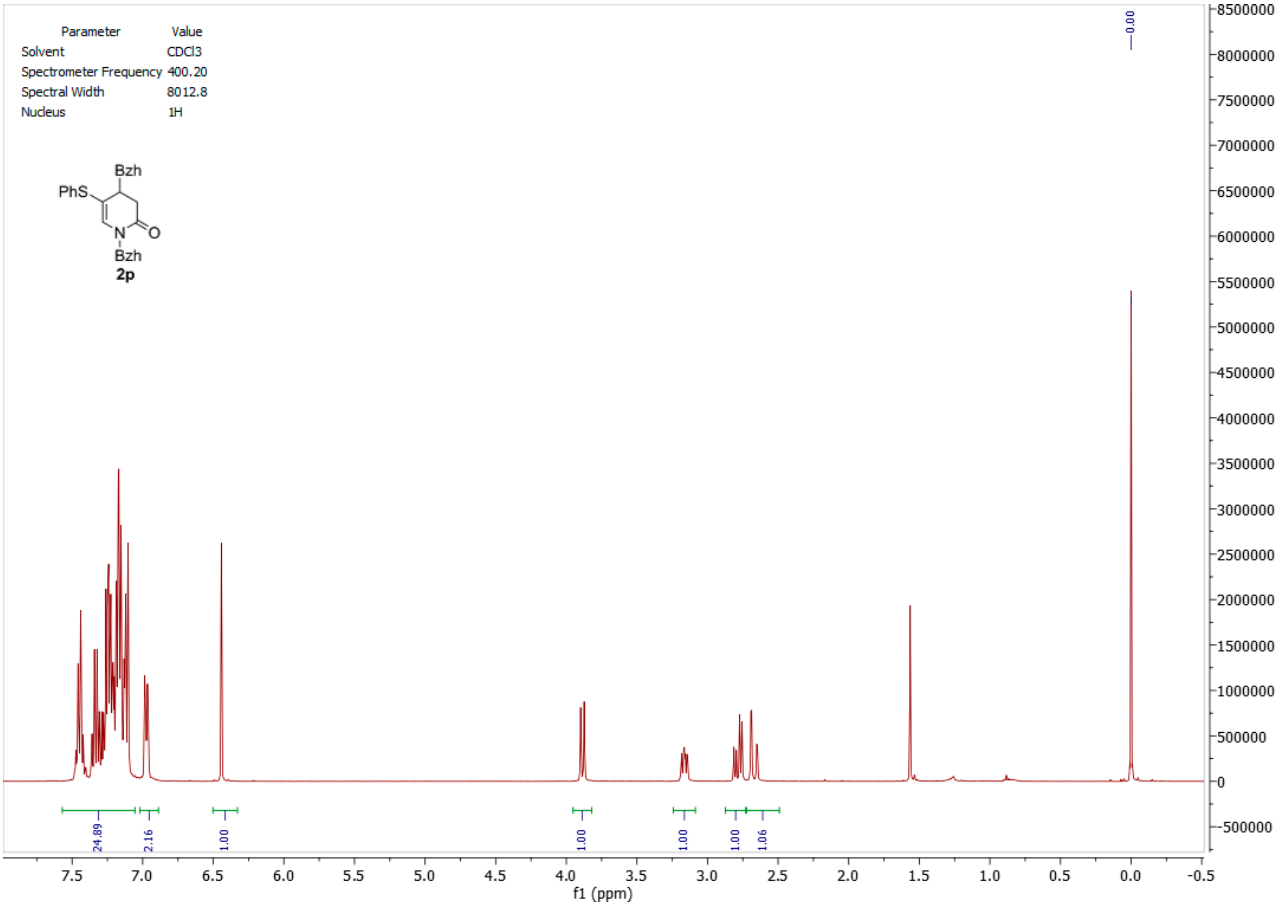

Parameter Value
Solvent CDCl3
Spectrometer Frequency 400.20
Spectral Width 8012.8
Nucleus 1H
Bzh
PhS
N
O
Bzh
2p
0.00
24.89
2.16
1.00
1.00
1.00
1.00
1.06
f1 (ppm)

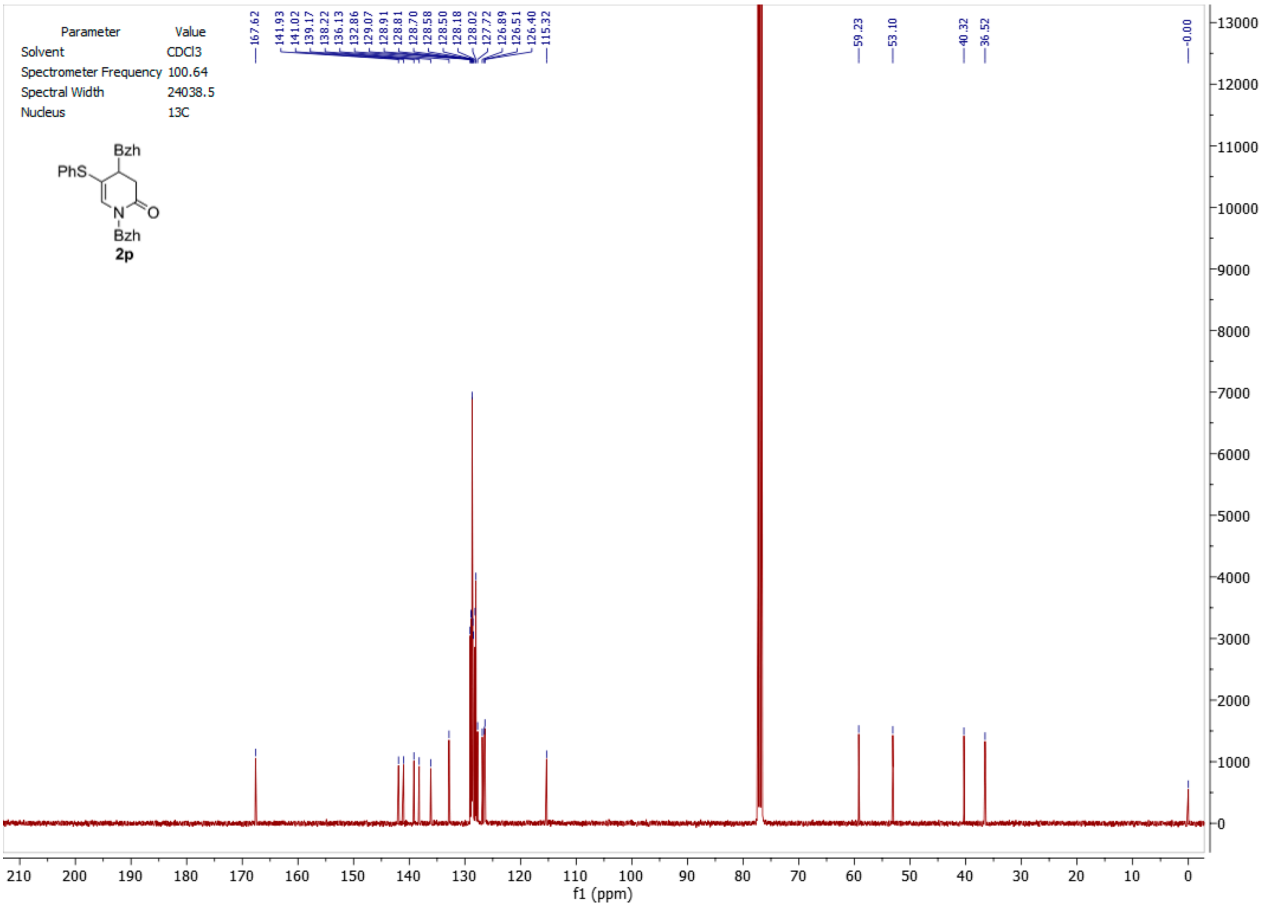

Parameter Value
Solvent CDCl3
Spectrometer Frequency 100.64
Spectral Width 24038.5
Nucleus 13C
Bzh
PhS
N
O
Bzh
2p
167.62
141.93
141.02
139.17
138.22
136.13
132.86
129.07
128.91
128.81
128.70
128.58
128.50
128.18
128.02
127.72
126.89
126.51
126.40
115.32
59.23
53.10
40.32
36.52
-0.00
f1 (ppm)

Parameter	Value
Solvent	CDCl3
Spectrometer Frequency	400.20
Spectral Width	8012.8
Nucleus	1H

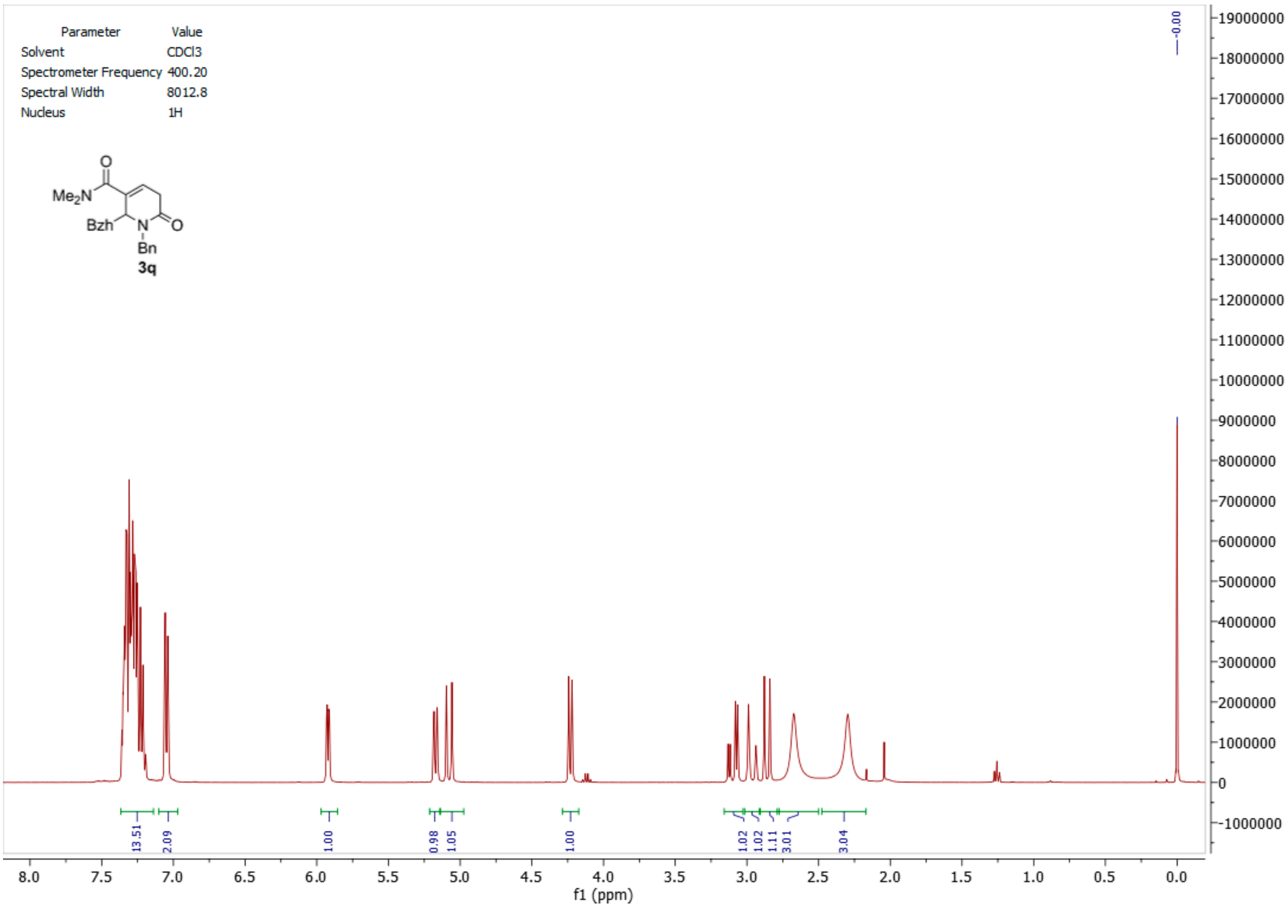

Parameter	Value
Solvent	CDCl3
Spectrometer Frequency	100.63
Spectral Width	24038.5
Nucleus	13C

* - solvent impurity

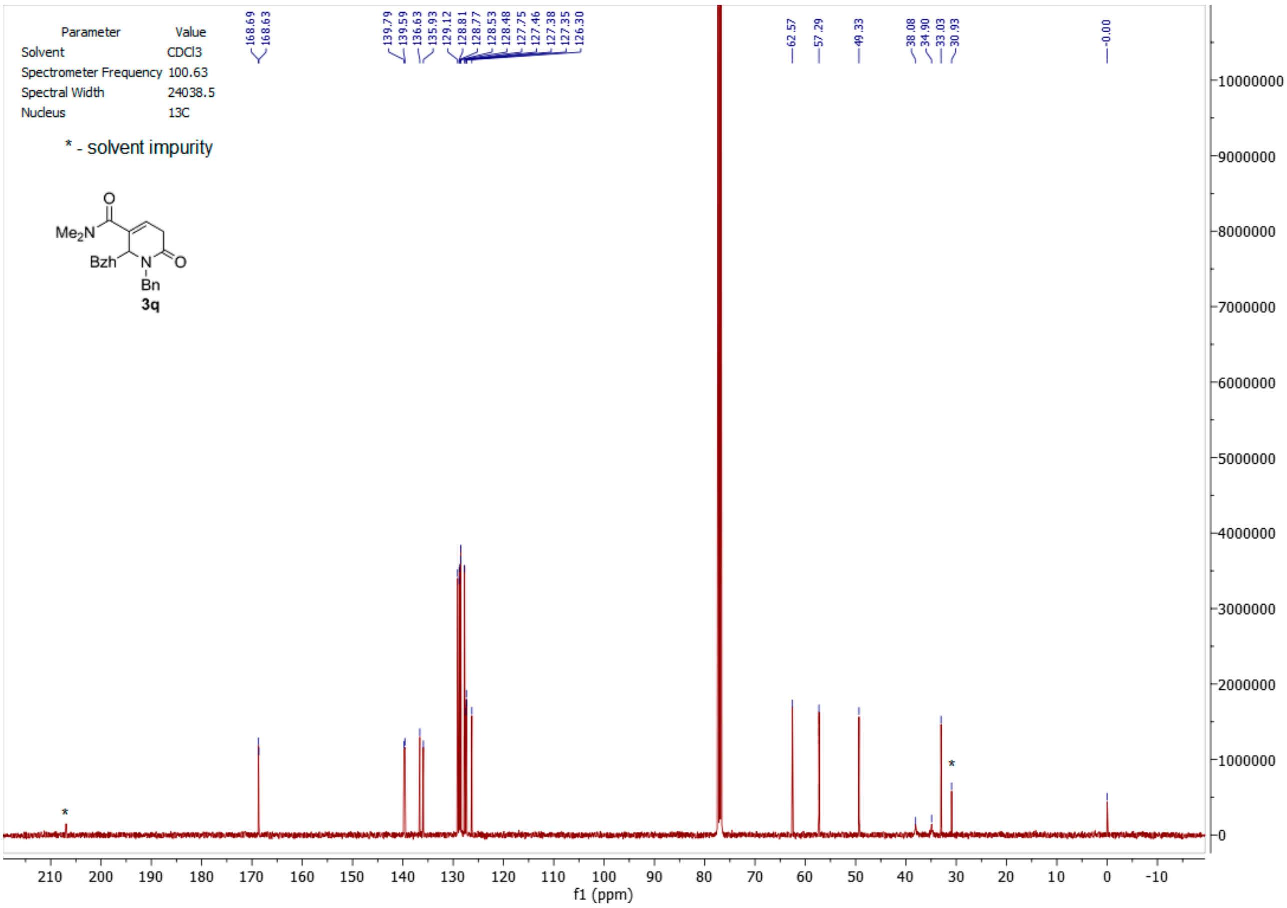

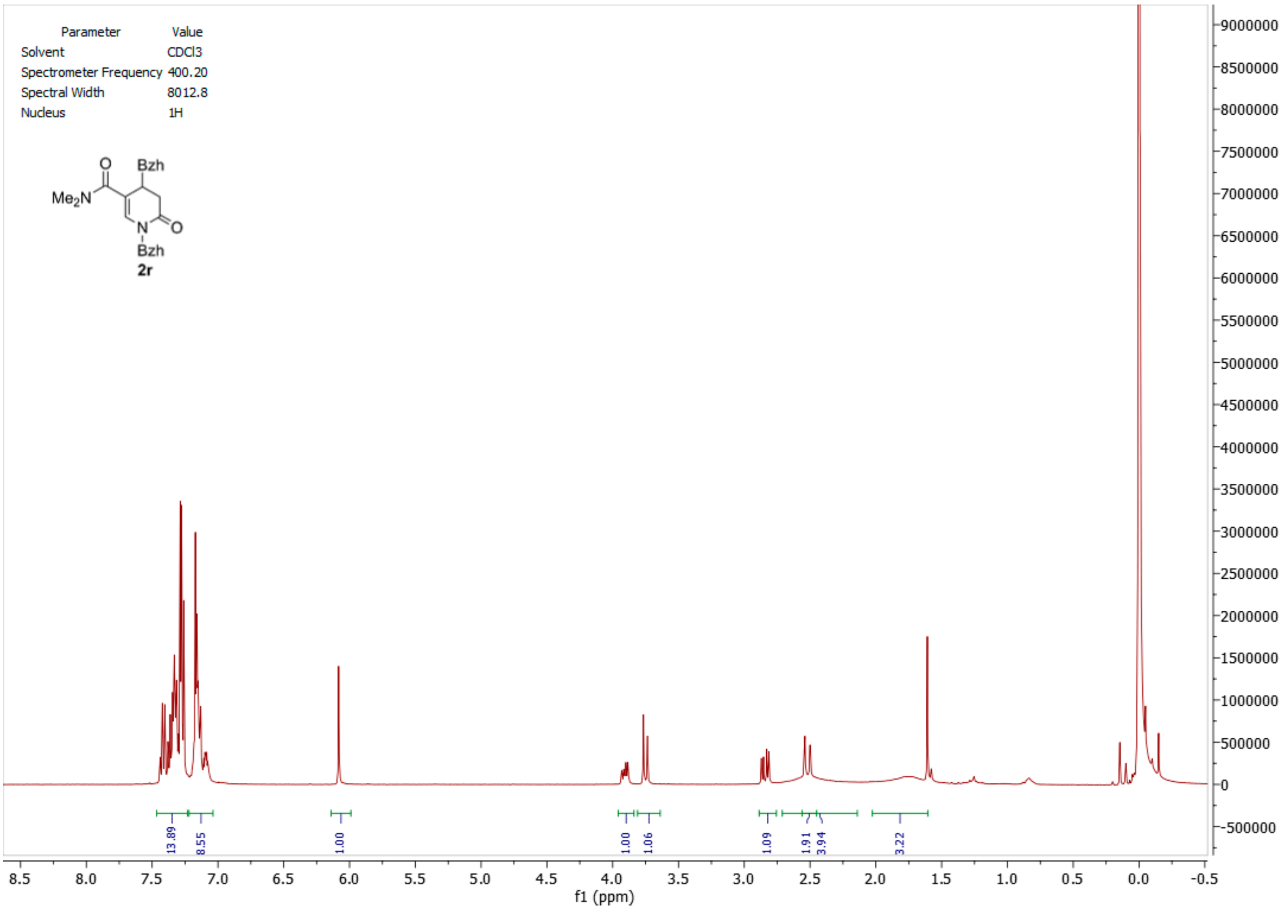
Parameter Value
Solvent CDCl3
Spectrometer Frequency 400.20
Spectral Width 8012.8
Nucleus 1H
Bzh
Me2N
O
N
Bzh
2r
13.89
8.55
1.00
1.00
1.06
1.09
1.91
3.94
3.22
f1 (ppm)

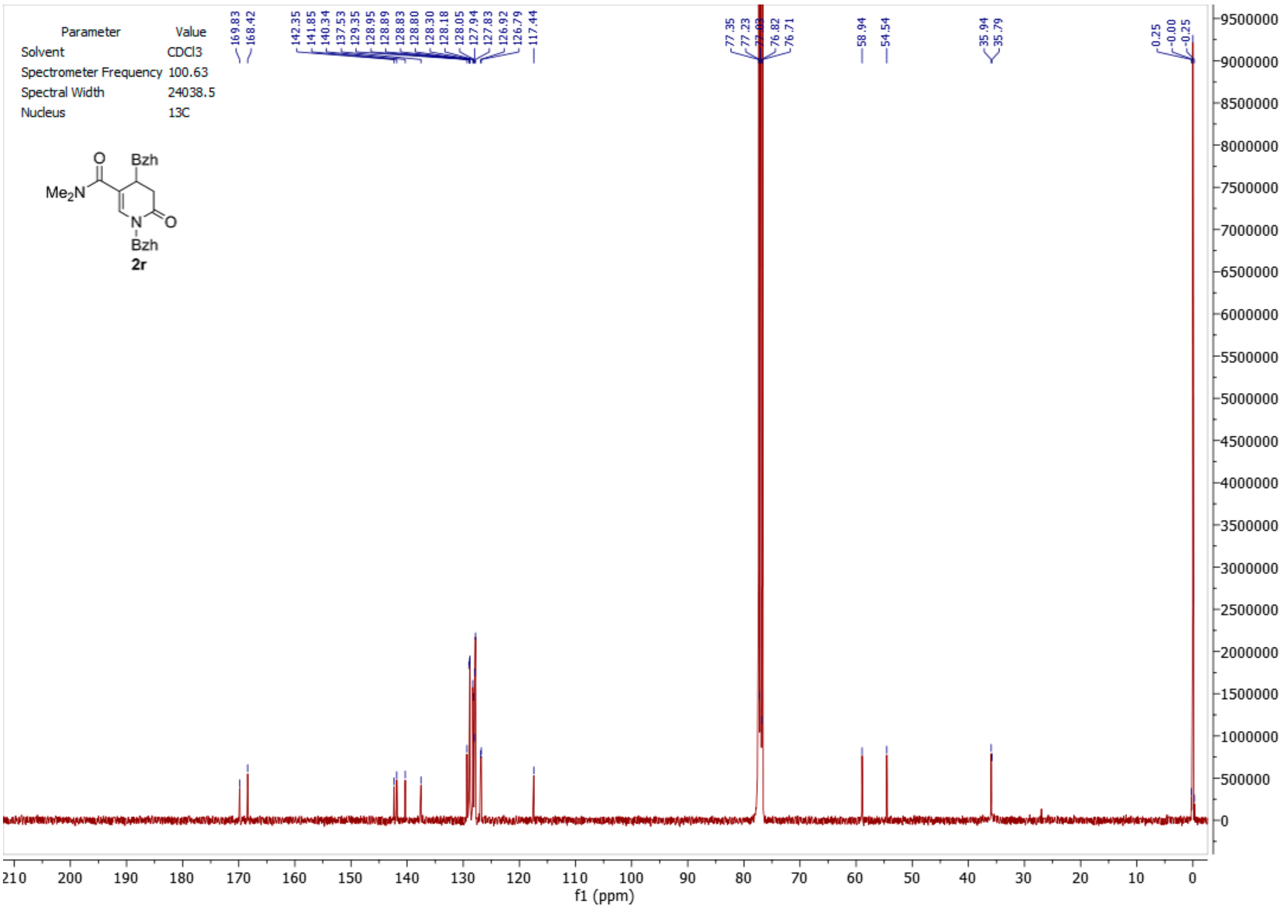
Parameter Value
Solvent CDCl3
Spectrometer Frequency 100.63
Spectral Width 24038.5
Nucleus 13C
Bzh
Me2N
O
N
Bzh
2r
169.83
168.42
142.35
141.85
140.34
137.53
129.35
128.95
128.89
128.83
128.80
128.30
128.18
128.05
127.94
127.83
126.92
126.79
117.44
77.35
77.23
76.82
76.71
58.94
54.54
35.94
35.79
0.25
-0.00
-0.25
f1 (ppm)

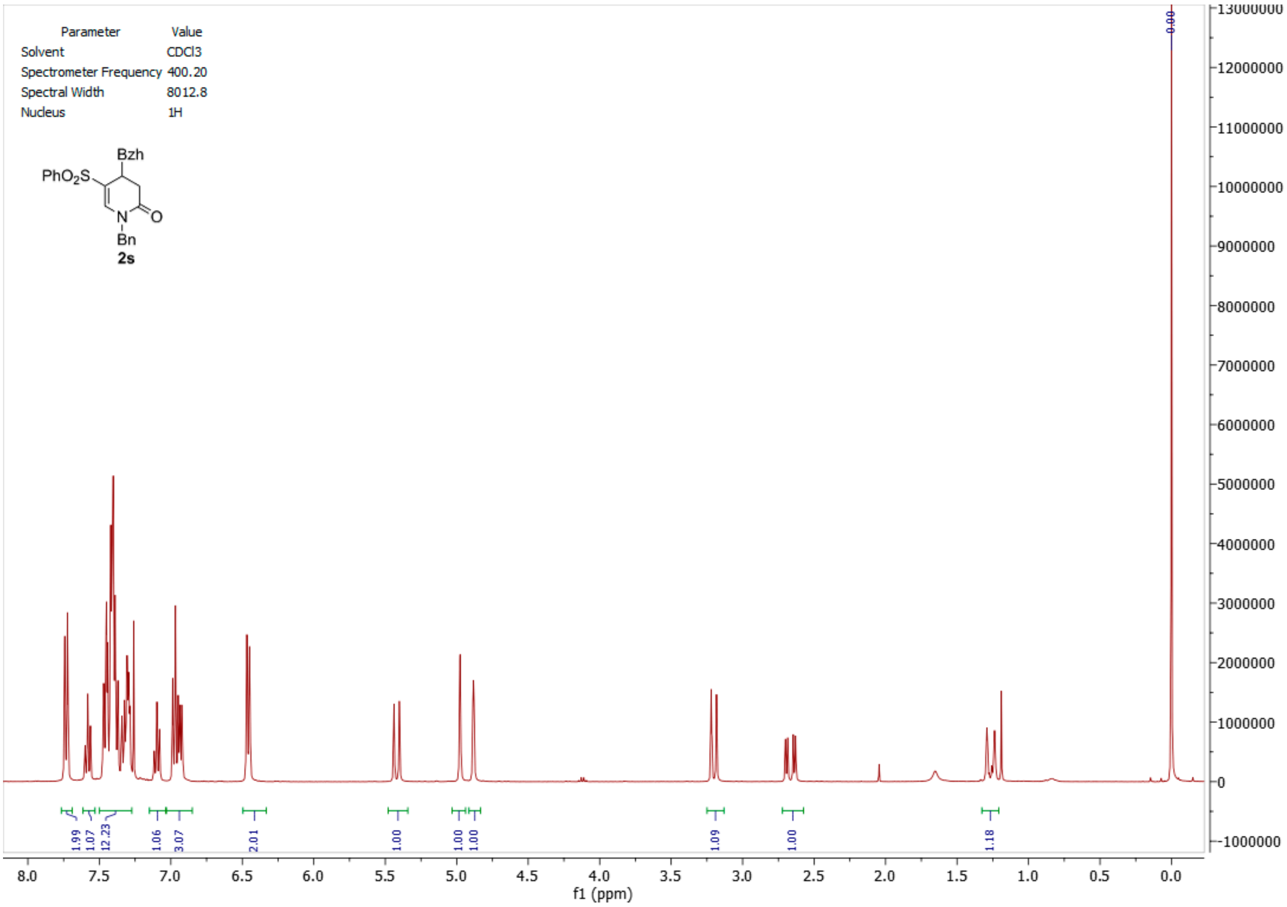
Parameter Value
Solvent CDCl3
Spectrometer Frequency 400.20
Spectral Width 8012.8
Nucleus 1H
Bzh
PhO2S
N
O
Bn
2s
f1 (ppm)

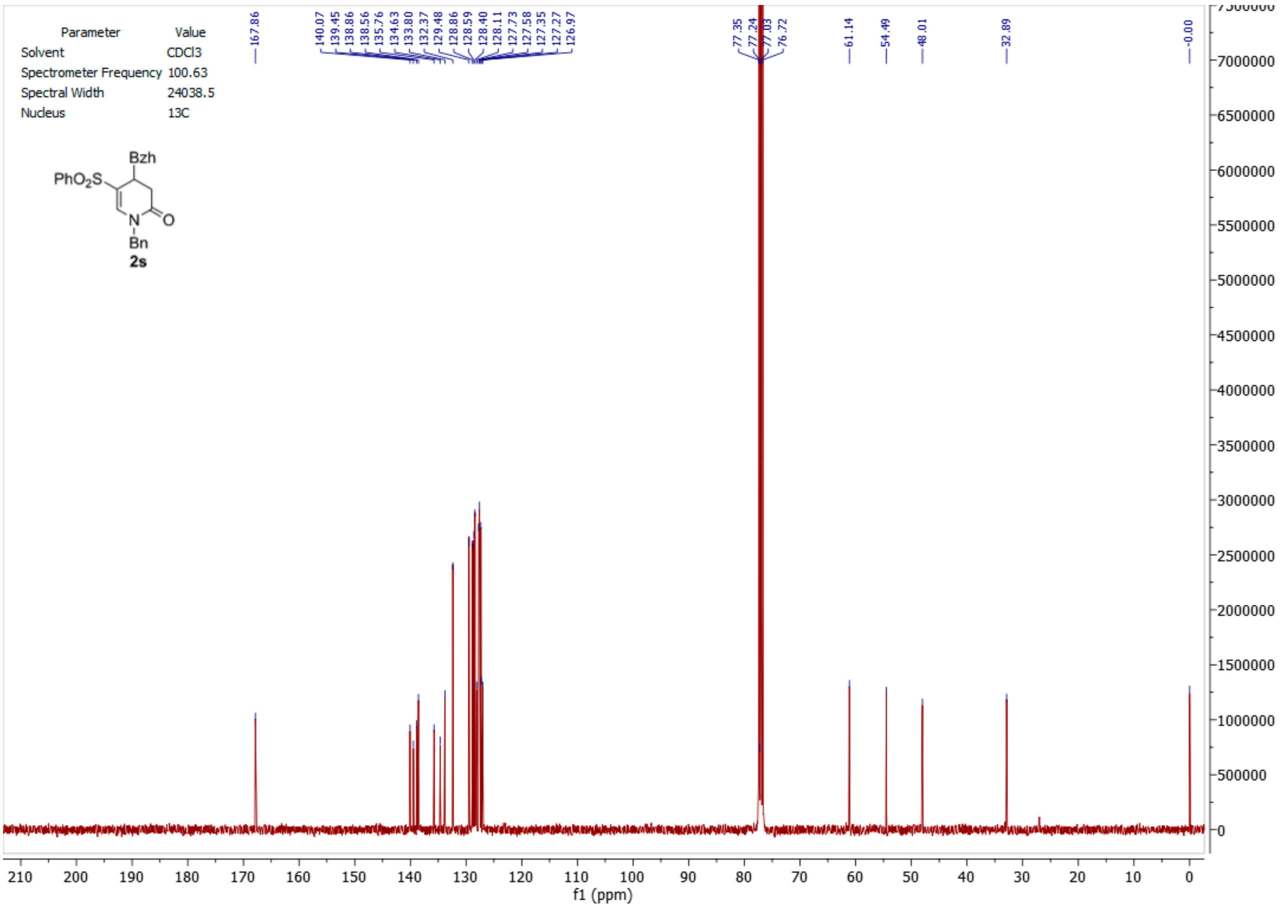
Parameter Value
Solvent CDCl3
Spectrometer Frequency 100.63
Spectral Width 24038.5
Nucleus 13C
Bzh
PhO2S
N
O
Bn
2s
f1 (ppm)

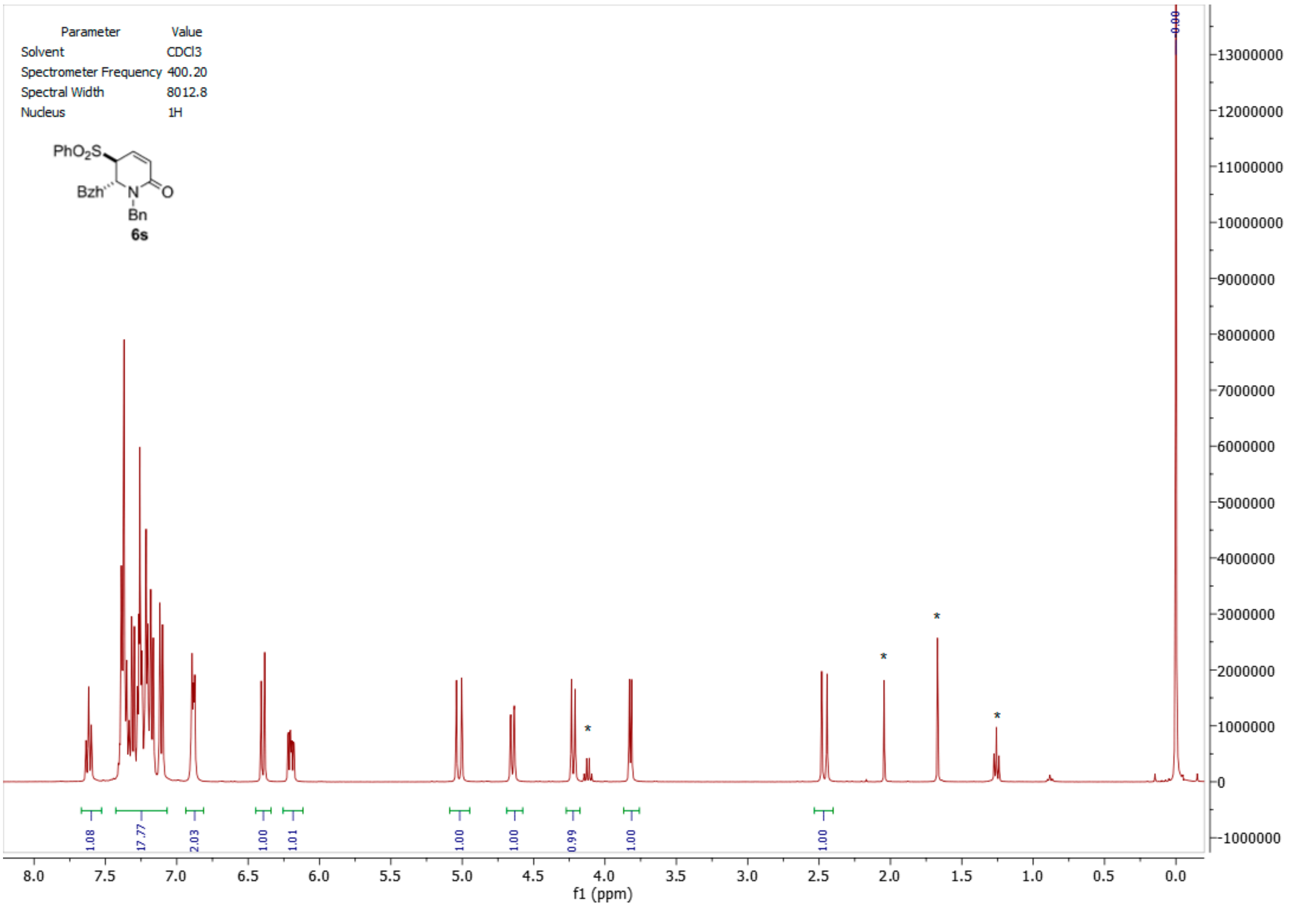

Parameter Value
Solvent CDCl3
Spectrometer Frequency 400.20
Spectral Width 8012.8
Nucleus 1H
PhO2S
Bzh
N
O
Bn
6s
f1 (ppm)

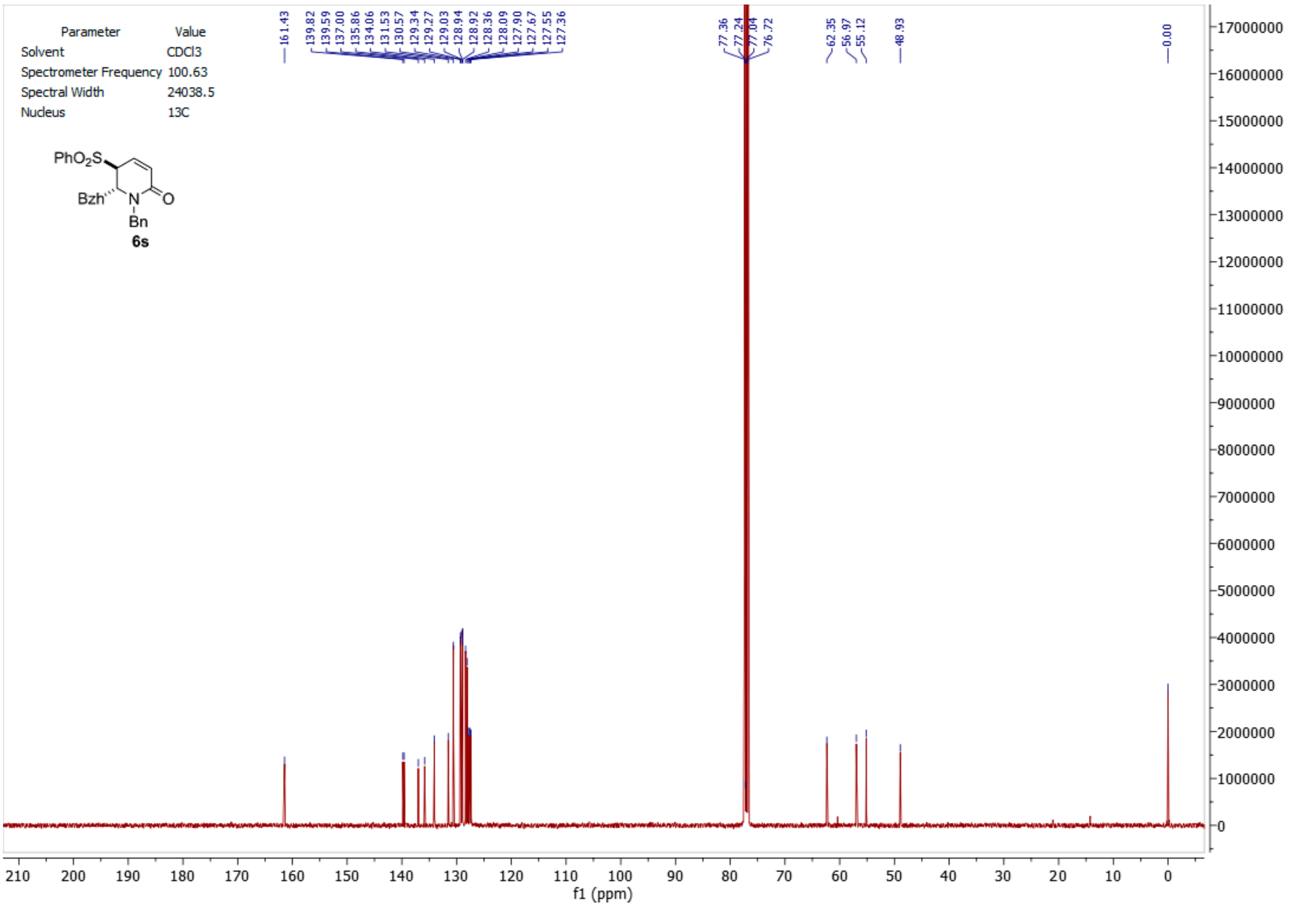

Parameter Value
Solvent CDCl3
Spectrometer Frequency 100.63
Spectral Width 24038.5
Nucleus 13C
PhO2S
Bzh
N
O
Bn
6s
161.43
139.82
139.59
137.00
135.86
134.06
131.53
130.57
129.34
129.27
129.03
128.94
128.92
128.36
128.09
127.90
127.67
127.55
127.36
77.36
77.24
77.04
76.72
62.35
56.97
55.12
48.93
0.00
f1 (ppm)

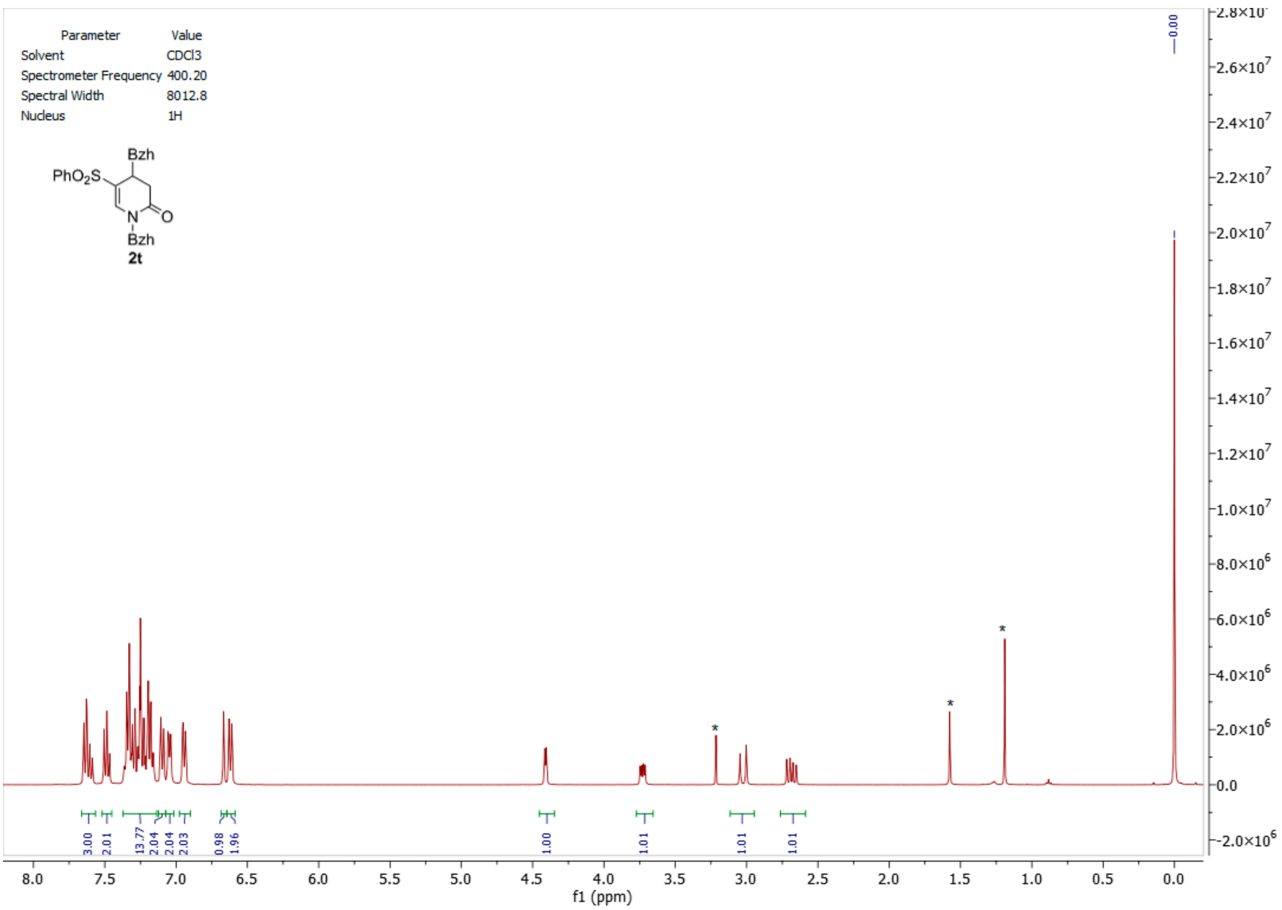
Parameter Value
Solvent CDCl3
Spectrometer Frequency 400.20
Spectral Width 8012.8
Nucleus 1H
Bzh
PhO2S
N
O
Bzh
2t
0.00
*
*
*
3.00
2.01
13.77
2.04
2.04
2.03
0.98
1.96
1.00
1.01
1.01
1.01
f1 (ppm)

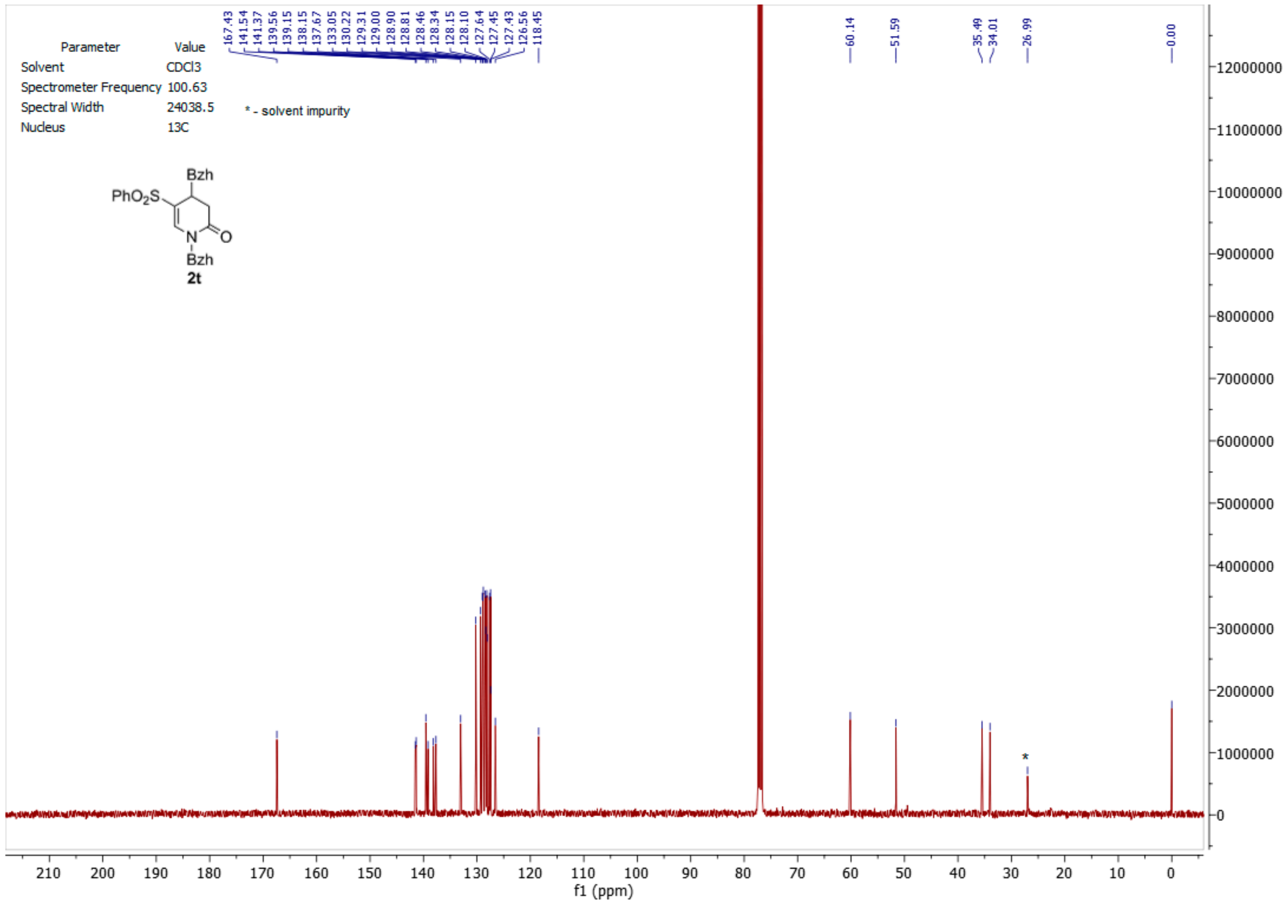
Parameter Value
Solvent CDCl3
Spectrometer Frequency 100.63
Spectral Width 24038.5
Nucleus 13C
* - solvent impurity
Bzh
PhO2S
N
O
Bzh
2t
167.43
141.54
141.37
139.56
139.15
138.15
137.67
133.05
130.22
129.31
129.00
128.90
128.81
128.46
128.34
128.15
128.10
127.64
127.45
127.43
126.56
118.45
60.14
51.59
35.49
34.01
26.99
0.00
*
f1 (ppm)

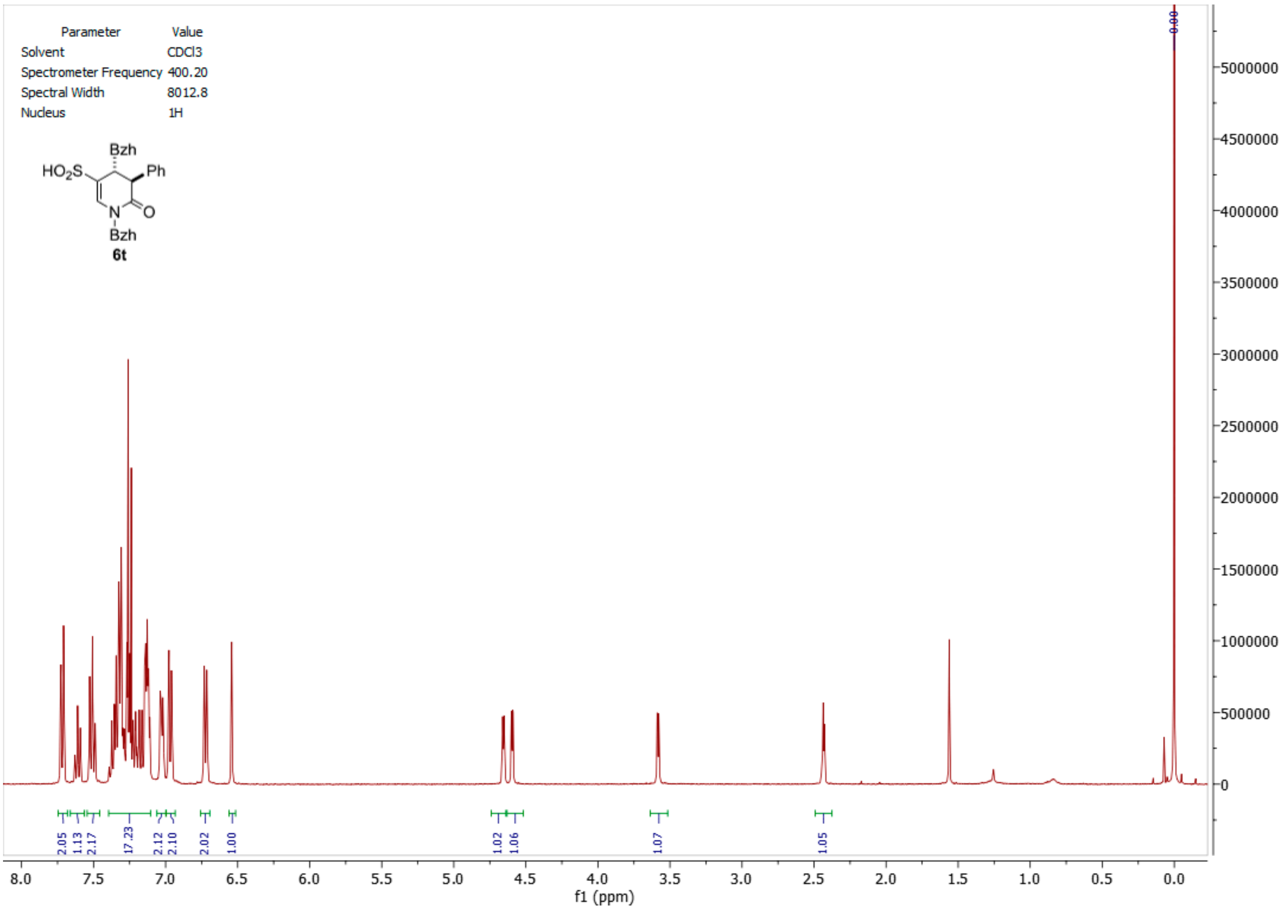

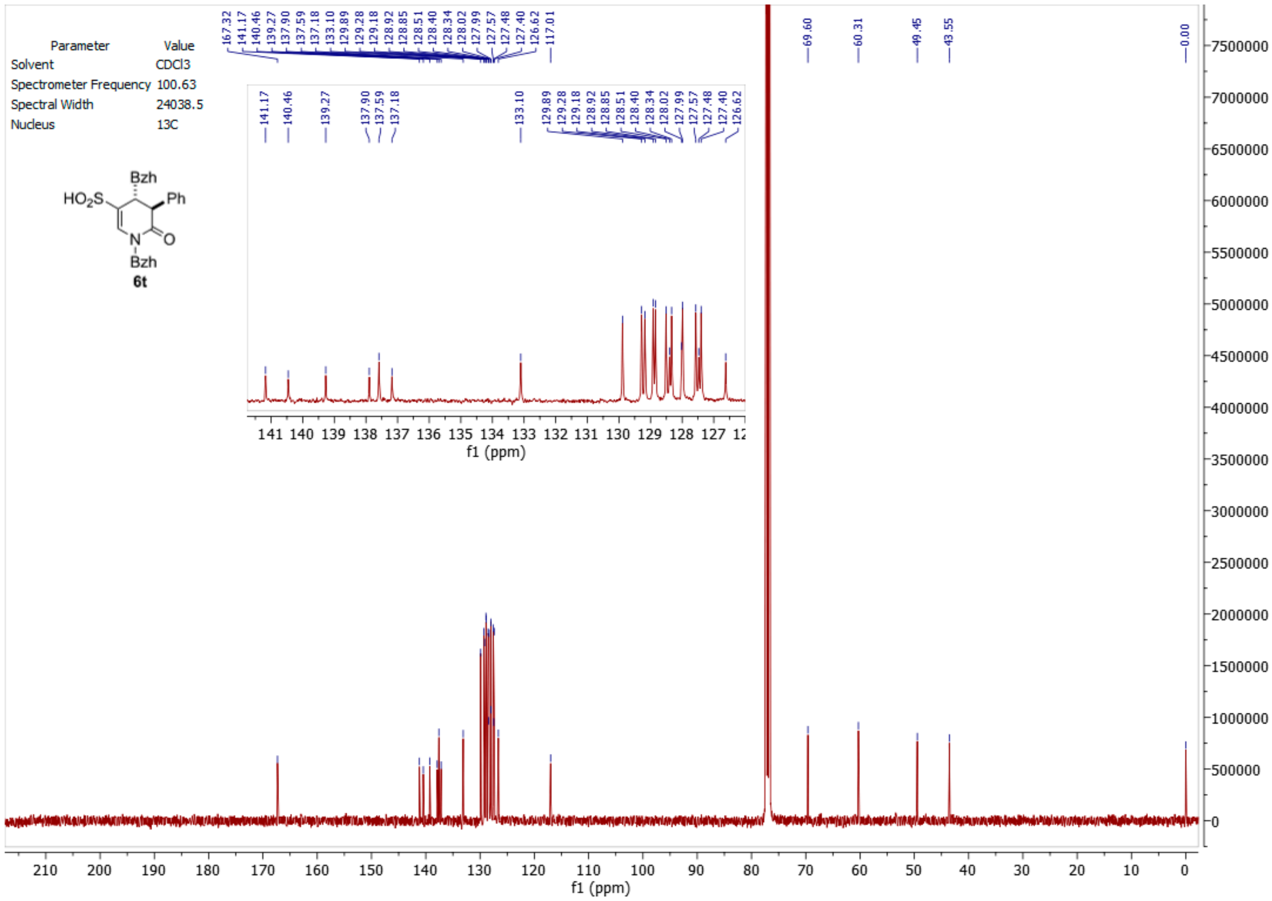

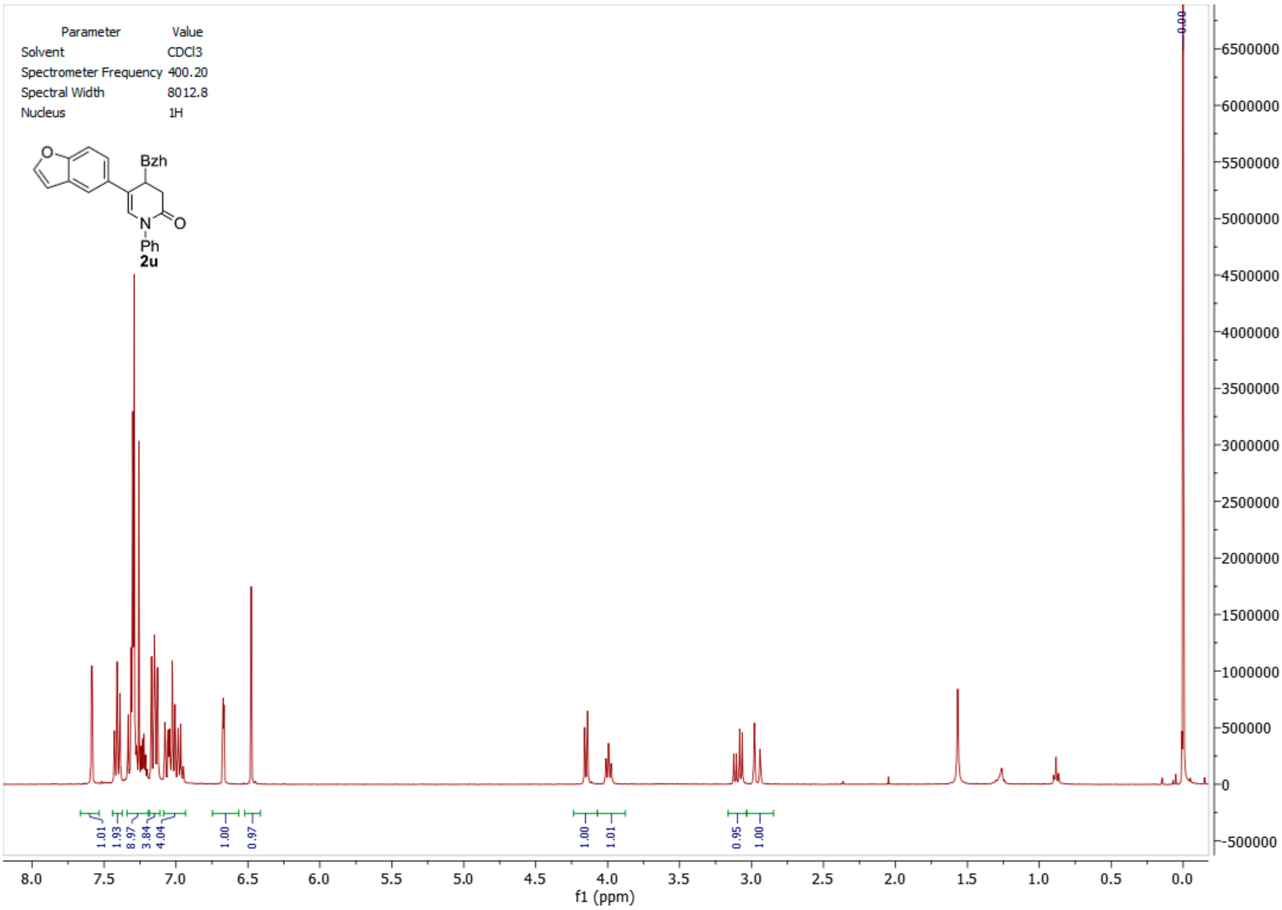
Parameter Value
Solvent CDCl3
Spectrometer Frequency 400.20
Spectral Width 8012.8
Nucleus 1H
Bzh
O
N
O
Ph
2u
f1 (ppm)

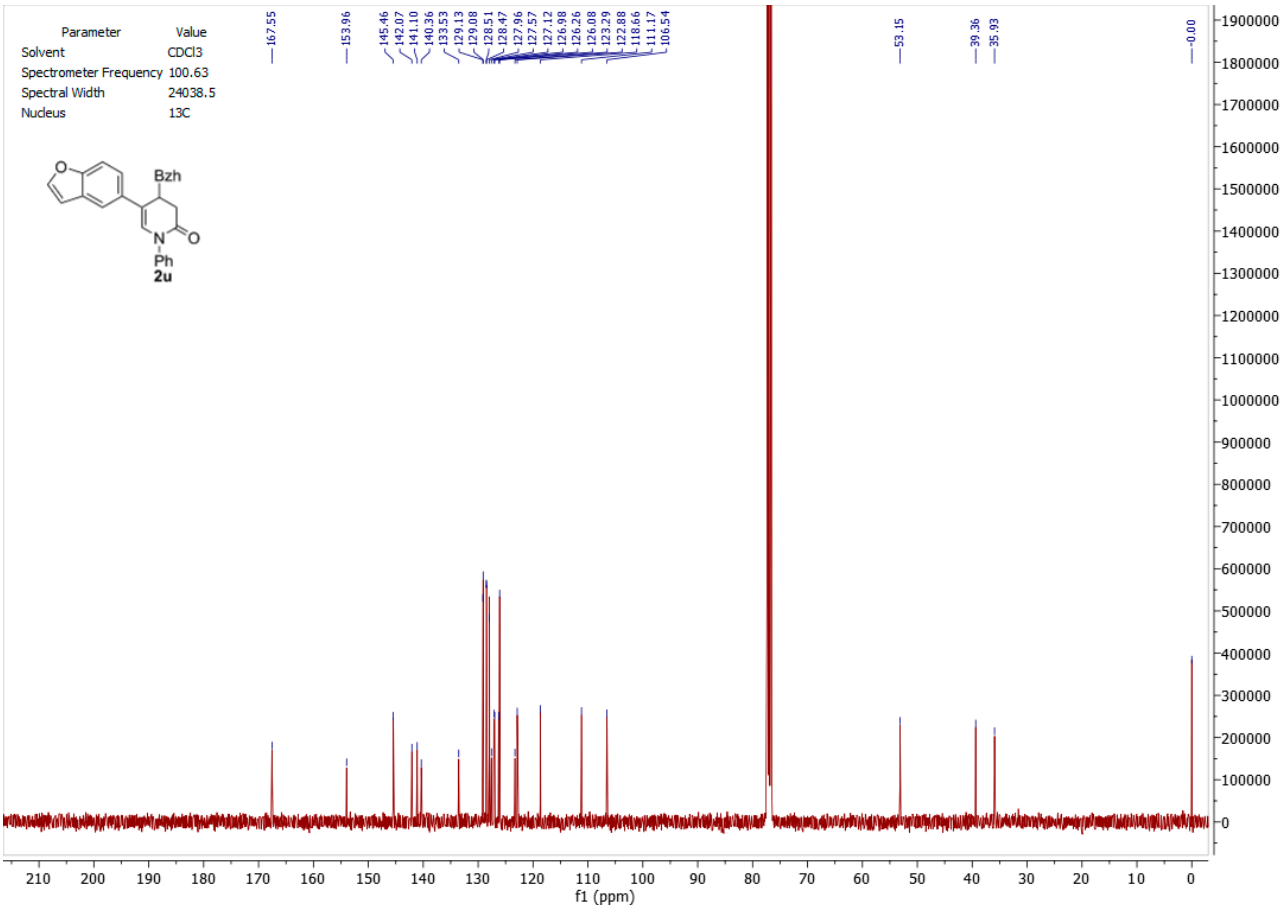
Parameter Value
Solvent CDCl3
Spectrometer Frequency 100.63
Spectral Width 24038.5
Nucleus 13C
Bzh
O
N
O
Ph
2u
167.55
153.96
145.46
142.07
141.10
140.36
133.53
129.13
129.08
128.51
128.47
127.96
127.57
127.12
126.98
126.26
126.08
123.29
122.88
118.66
111.17
106.54
53.15
39.36
35.93
-0.00
f1 (ppm)

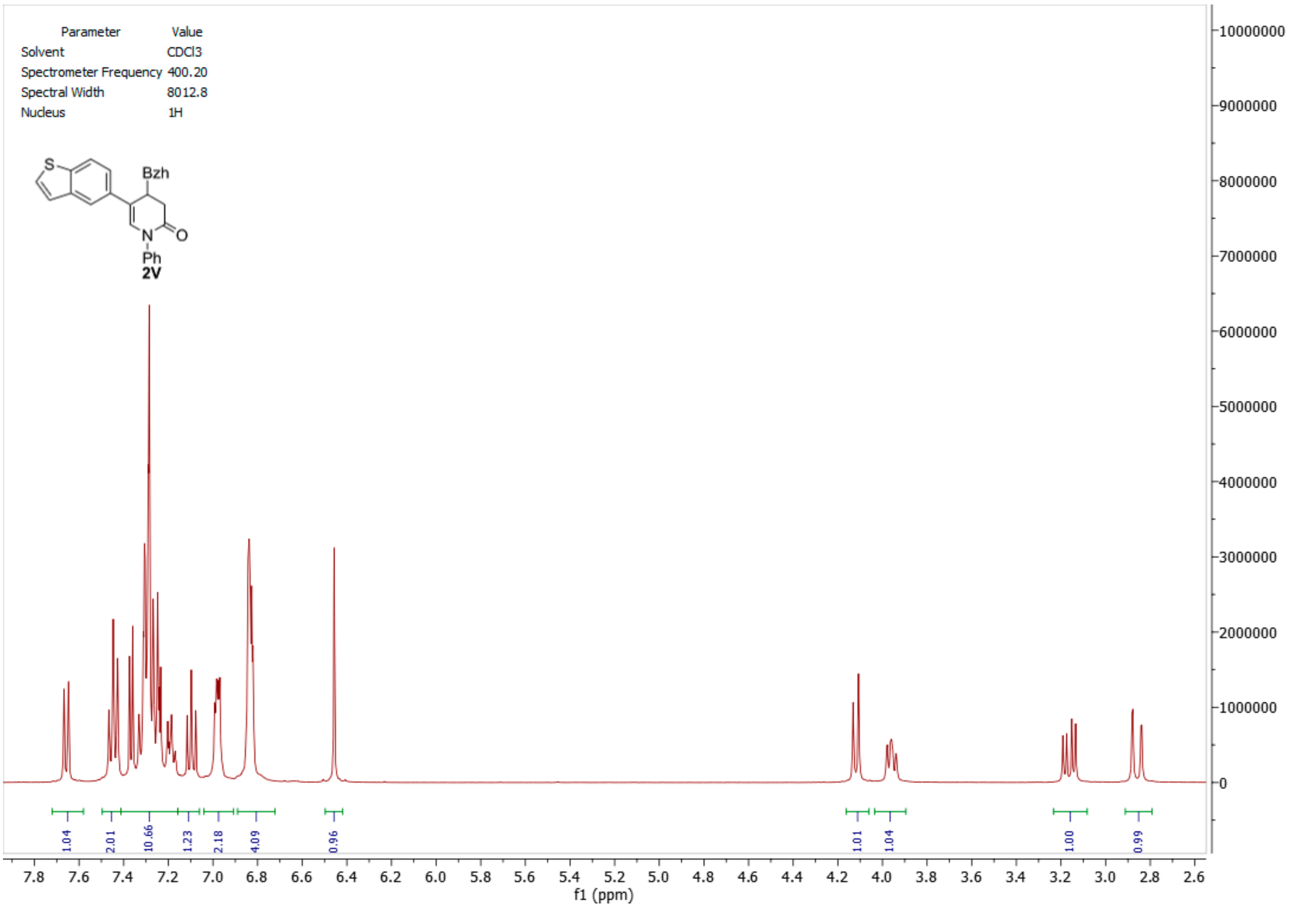

Parameter Value
Solvent CDCl3
Spectrometer Frequency 400.20
Spectral Width 8012.8
Nucleus 1H
Bzh
N
O
S
Ph
2V
f1 (ppm)

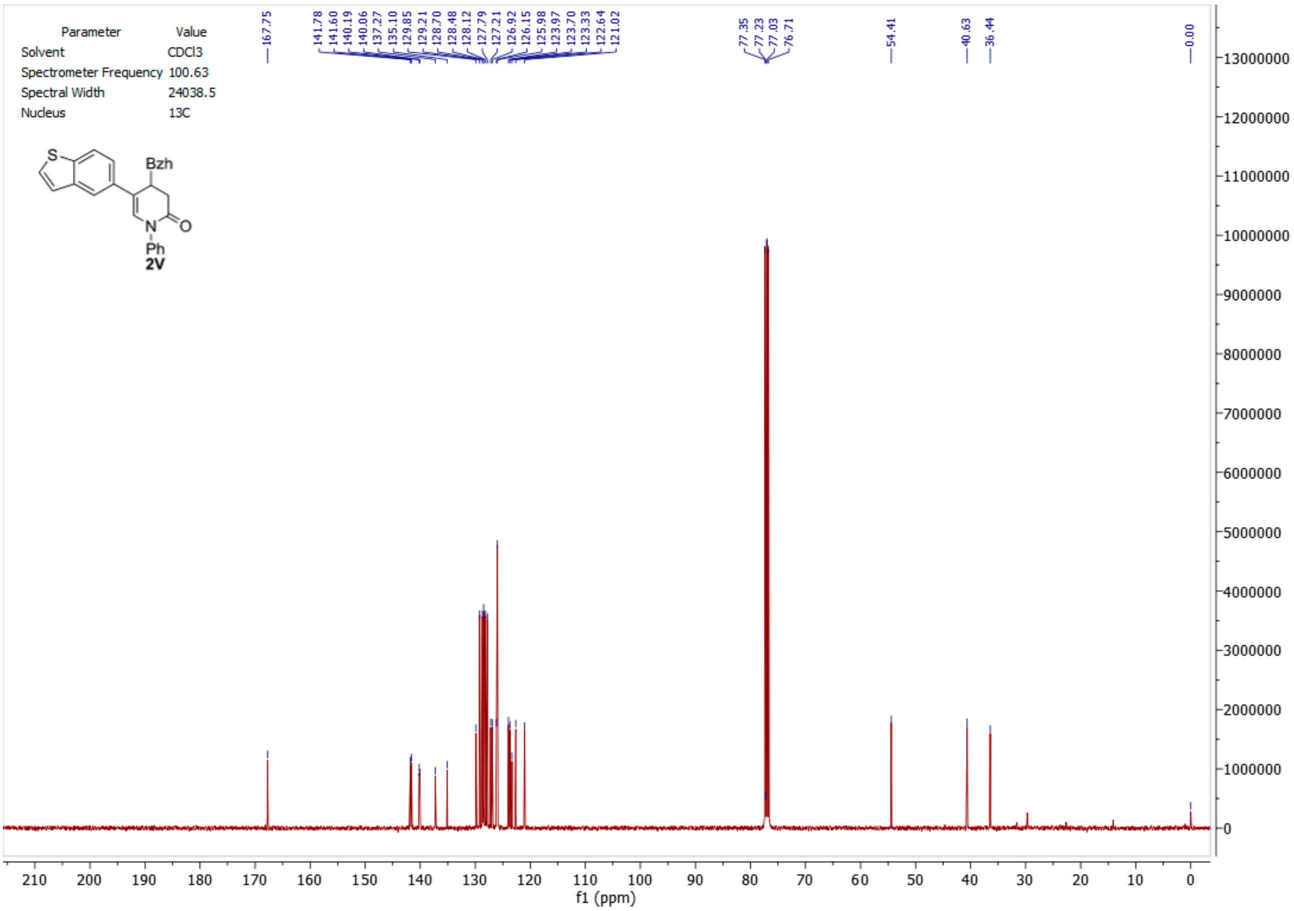

Parameter Value
Solvent CDCl3
Spectrometer Frequency 100.63
Spectral Width 24038.5
Nucleus 13C
Bzh
N
O
S
Ph
2V
f1 (ppm)

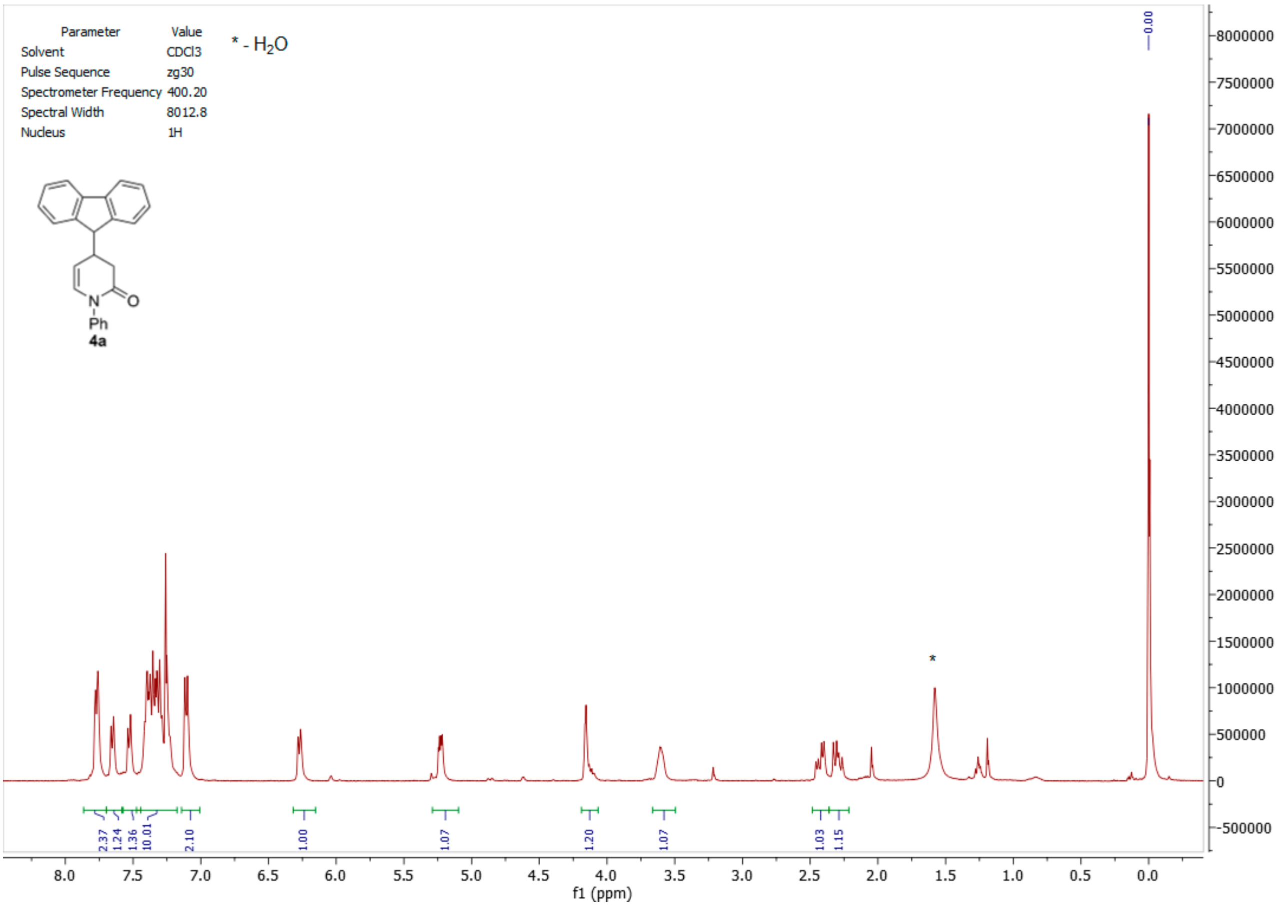
Parameter Value
Solvent CDCl3
Pulse Sequence zg30
Spectrometer Frequency 400.20
Spectral Width 8012.8
Nucleus 1H
* - H2O
4a
f1 (ppm)

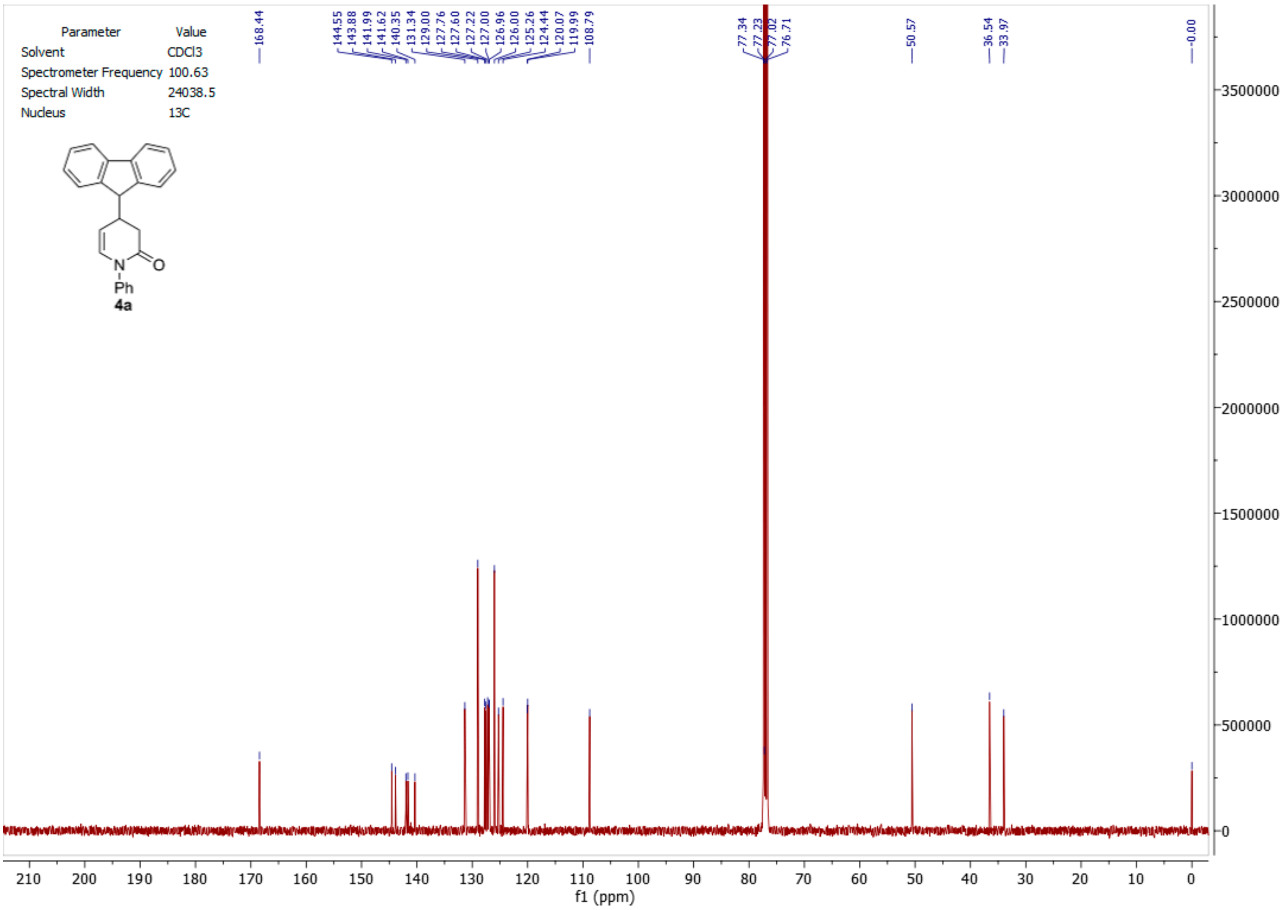
Parameter Value
Solvent CDCl3
Spectrometer Frequency 100.63
Spectral Width 24038.5
Nucleus 13C
4a
f1 (ppm)

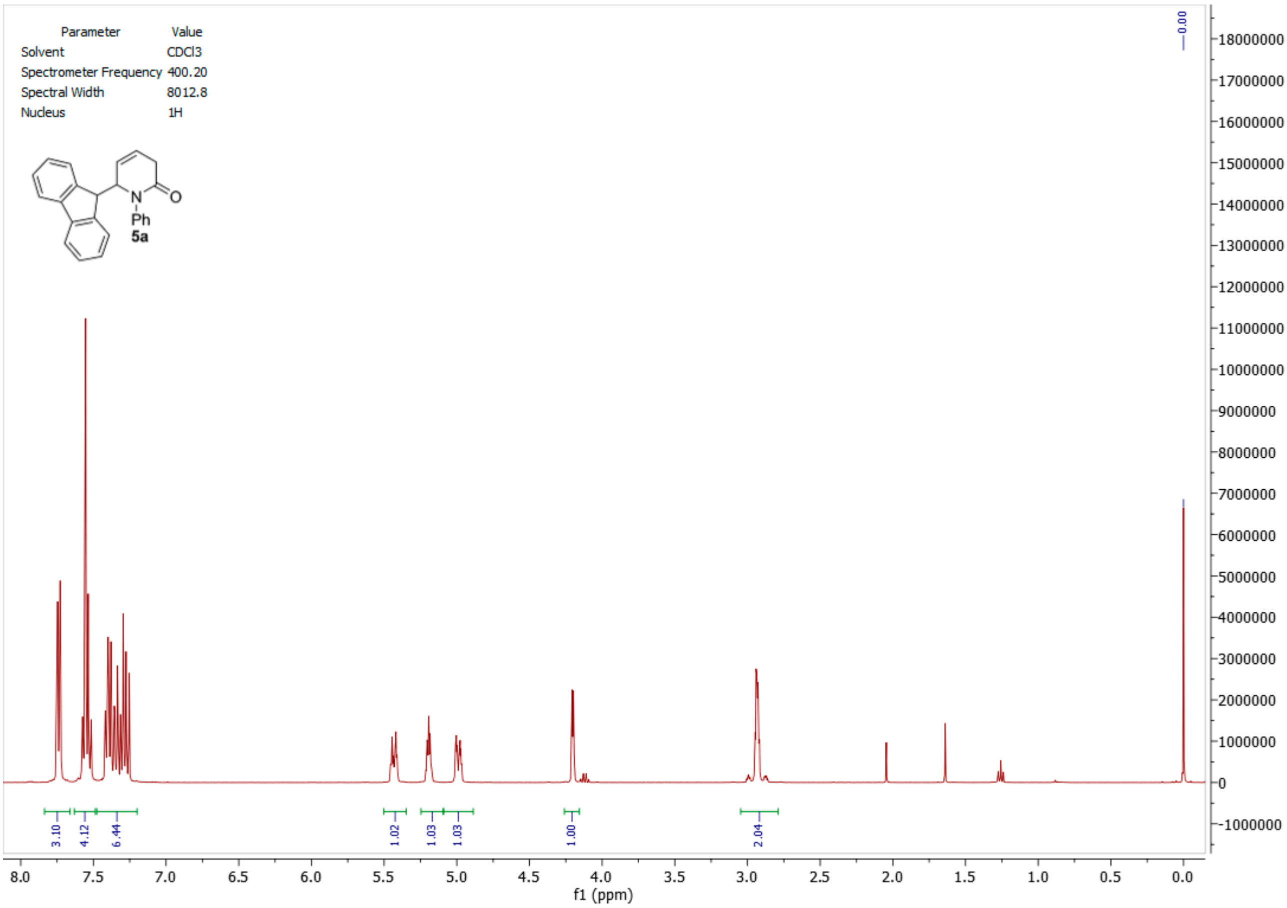

Parameter Value
Solvent CDCl3
Spectrometer Frequency 400.20
Spectral Width 8012.8
Nucleus 1H
N
Ph
O
5a
0.00
3.10
4.12
6.44
1.02
1.03
1.03
1.00
2.04
f1 (ppm)

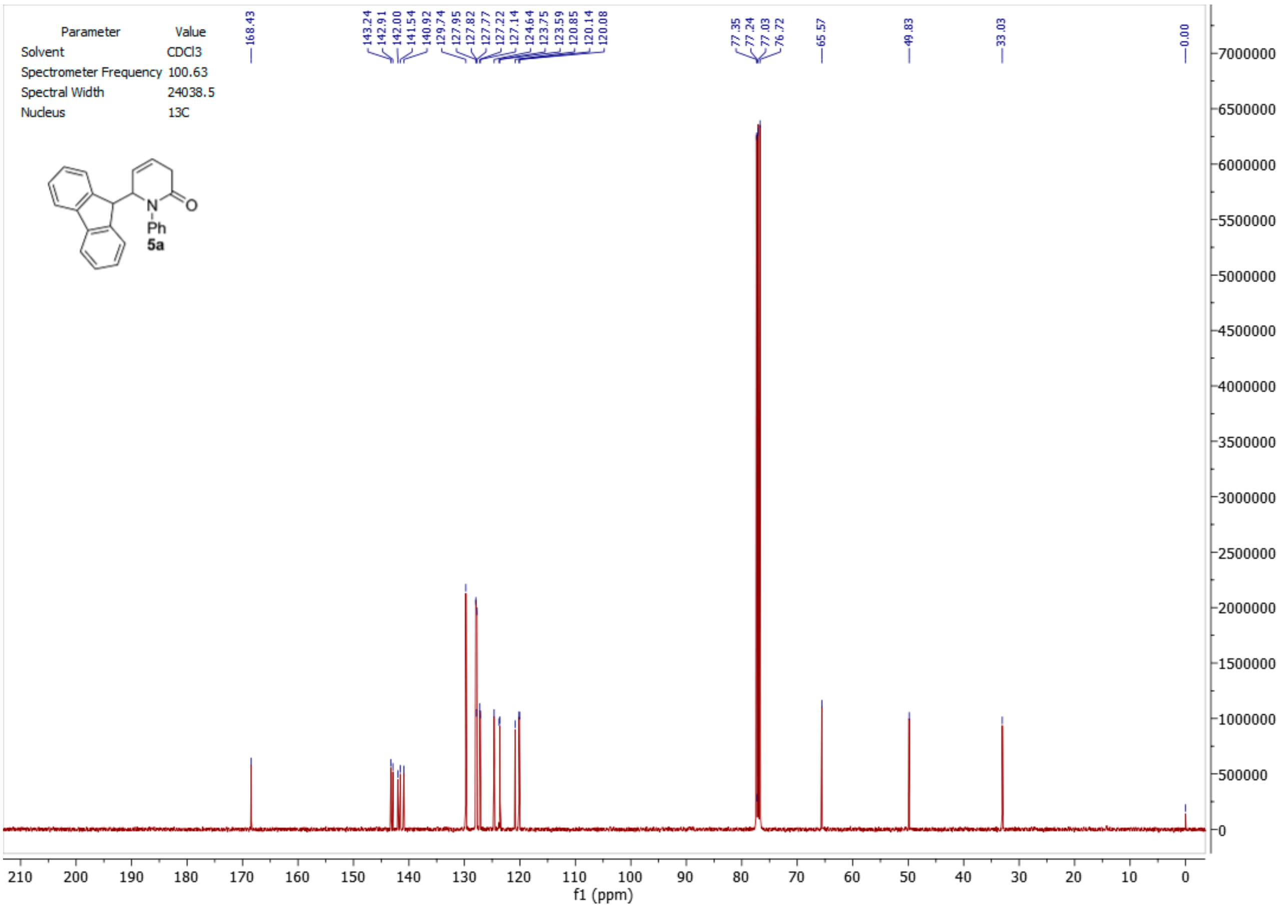

Parameter Value
Solvent CDCl3
Spectrometer Frequency 100.63
Spectral Width 24038.5
Nucleus 13C
N
Ph
O
5a
168.43
143.24
142.91
142.00
141.54
140.92
129.74
127.95
127.82
127.77
127.22
127.14
124.64
123.75
123.59
120.85
120.14
120.08
77.35
77.24
77.03
76.72
65.57
49.83
33.03
0.00
f1 (ppm)

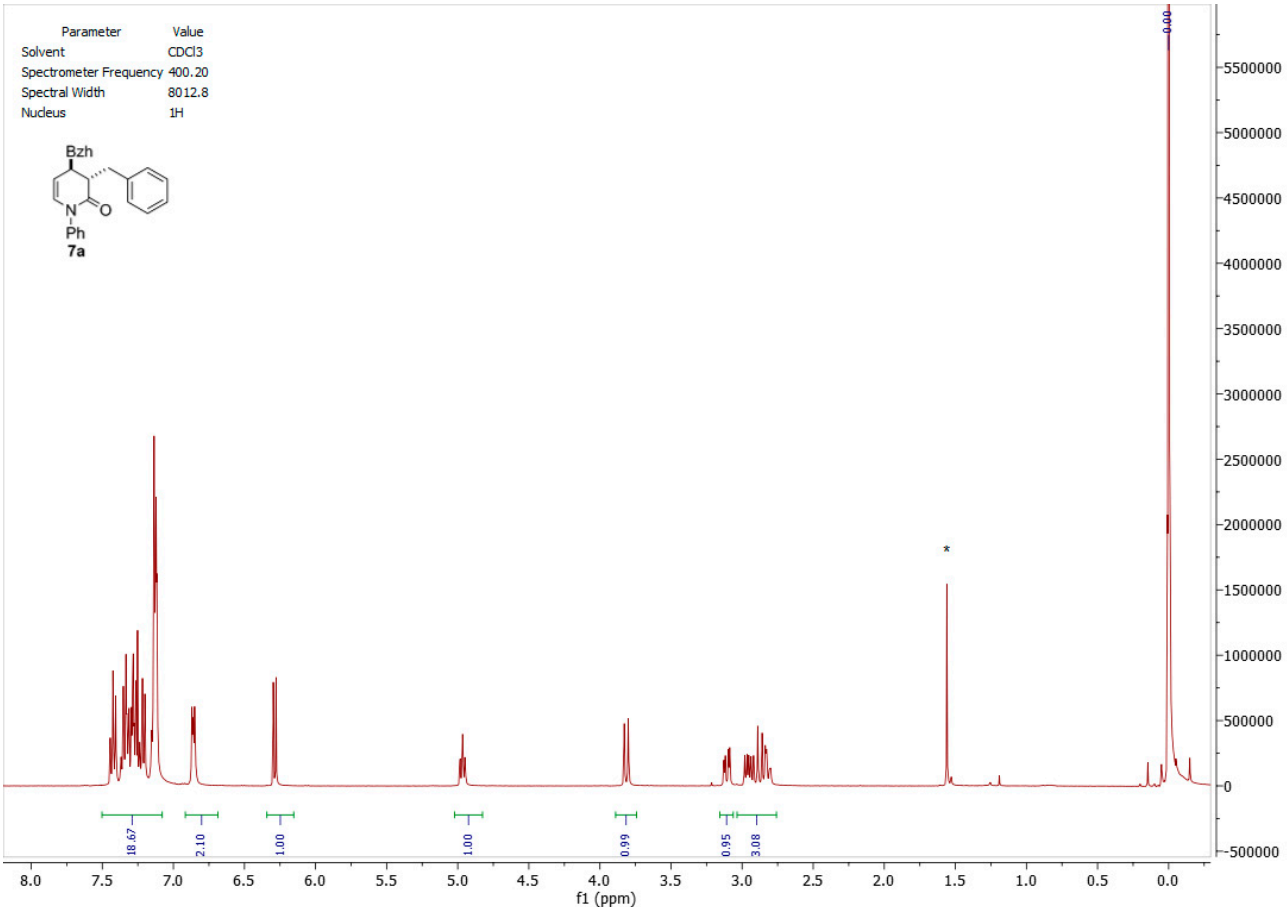
Parameter Value
Solvent CDCl3
Spectrometer Frequency 400.20
Spectral Width 8012.8
Nucleus 1H
Bzh
N
O
Ph
7a
f1 (ppm)

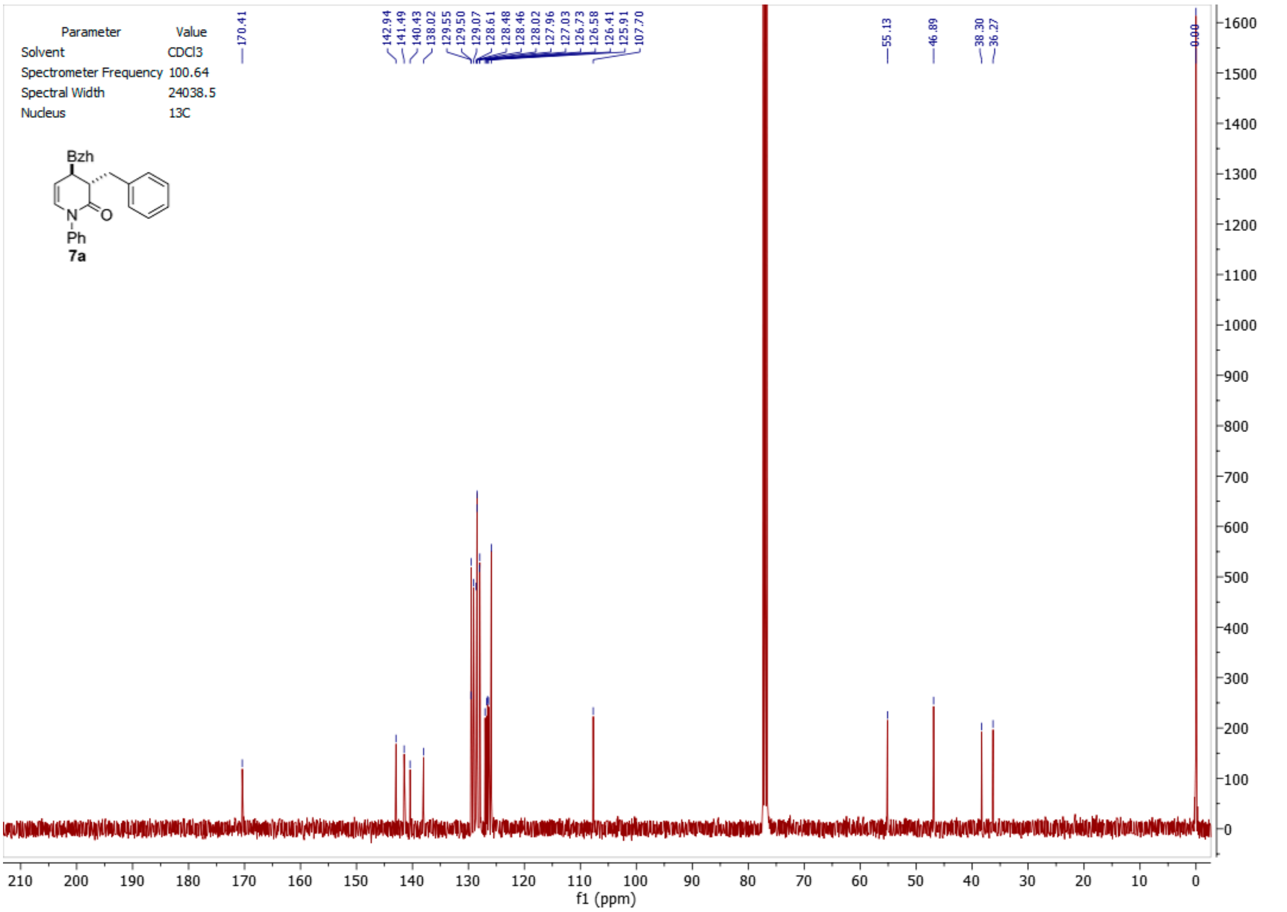
Parameter Value
Solvent CDCl3
Spectrometer Frequency 100.64
Spectral Width 24038.5
Nucleus 13C
Bzh
N
O
Ph
7a
f1 (ppm)

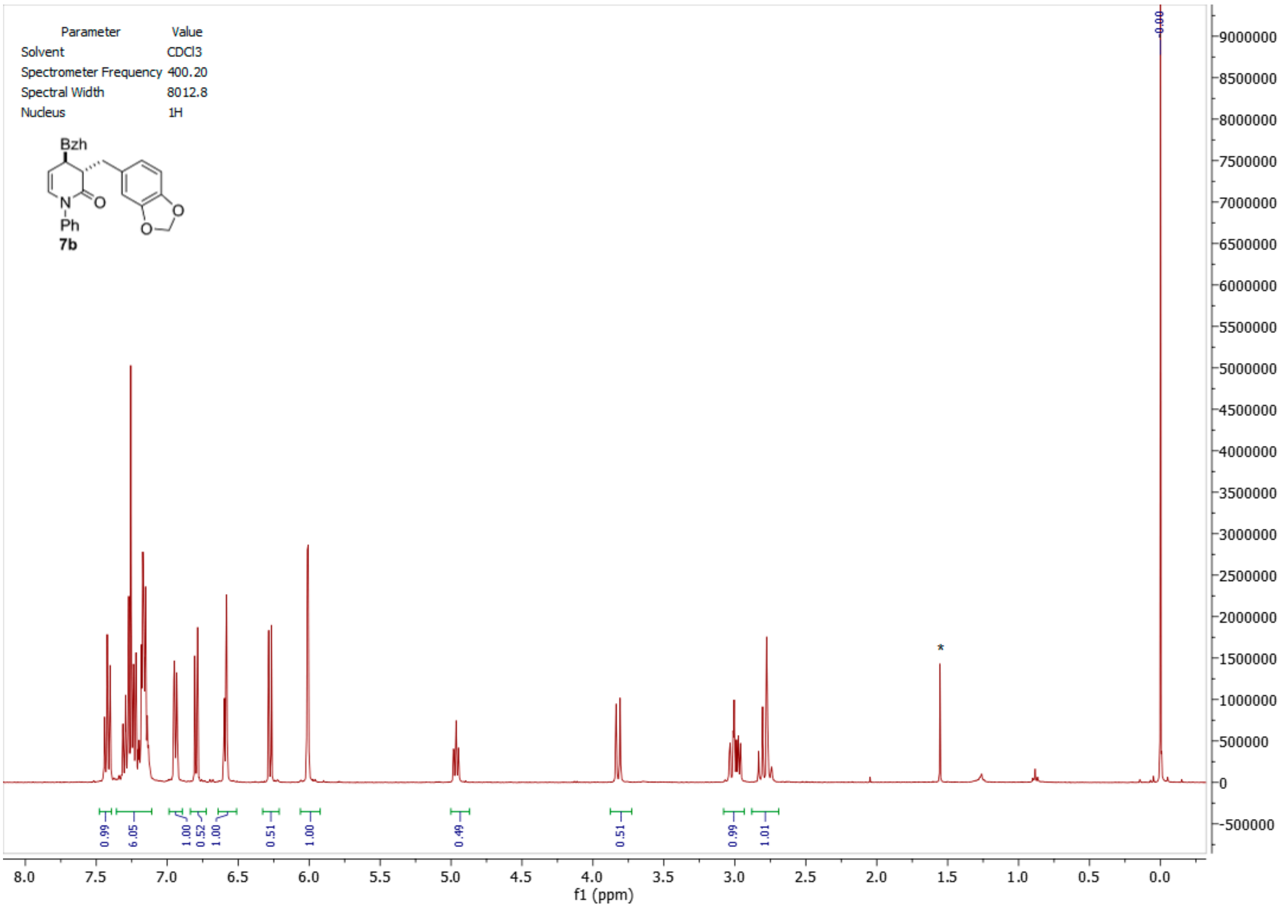

Parameter Value
Solvent CDCl3
Spectrometer Frequency 400.20
Spectral Width 8012.8
Nucleus 1H
Bzh
N
O
Ph
7b
O
O
f1 (ppm)

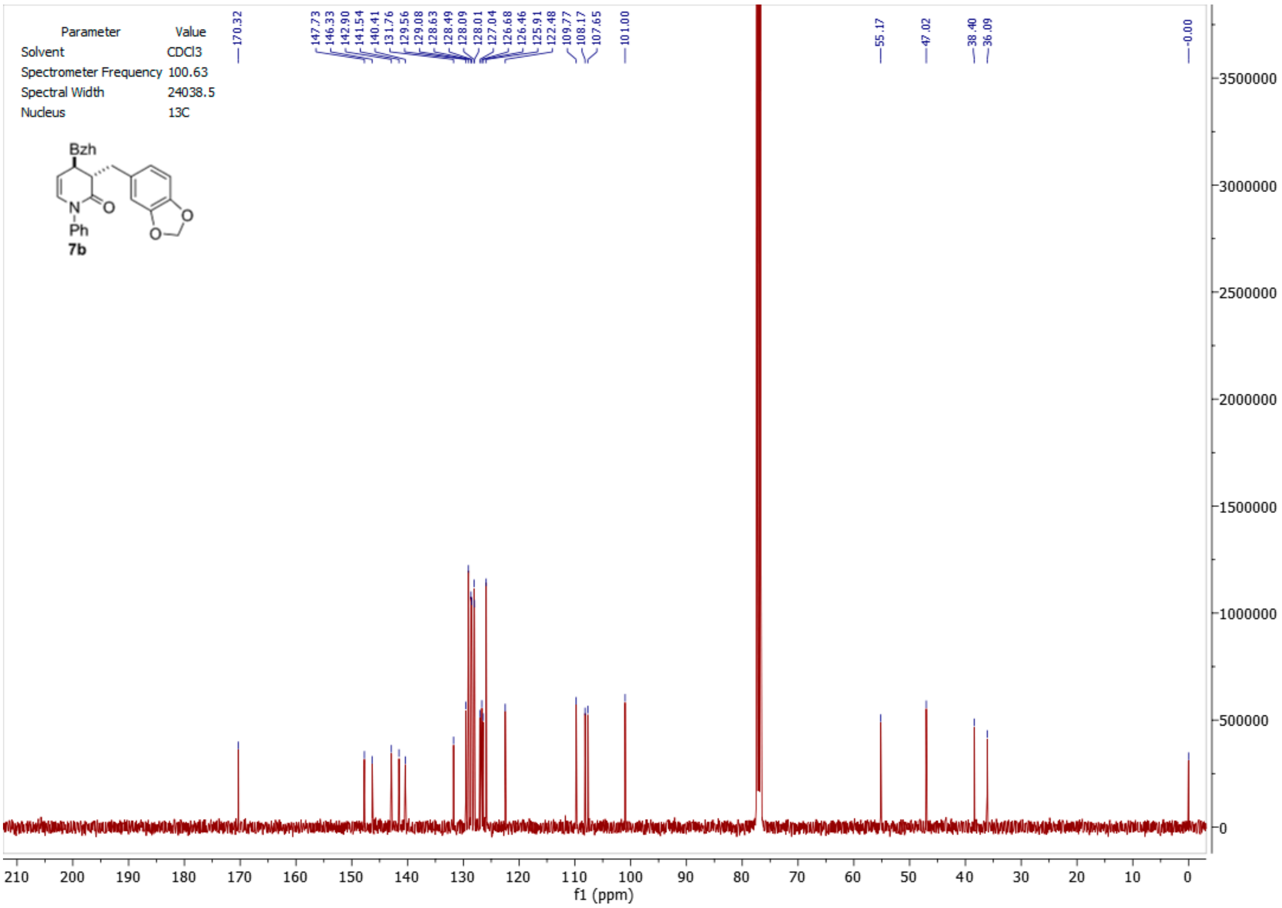

Parameter Value
Solvent CDCl3
Spectrometer Frequency 100.63
Spectral Width 24038.5
Nucleus 13C
Bzh
N
O
Ph
7b
O
O
f1 (ppm)

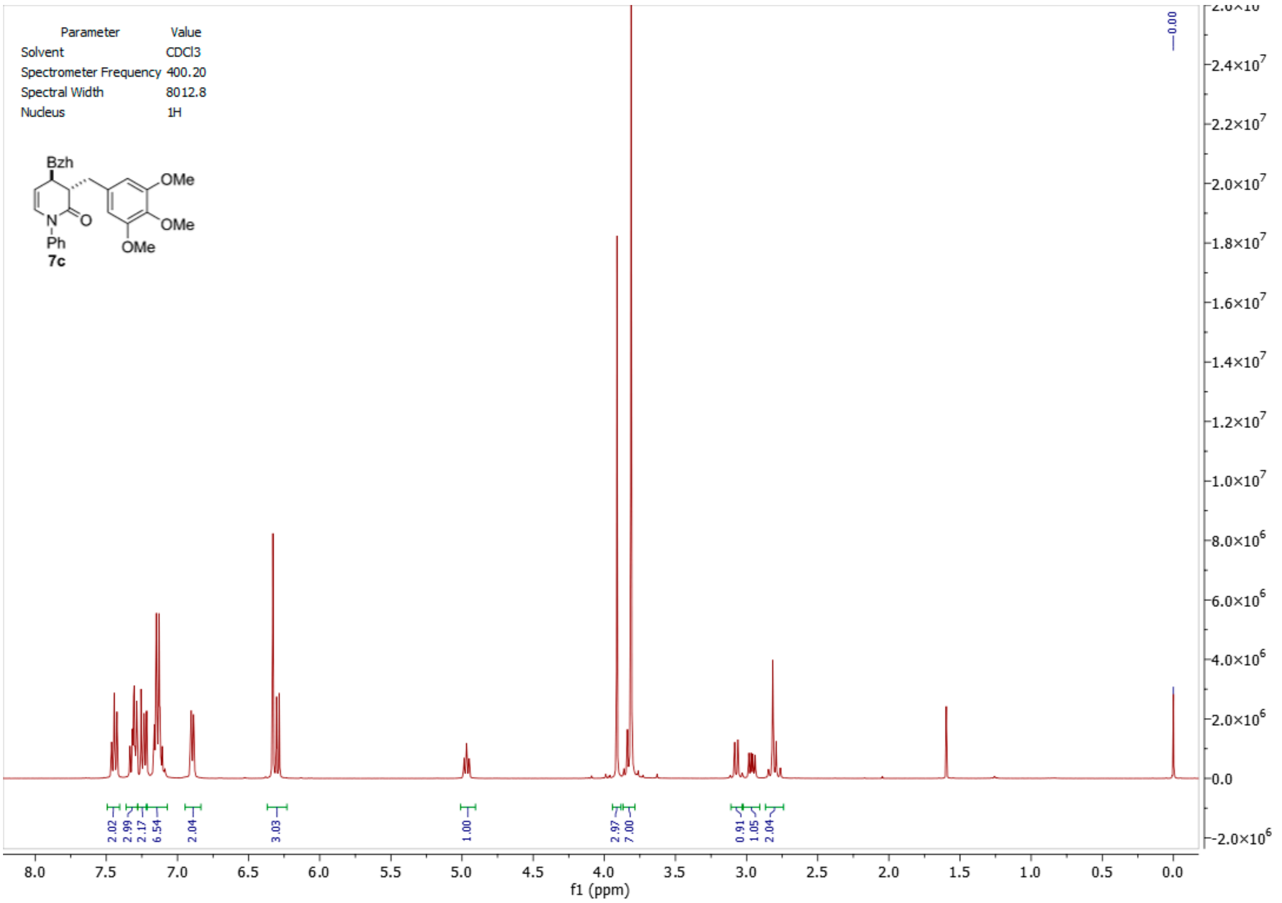

Parameter Value
Solvent CDCl3
Spectrometer Frequency 400.20
Spectral Width 8012.8
Nucleus 1H
Bzh
N
O
Ph
OMe
OMe
OMe
7c
f1 (ppm)

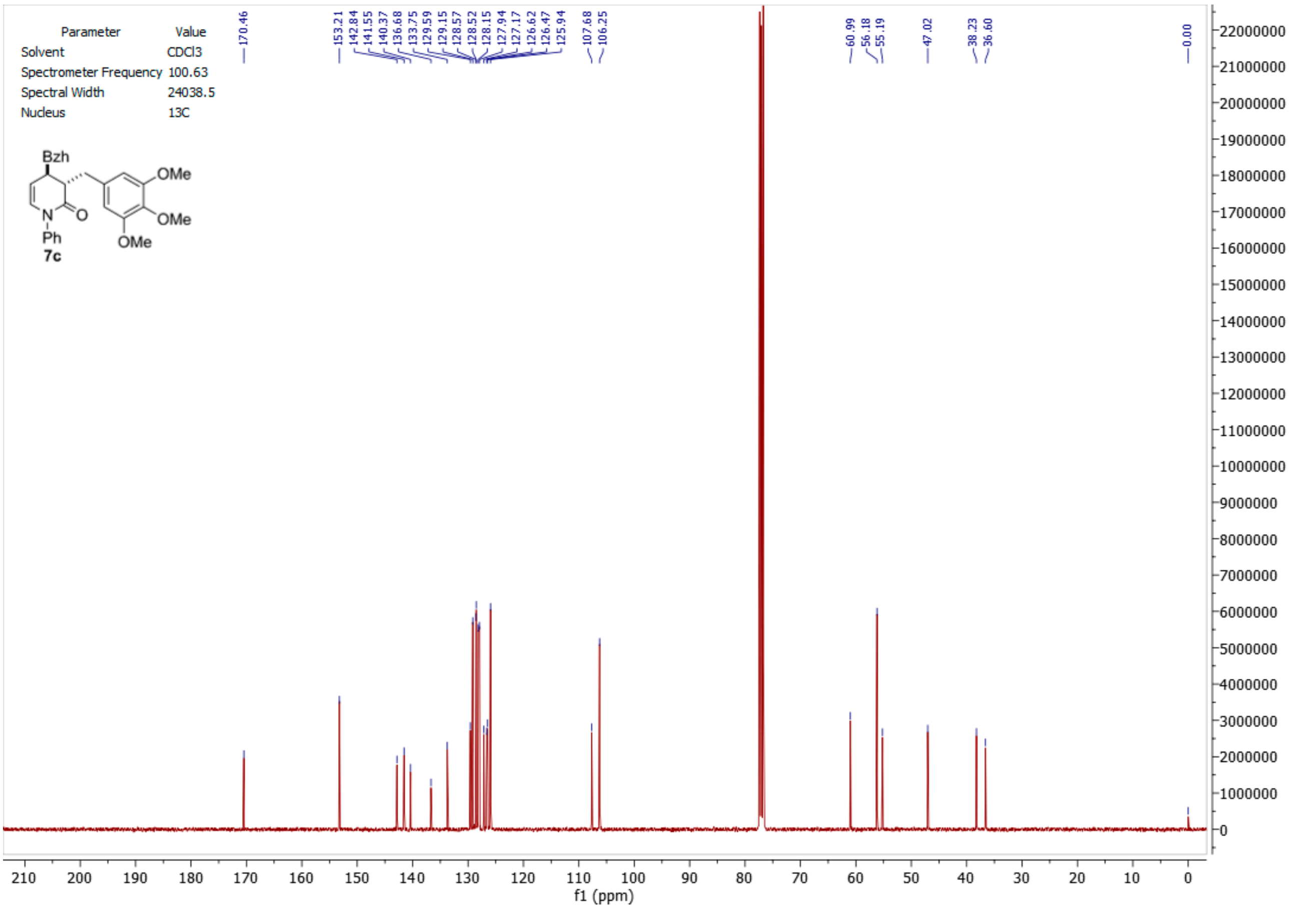

Parameter Value
Solvent CDCl3
Spectrometer Frequency 100.63
Spectral Width 24038.5
Nucleus 13C
Bzh
N
O
Ph
OMe
OMe
OMe
7c
170.46
153.21
142.84
141.55
140.37
136.68
133.75
129.59
129.15
128.57
128.52
128.15
127.94
127.17
126.62
126.47
125.94
107.68
106.25
60.99
56.18
55.19
47.02
38.23
36.60
0.00
f1 (ppm)

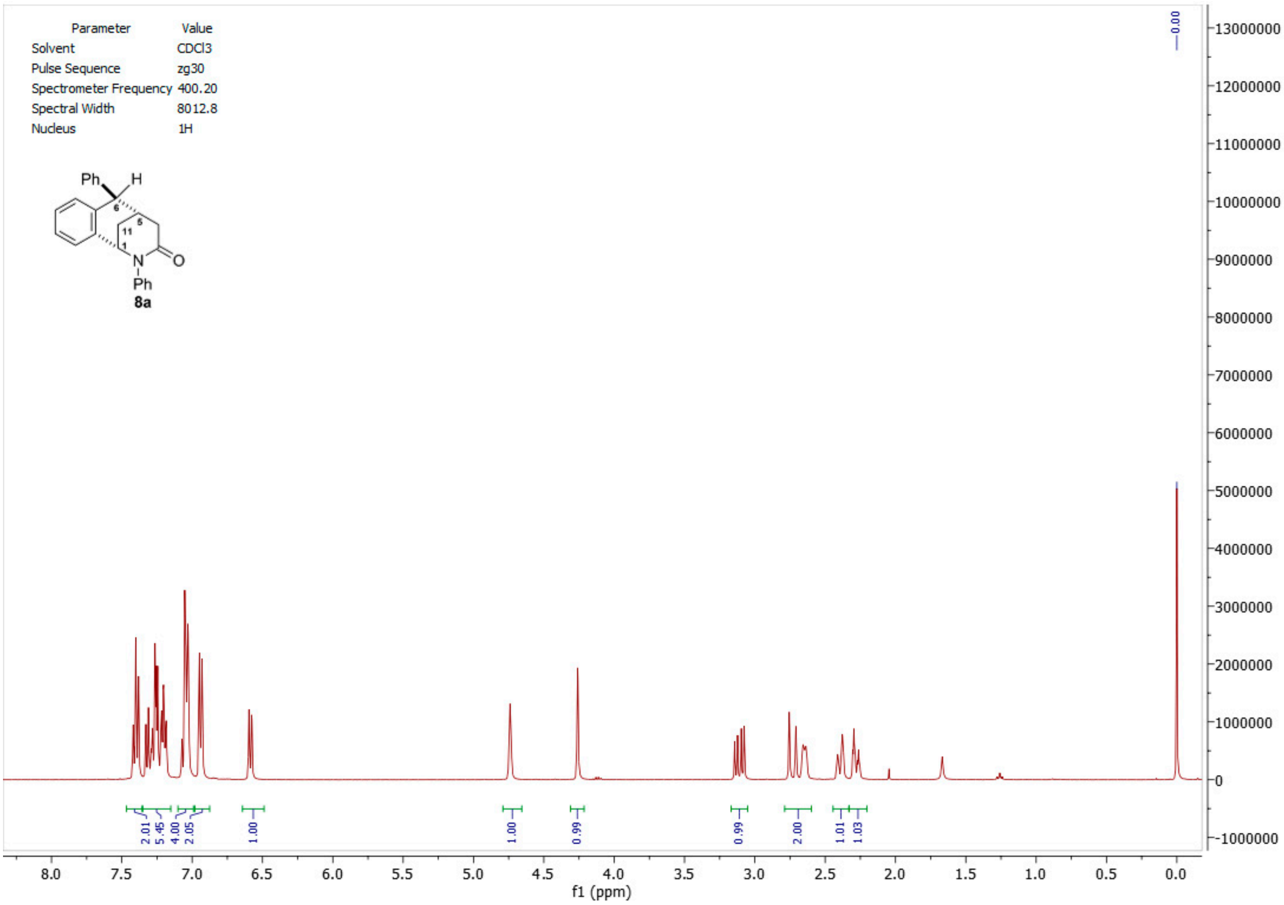
Parameter Value
Solvent CDCl3
Pulse Sequence zg30
Spectrometer Frequency 400.20
Spectral Width 8012.8
Nucleus 1H
Ph
H
N
O
Ph
8a
0.00
2.01
5.45
4.00
2.05
1.00
1.00
0.99
0.99
2.00
1.01
1.03
f1 (ppm)

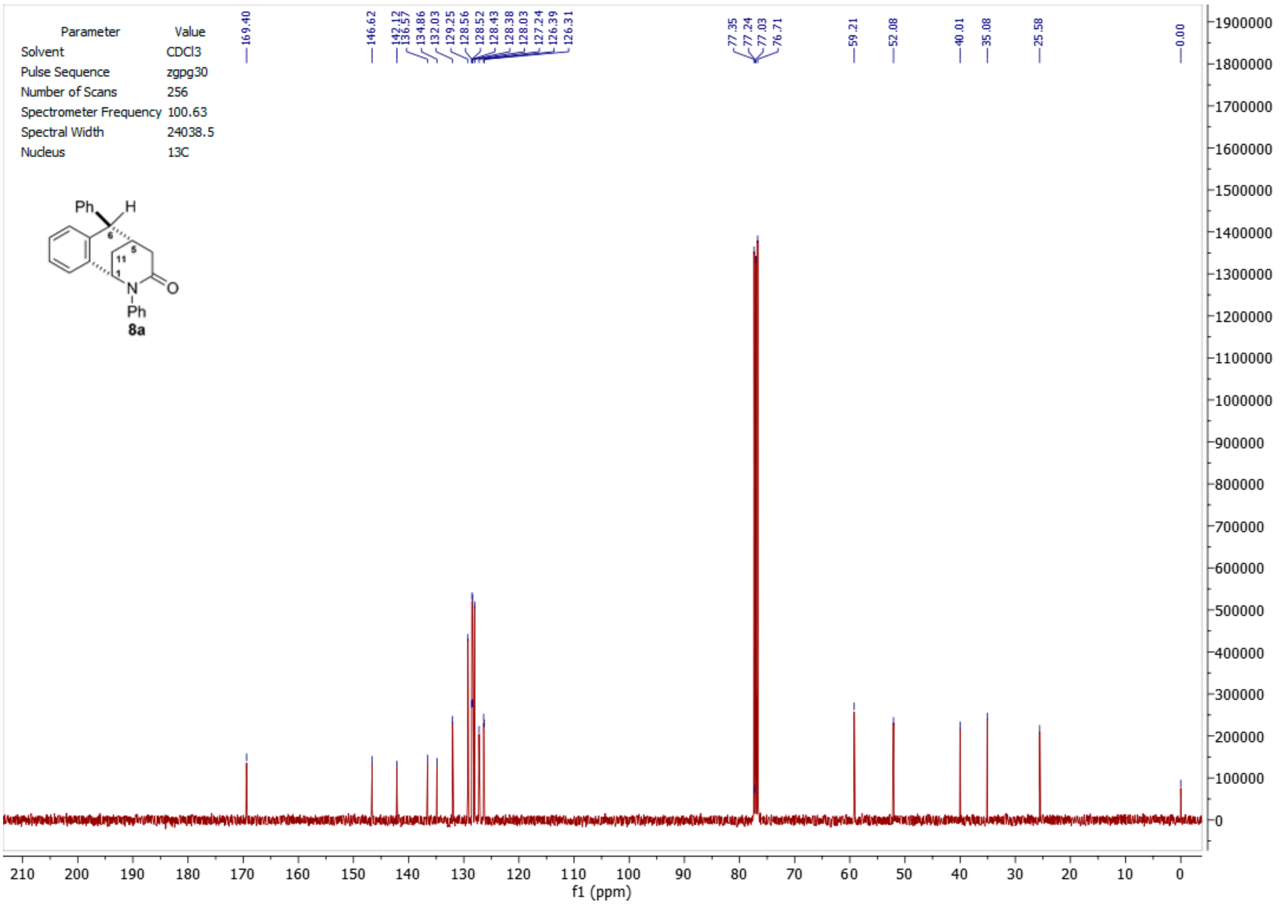
Parameter Value
Solvent CDCl3
Pulse Sequence zgpg30
Number of Scans 256
Spectrometer Frequency 100.63
Spectral Width 24038.5
Nucleus 13C
Ph
H
N
O
Ph
8a
169.40
146.62
142.12
136.57
134.86
132.03
129.25
128.56
128.52
128.43
128.38
128.03
127.24
126.39
126.31
77.35
77.24
77.03
76.71
59.21
52.08
40.01
35.08
25.58
0.00
f1 (ppm)

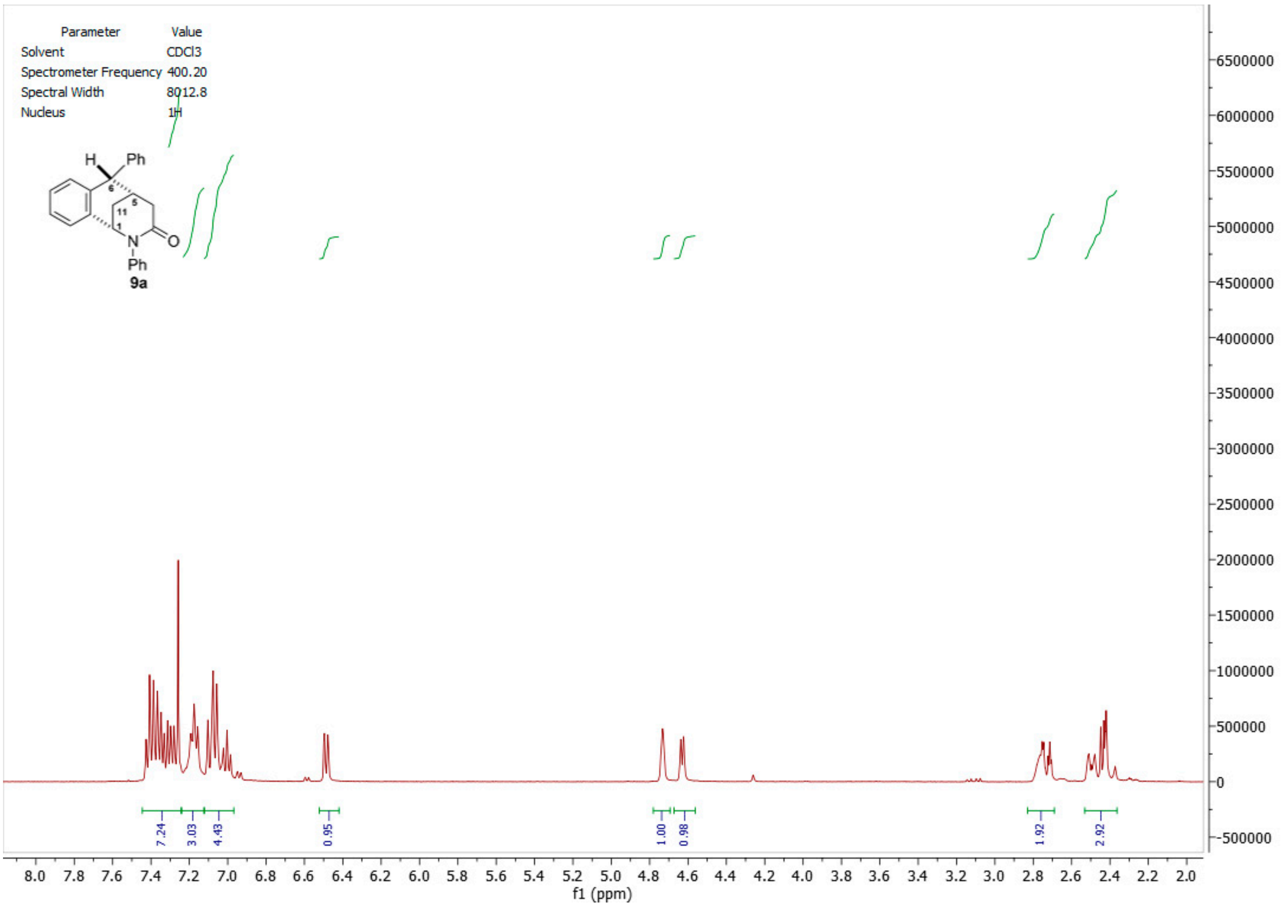
Parameter Value
Solvent CDCl3
Spectrometer Frequency 400.20
Spectral Width 8012.8
Nucleus 1H
9a
7.24
3.03
4.43
0.95
1.00
0.98
1.92
2.92
f1 (ppm)

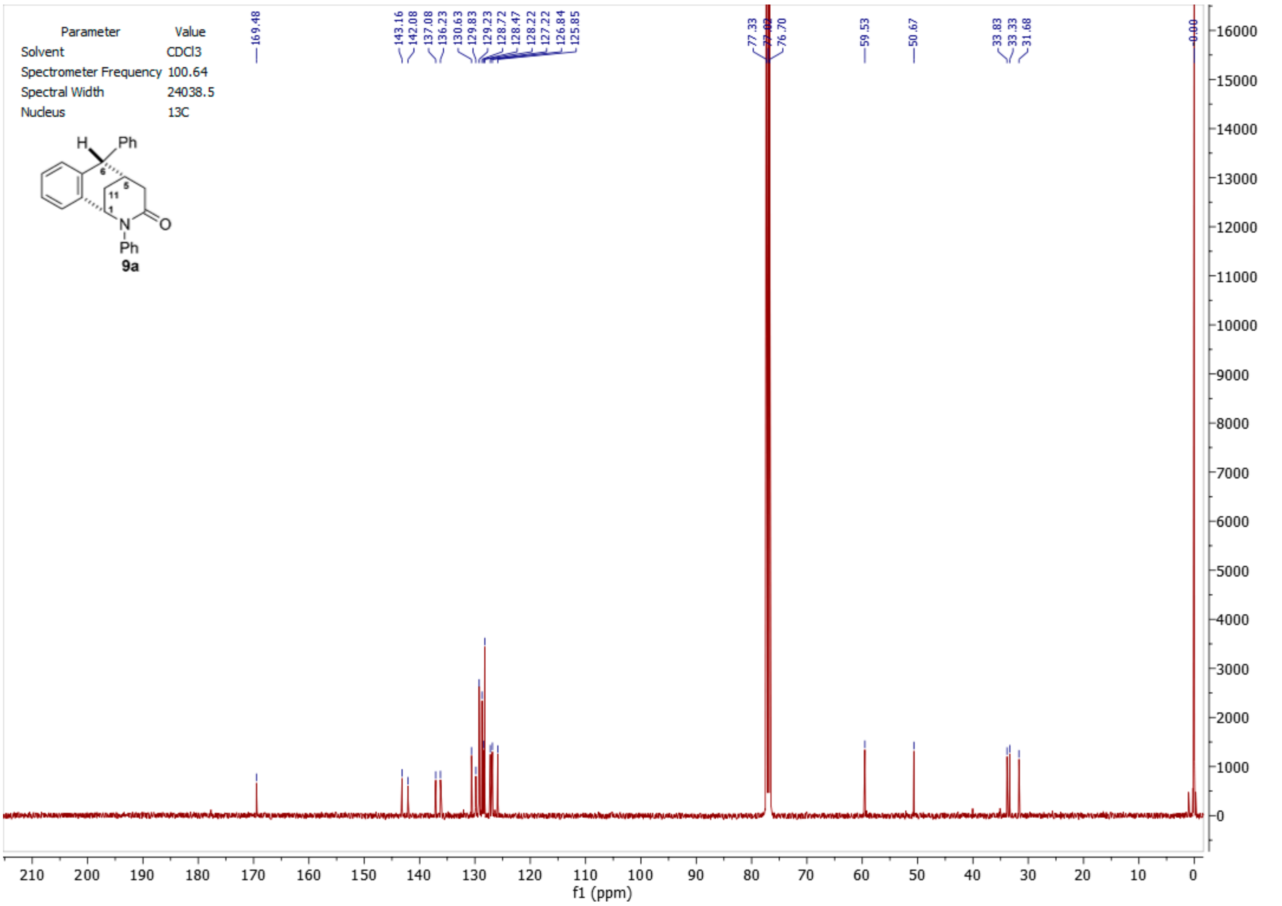
Parameter Value
Solvent CDCl3
Spectrometer Frequency 100.64
Spectral Width 24038.5
Nucleus 13C
9a
169.48
143.16
142.08
137.08
136.23
130.63
129.83
129.23
128.72
128.47
128.22
127.22
126.84
125.85
77.33
76.70
59.53
50.67
33.83
33.33
31.68
f1 (ppm)

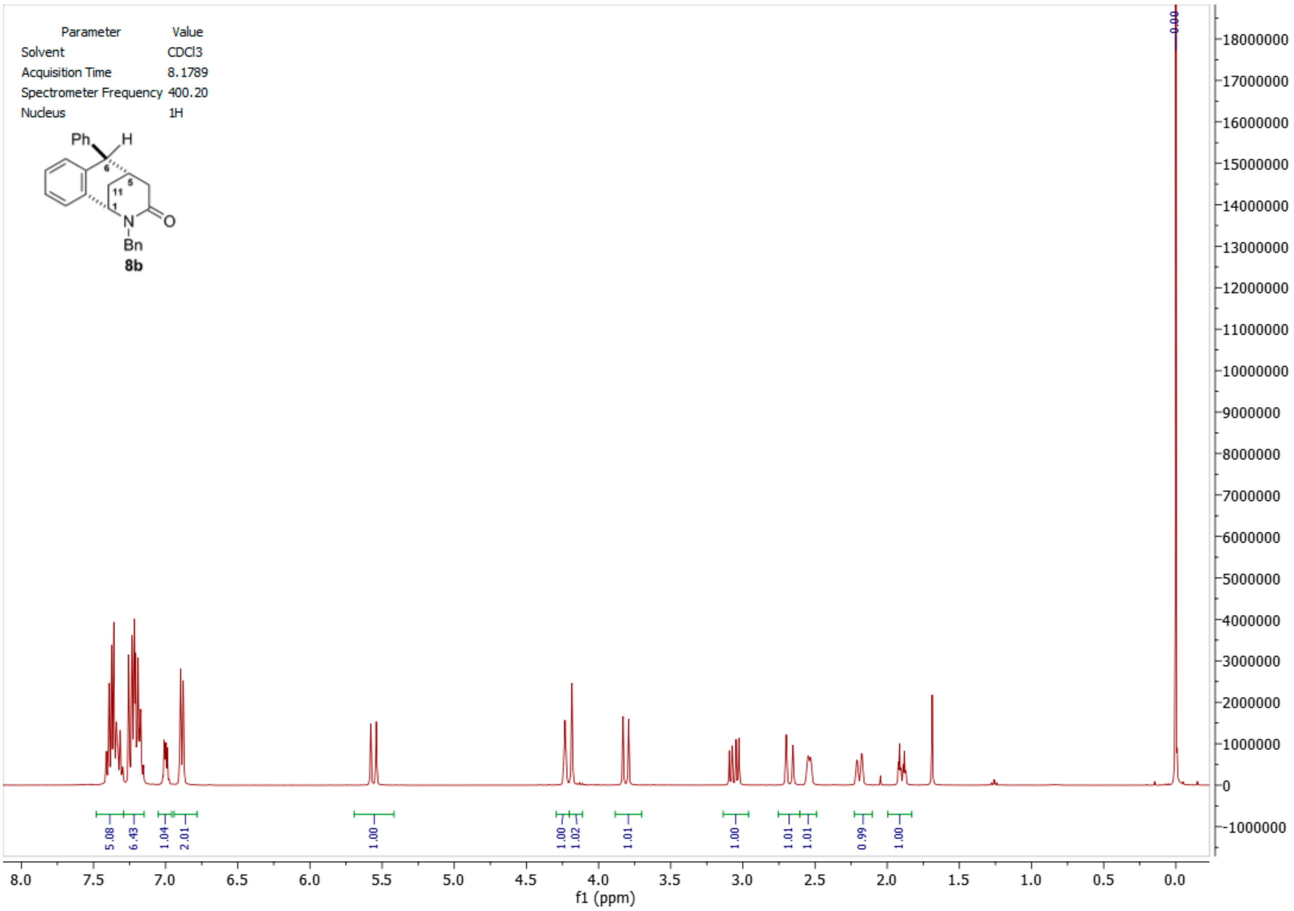
Parameter Value
Solvent CDCl3
Acquisition Time 8.1789
Spectrometer Frequency 400.20
Nucleus 1H
Ph H
N O
Bn
8b
5.08 6.43 1.04 2.01 1.00 1.00 1.02 1.01 1.00 1.01 1.01 0.99 1.00
0.00
f1 (ppm)

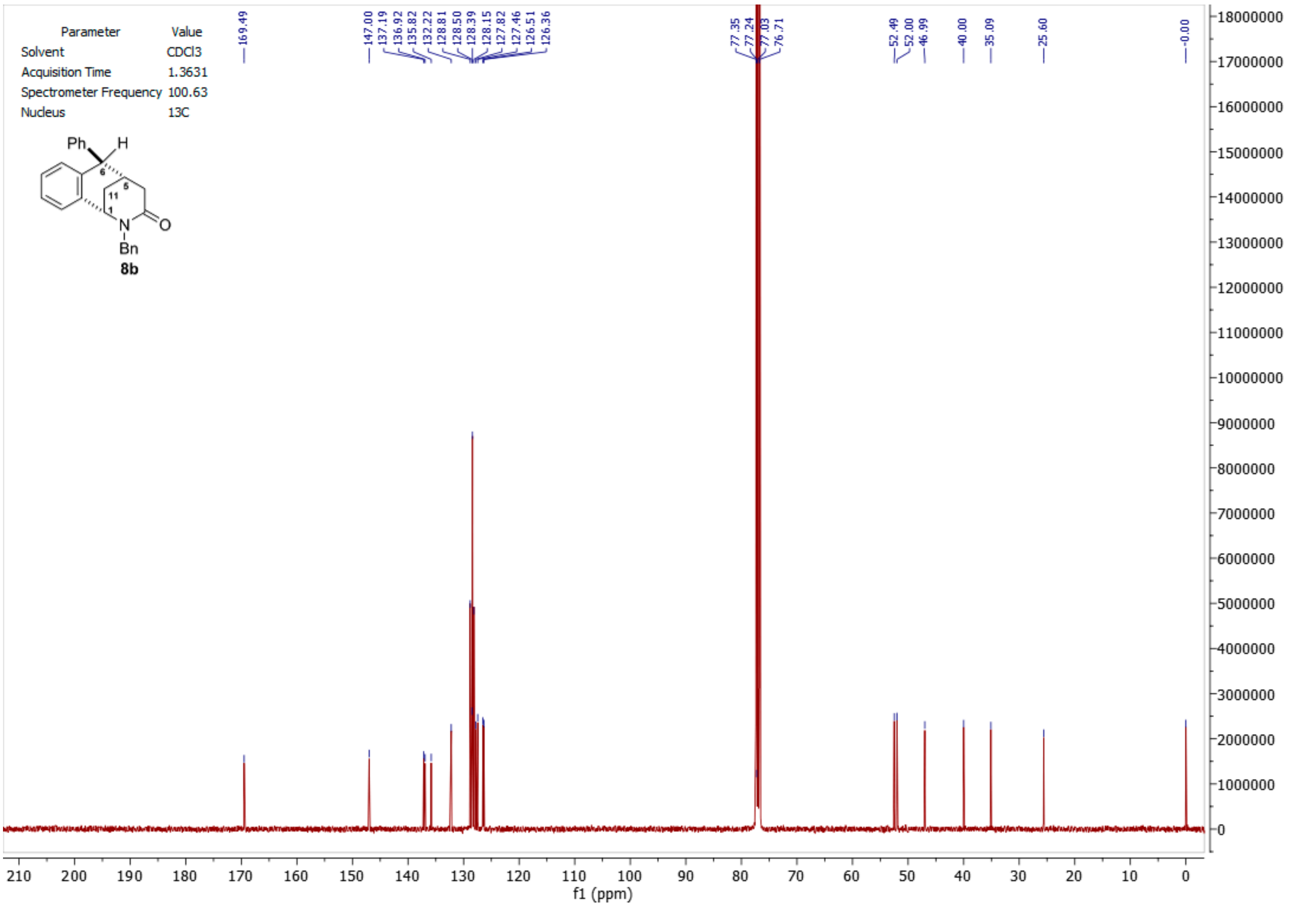
Parameter Value
Solvent CDCl3
Acquisition Time 1.3631
Spectrometer Frequency 100.63
Nucleus 13C
Ph H
N O
Bn
8b
169.49
147.00 137.19 136.92 135.82 132.22 128.81 128.50 128.39 128.15 127.82 127.46 126.51 126.36
77.35 77.24 77.03 76.71
52.49 52.00 46.99 40.00 35.09 25.60 -0.00
f1 (ppm)

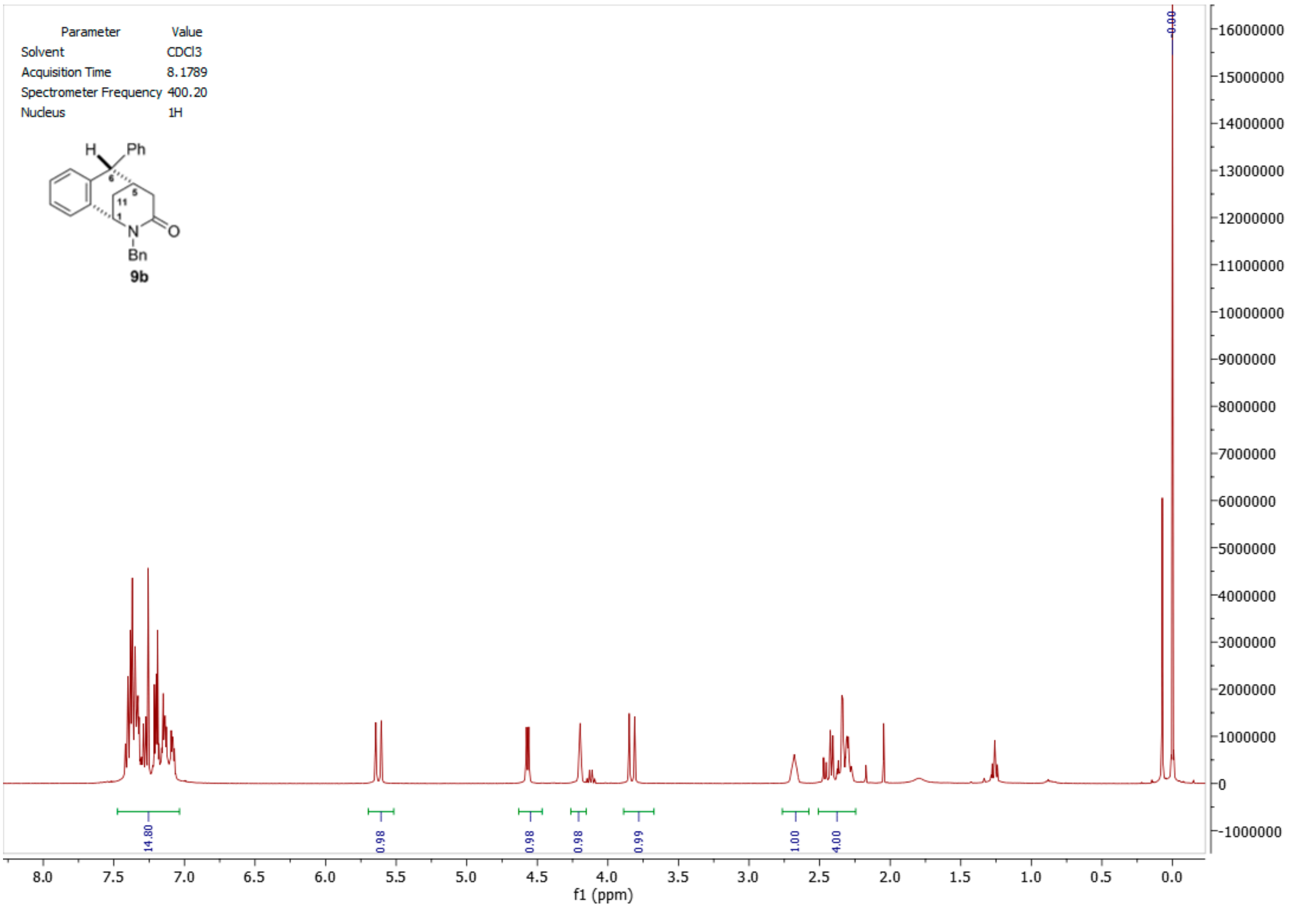
Parameter Value
Solvent CDCl3
Acquisition Time 8.1789
Spectrometer Frequency 400.20
Nucleus 1H
9b
f1 (ppm)

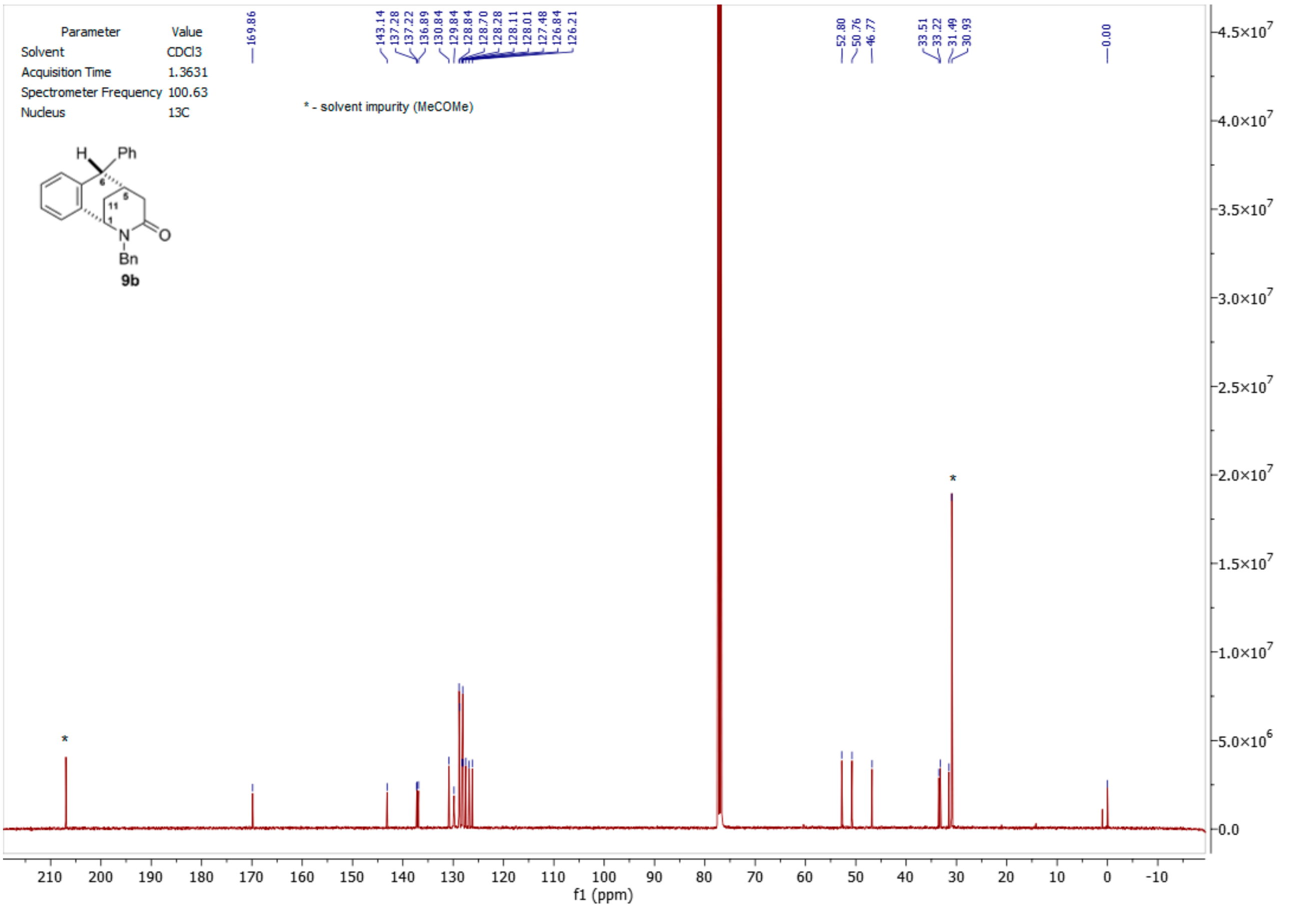
Parameter Value
Solvent CDCl3
Acquisition Time 1.3631
Spectrometer Frequency 100.63
Nucleus 13C
* - solvent impurity (MeCOMe)
169.86
143.14
137.28
137.22
136.89
130.84
129.84
128.84
128.70
128.28
128.11
128.01
127.48
126.84
126.21
52.80
50.76
46.77
33.51
33.22
31.49
30.93
0.00
9b
f1 (ppm)

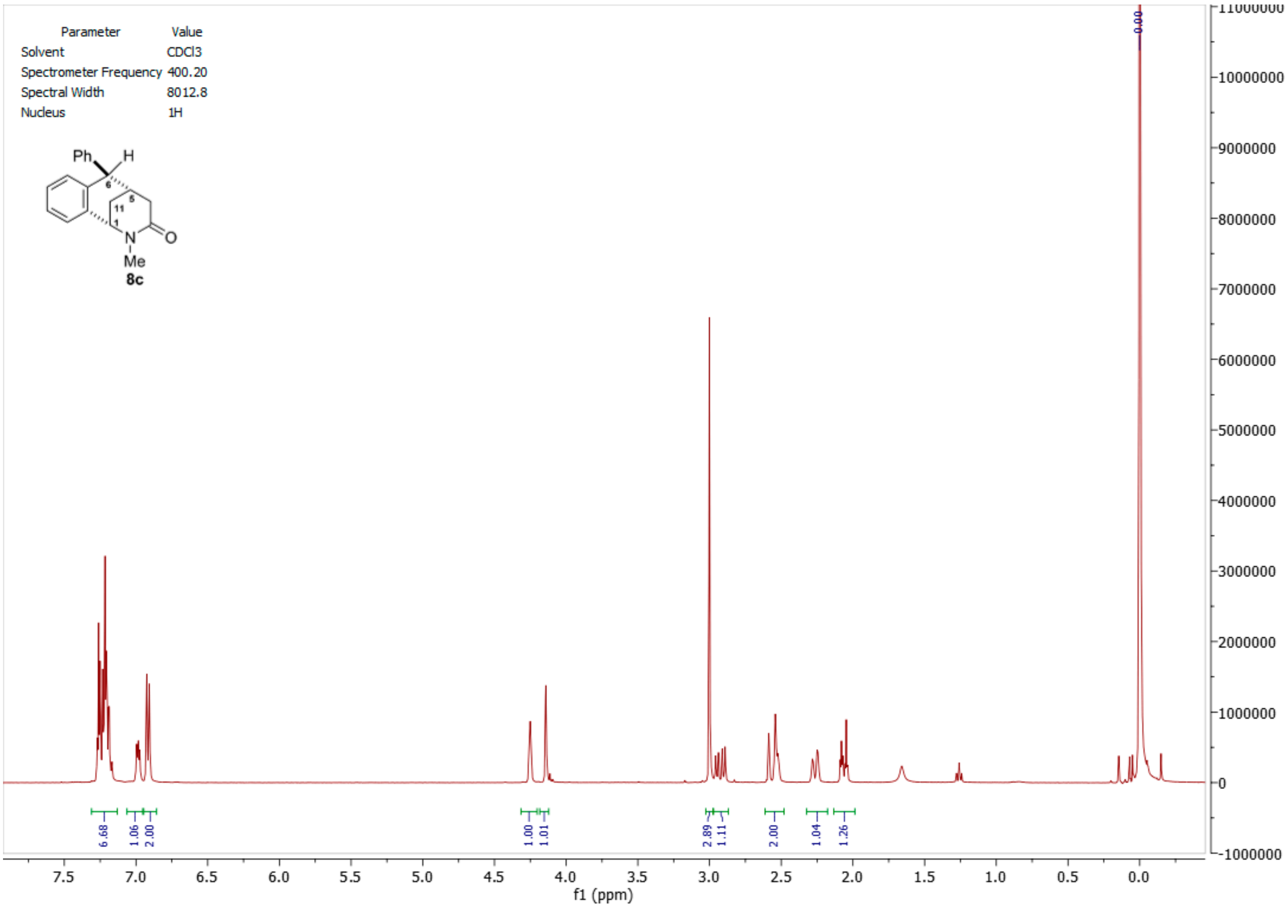
Parameter Value
Solvent CDCl3
Spectrometer Frequency 400.20
Spectral Width 8012.8
Nucleus 1H
Ph
H
N
O
Me
8c
6.68
1.06
2.00
1.00
1.01
2.89
1.11
2.00
1.04
1.26
0.00
f1 (ppm)

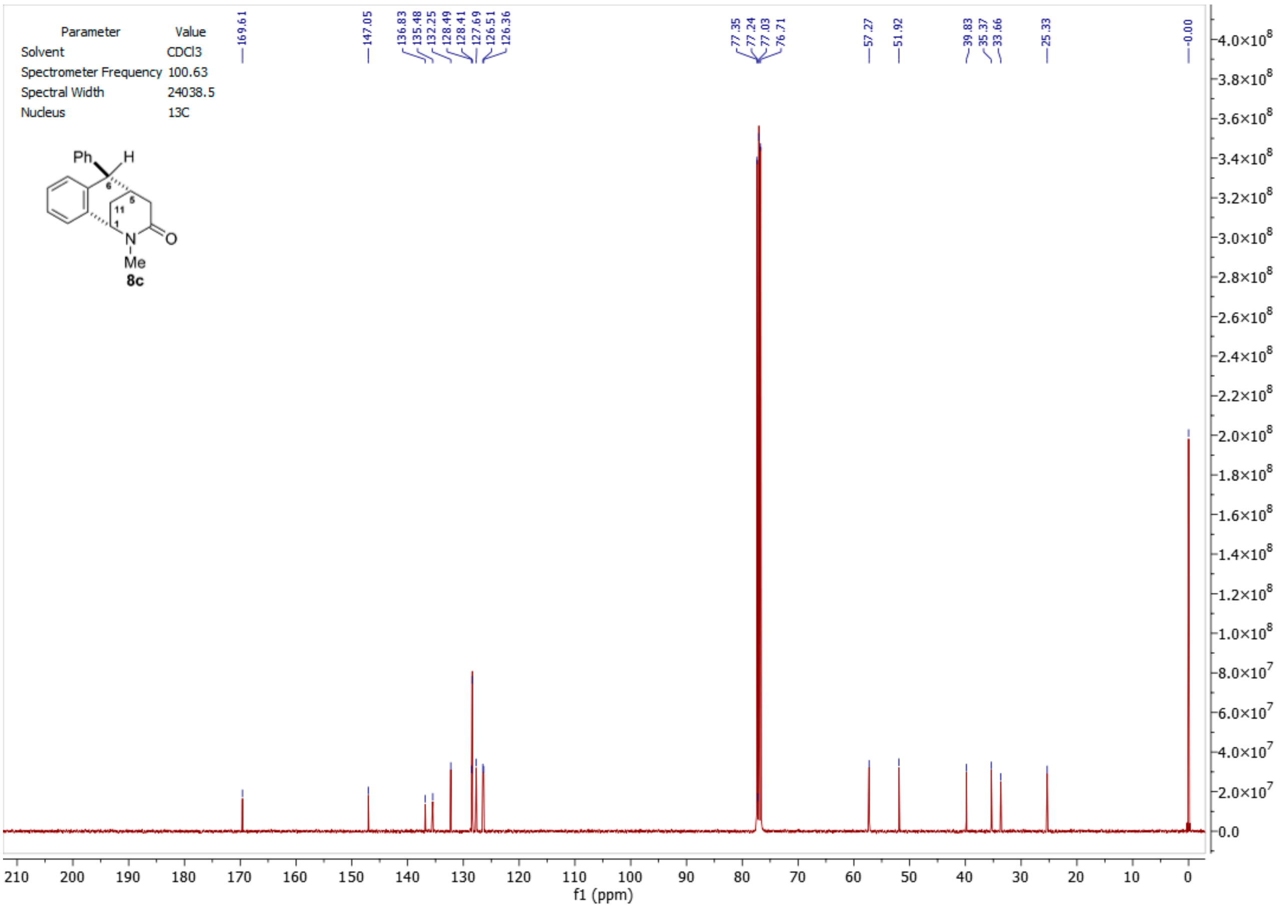
Parameter Value
Solvent CDCl3
Spectrometer Frequency 100.63
Spectral Width 24038.5
Nucleus 13C
Ph
H
N
O
Me
8c
169.61
147.05
136.83
135.48
132.25
128.49
128.41
127.69
126.51
126.36
77.35
77.24
77.03
76.71
57.27
51.92
39.83
35.37
33.66
25.33
0.00
f1 (ppm)

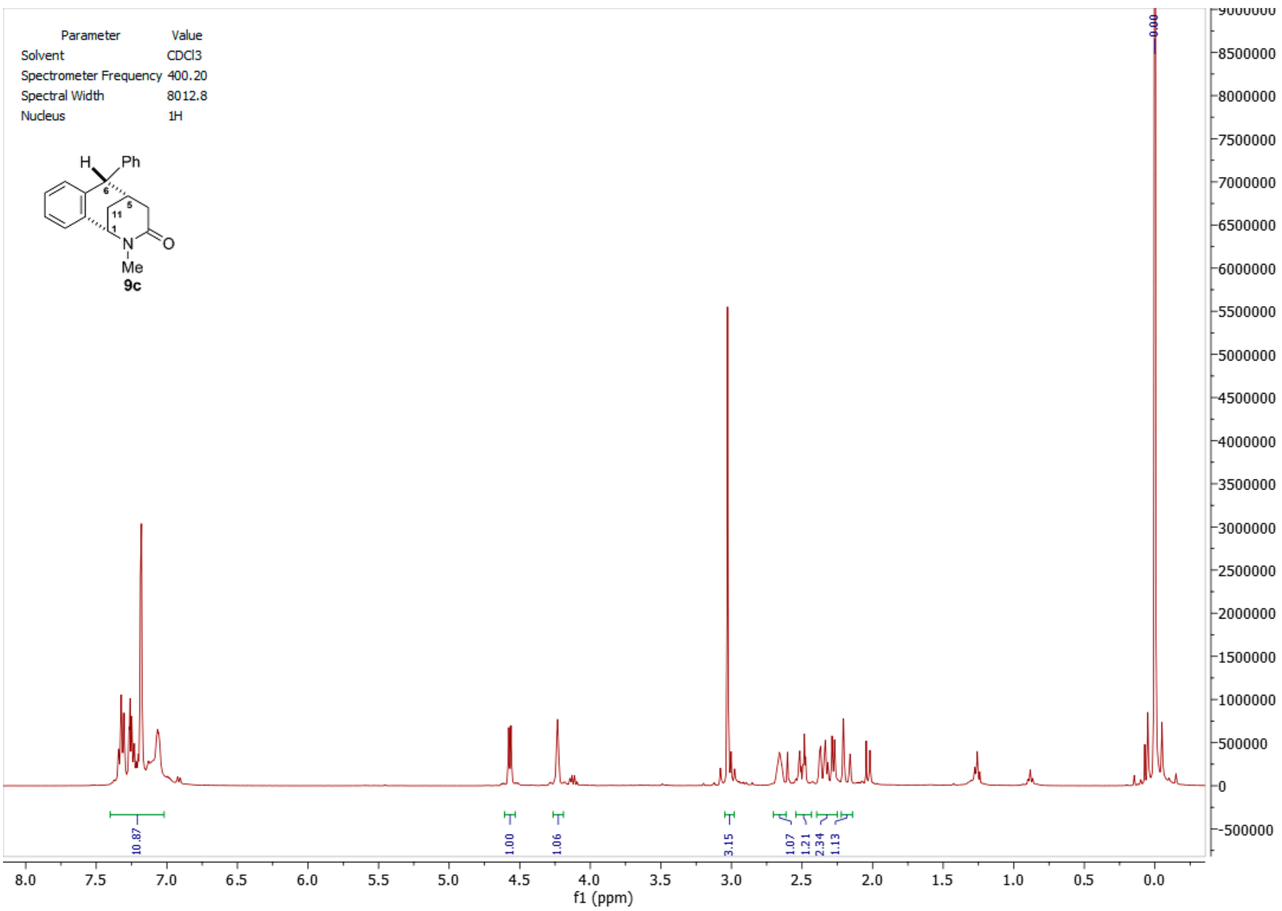
Parameter Value
Solvent CDCl3
Spectrometer Frequency 400.20
Spectral Width 8012.8
Nucleus 1H
9c
f1 (ppm)

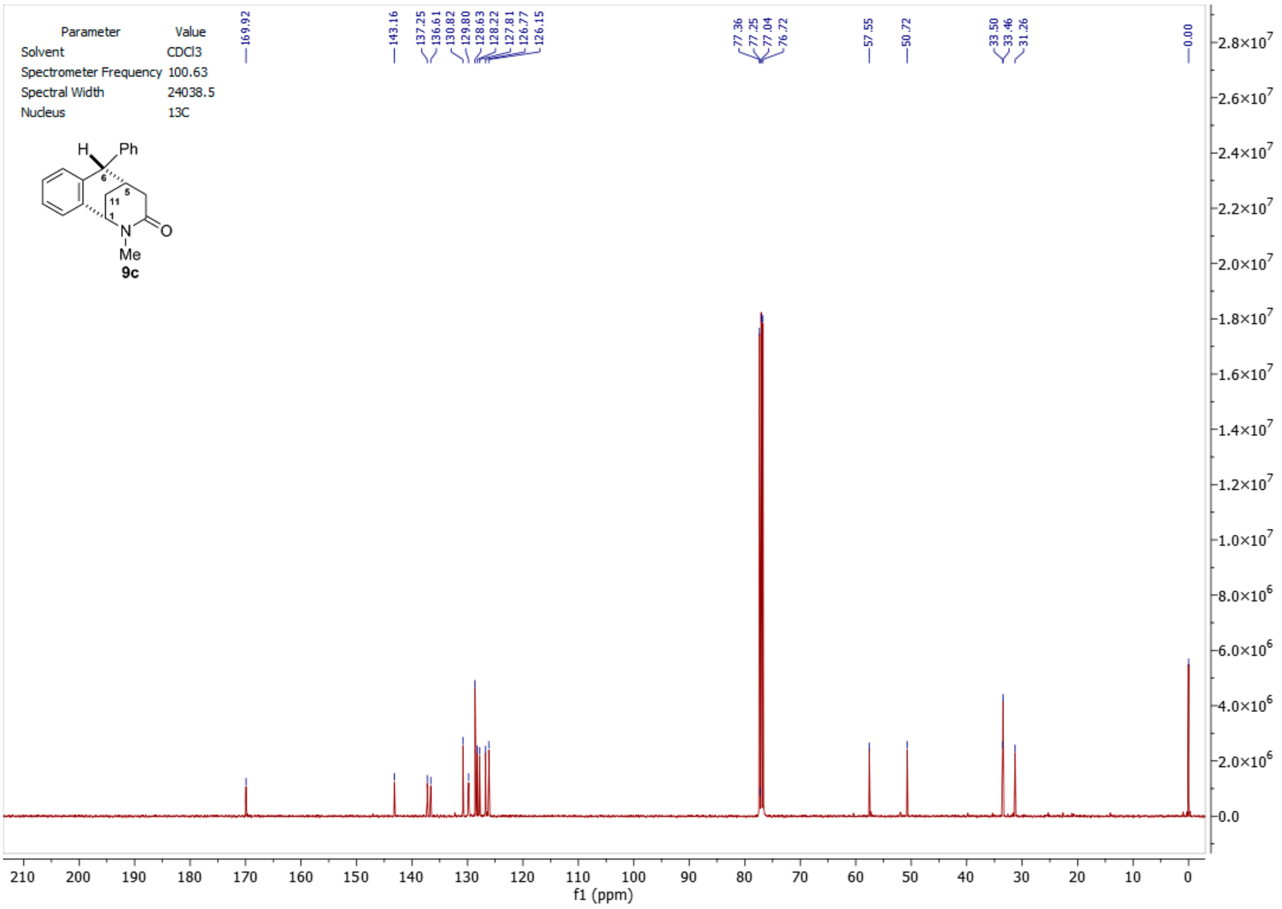
Parameter Value
Solvent CDCl3
Spectrometer Frequency 100.63
Spectral Width 24038.5
Nucleus 13C
9c
f1 (ppm)

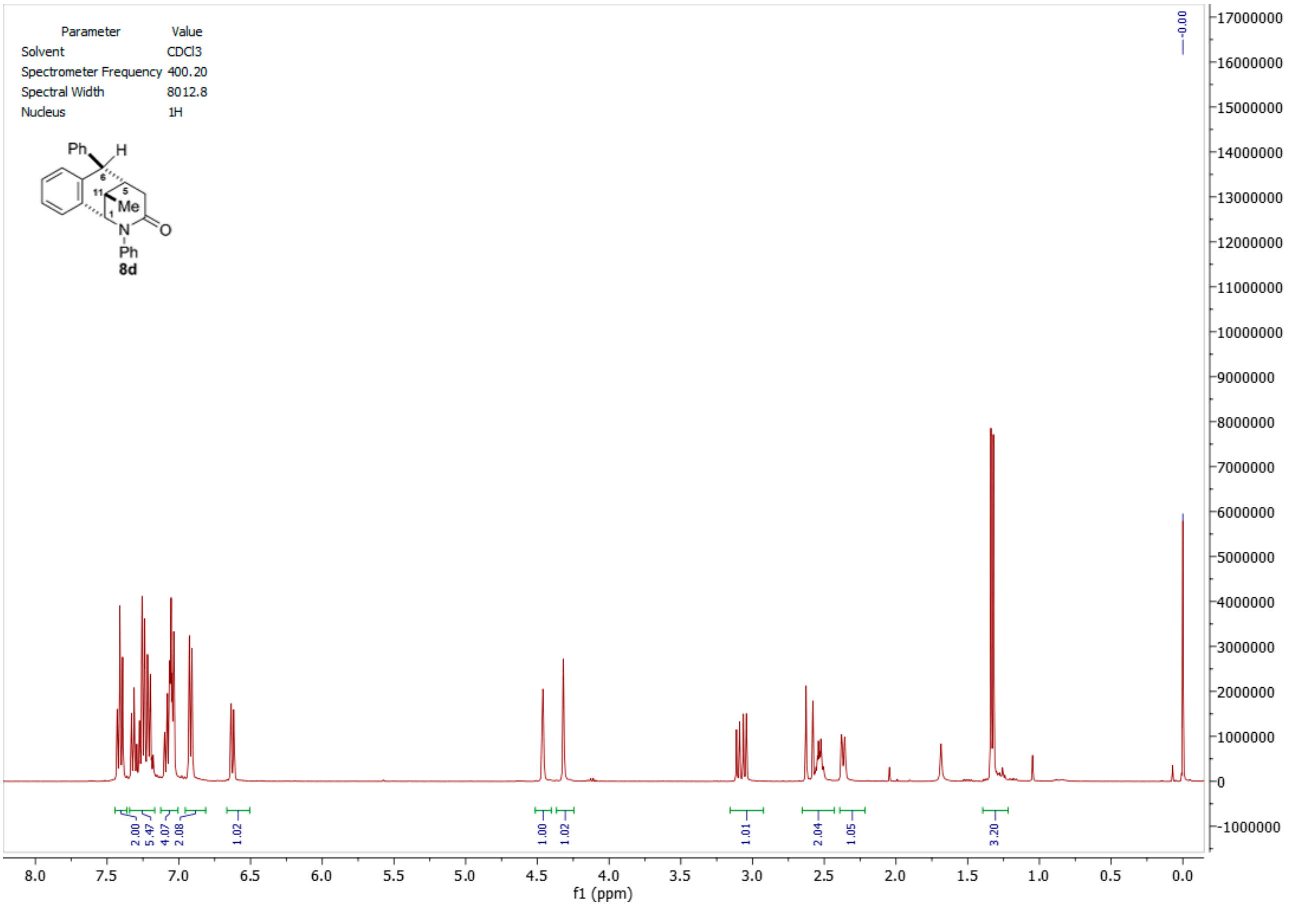
Parameter Value
Solvent CDCl3
Spectrometer Frequency 400.20
Spectral Width 8012.8
Nucleus 1H
Ph
H
Me
N
O
Ph
8d
0.00
2.00
5.47
4.07
2.08
1.02
1.00
1.02
1.01
2.04
1.05
3.20
f1 (ppm)

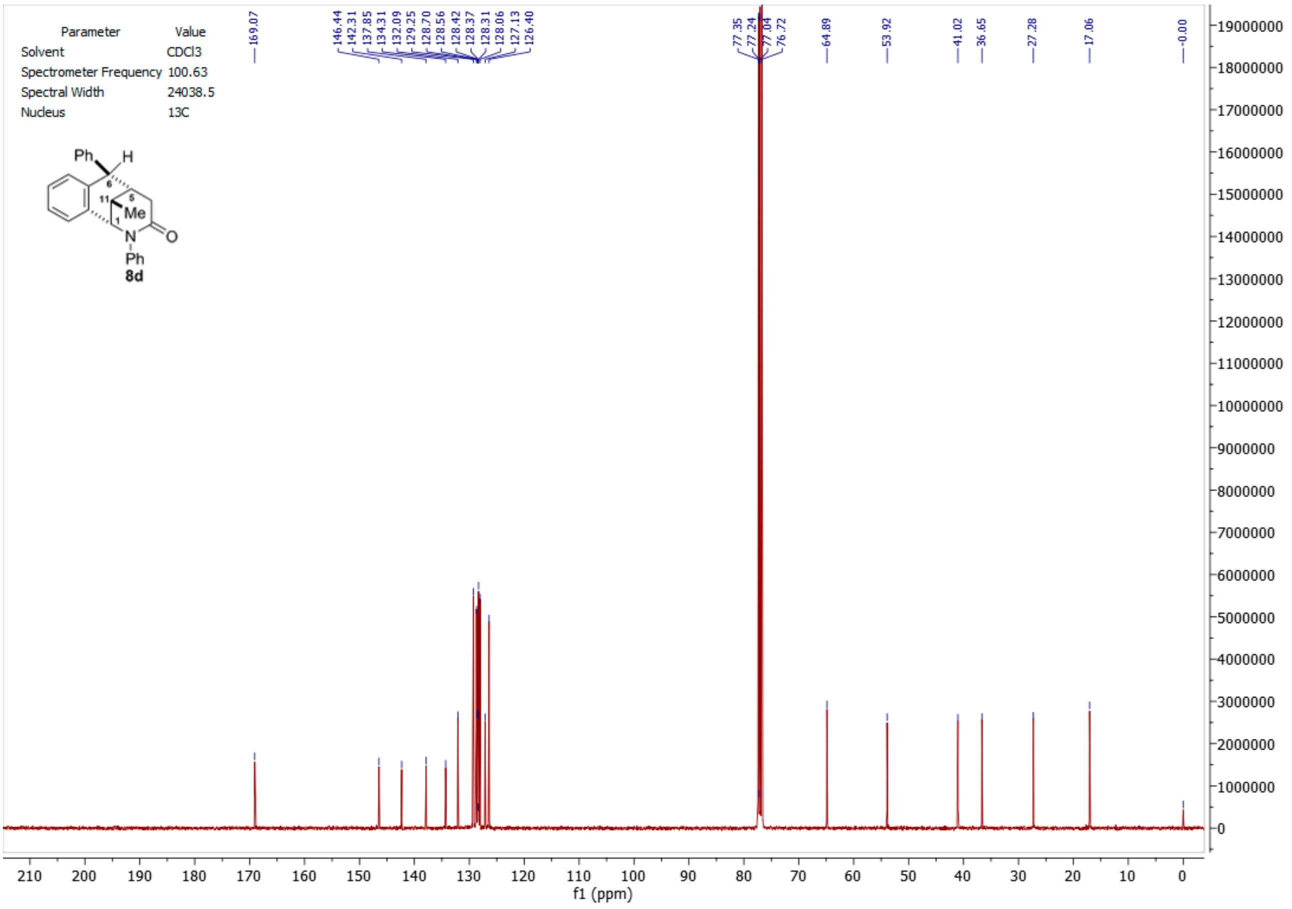
Parameter Value
Solvent CDCl3
Spectrometer Frequency 100.63
Spectral Width 24038.5
Nucleus 13C
Ph
H
Me
N
O
Ph
8d
169.07
146.44
142.31
137.85
134.31
132.09
129.25
128.70
128.56
128.42
128.37
128.31
128.06
127.13
126.40
77.35
77.24
77.04
76.72
64.89
53.92
41.02
36.65
27.28
17.06
0.00
f1 (ppm)

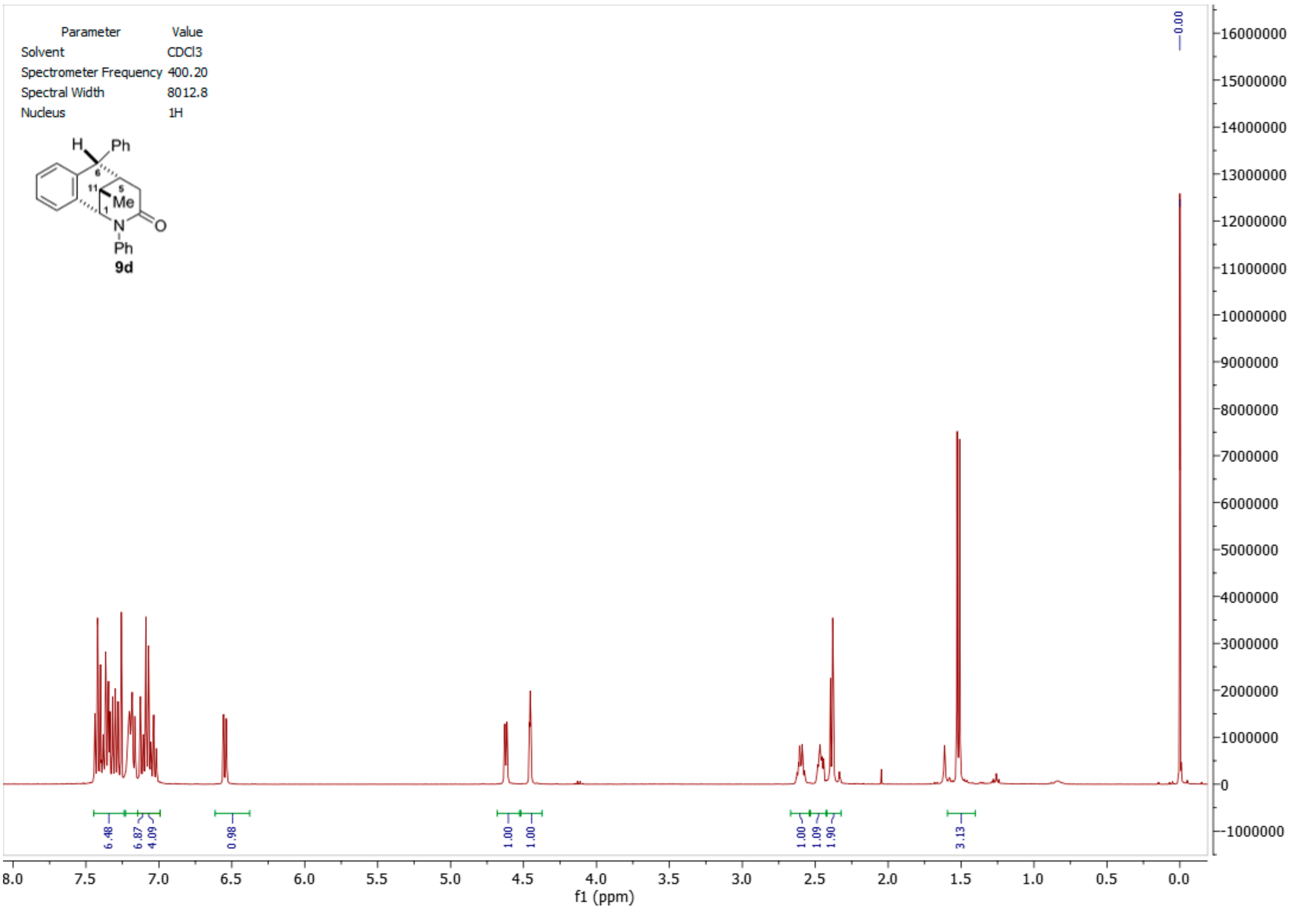
Parameter Value
Solvent CDCl3
Spectrometer Frequency 400.20
Spectral Width 8012.8
Nucleus 1H
H
Ph
6
11
5
Me
1
N
O
Ph
9d
0.00
6.48
6.87
4.09
0.98
1.00
1.00
1.00
1.09
1.90
3.13
f1 (ppm)

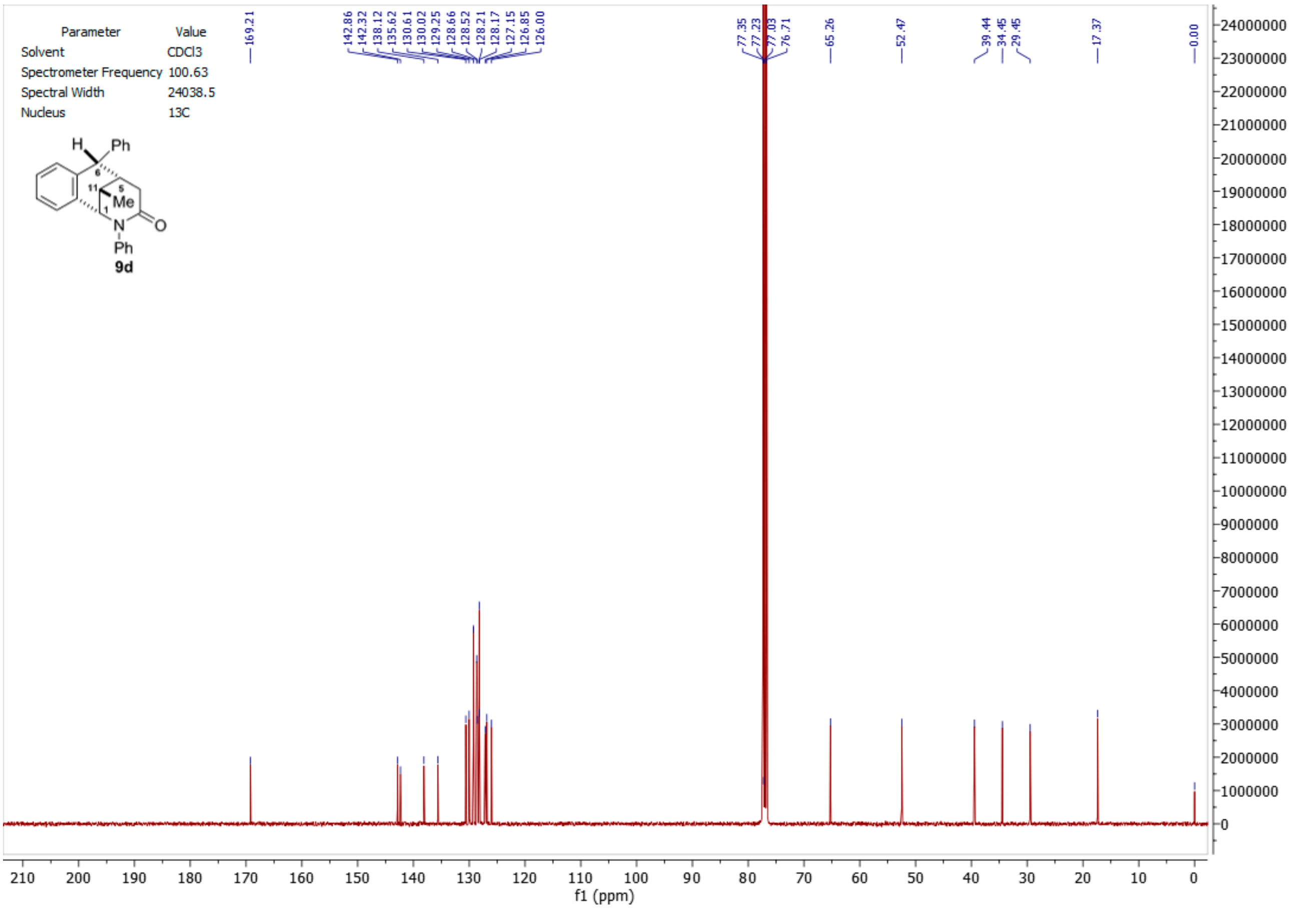
Parameter Value
Solvent CDCl3
Spectrometer Frequency 100.63
Spectral Width 24038.5
Nucleus 13C
H
Ph
6
11
5
Me
1
N
O
Ph
9d
169.21
142.86
142.32
138.12
135.62
130.61
130.02
129.25
128.66
128.52
128.21
128.17
127.15
126.85
126.00
77.35
77.23
77.03
76.71
65.26
52.47
39.44
34.45
29.45
17.37
0.00
f1 (ppm)

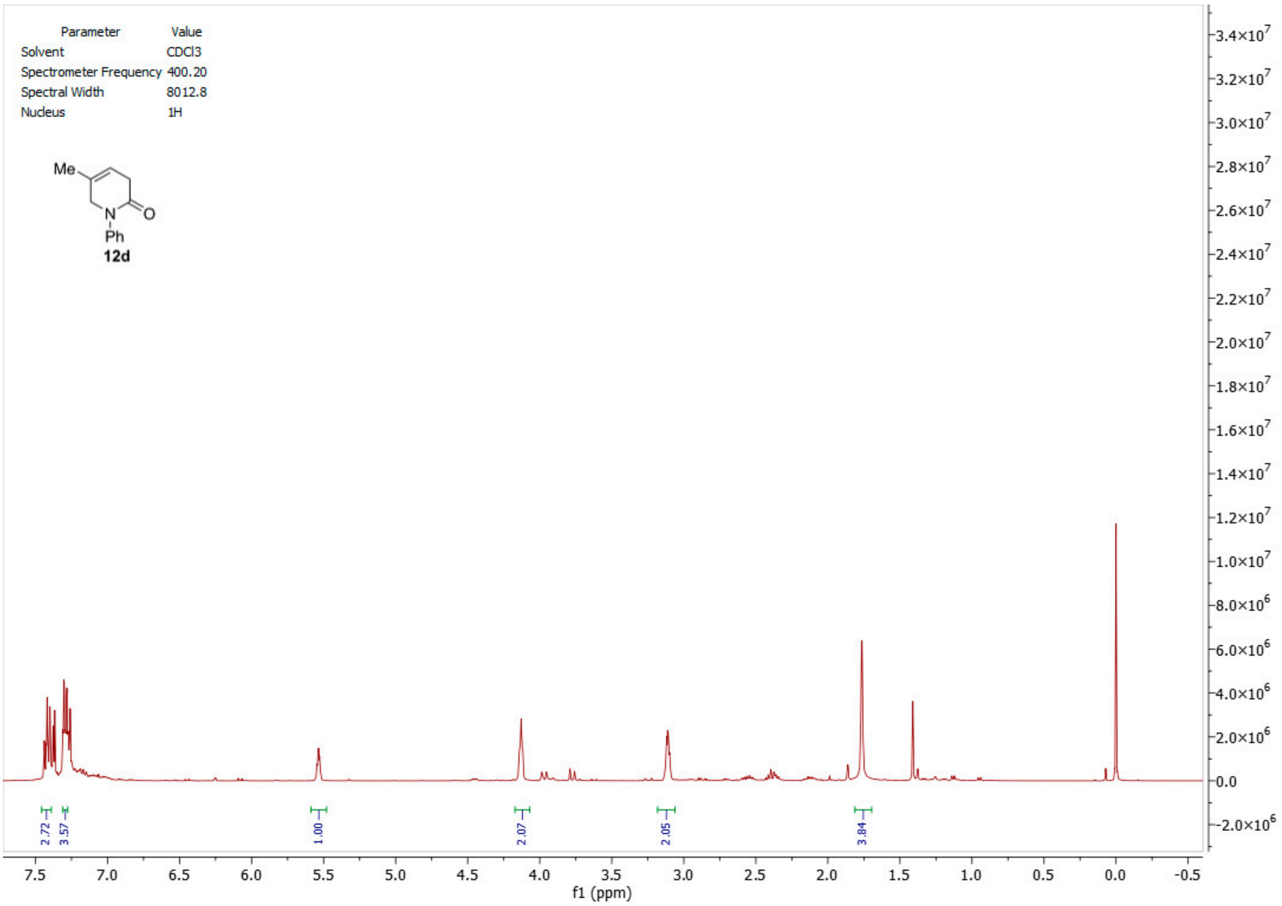
Parameter Value
Solvent CDCl3
Spectrometer Frequency 400.20
Spectral Width 8012.8
Nucleus 1H
Me
N
O
Ph
12d
2.72
3.57
1.00
2.07
2.05
3.84
f1 (ppm)

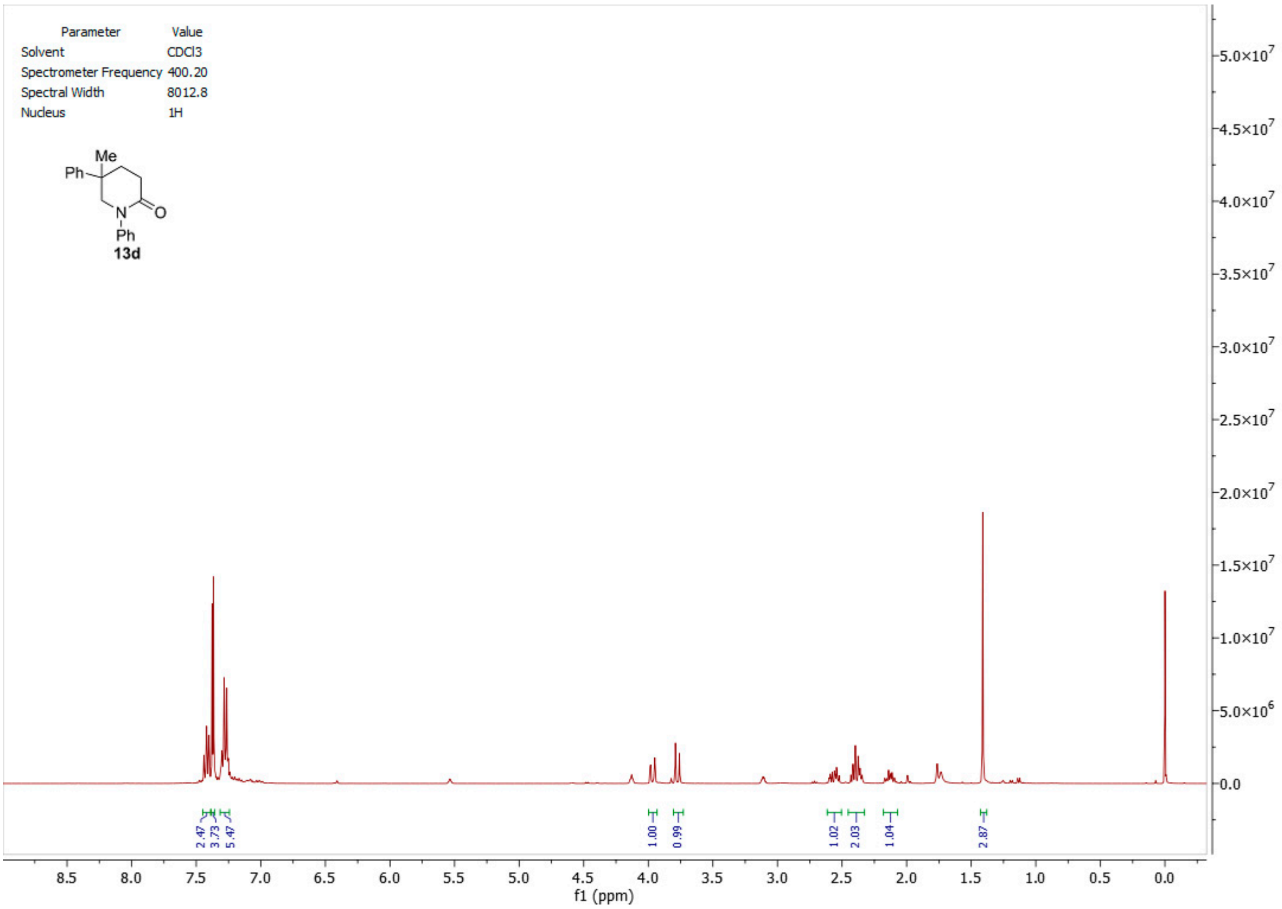
Parameter Value
Solvent CDCl3
Spectrometer Frequency 400.20
Spectral Width 8012.8
Nucleus 1H
Me
Ph
N
O
Ph
13d
2.47
3.73
5.47
1.00
0.99
1.02
2.03
1.04
2.87
f1 (ppm)

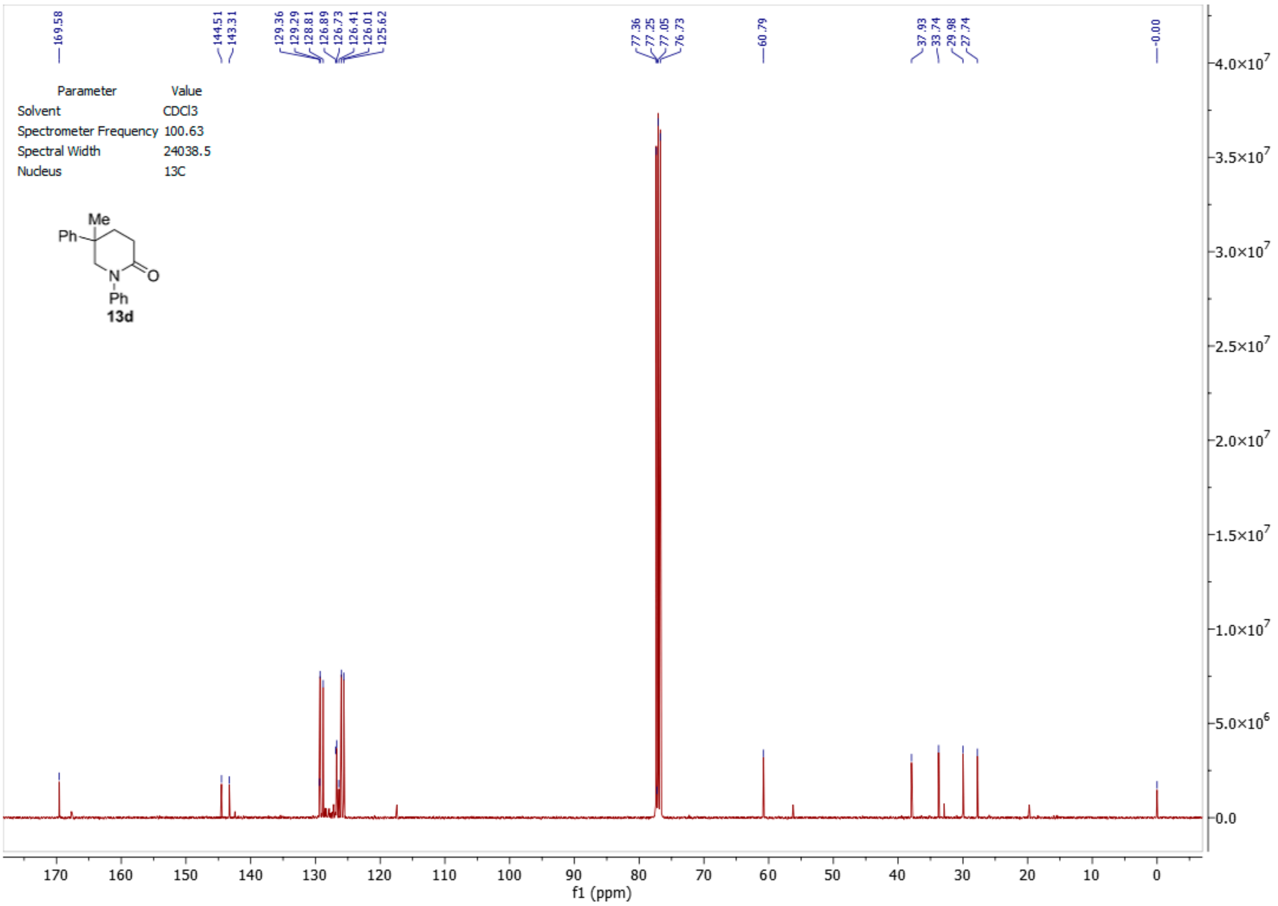

169.58
144.51
143.31
129.36
129.29
128.81
126.89
126.73
126.41
126.01
125.62
77.36
77.25
77.05
76.73
60.79
37.93
33.74
29.98
27.74
0.00
Parameter Value
Solvent CDCl3
Spectrometer Frequency 100.63
Spectral Width 24038.5
Nucleus 13C
Me
Ph
N
O
Ph
13d
f1 (ppm)

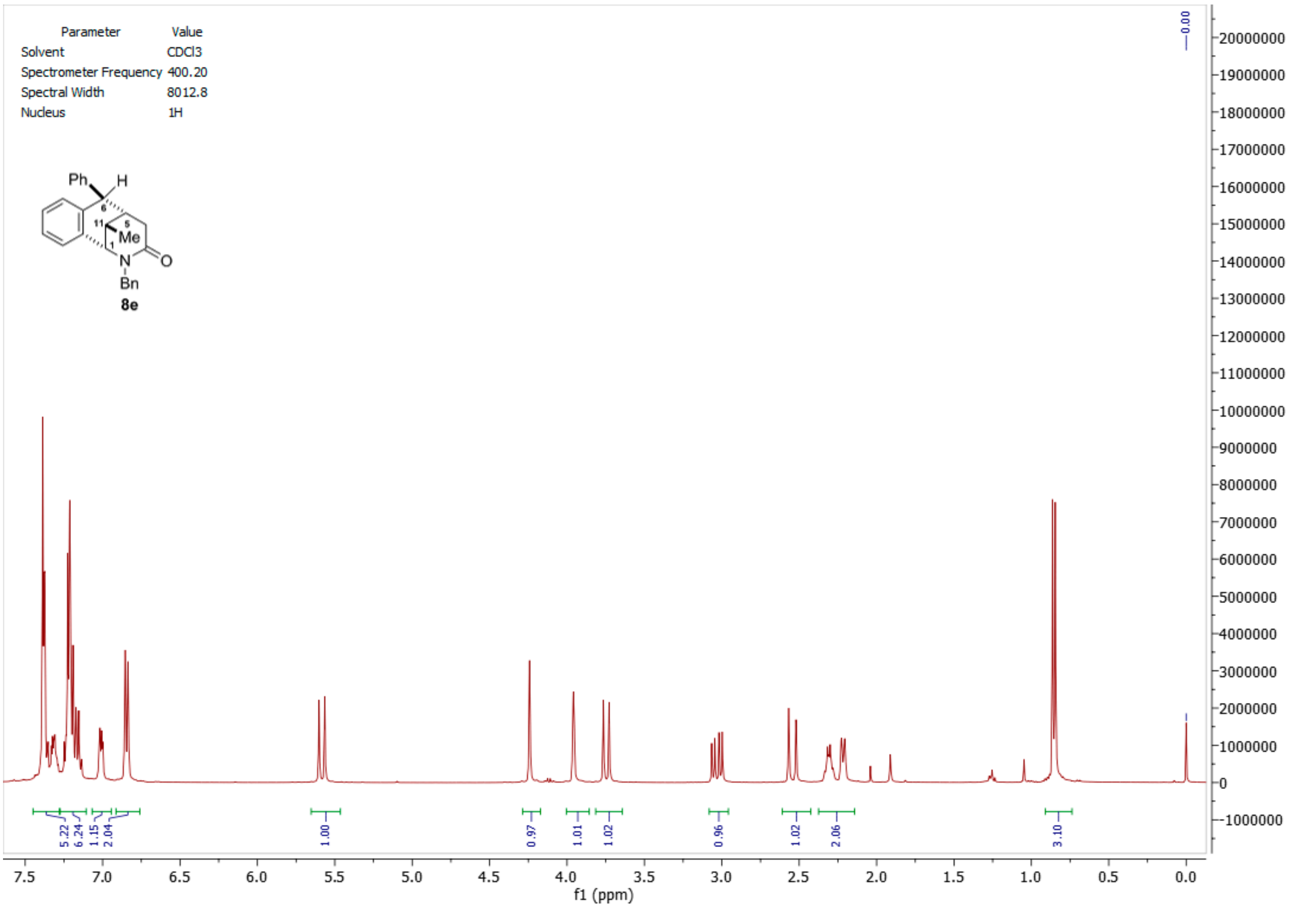

0.00
Parameter Value
Solvent CDCl3
Spectrometer Frequency 400.20
Spectral Width 8012.8
Nucleus 1H
Ph
H
6
11
5
1
Me
N
O
Bn
8e
5.22
6.24
1.15
2.04
1.00
0.97
1.01
1.02
0.96
1.02
2.06
3.10
f1 (ppm)

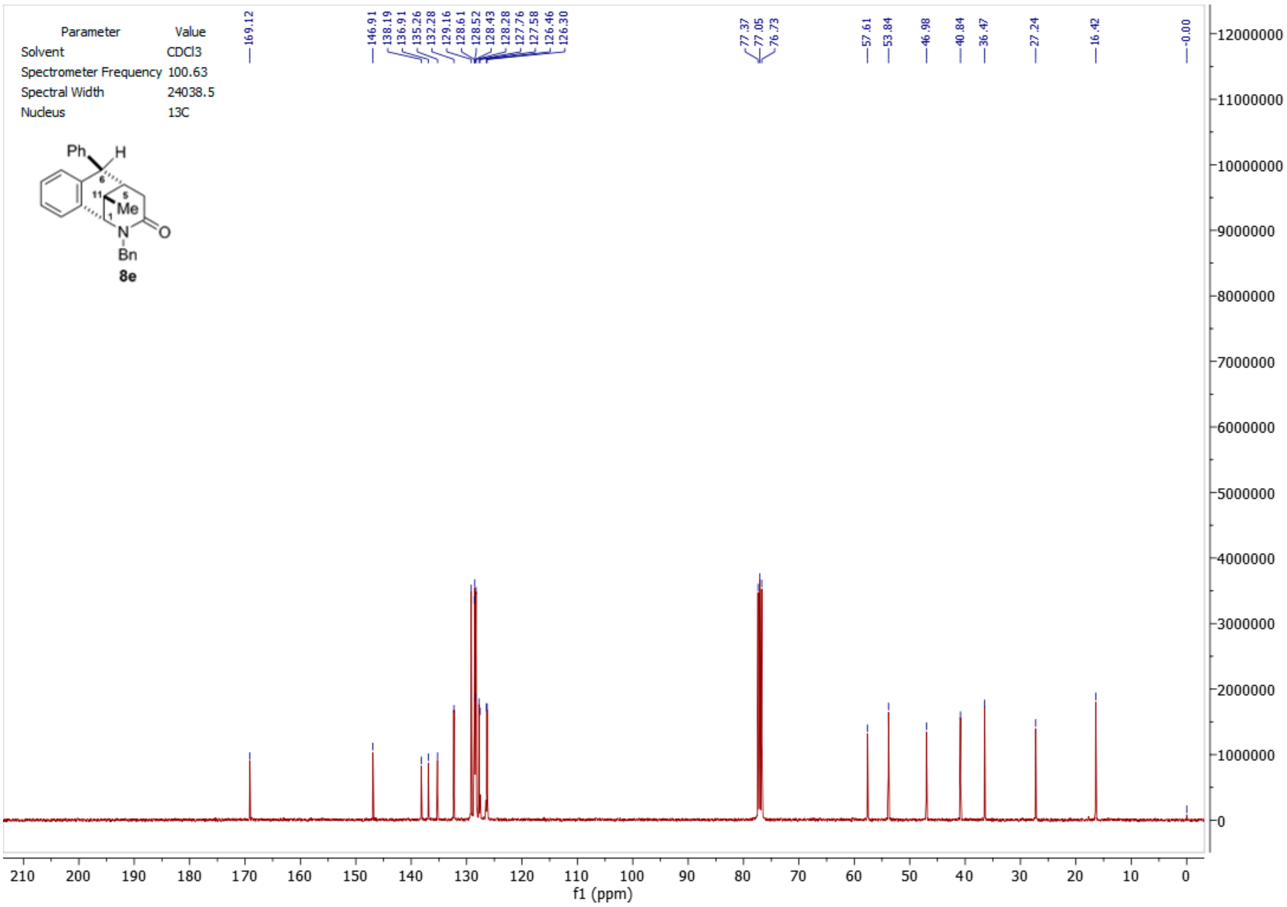
Parameter Value
Solvent CDCl3
Spectrometer Frequency 100.63
Spectral Width 24038.5
Nucleus 13C
8e
169.12
146.91
138.19
136.91
135.26
132.28
129.16
128.61
128.52
128.43
128.28
127.76
127.58
126.46
126.30
77.37
77.05
76.73
57.61
53.84
46.98
40.84
36.47
27.24
16.42
-0.00
f1 (ppm)

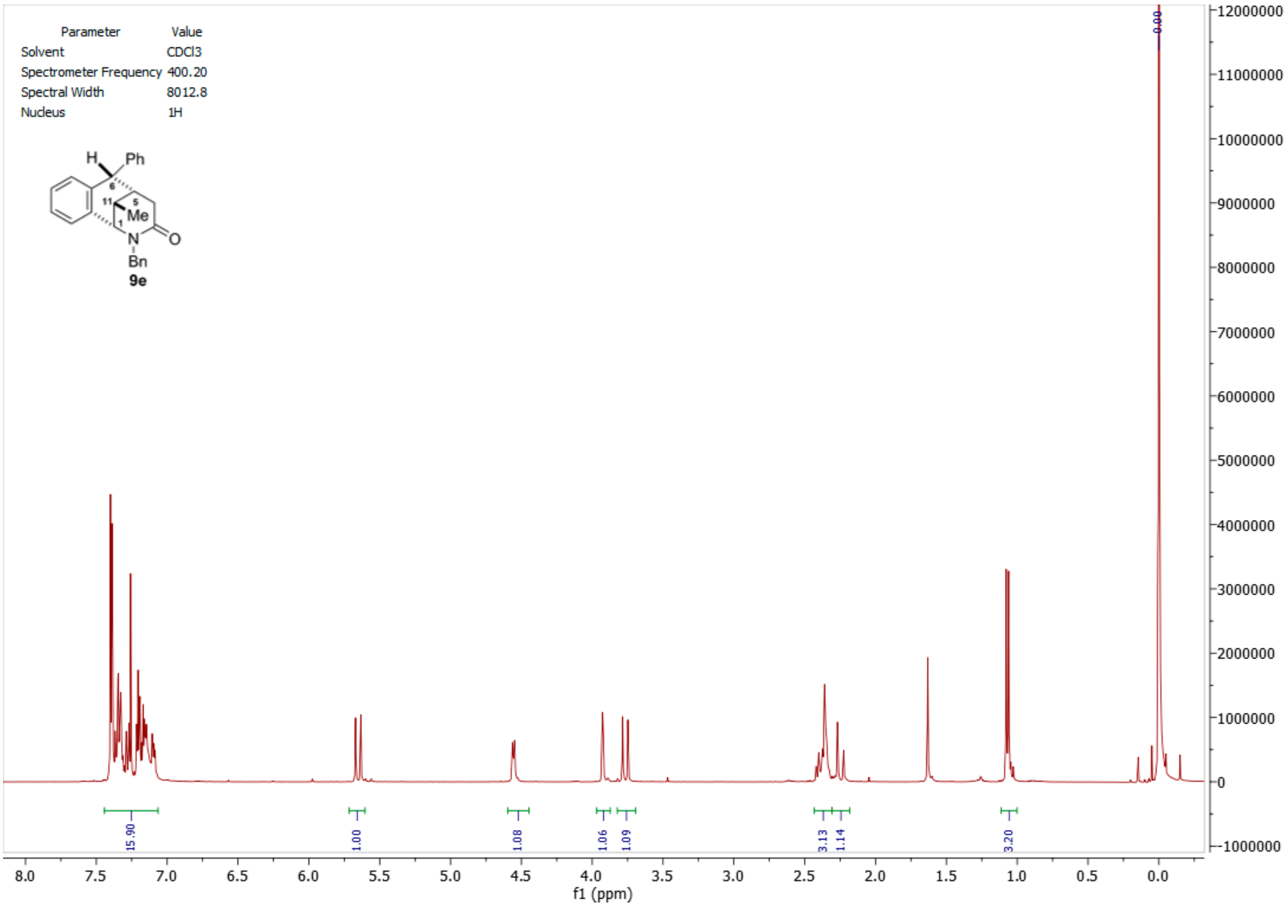
Parameter Value
Solvent CDCl3
Spectrometer Frequency 400.20
Spectral Width 8012.8
Nucleus 1H
9e
0.00
15.90
1.00
1.08
1.06
1.09
3.13
1.14
3.20
f1 (ppm)

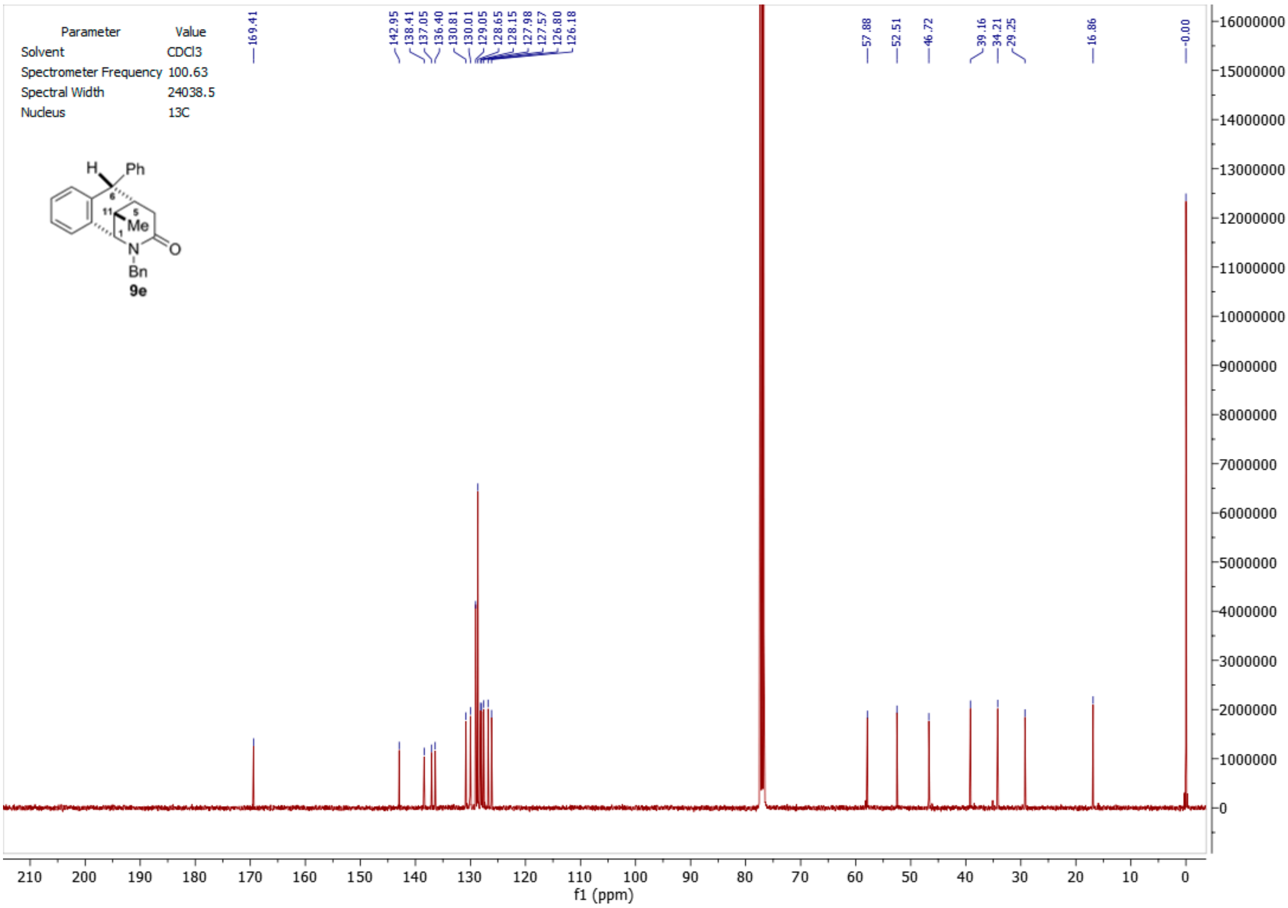
Parameter Value
Solvent CDCl3
Spectrometer Frequency 100.63
Spectral Width 24038.5
Nucleus 13C
9e
169.41
142.95
138.41
137.05
136.40
130.81
130.01
129.05
128.65
128.15
127.98
127.57
126.80
126.18
57.88
52.51
46.72
39.16
34.21
29.25
16.86
-0.00
f1 (ppm)

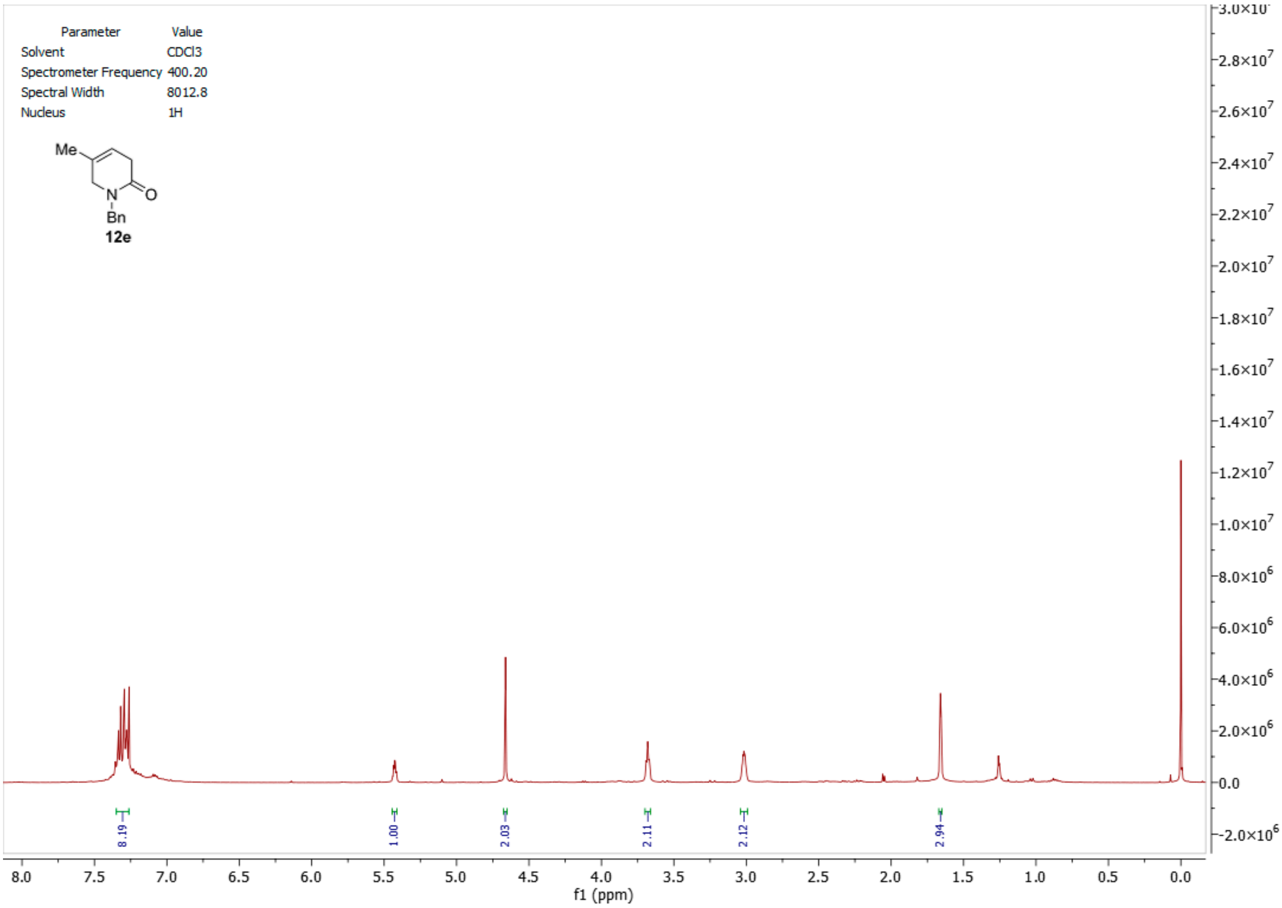
Parameter Value
Solvent CDCl3
Spectrometer Frequency 400.20
Spectral Width 8012.8
Nucleus 1H
12e
8.19
1.00
2.03
2.11
2.12
2.94
f1 (ppm)

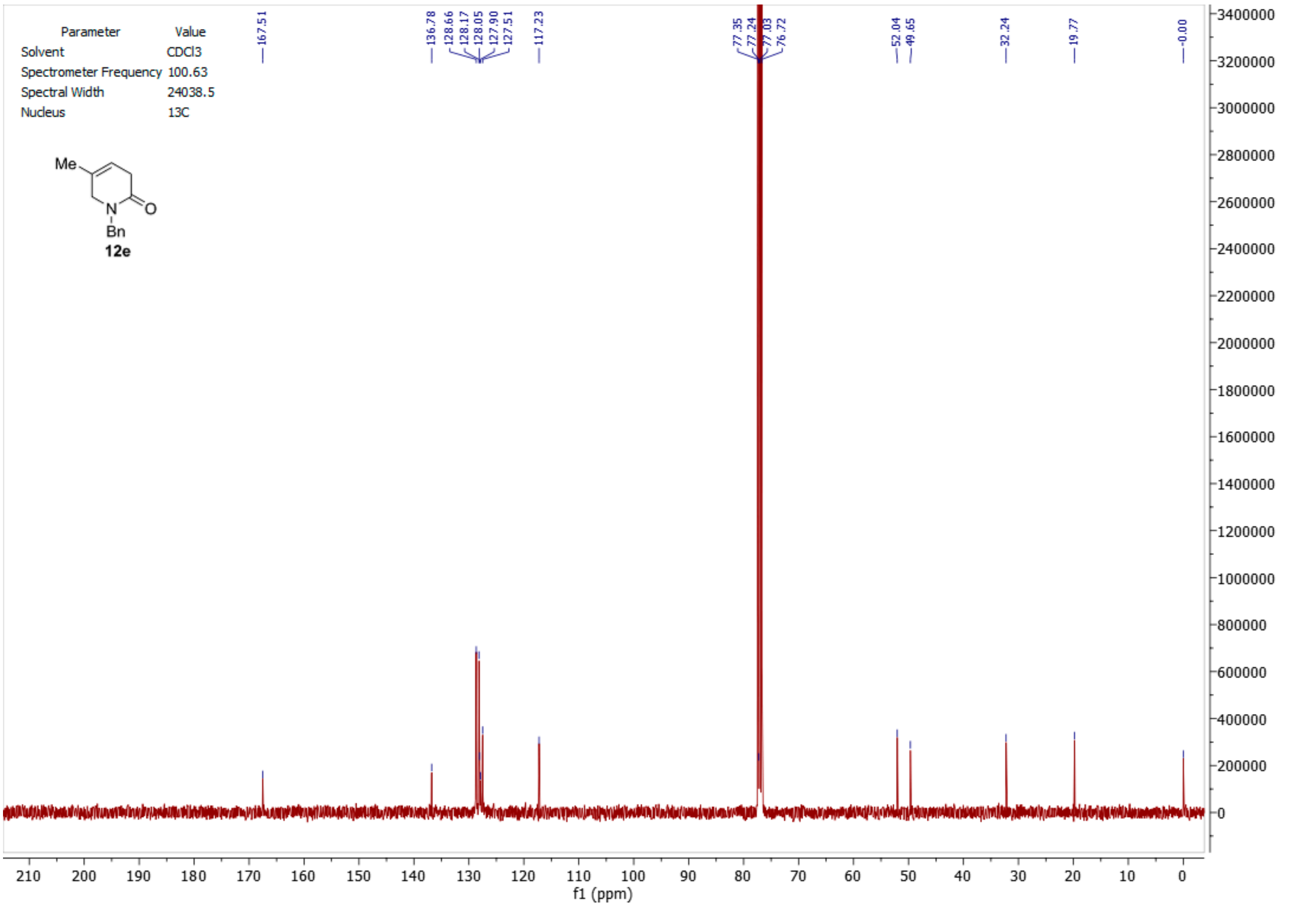
Parameter Value
Solvent CDCl3
Spectrometer Frequency 100.63
Spectral Width 24038.5
Nucleus 13C
Me
N
O
Bn
12e
167.51
136.78
128.66
128.17
128.05
127.90
127.51
117.23
77.35
77.24
77.03
76.72
52.04
49.65
32.24
19.77
-0.00
f1 (ppm)

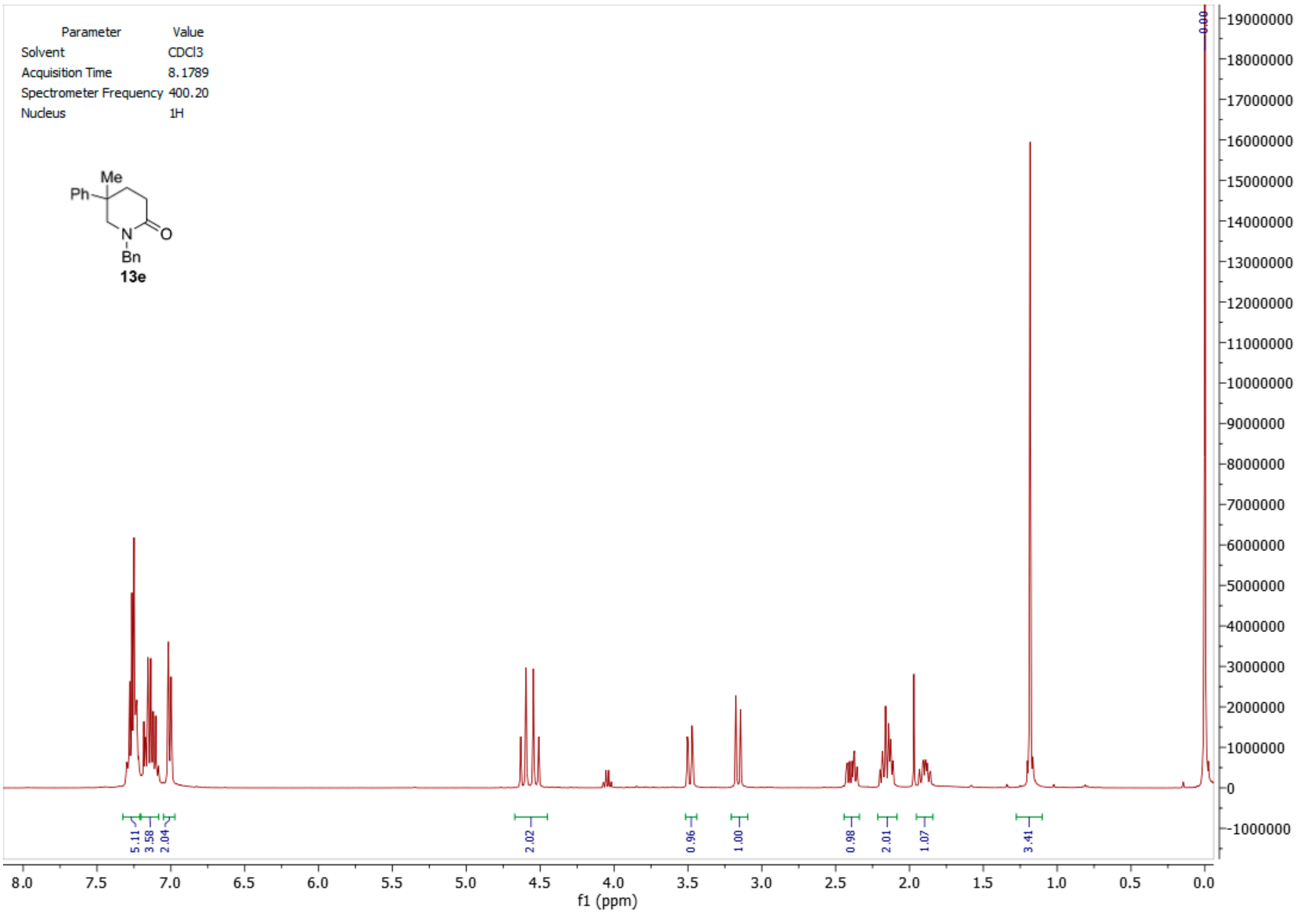
Parameter Value
Solvent CDCl3
Acquisition Time 8.1789
Spectrometer Frequency 400.20
Nucleus 1H
Me
Ph
N
O
Bn
13e
0.00
5.11
3.58
2.04
2.02
0.96
1.00
0.98
2.01
1.07
3.41
f1 (ppm)

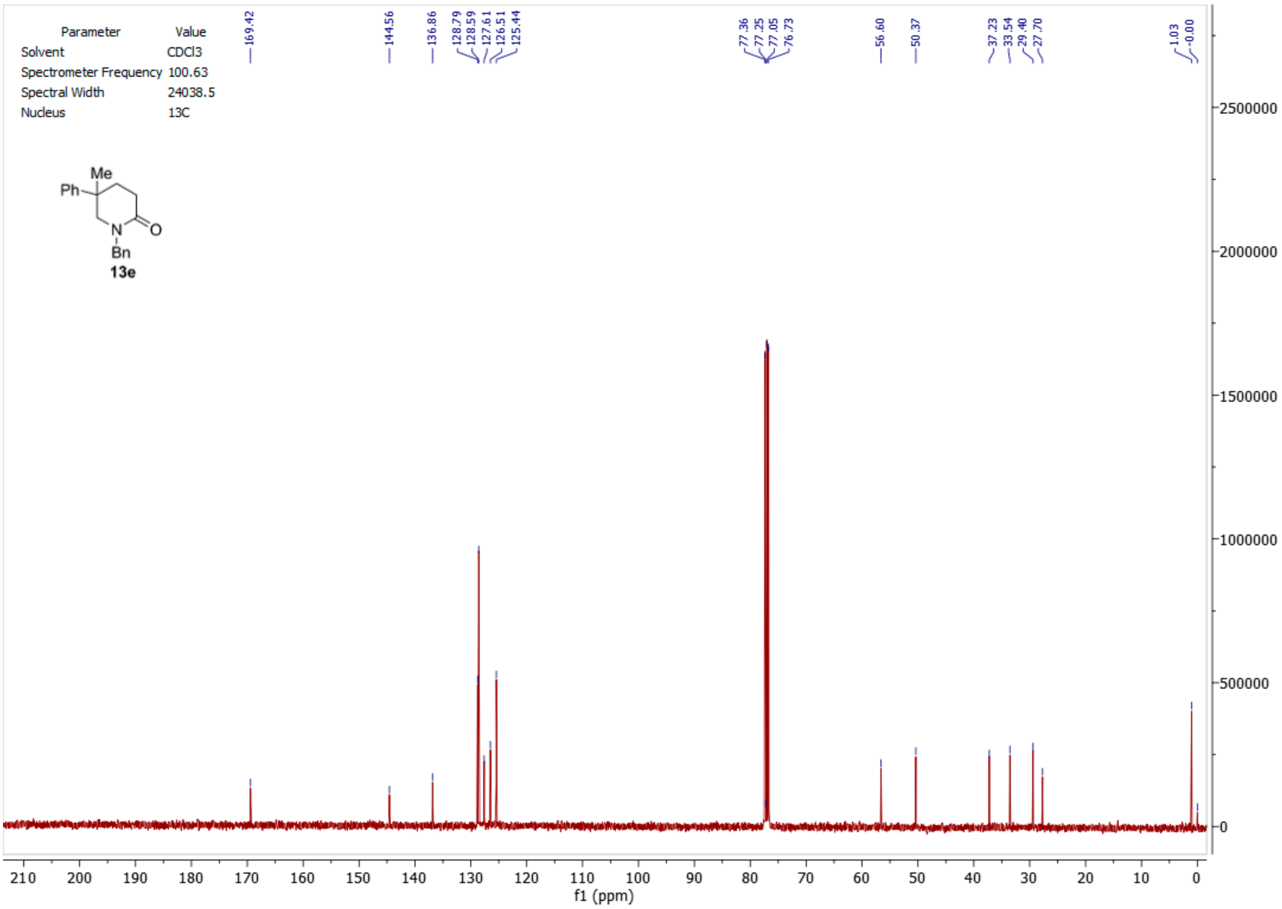

Parameter Value
Solvent CDCl3
Spectrometer Frequency 100.63
Spectral Width 24038.5
Nucleus 13C
Me
Ph
N
O
Bn
13e
169.42
144.56
136.86
128.79
128.59
127.61
126.51
125.44
77.36
77.25
77.05
76.73
56.60
50.37
37.23
33.54
29.40
27.70
1.03
-0.00
f1 (ppm)

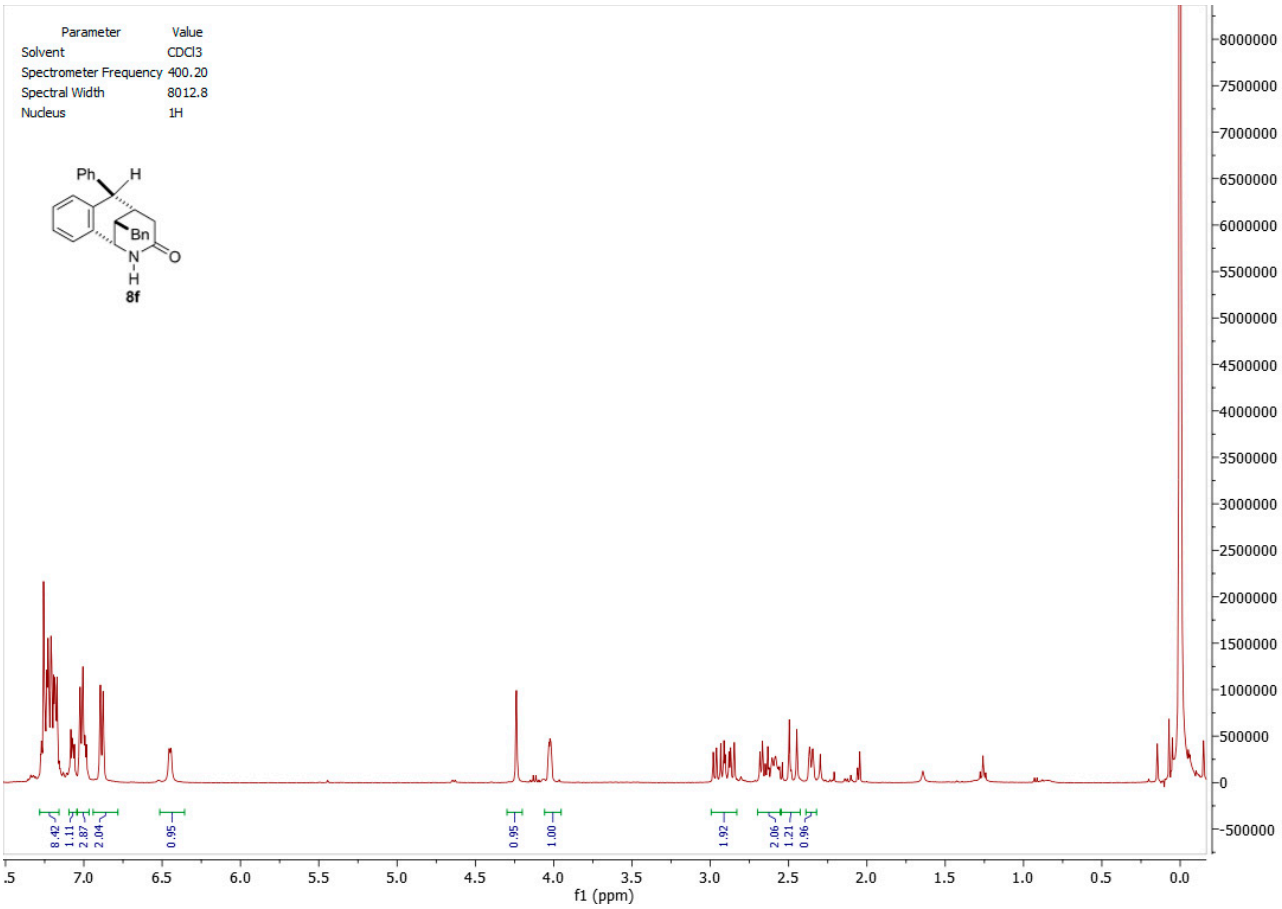

Parameter Value
Solvent CDCl3
Spectrometer Frequency 400.20
Spectral Width 8012.8
Nucleus 1H
Ph
H
Bn
N
H
O
8f
8.42
1.11
2.87
2.04
0.95
0.95
1.00
1.92
2.06
1.21
0.96
f1 (ppm)

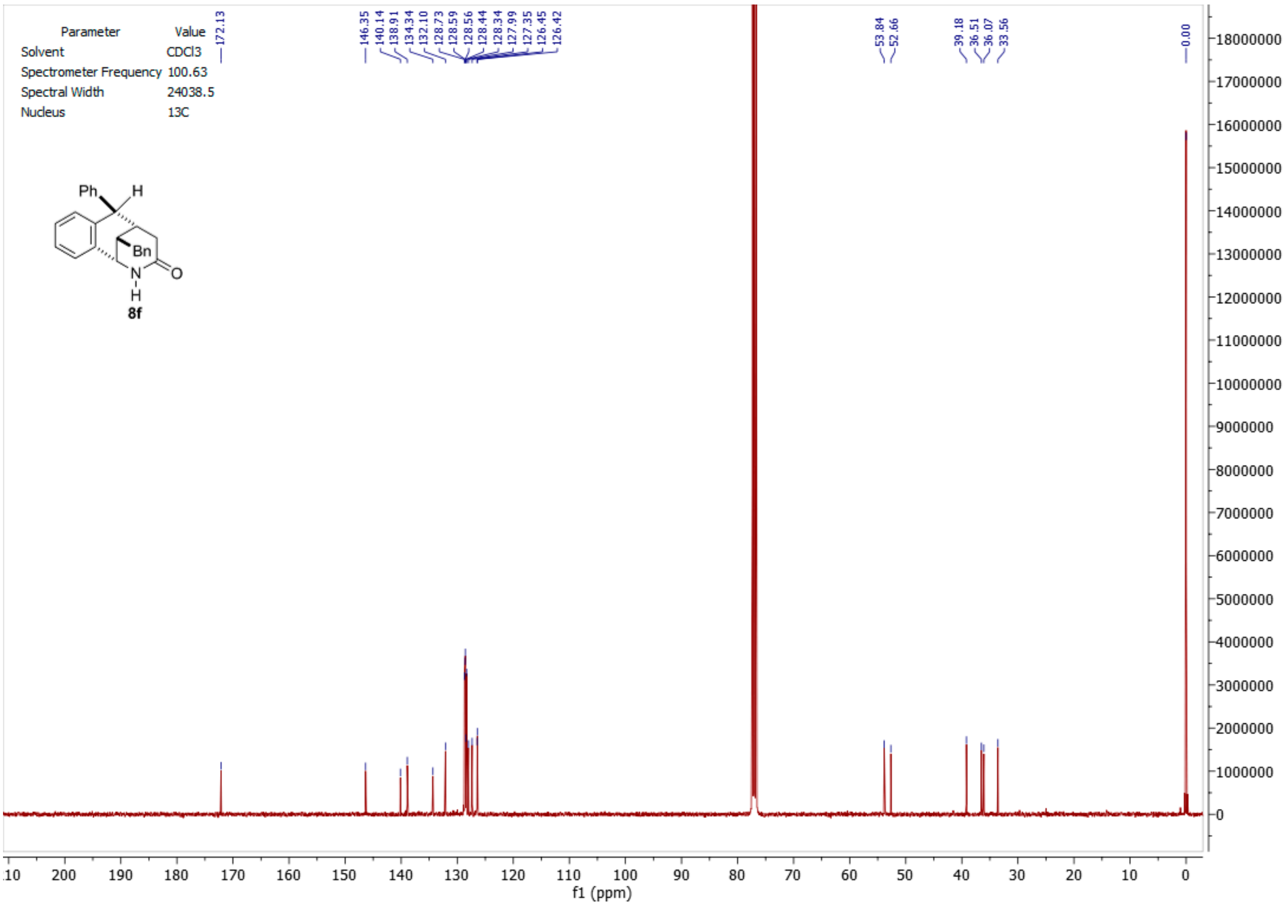

Parameter Value
Solvent CDCl3
Spectrometer Frequency 100.63
Spectral Width 24038.5
Nucleus 13C
Ph
H
Bn
N
H
O
8f
172.13
146.35
140.14
138.91
134.34
132.10
128.73
128.59
128.56
128.44
128.34
127.99
127.35
126.45
126.42
53.84
52.66
39.18
36.51
36.07
33.56
0.00
f1 (ppm)

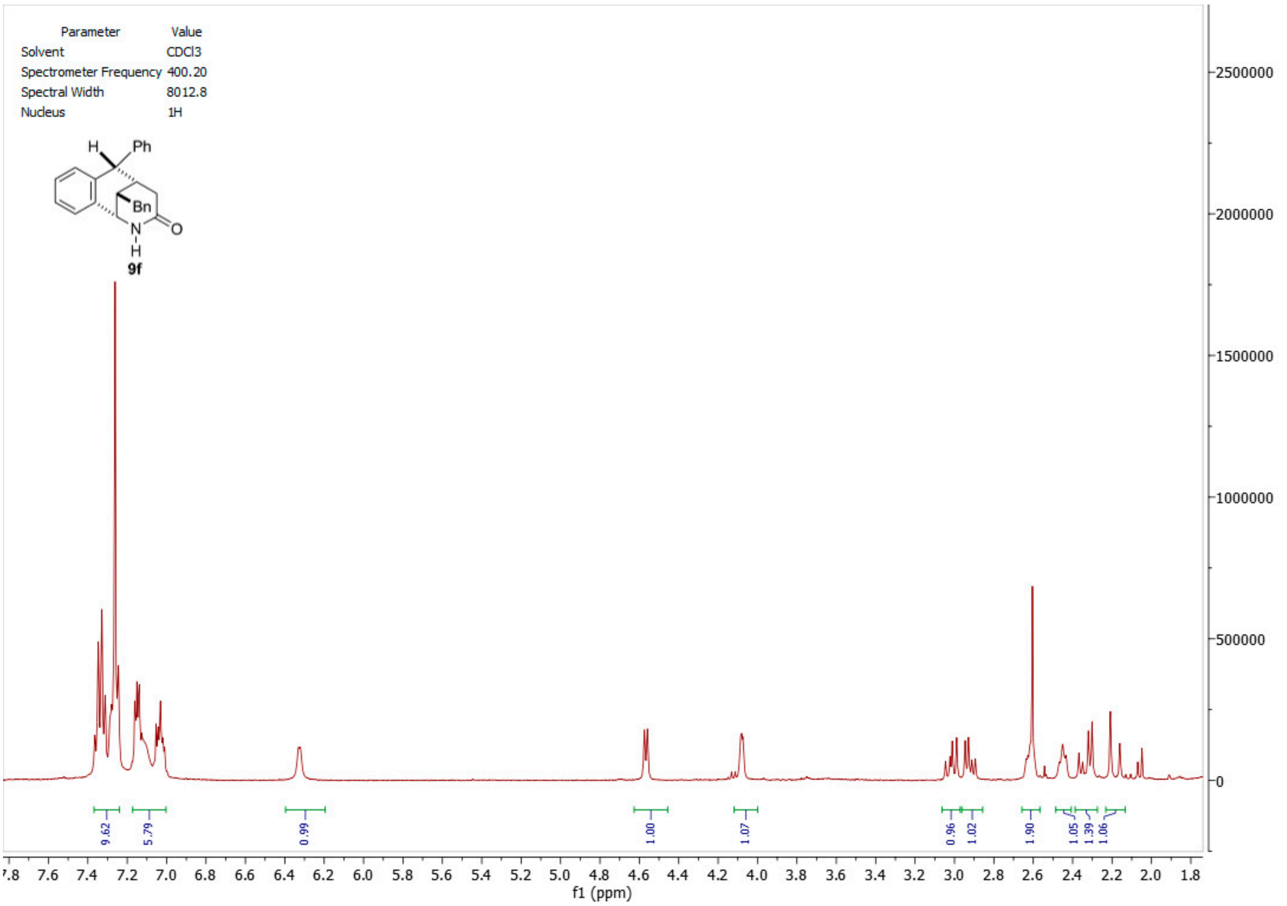

Parameter Value
Solvent CDCl3
Spectrometer Frequency 400.20
Spectral Width 8012.8
Nucleus 1H
H
Ph
Bn
N
H
O
9f
9.62
5.79
0.99
1.00
1.07
0.96
1.02
1.90
1.05
1.39
1.06
f1 (ppm)

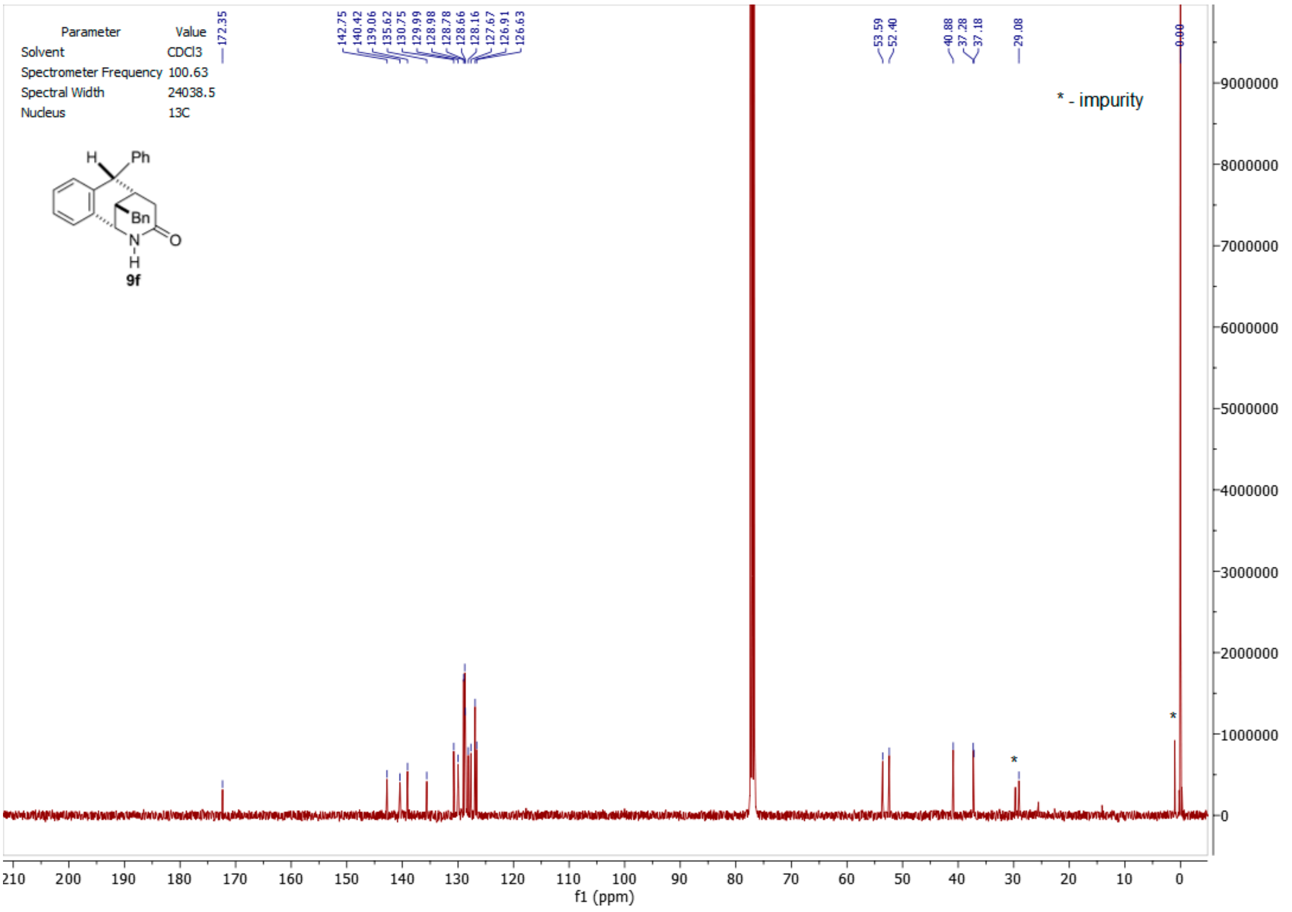
Parameter Value
Solvent CDCl3
Spectrometer Frequency 100.63
Spectral Width 24038.5
Nucleus 13C
9f
* - impurity
f1 (ppm)

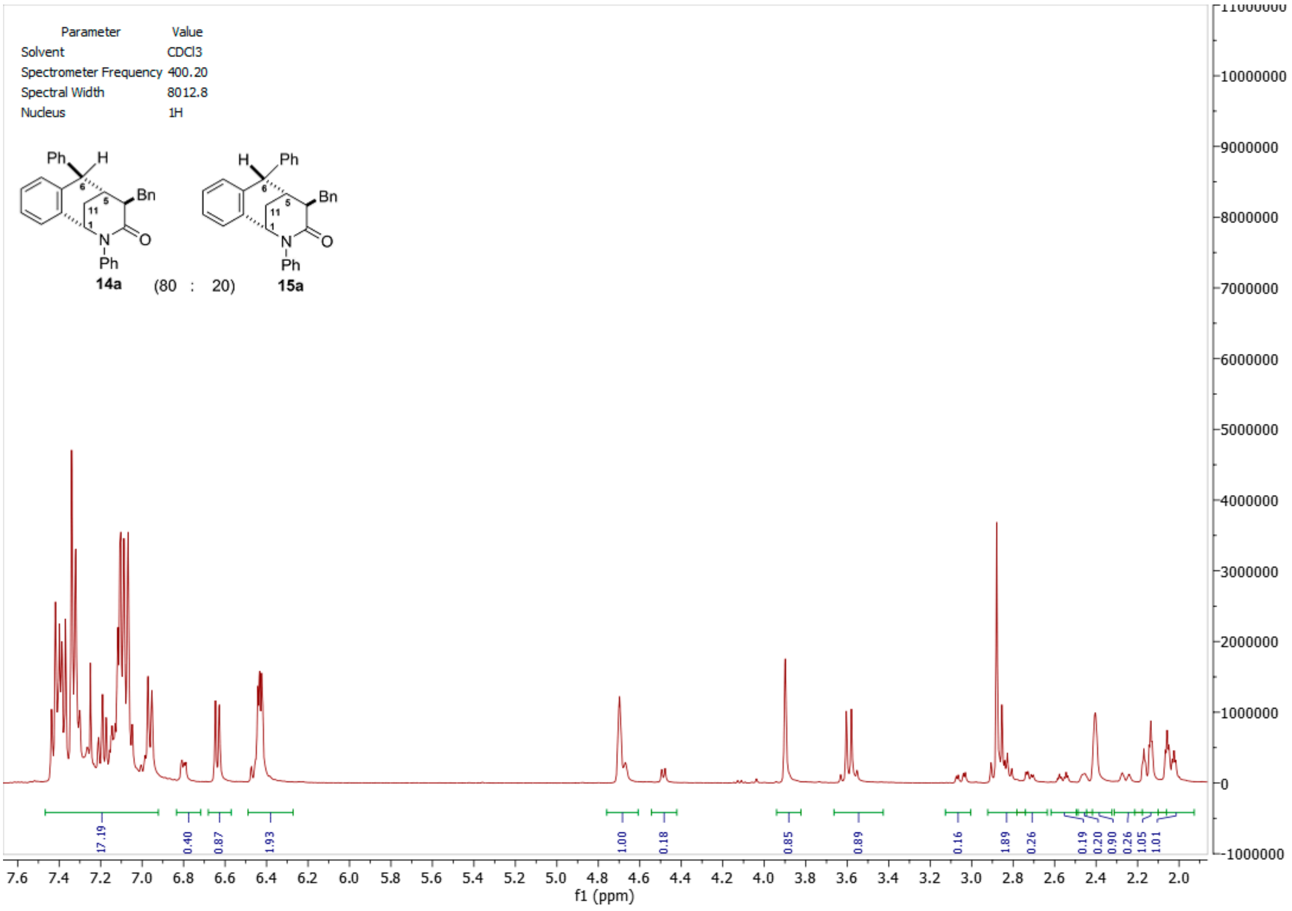
Parameter Value
Solvent CDCl3
Spectrometer Frequency 400.20
Spectral Width 8012.8
Nucleus 1H
14a
(80 : 20)
15a
f1 (ppm)

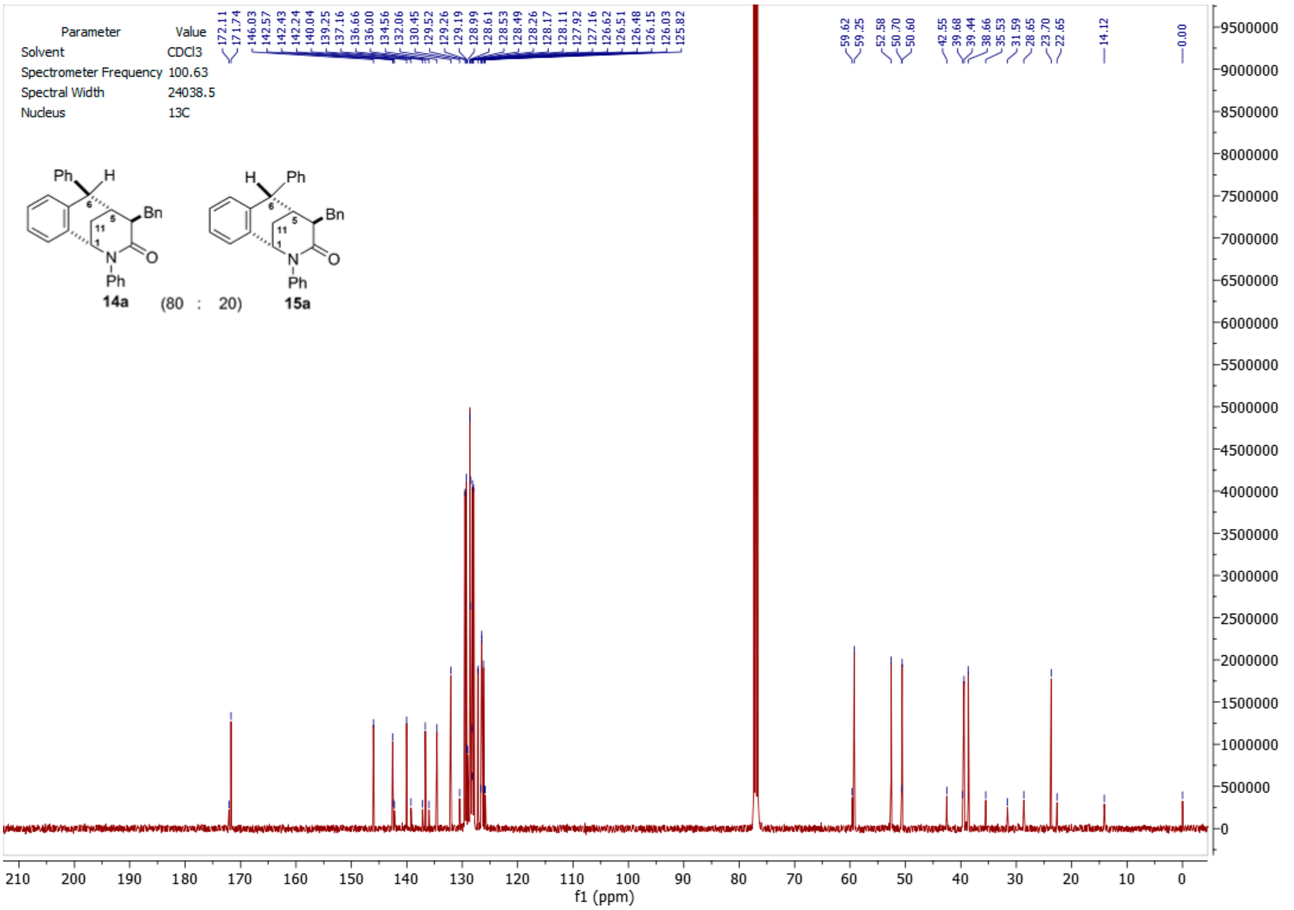

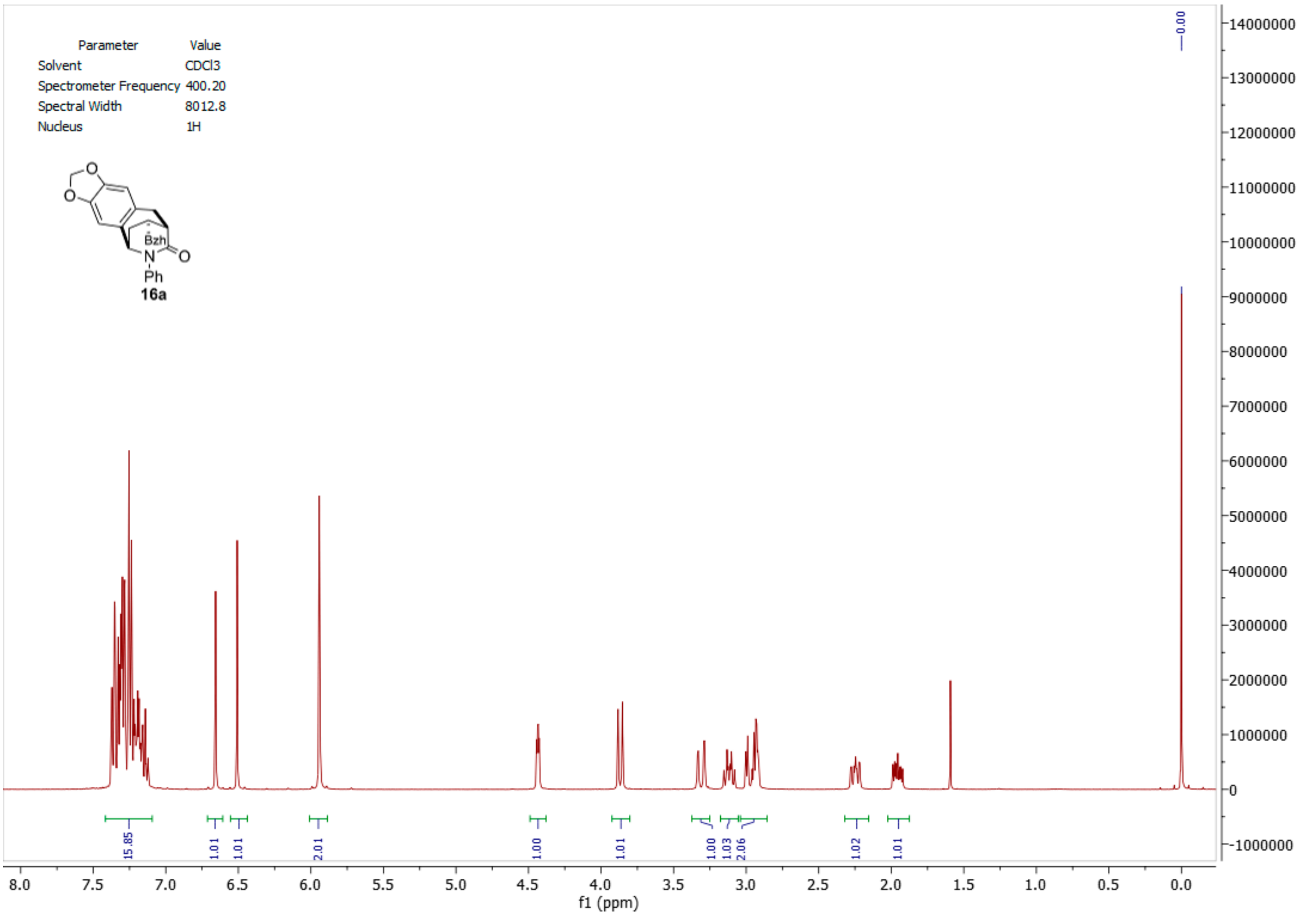

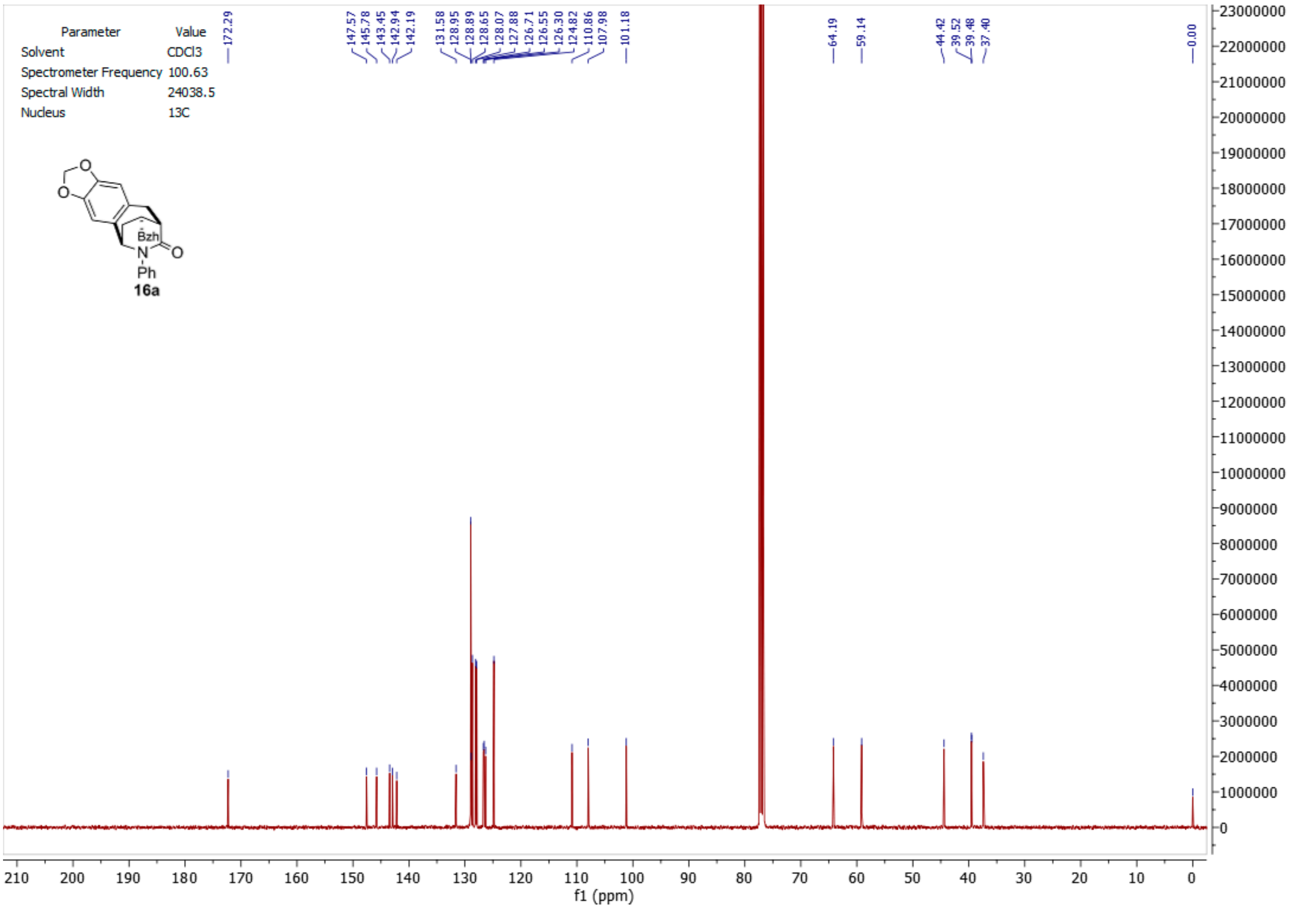
Parameter Value
Solvent CDCl3
Spectrometer Frequency 100.63
Spectral Width 24038.5
Nucleus 13C
Bzh
N
O
Ph
16a
172.29
147.57
145.78
143.45
142.94
142.19
131.58
128.95
128.89
128.65
128.07
127.88
126.71
126.55
126.30
124.82
110.86
107.98
101.18
64.19
59.14
44.42
39.52
39.48
37.40
0.00
f1 (ppm)

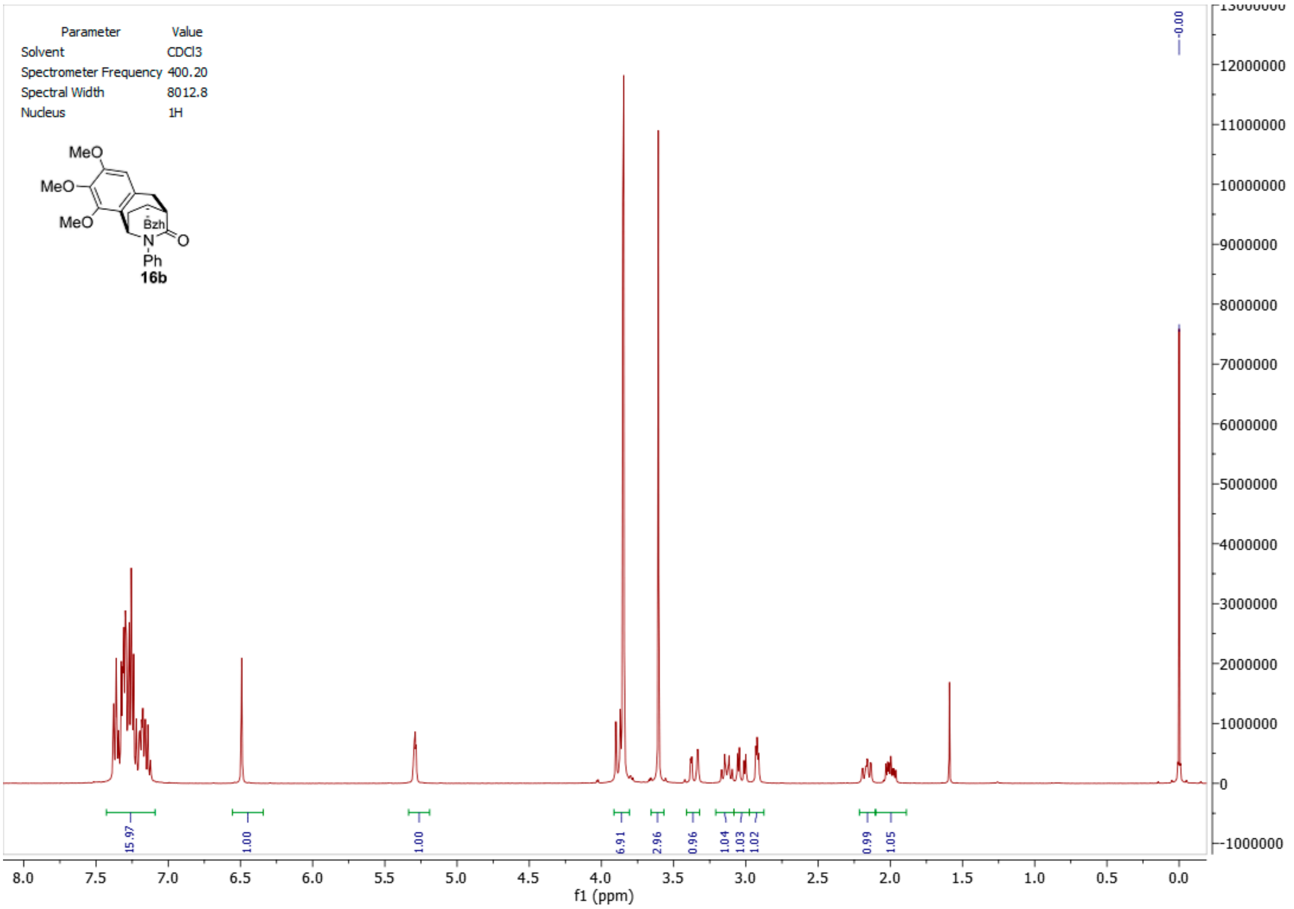
Parameter Value
Solvent CDCl3
Spectrometer Frequency 400.20
Spectral Width 8012.8
Nucleus 1H
MeO
MeO
MeO
Bzh
N
O
Ph
16b
0.00
15.97
1.00
1.00
6.91
2.96
0.96
1.04
1.03
1.02
0.99
1.05
f1 (ppm)

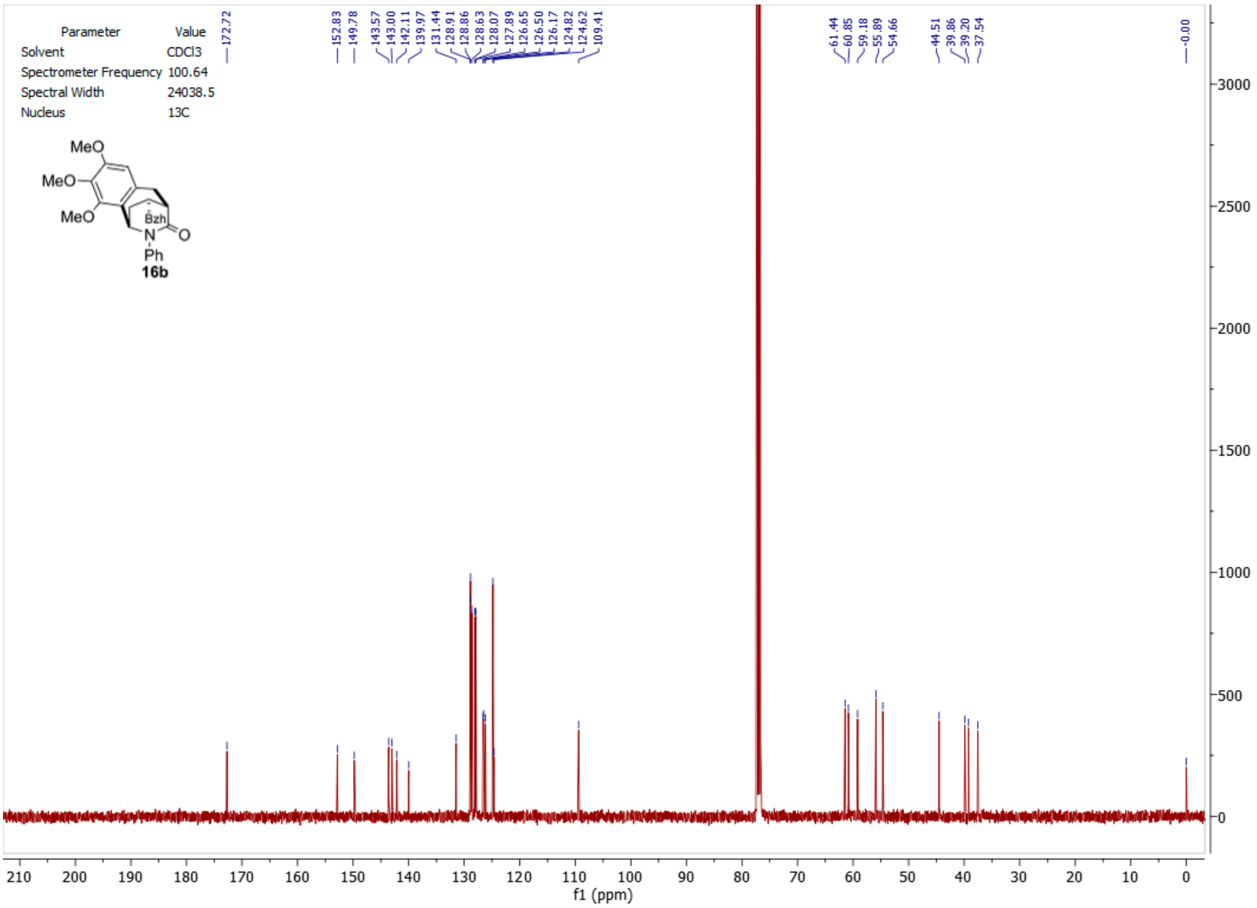

Parameter Value
Solvent CDCl3
Spectrometer Frequency 100.64
Spectral Width 24038.5
Nucleus 13C
MeO
MeO
MeO
Bzh
N
O
Ph
16b
172.72
152.83
149.78
143.57
143.00
142.11
139.97
131.44
128.91
128.86
128.63
128.07
127.89
126.65
126.50
126.17
124.82
124.62
109.41
61.44
60.85
59.18
55.89
54.66
44.51
39.86
39.20
37.54
0.00
3000
2500
2000
1500
1000
500
0
210 200 190 180 170 160 150 140 130 120 110 100 90 80 70 60 50 40 30 20 10 0
f1 (ppm)
